# Clinical importance of simple muscular fitness tests to predict long-term health conditions: a systematic review and meta-analysis of 94 cohort studies

**DOI:** 10.1136/bjsports-2024-109173

**Published:** 2026-02-10

**Authors:** Nuria Marín-Jiménez, Bruno Bizzozero-Peroni, Pablo Molina-Garcia, Francisco B Ortega, Jean-Philippe Chaput, Kai Zhang, Justin J Lang, Ryan McGrath, Grant R Tomkinson, Vicente Martínez-Vizcaíno, Magdalena Cuenca-García, Jose Castro-Piñero

**Affiliations:** 1 Department of Physical Education, GALENO Research Group, University of Cadiz Faculty of Education Sciences, Cadiz, Spain; 2 Instituto de Investigación e Innovación Biomédica de Cádiz (INiBICA), Cádiz, Spain; 3 Department of Education, Faculty of Educational Sciences, Health Research Centre, University of Almería, Almería, Spain; 4 Department of Neurobiology, Care Sciences and Society, Aging Research Center, Karolinska Institutet and Stockholm University, Stockholm, Sweden; 5 Department of Physical Education and Health, Higher Institute of Physical Education, Universidad de la República, Rivera, Uruguay; 6 Health and Social Research Center, Universidad de Castilla La-Mancha, Cuenca, Spain; 7 Instituto de Investigación Biosanitaria ibs, Granada, Spain; 8 Department of Physical Education and Sports, Faculty of Sport Sciences, Sport and Health University Research Institute (iMUDS), University of Granada, Granada, Spain; 9 Faculty of Sport and Health Sciences, University of Jyväskylä, Jyväskylä, Finland; 10 CIBER de Fisiopatología de la Obesidad y Nutrición (CIBEROBN), Instituto de Salud Carlos III, Granada, Spain; 11 Healthy Active Living and Obesity Research Group, Children’s Hospital of Eastern Ontario Research Institute, Ottawa, Ontario, Canada; 12 Department of Pediatrics, Faculty of Medicine, University of Ottawa, Ottawa, Ontario, Canada; 13 School of Human Kinetics, Faculty of Health Sciences, University of Ottawa, Ottawa, Ontario, Canada; 14 Centre for Surveillance and Applied Research, Public Health Agency of Canada, Ottawa, Ontario, Canada; 15 Alliance for Research in Exercise, Nutrition and Activity (ARENA), School of Allied Health and Human Performance, Adelaide University, Adelaide, SA, Australia; 16 Department of Health, Nutrition, and Exercise Sciences, North Dakota State University, Fargo, ND, USA; 17 Department of Geriatrics, University of North Dakota, Grand Forks, ND, USA; 18 Fargo VA Healthcare System, Fargo, ND, USA; 19 Faculty of Health Sciences, Universidad Autónoma de Chile, Talca, Chile

**Keywords:** Health, Physical fitness, Public health, Primary Health Care, Epidemiology

## Abstract

**Objective:**

To examine the predictive validity of field-based muscular strength tests in relation to incident long-term health conditions among adults.

**Design:**

Systematic review and meta-analysis.

**Data sources:**

PubMed, Web of Science, SPORTDiscus, Scopus, CINAHL, Epistemonikos and Google Scholar.

**Eligibility criteria:**

Cohort studies examining the predictive value of validated and/or reliable field-based muscular strength tests in relation to long-term health conditions in adults aged ≥18 years.

**Results:**

A total of 155 studies were included with 94 included in the meta-analysis. Adults with the highest (vs lowest) handgrip strength levels had a lower risk of multiple long-term health conditions (all p<0.05), including cardiovascular diseases (OR=0.73; 95% CI 0.67 to 0.80), type 2 diabetes mellitus (OR=0.79; 95% CI 0.68 to 0.91), musculoskeletal impairment (OR=0.65; 95% CI 0.56 to 0.76), disability (OR=0.57; 95% CI 0.47 to 0.70), anxiety (OR=0.79; 95% CI 0.63 to 0.99), depression (OR=0.70; 95% CI 0.63 to 0.78), cognitive decline (OR=0.57; 95% CI 0.44 to 0.75), dementia (OR=0.62; 95% CI 0.53 to 0.73) and Parkinson’s disease (OR=0.53; 95% CI 0.31 to 0.91). A 5 kg increase in handgrip strength was associated with a lower risk of developing most of these long-term health conditions. In turn, adults with the best (vs worst) performance on the 5-repetition chair-stand test had a lower risk of type 2 diabetes mellitus (OR=0.80; 95% CI 0.72 to 0.88), musculoskeletal impairment (OR=0.52; 95% CI 0.37 to 0.74), disability (OR=0.58; 95% CI 0.41 to 0.82), depression (OR=0.63; 95% CI 0.42 to 0.95), and dementia (OR=0.68; 95% CI 0.54 to 0.85). Every 1 s decrease was associated with 0.94 lower odds of musculoskeletal impairment. The overall quality of the evidence ranged from very low to moderate, indicating limited to moderate confidence in the results.

**Conclusions:**

Our findings suggest increased handgrip strength and chair-stand test performance are associated with a lower risk of multiple long-term health conditions among adults. This research underscores the predictive value of simple field-based muscular strength tests which appear to be clinically useful for adults across different age groups and demographic profiles.

**WHAT IS ALREADY KNOWN ON THIS TOPIC**

- Muscular strength is a significant health marker linked to lower morbidity and improved health outcomes. However, it remains unclear which field-based muscular strength tests are the strongest predictors of incident long-term health conditions among adults.

**WHAT THIS STUDY ADDS**

- This systematic review identified 155 cohort studies, of which 94 were included in the meta-analysis. The handgrip strength test was the most common field-based muscular strength measure for predicting multiple long-term health conditions (n=145 studies), followed by the 5-repetition chair-stand test (n=36).
- The highest levels of handgrip strength were associated with a lower risk of cardiovascular diseases, type 2 diabetes mellitus, musculoskeletal impairment, disability, anxiety, depression, cognitive decline, dementia and Parkinson’s disease. In turn, the best performance on the 5-repetition chair-stand test was associated with a lower risk of type 2 diabetes mellitus, musculoskeletal impairment, disability, depression and dementia.

**HOW THIS STUDY MIGHT AFFECT RESEARCH, PRACTICE OR POLICY**

- Simple field-based muscular strength tests, such as the handgrip strength test and the 5-repetition chair-stand test, should be considered in clinical practice as potential measures to predict a range of long-term health outcomes.

## Introduction

 Non-communicable diseases, such as cardiovascular disease, cancer, chronic respiratory disease and diabetes, account for around 74% of global deaths each year, including 17 million among people under 70.^1^ Importantly, many of these deaths could be prevented or delayed by addressing modifiable risk factors, such as an adequate level of physical fitness.^1^ Muscular strength is widely recognised as a health marker, associated with lower risk of major non-communicable diseases and mortality, and with the potential to signal long-term health conditions.^2^ Thereby, muscular strength assessments have emerged as promising, non-invasive tools in clinical practice, offering a cost-effective and practical means to monitor health status and predict prognosis,^3^ particularly in resource-limited settings. Importantly, although laboratory-based muscular strength assessments can be highly accurate, their feasibility in many clinical settings is limited due to cost, infrastructure and time constraints. Field-based muscular strength tests are simple, feasible tools that require minimal equipment, are quick to administer and can help identify individuals at risk of non-communicable diseases or functional decline.^4^ Therefore, a focused review is needed to determine which of these tests could support health screening in clinical practice.

Previous studies and meta-analyses have mostly focused on determining and quantifying the predictive validity of the field-based muscular strength tests on disease-specific or all-cause mortality.^35^,^10^ While these findings are crucial, the role of field-based muscular strength in predicting long-term health conditions—an equally significant burden for individuals and societies, especially non-communicable diseases—remains less explored. This gap warrants a comprehensive and updated meta-analysis of the link between these tests and future long-term health conditions.

A recent umbrella review^11^ assessing the predictive utility of handgrip strength (HGS) test as a predictor of mortality and morbidity in community-dwelling older people concluded that while the association between HGS test values and all-cause mortality is well established,^35 12^,^14^ it remains to be clarified whether other field-based muscular strength tests may also predict health prognosis. To the best of our knowledge, no previous studies have collected the latest information on the predictive validity of field-based muscular strength tests on multiple long-term health conditions among adults. Therefore, this study aimed to systematically review the evidence on the prognostic value of field-based muscular strength tests for long-term health conditions in adults aged ≥18 years.

## Methods

This systematic review with meta-analysis was conducted under the premises of the Prognosis Research Strategy framework and was structured as overall prognosis research describing the risk of a condition in a given population.^15^ In addition, it was performed according to the Preferred Reporting Items for Systematic Reviews and Meta-Analyses (PRISMA)^16^ guidelines and reported in accordance with the Meta-analysis Of Observational Studies in Epidemiology (MOOSE)^17^ guidelines. The study protocol was registered in PROSPERO (CRD42022324110).

### Data sources and search strategy

PubMed, Web of Science, SPORTDiscus, Scopus, CINAHL and Epistemonikos databases were searched from inception up to 20 November 2024. A subsequent search using the same search strategy was performed in the web search engine Google Scholar (initial 200 references). Additionally, reference lists of included studies were reviewed to find additional eligible studies. The detailed search strategies are shown in the online supplemental appendix 1.

### Eligibility criteria

Longitudinal and prospective/retrospective cohort studies were selected based on the following PECO criteria^18^: (1) Population: general adult population aged ≥18 years at baseline; (2) Exposure: the highest (top extreme category) or higher (continuous variable) levels of valid/reliable field-based muscular strength tests that have shown in recent systematic reviews^19 20^ to have at least moderate validity or reliability in relation to health outcomes; (3) Comparison*:* the lowest level (lower extreme category) of muscular strength tests and (4) Outcomes: specific long-term health conditions were classified and defined following Medical Subject Headings criteria (online supplemental appendix 1,methods) as major chronic diseases (cardiovascular diseases (eg, coronary artery disease, heart failure, stroke), cancer, type 2 diabetes mellitus (T2DM), and respiratory diseases), indicators of functional decline (musculoskeletal impairment (eg, osteoporosis risk, falls, fractures) and disability (eg, disability in activities of daily living, functional mobility, ambulatory status)), common mental disorders (ie, anxiety and depression) operationalised as diagnosed cases or subclinical symptoms, neurodegenerative disorders or conditions (ie, cognitive decline, dementia and Parkinson’s disease), and health-related quality of life. Studies conducted in populations with specific diseases at baseline (ie, diagnosed or self-reported) or special interest groups (eg, military, pregnant women) were excluded. No language, publication date or other restrictions were applied.

### Study selection

The selection process was independently conducted by two researchers (NMJ and PMG) using the Covidence software, achieving an excellent inter-rater agreement (*k*=0.85), indicating strong consistency; and any discrepancies were resolved through discussion and consensus. These two researchers also independently screened all citations, reviewed abstracts for eligibility and extracted data. Any remaining disagreements were resolved by consultation with senior authors (FBO, MC-G and JC-P).

### Data extraction

The following data were extracted: author(s) and year of publication, cohort/project name, participant characteristics at baseline (ie, sample size, mean age and sex distribution), length of follow-up, type of field-based muscular strength test measurement tool, sample categorisation (based on fitness results), long-term health conditions and health outcomes measurement. In addition, effect size estimates (the most fully adjusted risk ratios, HRs and ORs with their corresponding 95% CIs), and covariate adjustments were extracted for meta-analysis synthesis.

### Methodological quality, risk of bias and certainty of evidence

The Newcastle-Ottawa Scale^21^ was used to assess the methodological quality of included cohort studies, through three domains: the selection of participants, the comparability of study groups and the outcome ascertainment. Each domain contains a specific set of items that are subject to evaluation based on the level of risk of bias. The total score for a study could range from 0 to 9, with higher scores indicating higher methodological quality. Based on the total score, studies were categorised as having low (0–3), moderate (4–6) or high (7–9) methodological quality.^22^ In addition, the risk of bias was assessed at the individual study level using the Quality in Prognostic Studies (QUIPS) tool, which evaluates six domains: (1) study participation; (2) study attrition; (3) prognostic factor measurement; (4) outcome measurement; (5) study confounding and (6) statistical analysis and reporting.^23^

The Grading of Recommendations Assessment, Development and Evaluation (GRADE) approach was used to assess the overall certainty of evidence for long-term health conditions.^24^ According to the GRADE guideline, observational studies were initially rated as low quality of evidence, and certain domains could downgrade (ie, overall risk of bias, inconsistency, heterogeneity, indirectness, imprecision and publication bias) or upgrade (ie, large magnitude of effect, dose response gradient and plausible residual confounding) the quality of evidence. We developed consensus-based criteria for downgrading or upgrading GRADE domains (online supplemental appendix 2, table S1). Evidence tables were generated through the web-based GRADEpro software by exporting the results of all analyses (https://www.gradepro.org/),

NOS and risk of bias were applied to all included studies, while GRADE was applied only to those studies included in the meta-analyses. The risk of bias, NOS and GRADE evaluations were independently performed by two reviewers (NMJ and PMG). Discrepancies were resolved through discussion with a third author (JC-P). The reviewers achieved an excellent agreement (*k*=0.94) on the quality assessment prior to the consensus process and reached 100% agreement following a consensus meeting with the involvement of the third investigator (JC-P).

### Exposure harmonisation

To facilitate interpretation and comparison across studies, separate analyses were performed based on whether HGS test in absolute units (kilograms (kg)) was reported as categorical (highest vs lowest category) or continuous (per 5 kg increment) variables. A similar procedure was followed for chair-stand test (CST) performance (five-repetition CST (5-CST)) considering whether the exposure in absolute units (seconds) was reported as categorical (best vs worst category) or continuous (per 1 s decrease). First, the procedures included pooling data comparing the incidence of long-term health conditions between the highest/best and lowest/worst level (reference category=1) of HGS test or 5-CST. Therefore, when the included cohort studies categorised muscular strength into group percentiles, the extreme categories (ie, tertile 3 vs tertile 1, quartile 4 vs quartile 1, quintile 5 vs quintile 1) were compared. Second, data analysing the associations between a 5 kg increase in HGS test or a 1 s decrease in 5-CST and the risk of long-term health conditions were pooled. Cohort studies that reported results according to an increase in the HGS test with values other than 5 kg^25^,^44^ or according to an improvement in 5-CST performance with values other than 1 second^45 46^ were included in the meta-analysis assuming a linear relationship between the exposure as a continuous variable and the incidence of long-term health conditions. For example, if one study^30^ reported an OR of 0.80 for the risk of a certain health condition per 10 kg increment in HGS test, an OR of 0.90 was assumed per 5 kg increment.

### Effect size

The effect size estimators most frequently applied by the included studies were HRs and ORs. Although HR estimates assume proportional hazards (ie, the ratio of the hazard rates in the exposed vs unexposed groups is constant over time), whereas OR estimates do not, HR and OR can be used interchangeably, especially for rare events. Thus, because most cohort studies that reported HR had low population rates of incident cases related to long-term health conditions (ie, <15%), ORs provide a good approximation as a risk ratio estimator.^47^ In addition, ORs cannot be converted to HRs because most of the studies that reported ORs did not provide the number of cases, and an assumed control risk cannot be obtained.^2^ Therefore, the ratio summary statistics for the associations between HGS test or 5-CST performance and the risk of long-term health conditions were jointly included as ORs in our meta-analyses and subjected to log transformations before being analysed.^48^ When studies compared exposure as categorical, the lowest level was set as the reference (ie, equal to 1). OR values less than one indicate a lower risk of long-term health conditions associated with the highest/best (vs lowest/worst) exposure category, per 5 kg increase in HGS test, or per 1 s decrease in 5-CST.

### Data synthesis

Meta-analyses were conducted separately based on whether HGS test in kilograms (kg) and 5-CST performance in seconds were reported as categorical (highest vs lowest category) or continuous variables (per 5 kg increase in HGS and per 1 s decrease in 5-CST). A random effects model with the Sidik-Jonkman method was applied.^49^ Heterogeneity across cohort studies was assessed using the I^2^ metric and the corresponding p values were also considered.^48^ Heterogeneity was classified as not important (0%–40%), moderate (30%–60%), substantial (50%–90%) or considerable (75%–100%).^48^ Forest plots were used to display the pooled ORs with their 95% CIs for the prospective associations between HGS test or 5-CST performance (as categorical or continuous data) and the incidence of long-term health conditions (ie, cardiovascular diseases, cancer, T2DM, respiratory diseases, musculoskeletal impairment, disability, anxiety, depression, cognitive decline, dementia and Parkinson’s disease).

Subgroup analyses were performed according to sex (females vs males), age group (adults (18–64 years) vs older adults (≥65 years)) and region of the study population (Asia vs Europe vs North America). Given the limited availability of stratified results by coexisting health condition status within individual studies, we performed subgroup analyses based on the proportion of participants with at least one co-existing long-term health condition at baseline, distinct from the outcome of interest (lower (<50%) vs higher (≥50%) clinical complexity), to investigate whether this factor modified the associations under study. Sensitivity analyses were performed excluding studies that reported increased or decreased HGS test with values other than 5 kg and including only studies that used a sex-specific cut-off point for clinically relevant muscular weakness according to the Foundation for the National Institutes of Health Sarcopenia Project (HGS test <26 kg for males and <16 kg for females)^50^ and to other specific criteria that applied similar cut-off points (<31 kg for males and <21 kg for females).^1451^,^54^ Additional sensitivity analyses were performed using the leave-one-out method to evaluate the robustness of the summary estimates.^48^

Statistical significance was set at a two-sided p<0.05. All analyses were conducted using the meta^55^ and metafor^56^ packages in the R software and the RStudio environment.

### Methodological considerations

ORs were converted to 1/OR when the highest/best level of HGS test or 5-CST performance was set as the reference group to place all effect sizes in a common frame.^57^ In turn, the same procedure was applied for follow-up studies reporting the influence of a 5 kg decrease in HGS test on the incidence of long-term health conditions. Furthermore, studies from the same epidemiological cohort were included in the corresponding meta-analysis depending on the health-related outcome examined and whether they reported exposure in different units (absolute or relative) or data analysis (categorical or continuous exposure). This strategy allows the largest number of studies to be included without double counting the same participants in each meta-analysis. In turn, when two or more studies examined data from the same cohort, exposure harmonisation and outcome, we performed a three-level random-effects meta-analysis model.^58^ Follow-up studies of the same cohorts have potential sample overlapping and dependency between effect sizes and consequently lead to overconfidence in the results of a meta-analysis.^57^ The multilevel approach accounts for this unit-of-analysis error containing three pooling steps (ie, sampling variance, variance between effect sizes from the same sample and variance between studies) that leads to an overall true effect size, increasing statistical power and providing maximum information from the data.^59^

The multilevel approach was not used when studies examined the same cohort, but one of them analysed the incidence of long-term health conditions excluding participants with the event at baseline (studies included in the meta-analysis) and the others evaluated changes in symptoms without excluding participants with the event at baseline (studies excluded from the meta-analysis). When cohort studies reported HGS test or 5-CST performance as a categorical and continuous variable, data were included according to the appropriate meta-analysis. When the sample size and incident cases of follow-up studies examining exposure levels were not reported for the extreme categories, the total sample size was presented. If the included studies applied adjustment models, those reflecting the maximum extent of adjustment were selected. In those cases where studies stratified the results by sex, we combined the respective measures to calculate a single pooled estimate for each study. In follow-up studies reporting results for >1 follow-up length, the longest period was selected.^60^,^63^ Furthermore, in studies that provided results on the incidence of long-term health conditions according to exposure categories only graphically (ie, without specifying numerical data), risk estimates were extracted using the WebPlotDigitizer software.^64^ Finally, studies that analysed HGS test or 5-CST performance as a continuous variable but did not specify the increase or decrease value were not included in the respective meta-analyses.

### Equity, diversity and inclusion statement

Our research team encompasses equity across sex and diversity across countries, professions and career stages, including junior, mid-career and senior researchers. The study population is sex-balanced and includes a spectrum of ages, geographic locations and socioeconomic statuses that span the general adult population without relying entirely on specific conditions or special activities. When information was available, the results were stratified by age, geographic location and sex, which allowed us to analyse the generalisability of the results.

## Results

### Study selection

**Figure 1:**
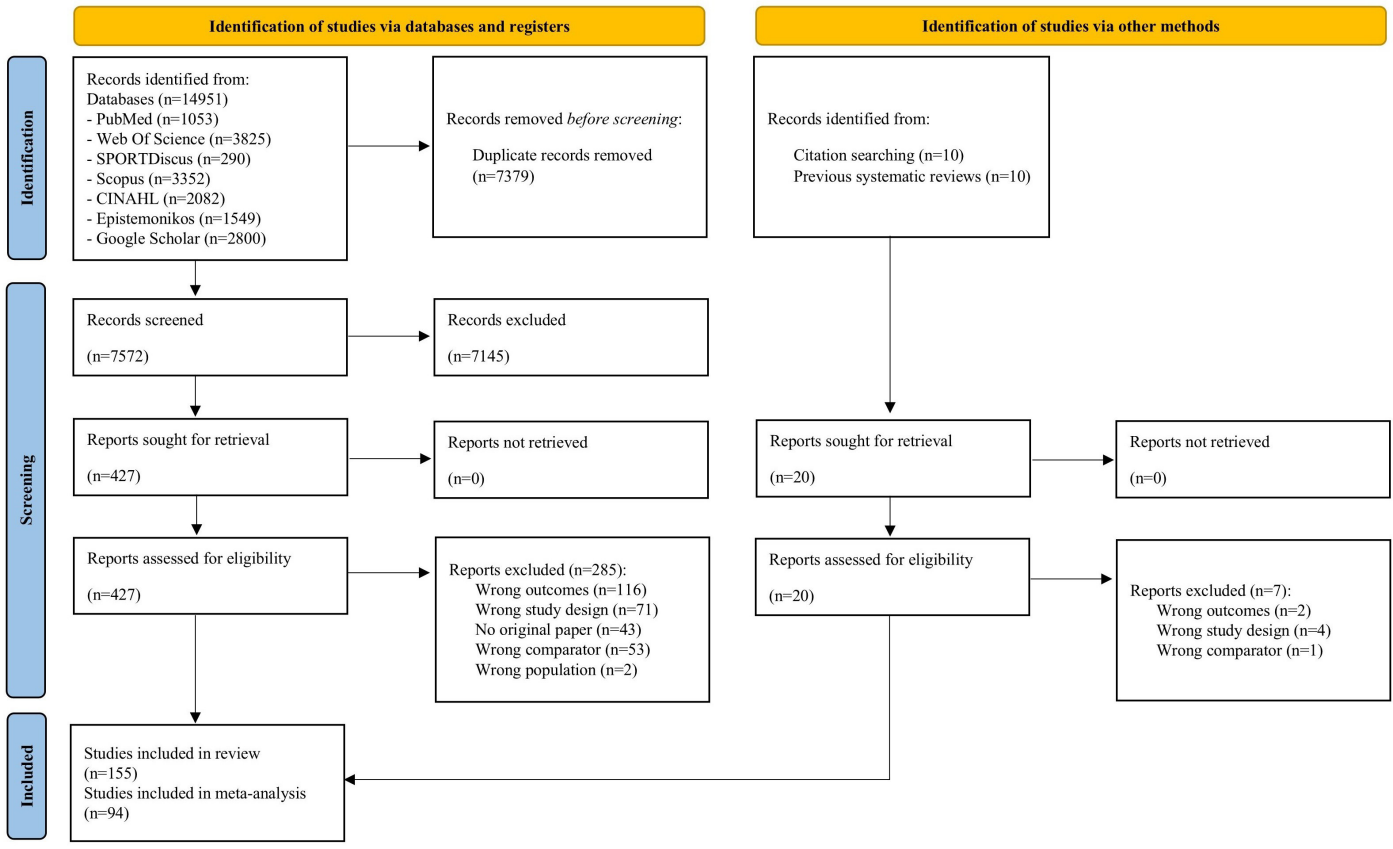
PRISMA 2020 flow diagram of the study selection process. PRISMA, Preferred Reporting Items for Systematic Reviews and Meta-Analyses.

The PRISMA flowchart is provided in figure 1. The electronic search identified 14 951 records, with 20 additional references from other sources. After screening, 155 longitudinal studies were included in the systematic review, and 94 were meta-analysed. A total of 292 full-text articles were excluded, mainly for not using a field-based muscular strength test as the exposure or for applying cross-sectional or case–control designs (online supplemental appendix 1, results).

### Methodological quality, risk of bias and certainty of evidence

All studies scored at least a ‘5’ on the Newcastle-Ottawa quality assessment criteria, with 92.6% of studies rated over 7 (ie, high methodological quality) and the mean score being 8 (online supplemental appendix 2, table S2). According to the QUIPS assessment, most included studies presented an overall low risk of bias. Low risk was generally observed across all six domains, with some concerns identified in domain 5 (study confounding), mainly related to the limited control for potential residual confounders inherent with observational prognostic research. Additionally, minor concerns were observed in some studies for domain 4 (outcome measurement), particularly those evaluating musculoskeletal impairment, and to a lesser extent cardiovascular disease and disability, due to the use of self-reported outcomes (ie, symptom-based reports) that were not always based on validated or clinically confirmed measures. Overall, the body of evidence from studies specifically included in GRADE was considered to have a low risk of bias. The risk of bias summary figures were generated using the robvis tool^65^ (https://mcguinlu.shinyapps.io/robvis/) and are presented in online supplemental appendix 3, figures S1–12).

According to the GRADE approach, the certainty of the evidence was assessed as low or very low for most long-term health conditions, except for T2DM and dementia, which showed moderate certainty in both muscular strength tests (tables1  2 and online supplemental appendix 2, table S3 and S4).

**Table 1:** Certainty of the evidence for the handgrip strength test assessed using the GRADE tool

No of studies	Certainty assessment						Effect			Certainty
	Study design	Risk of bias	Inconsistency	Indirectness	Imprecision	Other considerations	No of events	No of individuals	Rate(95% CI)	
Cardiovascular disease										
15	cohort studies	not serious	serious*	not serious	not serious	dose response gradient	44 496	568 975	even rate 0.7% (0.67% to 0.8%)	⨁⨁◯◯Low*
Cancer										
3	cohort studies	not serious	not serious	not serious	serious†	dose response gradient	30 985	580 162	even rate 1.0% (0.96% to 1.1%)	⨁⨁◯◯Low†
Type 2 diabetes mellitus										
8	cohort studies	not serious	not serious	not serious	not serious	dose response gradient	10 336	248 804	even rate 0.8% (0.68% to 0.91)	⨁⨁⨁◯Moderate
Respiratory diseases										
2	cohort studies	not serious	very serious‡	not serious	serious†	dose response gradient	12 700	593 329	even rate 0.9% (0.78% to 1.07%)	⨁◯◯◯Very low†‡
Musculoskeletal impairment										
10	cohort studies	serious§	serious¶	not serious	not serious	dose response gradient	3197	68 777	even rate 0.7% (0.56% to 0.76%)	⨁◯◯◯Very low§¶
Disability (disability in activities of daily living, functional mobility, ambulatory status)										
13	cohort studies	not serious	not serious	serious**	not serious	dose response gradient	15 406	88 458	even rate 0.8% (0.63% to 0.99%)	⨁⨁◯◯Low**
Anxiety (diagnosis or moderate to severe symptoms)										
3	cohort studies	not serious	not serious	serious**	not serious	dose response gradient	6742	167 438	even rate 0.7% (0.63% to 0.78%)	⨁⨁◯◯Low**
Depression (diagnosis or moderate to severe symptoms)										
9	cohort studies	not serious	serious††	serious**	not serious	dose response gradient	18 079	453 401	even rate 0.7% (0.44% to 0.75%)	⨁◯◯◯ Very low**††
Cognitive decline										
8	cohort studies	not serious	serious‡‡	serious**	not serious	dose response gradient	1570	25 691	even rate 0.7% (0.67% to 0.8%)	⨁◯◯◯Very low**‡‡
Dementia										
5	cohort studies	not serious	not serious	not serious	not serious	dose response gradient	2905	251 626	even rate 0.6% (0.53% to 0.73%)	⨁⨁⨁◯Moderate
Parkinson’s disease										
3	cohort studies	not serious	very serious§§	not serious	serious¶¶	dose response gradient	1810	358 791	even rate 0.5% (0.31% to 0.91%)	⨁◯◯◯Very low§§¶¶

Publication bias: long-term health conditions with ≥10 studies showed a funnel plot symmetry and Egger’s test p≥0.05. It was assumed that search strategy appeared comprehensive in long-term health conditions with <10 studies.

One level of certainty was upgraded owing to the presence of a dose–response gradient.

* I2=50.9% and CIs overlap.

† Sample size >4000, CIs include 1, but exclude important harm (ie, 10%).

‡ I2=97%, point estimates vary widely across studies and CIs show no overlap.

§ >20% studies included were assessed an overall moderate RoB, and no studies as high RoB.

¶ I2=51.6, results are consistent in direction and CIs minimal overlap.

** Good global representation, standardised and validated protocols to assess HG, but surrogate outcome.

†† I2=52.1%, results are consistent in direction and magnitude, and CIs overlap.

‡‡ I2=73.5%, results are consistent in direction and CIs have minimal overlap.

§§ I2=89%, point estimates vary widely across studies and CIs show no overlap.

¶¶ Sample size >4000, CIs do not include 1, but large 95% CI (upper/lower>2).

HG, handgrip strength; RoB, risk of bias.

**Table 2:** Certainty of the evidence for the 5-chair-stand test assessed using the GRADE tool

No of studies	Certainty assessment						Effect			Certainty
	Study design	Risk of bias	Inconsistency	Indirectness	Imprecision	Other considerations	No of events	No of individuals	Rate(95% CI)	
Type 2 diabetes mellitus										
2	non-randomised studies	not serious	not serious	not serious	not serious	dose response gradient	1866	23 578	event rate 0.8% (0.72% to 0.88%)	⨁⨁⨁◯Moderate
Musculoskeletal impairment										
6	non-randomised studies	serious*	not serious	not serious	not serious	dose response gradient	2325	16 172	event rate 0.5% (0.37% to 0.74%)	⨁⨁◯◯Low*
Disability (disability in activities of daily living, functional mobility, ambulatory status)										
5	non-randomised studies	not serious	not serious	serious†	not serious	dose response gradient	2112	5561	event rate 0.6% (0.41% to 0.82%)	⨁⨁◯◯Low†
Depression (diagnosis or moderate to severe symptoms)										
2	non-randomised studies	not serious	serious‡	not serious	serious§	dose response gradient	983	3578	event rate 0.6% (0.42% to 0.95%)	⨁◯◯◯Very low‡§
Dementia (diagnosis or moderate to severe symptoms)										
2	non-randomised studies	not serious	not serious	not serious	not serious	dose response gradient	569	7753	event rate 0.7% (0.54% to 0.85%)	⨁⨁⨁◯Moderate

Publication bias: long-term health conditions with ≥10 studies showed a funnel plot symmetry and Egger’s test p≥0.05. It was assumed that search strategy appeared comprehensive in long-term health conditions with <10 studies.

One level of certainty was upgraded owing to the presence of a dose–response gradient.

* >20% studies included were assessed an overall moderate RoB and no studies as high RoB.

† Good global representation, standardised and validated protocols to assess HG, but surrogate outcome.

‡ I2=72% and results are consistent in direction and CIs overlap.

§ Sample size <4000 and large 95% CI interval (upper/lower >2).

GRADE, Grading of Recommendations Assessment, Development and Evaluation; HG, handgrip strength; RoB, risk of bias.

### Study characteristics

Online supplemental appendix 2, table S5 summarises the characteristics of the 155 included studies, published from 1999 to 2024. Sample sizes ranged from 24 to 502 293 participants, being most of them recruited from the UK Biobank, The China Health and Retirement Longitudinal Study, and the Korean Longitudinal Study of Ageing. The lowest mean cohort age at baseline was 18 years, and the oldest was ≥90 years. The follow-up duration ranged from 1.5 to 25.3 years.

The outcomes were distributed in 12 long-term health conditions: cardiovascular diseases (n=18),^1425 41 44 66^,^79^ cancer (n=6),^14 26 66 68 80 81^ T2DM (n=22),^1427 28 33 34 62 82^,^97^ respiratory diseases (n=3),^14 66 68^ musculoskeletal impairment (n=30),^1432 42 43 46 60 61 78 98^,^119^ disability (n=31),^2935 37 38 63 106 120^,^143^ anxiety, assessed either as a clinical diagnosis (generalised, phobic and other anxiety disorders) or as moderate to severe self-reported symptoms (n=3),^144^,^146^ depression, assessed either as a clinical diagnosis (single episodes and recurrent depression, across mild, moderate, severe and unspecified types) or as mild to severe self-reported symptoms (n=17),^3031 35 142 144 145 147^,^157^ health-related quality of life (n=4),^123158^,^160^ cognitive decline (n=26),^3536 39 40 142 161^,^181^ dementia (n=12),^25137 178 181^,^189^ and Parkinson’s disease (n=3).^190^,^192^

No other field-based muscular strength tests were identified apart from the HGS test and the CST. The HGS test was the most studied (n=145),^1425^,^44 46 60^ followed by the CST (n=36).^3236 46 60 78 84 86 95 98^,^100 102 107^ For the HGS test, most studies used a Jamar handgrip dynamometer, with assessments based on the highest or mean value across two or three attempts, expressed primarily in kilograms or bars. Regarding the CST, the most commonly used protocol was the 5-repetition version (n=30),^3236 46 60 78 84 86 95 98^,^100 107 111 118^ followed by other less frequently used versions, including the 30 s version (n=3),^108 109 159^ 10 repetitions (n=1),^126^ 3 repetitions (n=1)^128^ and 1 repetition (n=1).^102^

### Meta-analysis

Cohort studies that reported HGS test results in non-kg equivalent measurements (eg, unit of torque such as newton-metre or unit of pressure such as bar, kg/centimetres^2^ or kilopascal) were not included in the meta-analyses of absolute HGS test in kg to facilitate interpretation and comparison across studies. In turn, due to the small number of studies and the heterogeneity of measures, no additional meta-analysis was performed for these quantifications of HGS test (online supplemental appendix 2, table S5). Similarly, studies that reported the results of 5-CST performance on units other than seconds (ie, repetitions/s) were not included in the meta-analyses of 5-CST.

### Highest (vs lowest) category of HGS test

**Figure 2:**
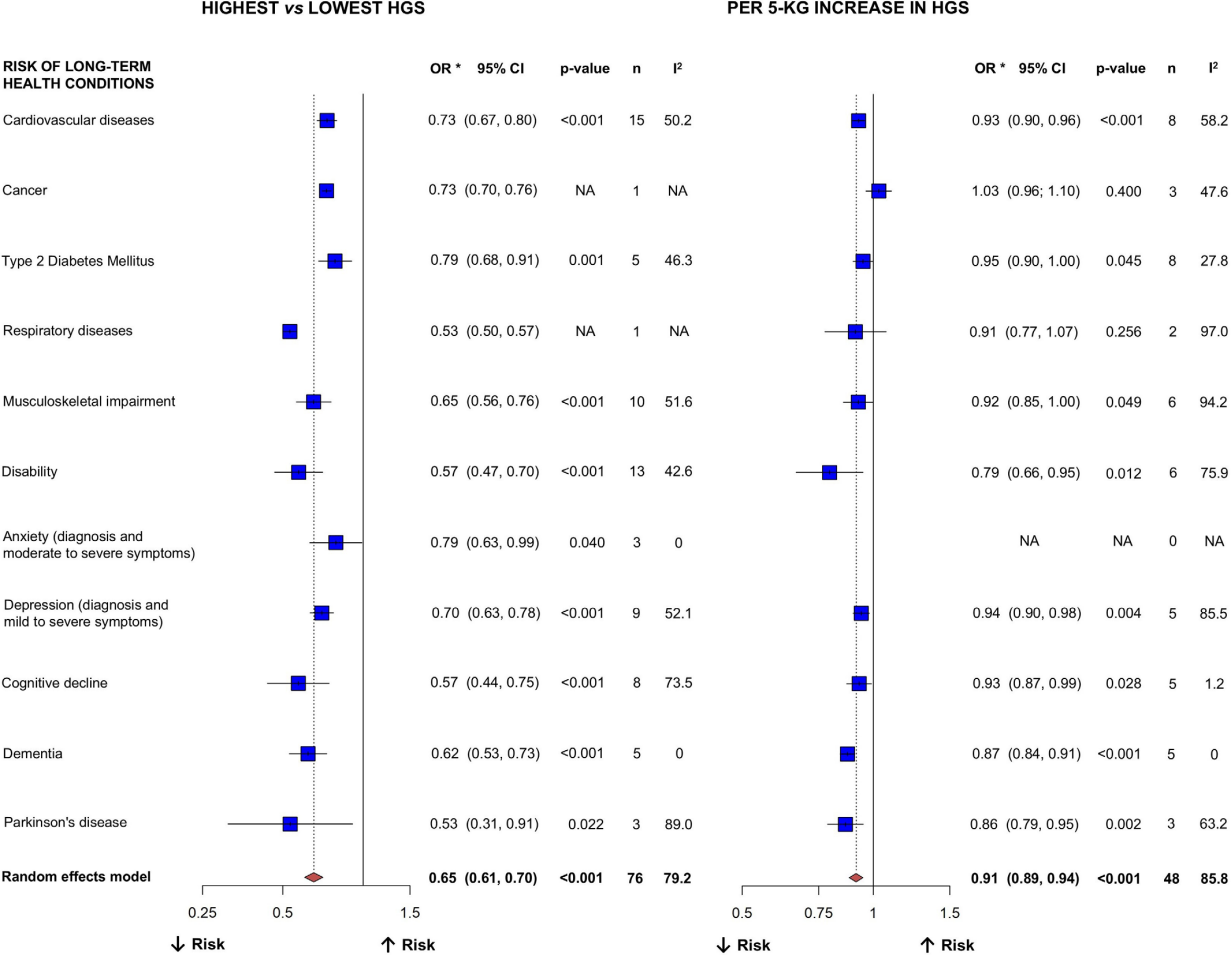
Pooled ORs of associations between handgrip strength levels and incident long-term health conditions. n, number of studies relating HGS with each of the outcomes; I^2^, used to classify heterogeneity across follow-up studies as not important (0%–40%), moderate (30%–60%), substantial (50%–90%) or considerable (75%–100%); Notes: High handgrip strength was defined as the highest group and low handgrip strength as the lowest group (ie, tertile 3 vs tertile 1, quartile 4 vs quartile 1 or quintile 5 vs quintile 1). Residual heterogeneity may reflect differences in the cut-off values used to define handgrip strength categories across the included studies. *Pooled ORs derived from all the studies investigating each long-term health condition. Online supplemental figures 13 and 14 provide the specific studies included in each long-term health condition. Long-term health conditions (online supplemental table S2) provide the specific outcomes that were classified and defined following Medical Subject Headings criteria: Cardiovascular diseases, ‘pathological conditions involving the cardiovascular system including the heart; the blood vessels; or the pericardium’; Cancer (classified as Neoplasms), ‘new abnormal growth of tissue. Malignant neoplasms show a greater degree of anaplasia and have the properties of invasion and metastasis, compared with benign neoplasms’; type 2 diabetes mellitus, ‘a subclass of diabetes mellitus that is not insulin-responsive or dependent’; Respiratory diseases (classified as respiratory tract diseases), ‘diseases involving the respiratory system’; Musculoskeletal impairment (classified as musculoskeletal diseases), ‘diseases of the muscles and their associated ligaments and other connective tissue and of the bones and cartilage viewed collectively’; Disability (classified as mobility limitation), ‘difficulty in walking from place to place’; Anxiety (clinical diagnosis or symptom severity), ‘persistent and disabling anxiety, or feelings or emotions of dread, apprehension and impending disaster’; Depression (clinical diagnosis or symptom severity), ‘depressive states usually of moderate intensity, in contrast with major depressive disorder present in neurotic and psychotic disorders; an affective disorder manifested by either a dysphoric mood or loss of interest or pleasure in usual activities, in which the mood disturbance is prominent and relatively persistent, with major depression defined as a disorder in which five (or more) symptoms have been present during the same 2-week period and represent a change from previous functioning, with at least one symptom being depressed mood or loss of interest or pleasure’; Cognitive decline (classified as cognitive dysfunction), ‘diminished or impaired mental and/or intellectual function’; Dementia, ‘an acquired organic mental disorder with loss of intellectual abilities of sufficient severity to interfere with social or occupational functioning. The dysfunction is multifaceted and involves memory, behaviour, personality, judgement, attention, spatial relations, language, abstract thought and other executive functions. The intellectual decline is usually progressive and initially spares the level of consciousness; and Parkinson’s disease, ‘a progressive, degenerative neurologic disease characterised by a tremor that is maximal at rest, retropulsion (ie, a tendency to fall backwards), rigidity, stooped posture, slowness of voluntary movements and a masklike facial expression’. HGS, handgrip strength; NA, not applicable.

A total of 58 independent comparisons (derived from 76 outcome-specific estimates) between the highest (vs lowest) category of HGS test and long-term health conditions from 64 cohort studies (mean age range: 50.0–84.7 years) were included in the meta-analysis. The studies for each long-term health condition, along with their applied cut-off points for defining the highest and lowest levels of HGS test, are displayed in (online supplemental appendix 3, figure S13). All included studies provided data for HGS test categories by standardising the cut-off point for high and low levels according to the participant’s sex or adjusting for this variable. Higher HGS was consistently associated with a lower risk of several long-term health conditions (all p<0.05), including cardiovascular diseases (OR=0.73), T2DM (OR=0.79), musculoskeletal impairment (OR=0.65), disability (OR=0.57), anxiety (OR=0.79), depression (OR=0.70), cognitive decline (OR=0.57), dementia (OR=0.62) and Parkinson’s disease (OR=0.53). Detailed estimates are provided in figure 2.

### Per 5-kg increase in HGS test

A total of 44 independent comparisons (derived from 48 outcome-specific estimates) between a 5 kg increase in HGS test and long-term health conditions from 41 cohort studies (mean age range: 37.2–85.0 years) were included in the meta-analysis. The studies within each long-term health condition are detailed in (online supplemental appendix 3,figure S14). A 5 kg increase in HGS was associated with a lower risk of several long-term health conditions (all p<0.05), including cardiovascular diseases (OR=0.93), T2DM (OR=0.95), musculoskeletal impairment (OR=0.92), disability (OR=0.79), depression (OR=0.94), cognitive decline (OR=0.93), dementia (OR=0.87) and Parkinson’s disease (OR=0.86). No significant associations were observed for cancer (OR=1.03) or respiratory diseases (OR=0.91). Detailed estimates are presented in figure 2.

### CST performance

**Figure 3:**
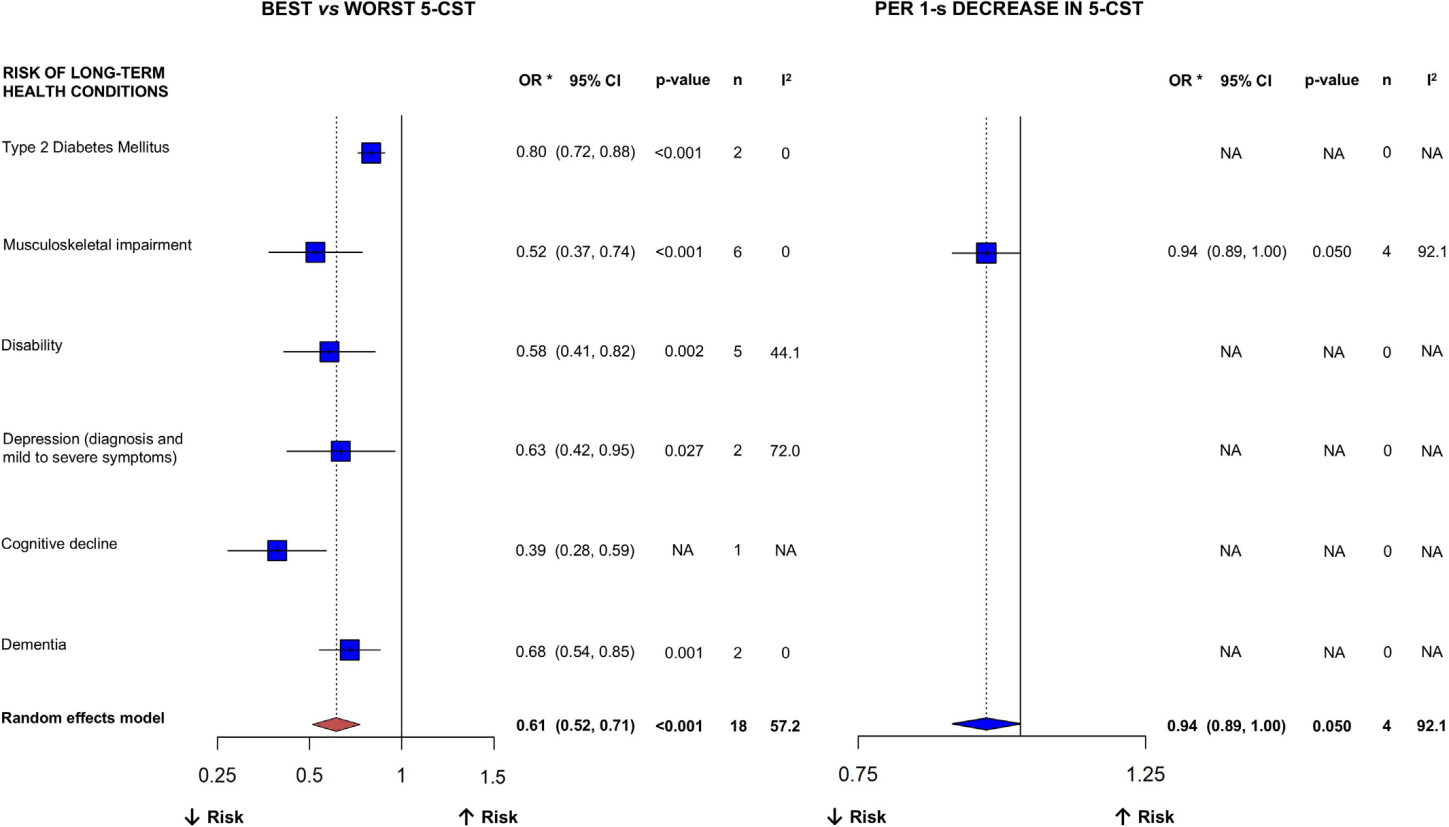
Pooled ORs of associations between 5-repetition chair-stand performance and incident long-term health conditions. n, number of studies relating 5-CST with each of the outcomes; I^2^, used to classify heterogeneity across follow-up studies as not important (0%–40%), moderate (30%–60%), substantial (50%–90%) or considerable (75%–100%). Notes: Best 5-repetition chair-stand performance was defined as the highest group and worst 5-repetition chair-stand performance as the lowest group (ie, tertile 3 vs tertile 1, quartile 4 vs quartile 1, quintile 5 vs quintile 1). Residual heterogeneity may reflect differences in the cut-off values used to define chair-stand performance categories across the included studies. *Pooled ORs derived from all the studies investigating each long-term health condition. Online supplemental figures 15 and 16 provide the specific studies included in each long-term health condition. Long-term health conditions (online supplemental table S2) provide the specific outcomes that were classified and defined following Medical Subject Headings criteria: Type 2 diabetes mellitus, ‘a subclass of diabetes mellitus that is not insulin-responsive or dependent’; Musculoskeletal impairment (classified as musculoskeletal diseases) ‘diseases of the muscles and their associated ligaments and other connective tissue and of the bones and cartilage viewed collectively’; Disability (classified as mobility limitation), ‘difficulty in walking from place to place’; Depression (clinical diagnosis or symptom severity), ‘depressive states usually of moderate intensity, in contrast with major depressive disorder present in neurotic and psychotic disorders; an affective disorder manifested by either a dysphoric mood or loss of interest or pleasure in usual activities, in which the mood disturbance is prominent and relatively persistent, with major depression defined as a disorder in which five (or more) symptoms have been present during the same 2-week period and represent a change from previous functioning, with at least one symptom being depressed mood or loss of interest or pleasure’; Cognitive decline (classified as cognitive dysfunction), ‘diminished or impaired mental and/or intellectual function’ and Dementia, ‘an acquired organic mental disorder with loss of intellectual abilities of sufficient severity to interfere with social or occupational functioning. The dysfunction is multifaceted and involves memory, behaviour, personality, judgement, attention, spatial relations, language, abstract thought and other executive functions. The intellectual decline is usually progressive and initially spares the level of consciousness’. 5-CST, 5-repetition chair-stand test; NA, not applicable.

The results of the meta-analysis according to the 5-CST are shown in figure 3. A total of 16 cohort studies comparing the best (vs worst) category of 5-CST performance and long-term health conditions (mean age range: 57.6–84.2 years) were included in the meta-analysis. The studies within each long-term health condition, along with their applied cut-off points for defining the best and worst levels of 5-CTS, are displayed in online supplemental appendix 3, figure S15. The studies applied different cut-off points for defining the best (ie, ranging from <6.8 to <17.0 s) and worst (ie, ranging from ≥11.2 to ≥17.0 s) levels of 5-CST. Better performance in the 5-CST was associated with a lower risk of several long-term health conditions (all p<0.05), including T2DM (OR=0.80), musculoskeletal impairment (OR=0.52), disability (OR=0.58), depression (OR=0.63) and dementia (OR=0.68). When analysed as a continuous variable (online supplemental appendix 3, figure S16), a 1 s decrease on the 5-CST was associated with a lower risk of musculoskeletal impairment (OR=0.94).

Further details of the meta-analysis results are presented in online supplemental appendix 1.

### Subgroup and sensitivity analyses

The protective associations between higher HGS and most long-term health conditions were generally consistent across sex and age groups, with similar risk reductions observed for cardiovascular diseases, T2DM, musculoskeletal impairment, disability and depression (tables3  4). Some variation was observed by geographic region and baseline clinical complexity (online supplemental appendix 2, tables S6 and S7).

**Table 3:** Subgroup analyses according to participant sex for the prospective associations between handgrip strength test and long-term health conditions

Highest vs lowest category	Females				Males			
	N	OR (95% CI)	P value	I^2^ (%)	N	OR (95% CI)	P value	I^2^ (%)
Cardiovascular diseases^41 66 69 70 75 79^	8	**0.72 (0.62 to 0.83)**	<0.001	84.4	8	**0.73 (0.63 to 0.85)**	<0.001	77.4
Cancer^66^	1*	**0.72 (0.68 to 0.76)**	NA	NA	1*	**0.74 (0.70 to 0.78)**	NA	NA
Type 2 diabetes mellitus^82 83 89 94^	4	**0.83 (0.70 to 0.98)**	0.033	0	4	**0.75 (0.60 to 0.93)**	0.010	14.7
Respiratory diseases^66^	1*	**0.53 (0.48 to 0.57)**	NA	NA	1*	**0.54 (0.49 to 0.58)**	NA	NA
Musculoskeletal impairment^42 43 61 100 111^	4	**0.67 (0.56 to 0.81)**	<0.001	13.0	3	**0.49 (0.38 to 0.65)**	<0.001	0
Disability (disability ADL, functional mobility, ambulatory status)^63 125 132 138 141^	4	**0.54 (0.41 to 0.71)**	<0.001	52.2	4	**0.60 (0.50 to 0.72)**	<0.001	15.2
Depression (diagnosis or mild to severe symptoms)^30 153 156^	3	**0.68 (0.50 to 0.93)**	0.015	61.9	3	**0.82 (0.67 to 0.99)**	0.042	0
Cognitive decline^165 167 174 180^	4	**0.60 (0.38 to 0.93)**	0.024	53.6	4	0.70 (0.47 to 1.05)	0.082	0
Dementia^187^	2*	**0.78 (0.64 to 0.94)**	NA	NA	2*	0.84 (0.68 to 1.05)	NA	NA
Parkinson’s disease^190 192^	2*	**0.68 (0.47 to 1.00)**	NA	NA	2*	**0.72 (0.60 to 0.85)**	NA	NA
Random effects model	32	**0.68 (0.62 to 0.74)**	<0.001	77.8	34	**0.69 (0.63 to 0.75)**	<0.001	73.8

Per 5 kg increment	n	OR (95% CI)	P value	*I*^2^ (%)	n	OR (95% CI)	P value	*I*^2^ (%)
Cardiovascular diseases^41 66^	2	0.92 (0.82 to 1.02)	0.108	98.1	2	0.94 (0.87 to 1.01)	0.083	98.2
Cancer^26 66^	2*	0.95 (0.86 to 1.05)	NA	NA	2*	0.99 (0.90 to 1.09)	NA	NA
Type 2 diabetes mellitus^27 33 82 90^	3	0.97 (0.87 to 1.08)	0.551	56.8	4	**0.92 (0.87 to 0.97)**	0.002	7.8
Respiratory diseases^66^	1	**0.82 (0.80 to 0.84)**	NA	NA	1	**0.85 (0.84 to 0.87)**	NA	NA
Musculoskeletal impairment^32 42 43 61 107^	4	**0.90 (0.81 to 0.99)**	0.030	77.2	4	**0.92 (0.86 to 0.99)**	0.022	86.0
Disability (disability ADL, functional mobility, ambulatory status)^63 136^	2	**0.70 (0.52 to 0.95)**	0.021	54.2	1	**0.86 (0.80 to 0.93)**	NA	NA
Depression (diagnosis or mild to severe symptoms)^30 144 153^	3	**0.94 (0.91 to 0.98)**	0.001	0	3	**0.93 (0.91 to 0.95)**	<0.001	0
Cognitive decline^165 174 175^	3	0.83 (0.63 to 1.09)	0.176	76.7	3	0.98 (0.88 to 1.08)	0.645	0
Dementia^183 184 187^	3*	**0.89 (0.84 to 0.94)**	NA	NA	3*	**0.87 (0.83 to 0.90)**	NA	NA
Parkinson’s disease^190^,^192^	3	**0.87 (0.84 to 0.91)**	<0.001	0	3	**0.86 (0.75 to 0.98)**	0.021	69.4
Random effects model	26	**0.89 (0.85 to 0.94)**	<0.001	89.9	26	**0.91 (0.89 to 0.94)**	<0.001	90.6

Bold values indicate a statistically significant association (p<0.05). N represents the number of studies included in each subgroup analysis. Citations from both groups are presented together. For detailed study characteristics, please refer to online supplemental table S2).

Notes: Long-term health conditions (online supplemental table S2 provides the specific outcomes) were classified and defined following Medical Subject Headings (MeSH) criteria when available. Cardiovascular diseases, ‘pathological conditions involving the cardiovascular system including the heart; the blood vessels; or the pericardium’; Cancer (classified as neoplasms), ‘new abnormal growth of tissue. Malignant neoplasms show a greater degree of anaplasia and have the properties of invasion and metastasis, compared with benign neoplasms’; Type 2 diabetes mellitus, ‘a subclass of diabetes mellitus that is not insulin-responsive or dependent’; Respiratory diseases (classified as respiratory tract diseases), ‘diseases involving the respiratory system’; Musculoskeletal impairment (classified as musculoskeletal diseases) ‘diseases of the muscles and their associated ligaments and other connective tissue and of the bones and cartilage viewed collectively’; Disability (classified as mobility limitation), ‘difficulty in walking from place to place’; Depression (diagnosis or symptom severity), ‘depressive states usually of moderate intensity in contrast with major depressive disorder present in neurotic and psychotic disorders; an affective disorder manifested by either a dysphoric mood or loss of interest or pleasure in usual activities, in which the mood disturbance is prominent and relatively persistent, with major depression defined as a disorder in which five (or more) symptoms have been present during the same 2-week period and represent a change from previous functioning, with at least one symptom being depressed mood or loss of interest or pleasure’; Cognitive decline, (classified as cognitive dysfunction) ‘diminished or impaired mental and/or intellectual function’; Dementia, ‘an acquired organic mental disorder with loss of intellectual abilities of sufficient severity to interfere with social or occupational functioning. The dysfunction is multifaceted and involves memory, behaviour, personality, judgement, attention, spatial relations, language, abstract thought and other executive functions. The intellectual decline is usually progressive and initially spares the level of consciousness; and Parkinson’s disease, ‘a progressive, degenerative neurologic disease characterised by a tremor that is maximal at rest, retropulsion (ie, a tendency to fall backwards), rigidity, stooped posture, slowness of voluntary movements, and a masklike facial expression’.

* A meta-analysis was not conducted on studies from the same cohort (n=1 comparison).

ADL, activities of daily living; NA, not applicable.

**Table 4:** Subgroup analyses according to adult age group for the prospective associations between handgrip strength test and long-term health conditions

Highest vs lowest category	Middle-aged adults (<65 years)				Older adults (≥65 years)			
	N	OR (95% CI)	P value	I^2^ (%)	N	OR (95% CI)	P value	I^2^ (%)
Cardiovascular diseases^2541 44 66 67 69 70 72^,^75 77 79^	12	**0.70 (0.64 to 0.76)**	<0.001	0	4	**0.80 (0.74 to 0.88)**	<0.001	0
Cancer^66^	1	**0.73 (0.70 to 0.76)**	NA	NA	0	NA	NA	NA
Type 2 diabetes mellitus^82 83 89 93 94^	4	**0.79 (0.67 to 0.94)**	0.006	57.1	1	0.70 (0.46 to 1.06)	NA	NA
Respiratory diseases^66^	1	**0.53 (0.50 to 0.57)**	NA	NA	0	NA	NA	NA
Musculoskeletal impairment^42 43 61 100 111 112 114 124^	3	**0.63 (0.50 to 0.81)**	<0.001	59.7	5	**0.58 (0.49 to 0.68)**	<0.001	0
Disability (disability ADL, functional mobility, ambulatory status)^63124^,^126 130 132^	5	**0.64 (0.56 to 0.74)**	<0.001	73.4	8	**0.58 (0.43 to 0.78)**	<0.001	68.9
Anxiety (diagnosis or moderate to severe symptoms)^144^,^146^	3	**0.79 (0.63 to 0.99)**	0.040	0	0	NA	NA	NA
Depression (diagnosis or mild to severe symptoms)^30 31 142 144 149 150 152 153 156^	7	**0.71 (0.64 to 0.79)**	<0.001	60.3	3	**0.71 (0.52 to 0.97)**	0.033	53.2
Cognitive decline^142161 164^,^167 174 180^	3	**0.71 (0.61 to 0.83)**	<0.001	0	6	**0.56 (0.40 to 0.79)**	0.001	75.8
Dementia^25 182 185 187^	3	0.76 (0.55 to 1.06)	0.105	23.9	4	**0.69 (0.51 to 0.92)**	0.011	78.6
Parkinson’s disease^191 192^	1	**0.76 (0.62 to 0.92)**	NA	NA	2	0.59 (0.28 to 1.25)	0.172	94.6
Random effects model	43	**0.70 (0.67 to 0.74)**	<0.001	78.3	33	**0.63 (0.56 to 0.71)**	<0.001	78.3

Per 5 kg increment	n	OR (95% CI)	P value	I^2^ (%)	n	OR (95% CI)	P value	I^2^ (%)
Cardiovascular diseases^14 25 41 44 66 73 77 79^	8	**0.92 (0.88, 0.95)**	<0.001	81.2	3	**0.89 (0.84, 0.94)**	<0.001	0
Cancer^14 26 66^	3	1.03 (0.95 to 1.11)	0.475	44.5	1	**0.92 (0.88 to 0.96)**	NA	NA
Type 2 diabetes mellitus^14 27 28 33 34 62 82 90^	7	**0.93 (0.88 to 0.97)**	0.003	33.3	1	1.01 (0.86 to 1.19)	NA	NA
Respiratory diseases^14 66^	2	0.91 (0.77 to 1.07)	0.255	98.1	1	**0.84 (0.81 to 0.87)**	NA	NA
Musculoskeletal impairment^14 32 42 43 61 107^	4	0.92 (0.80 to 1.04)	0.191	94.3	3	**0.95 (0.91 to 0.98)**	0.007	63.8
Disability (disability ADL to functional mobility to ambulatory status)^29 35 37 38 63 136^	2	0.76 (0.46 to 1.26)	0.289	82.1	4	**0.79 (0.66 to 0.96)**	0.016	76.6
Depression (diagnosis or mild to severe symptoms)^30 31 35 144 153^	4	**0.92 (0.90 to 0.95)**	<0.001	17.7	2	0.94 (0.82 to 1.08)	0.396	95.2
Cognitive decline^35 40 165 174 175^	3	0.93 (0.85 to 1.02)	0.148	0	4	0.93 (0.86 to 1.00)	0.055	30.9
Dementia^25 39 183 184 187^	3	**0.83 (0.75 to 0.93)**	0.001	0	5	**0.88 (0.83 to 0.92)**	<0.001	0
Parkinson’s disease^191 192^	1	**0.82 (0.73 to 0.92)**	NA	NA	2	**0.86 (0.78 to 0.96)**	0.005	63.4
Random effects model	37	**0.92 (0.89 to 0.95)**	<0.001	90.6	26	**0.89 (0.85 to 0.93)**	<0.001	86.9

Bold values indicate a statistically significant association (p<0.05). N represents the number of studies included in each subgroup analysis. Citations from both groups are presented together. For detailed study characteristics, please refer to online supplemental table S2).

Notes: Long-term health conditions (online supplemental table S2 provides the specific outcomes) were classified and defined following Medical Subject Headings (MeSH) criteria when available. Cardiovascular diseases, ‘pathological conditions involving the cardiovascular system including the heart; the blood vessels; or the pericardium’; Cancer (classified as neoplasms), ‘new abnormal growth of tissue. Malignant neoplasms show a greater degree of anaplasia and have the properties of invasion and metastasis, compared with benign neoplasms’; Type 2 diabetes mellitus, ‘a subclass of diabetes mellitus that is not insulin-responsive or dependent’; Respiratory diseases (classified as respiratory tract diseases), ‘diseases involving the respiratory system’; Musculoskeletal impairment (classified as musculoskeletal diseases) ‘diseases of the muscles and their associated ligaments and other connective tissue and of the bones and cartilage viewed collectively’; Disability (classified as mobility limitation), ‘difficulty in walking from place to place’; Anxiety (diagnosis or symptom severity), ‘persistent and disabling anxiety, or feelings or emotions of dread, apprehension, and impending disaster’; Depression (diagnosis or symptom severity), ‘depressive states usually of moderate intensity in contrast with major depressive disorder present in neurotic and psychotic disorders; an affective disorder manifested by either a dysphoric mood or loss of interest or pleasure in usual activities, in which the mood disturbance is prominent and relatively persistent, with major depression defined as a disorder in which five (or more) symptoms have been present during the same 2-week period and represent a change from previous functioning, with at least one symptom being depressed mood or loss of interest or pleasure’; Cognitive decline, (classified as cognitive dysfunction) ‘diminished or impaired mental and/or intellectual function’; Dementia, ‘an acquired organic mental disorder with loss of intellectual abilities of sufficient severity to interfere with social or occupational functioning. The dysfunction is multifaceted and involves memory, behaviour, personality, judgement, attention, spatial relations, language, abstract thought and other executive functions. The intellectual decline is usually progressive and initially spares the level of consciousness’; and Parkinson’s disease, ‘a progressive, degenerative neurologic disease characterised by a tremor that is maximal at rest, retropulsion (ie, a tendency to fall backwards), rigidity, stooped posture, slowness of voluntary movements and a masklike facial expression’.

ADL, activities of daily living; NA, not applicable.

Analyses using definitions of muscular weakness (ie, ≥26**–**31 kg for males and≥16**–**21 kg for females) yielded comparable results (online supplemental appendix 2, table S8). Likewise, higher relative HGS test was associated with a reduced risk of cardiovascular diseases, T2DM and Parkinson’s disease in most cohort studies (online supplemental appendix 2, table S9). The leave-one-out sensitivity analyses showed that the pooled associations remained largely unchanged after excluding individual studies (online supplemental figures S17-S33). A detailed synthesis of all subgroup and sensitivity analyses is provided in online supplemental appendix 1.

## Discussion

The results of the systematic review indicate that the HGS test is the most widely used simple and feasible field-based muscular strength test in relation to incident long-term health conditions, followed by the 5-CST. Overall, the methodological quality of the studies was high and the certainty of evidence was low or very low, except T2DM and Dementia which were rated as moderate in both muscular strength tests. Our meta-analysis supports the HGS test as a consistent marker of future long-term health conditions, with the highest health-related validity among existing field-based tests.

The predictive associations between HGS test and long-term health conditions were broadly consistent across subgroup and sensitivity analyses. Despite this, specific differences emerged by age, sex, geographical region and clinical complexity. Stronger associations between HGS and musculoskeletal impairment, disability, cognitive decline and dementia in older adults likely reflect early manifestations of subclinical or functional declines associated with ageing. Whereas stronger associations with cardiovascular disease in middle-aged adults suggest that muscular strength may serve as an early indicator of long-term physiological resilience. Additionally, more pronounced associations for disability, depression and cognitive decline among women, and for T2DM and musculoskeletal impairment among men, suggest potential sex-specific pathways linking muscular strength with long-term health conditions. Regional differences were also observed with stronger protective associations for cardiovascular disease and depression in Europe, T2DM in Asia, and disability and cognitive decline in North America. In turn, in populations with higher clinical complexity, muscular strength may be particularly relevant as an indicator of reduced risk for cardiovascular disease and cognitive decline. Whereas in populations with lower clinical complexity, stronger associations with musculoskeletal impairment and disability highlight the importance of preserving muscular function to prevent early physical limitations. These variations may reflect differences in underlying health status, healthcare systems, lifestyle factors and the burden of chronic diseases across populations.

While some associations, such as a 7% reduction in cardiovascular disease risk for every 5 kg increase in HGS or a 6% reduction in musculoskeletal impairment for every 1 s improvement in the 5-CST performance, may appear modest at an individual level, they can be clinically significant at a population level. Indeed, an increase in HGS of 5 kg exceeds the minimal clinically important difference for this measure (≈3–5 kg) and is associated with a 7–10% reduction in cardiovascular risk. This represents a clinically meaningful improvement at both the individual and population levels.^195 196^ In public health, even small reductions in risk across large populations can translate into a significant number of prevented cases and associated healthcare costs.

The considerable heterogeneity (I²>75%) observed in some pooled estimates, particularly for Parkinson’s disease (high vs low HGS), respiratory diseases, musculoskeletal impairment, disability and depression (per 5 kg increase in HGS), as well as musculoskeletal impairment (per 1 s decrease in 5-CST), warrants cautious interpretation. Potential sources of between-study heterogeneity include differences in population characteristics (eg, age distribution, sex composition, baseline health status), outcome measurement methods (self-reported symptom-based reporting vs clinically confirmed diagnoses), follow-up durations, and the covariate adjustments applied across studies. Inconsistencies in the thresholds used to define muscle strength categories, as well as variations in test protocols, may have further contributed to between-study variability.

In addition, the subgroup analyses by baseline clinical complexity (eg, apparently healthy vs clinical populations) were performed at the study level. Because these analyses are ecological in nature, they cannot account for within-study confounding factors or effect modification and should therefore be interpreted with caution. Nonetheless, they provide exploratory insights that may generate hypotheses for future individual-level research into how baseline clinical status influences the relationship between muscular strength and long-term health conditions.

To note, adults not classified as having muscular weakness were found to have a significantly lower risk of developing several long-term health conditions compared with those classified as having weakness. These findings emphasise the potential of HGS tests as a simple clinical indicator of muscular strength in forecasting multiple long-term health conditions. The HGS test is a comprehensive measure of strength, encapsulating various facets of physical capability.^197^ Moreover, the protective role of the highest HGS test values for cardiovascular diseases and T2DM is robust and clinically significant, irrespective of whether HGS test was expressed as an absolute value or normalised for body size.

Regarding the 5-CST, to our knowledge, this is the first study to systematically gather existing evidence regarding the predictive value of this test in relation to long-term health conditions, including T2DM, musculoskeletal impairment, disability, depression and dementia. Consequently, incorporating the 5-CST into clinical practice could be used as a simple and useful screening tool to potentially identify individuals at higher risk of these conditions. The 5-CST is a reliable and valid measure of lower limb strength, as well as static and dynamic balance, and functional mobility, mainly among older adults.^198^ This makes it a valuable instrument for evaluating musculoskeletal and balance impairments in older adults.^198^ Although most cohort studies exploring the predictive validity of the 5-CST included in this review focused on older adults, preliminary evidence suggests its predictive validity for musculoskeletal impairment,^199^ disability^126 136^ and depression^147^ in middle-aged populations. Therefore, it might be useful to include this test as a health prevention tool earlier in adulthood, although more studies are needed to corroborate its robustness. The lack of standardisation in the cut-off points applied for the 5-CST introduces variability across studies, which may impact the comparability and interpretation of the pooled findings.

Our research indicates that the evaluation of HGS test and 5-CST serves as a simple method for risk stratification in relation to multiple long-term health conditions. However, it is important to note that the 5-CST is more challenging than the HGS test, as evidenced by the increased percentage of individuals who are unable to complete the test in older age groups.^200^ Consequently, the HGS test is more widely adopted in clinical and research settings. Most likely, it is easier to standardise, supported by well-established protocols and particularly suitable for use in older adults and inpatient populations. Taken together, our findings underscore the clinical relevance of field-based muscular strength tests as simple, scalable tools to support early risk stratification and preventive action in both clinical and community health settings.

### Limitations and strengths

While the findings of this study provide valuable insights, several limitations warrant consideration. First, this meta-analysis primarily focused on the HGS test and the 5-CST. Future research should explore a broader range of muscular strength assessments to enhance our understanding. Second, although we investigated the HGS test and 5-CST, variations in protocols, equipment and cut-off values were not fully accounted for, and these can influence the between-study heterogeneity of the meta-analysis. Moreover, high statistical heterogeneity (I² >75%) for several long-term health conditions (eg, cognitive decline, musculoskeletal impairment, depression and respiratory diseases) was observed, which may limit the interpretability of pooled estimates despite the consistency of directional effects.

Third, long-term health conditions such as cancer, respiratory diseases or health-related quality of life require further investigation, given that the small number of studies incorporated in this systematic review yield inconclusive or contradictory results. Fourth, the subgroup analyses comprised a limited number of studies, and the exploratory data should be interpreted with caution. Fifth, due to the paucity of data in the extant evidence and its considerable heterogeneity, it was not feasible to perform some of the proposed subgroup and sensitivity analyses in the systematic review protocol. The aim of these analyses would have been to evaluate the impact of relevant factors, including individual studies and risk factors such as physical activity or smoking. Additional sensitivity analyses were not conducted to consider the role of biomarkers due to the same limitation. Furthermore, it should be noted that each major group of long-term health conditions included a range of heterogeneous diagnoses and symptom-based outcomes, which may have introduced variability into the pooled estimates. Such outcome heterogeneity could reduce the precision of the summary estimates and limit their clinical applicability across specific conditions, as combining diverse disease entities may obscure condition-specific associations. Besides, due to the limited number of comparisons (n<10) analysing the predictive value of HGS test and 5-CST on long-term health conditions, meta-regressions^48^ and publication bias^201^ could not be assessed. Finally, further studies are needed in underrepresented populations, including individuals from low-income countries, ethnic minorities and younger age groups, to enhance the global applicability and equity of muscular strength-based risk stratification tools.

This study sheds light on the predictive validity of the HGS test and the 5-CST as predictors of multiple long-term health conditions. The robustness of our meta-analysis is supported by the synthesis of 94 unique cohort studies with large samples of participants from different regions, long follow-up and a wide age range. These cohorts include a variety of subgroups, allowing us to conduct nuanced and detailed analyses. This, in turn, increases the generalisability and scope of our conclusions, making them applicable to a wider array of contexts and scenarios.

### Clinical implications

These findings highlight the clinical utility of simple, low-cost field-based muscular strength tests, such as the HGS test and the 5-CST, as effective predictors of multiple long-term health conditions. In primary care settings, the HGS test can serve as a quick and practical screening tool to assess overall health^11 197^ and identify individuals at risk of long-term health conditions, including cardiovascular diseases, T2DM, musculoskeletal impairment, disability, anxiety, depression, cognitive decline, dementia and Parkinson’s disease. While the 5-CST provides valuable insights into lower limb impairments and functional decline, particularly in older adults, and also T2DM, depression and dementia. However, given that the certainty of evidence was rated as very low for respiratory diseases and Parkinson’s disease when using the HGS test, and low for depression when using the 5-CST, the results for these outcomes should be interpreted with caution. In contrast, outcomes such as cardiovascular diseases, T2DM, musculoskeletal impairment, disability, anxiety, depression (these last two only for the HGS test), cognitive decline and dementia were supported by high or moderate certainty evidence, reinforcing the clinical relevance of field-based muscular strength assessments.

Importantly, our findings suggest that in populations with higher clinical complexity, maintaining muscular strength may be particularly critical for reducing the risk of cardiovascular disease and cognitive decline, whereas in populations with lower clinical complexity, muscular strength assessments may be especially valuable for early detection and prevention of musculoskeletal impairment and disability. These complementary tests can be seamlessly integrated into routine clinical workflows due to their simplicity, minimal equipment requirements and non-invasive nature.^202^ The HGS test can be completed in less than a minute using a portable dynamometer, while the 5-CST can usually be performed in less than 15–30 s, in a standard consultation room without additional infrastructure. We recommend performing both the HGS test and the 5-CST as part of routine healthcare provider visits for adults and older adults, as it offers an efficient method to support early detection and timely intervention, particularly for those with significant risk factors. This approach is also particularly beneficial for older adults and individuals with mobility limitations, supporting preventive and therapeutic strategies. Although no formal guidelines exist, periodic reassessment of muscular strength may be valuable, particularly in clinical or rehabilitation contexts, based on individual risk profiles and intervention goals. Intervals of approximately 3 months may be appropriate, reflecting the typical duration required to observe physiological changes resulting from effective exercise interventions.^203^ However, this estimate is extrapolated from strength adaptation timelines rather than from outcome trials, and formal studies are needed to identify the optimal testing interval.

### Future research

Future research should aim to integrate objective measures such as biomarkers alongside assessments of muscular strength to provide a more comprehensive understanding of the mechanisms underlying long-term health conditions. Biomarkers (eg, analytical, tumorous or metabolic) could offer valuable insight into the biological pathways underlying risk and progression of long-term health conditions. Incorporating biomarker data in future studies may enhance the predictive power of field-based muscular strength tests and facilitate the identification of individuals at higher risk for specific long-term health conditions. Such integration could help standardise methodologies across studies, improving comparability and the reliability of pooled evidence. Furthermore, the development of consensus guidelines for the implementation and reporting of field-based muscular strength tests is essential to enhance cross-study comparability and reproducibility. Establishing standardised protocols for commonly used tests, such as HGS test and 5-CST, alongside clear reporting criteria for measurement units, repetitions and cut-off values, will facilitate the consistency of research findings and support their clinical applicability.

Additionally, it is recommended that future studies track co-existing long-term health conditions as part of the clinical complexity of general adult populations^204^ and examine how specific patterns of health conditions (eg, number, accumulation, clusters, trajectories) may influence the associations under investigation. In our study, exploratory subgroup analyses were conducted at the study level using aggregated data, rather than individual participant data, which limits the ability to draw definitive conclusions about the influence of coexisting long-term health conditions on the associations observed. Moreover, considering known biological and social differences by sex and age, further studies are recommended to analyse the influence of these factors in more detail. Additionally, given the potential impact of population health profiles, healthcare systems, lifestyle factors and disease burden across different contexts, our findings highlight the need for future research to include underrepresented populations, such as those from specific regions (ie, Africa, Oceania, Latin America and the Caribbean), low-income countries and ethnically diverse groups, to enhance the generalisability, equity and applicability of evidence related to field-based muscular strength tests and long-term health conditions.

## Conclusions

This comprehensive systematic review and meta-analysis provides evidence supporting predictive validity of the HGS test and the 5-CST across a broad spectrum of long-term health conditions among adults. Both tests emerge as simple and accessible tools that may be useful for preventive healthcare and intervention planning in clinical practice. Notably, HGS was by far the most used field-based muscular strength test, with evidence of varying certainty supporting its clinical prognostic value across multiple long-term health conditions. Subgroup analyses suggested that predictive associations were generally consistent across diverse population groups, with some specific variations. Stronger associations were observed between muscular strength and musculoskeletal impairment, disability, cognitive decline and dementia in older adults, and cardiovascular disease in middle-aged adults. Additionally, disability, depression and cognitive decline showed stronger associations among women, whereas T2DM and musculoskeletal impairment showed a stronger association among men. Overall, our findings reinforce the widespread applicability of field-based muscular strength tests as valuable measures for health monitoring and risk stratification in clinical practice, emphasising their role in the early identification of individuals at greater risk of developing long-term health conditions. These findings ultimately highlight the clinical and public health relevance of integrating simple muscular strength assessments into routine evaluations, as they can meaningfully contribute to early prevention, personalised care and healthier ageing trajectories. However, given that the certainty of evidence varied substantially across long-term health conditions, these findings should be interpreted with caution. Further high-quality longitudinal studies are warranted, particularly for long-term health conditions with limited evidence or rated as low or very low certainty, to strengthen the evidence base and improve confidence in these associations.

## Supplementary material

10.1136/bjsports-2024-109173 online supplemental appendix 1

10.1136/bjsports-2024-109173 online supplemental appendix 2

10.1136/bjsports-2024-109173 online supplemental appendix 3

## Data Availability

Data are available on reasonable request. All data relevant to the study are included in the article or uploaded as supplementary information.
