# Supplementary material for: Clinical importance of simple muscular fitness tests to predict long-term health conditions: a systematic review and meta-analysis of 94 cohort studies
Source: Br J Sports Med. 2026 Feb 10;60(6):e109173. doi: 10.1136/bjsports-2024-109173 (PMC13018823; doi:10.1136/bjsports-2024-109173)
Supplement: online supplemental appendix 1 [file bjsports-60-6-s001.docx]

**Appendix 1. Online Supplemental Material**

**SUPPLEMENTARY METHODS**

**Complete search strategy for PubMed**

((((("old"[Title/Abstract]) AND (adult*[Title/Abstract])) OR (Adult*[Title/Abstract] OR "Middle aged"[Title/Abstract] OR aged[Title/Abstract] OR elder*[Title/Abstract])) AND ((((((((((("bone health"[Title/Abstract] OR "Bone Diseases"[Title/Abstract] OR "Bone Density"[Title/Abstract] OR "bone quality"[Title/Abstract] OR osteop*[Title/Abstract] OR "mineral density"[Title/Abstract] OR "bone content"[Title/Abstract] OR "Bone Mineral Content"[Title/Abstract] OR "bone strength"[Title/Abstract] OR "bone mass"[Title/Abstract] OR "Accidental Falls"[Title/Abstract] OR Falls[Title/Abstract]) OR (Rheumat*[Title/Abstract] OR Rheumatism[Title/Abstract] OR "rheumatic diseases"[Title/Abstract] OR "Disease Rheumatic"[Title/Abstract] OR "Chronic Fatigue Syndrome"[Title/Abstract] OR osteoarth*[Title/Abstract] OR arthritis[Title/Abstract] OR arthrosis[Title/Abstract] OR fibromyalgia[Title/Abstract])) OR (Parkinson[Title/Abstract])) OR (Dementia*[Title/Abstract] OR Amentia*[Title/Abstract] OR Alzheimer[Title/Abstract])) OR ("Mental Health" OR "Self Concept*"[Title/Abstract] OR "self-perception*"[Title/Abstract] OR "Self-esteem*"[Title/Abstract] OR "Body Image*"[Title/Abstract] OR "Body representation*"[Title/Abstract] OR "Body Schema"[Title/Abstract] OR Self efficacy*[Title/Abstract] OR Happiness*[Title/Abstract] OR "Positive affect*"[Title/Abstract] OR Mood*[Title/Abstract] OR Optimis[Title/Abstract])) OR (Anxiet*[Title/Abstract] OR Hypervigilance[Title/Abstract] OR Nervousness[Title/Abstract] OR Depression*[Title/Abstract] OR "Depressive Symptom"[Title/Abstract] OR Pessimism[Title/Abstract] OR "Psychological stress"[Title/Abstract] OR stress*[Title/Abstract] OR "Negative affect*"[Title/Abstract])) OR ("quality of life"[Title/Abstract] OR "Life Quality"[Title/Abstract] OR "QOL"[Title/Abstract] OR "HRQOL"[Title/Abstract])) OR ("Mobility Limitation*"[Title/Abstract] OR "Ambulation Difficult*"[Title/Abstract] OR "Ambulatory Difficult*"[Title/Abstract] OR "Difficulty Walking"[Title/Abstract] OR frailt*[Title/Abstract] OR "Frail Elderly"[Title/Abstract] OR "Functionally-Impaired Elderly"[Title/Abstract] OR "Frail Older Adults"[Title/Abstract] OR "ADL"[Title/Abstract] OR "Activities, Daily Living"[Title/Abstract] OR "Chronic Limitation of Activity"[Title/Abstract])) OR ("Low Back Pain"[Title/Abstract] OR Lumbago[Title/Abstract] OR "Lower Back Pain*"[Title/Abstract] OR "Low Back Ache*"[Title/Abstract] OR "Low Backache"[Title/Abstract] OR sciatica[Title/Abstract] OR "Sciatic Neuralgia"[Title/Abstract] OR "neck pain"[Title/Abstract] OR Cervicalgia[Title/Abstract] OR Neckache[Title/Abstract] OR "Cervical Pain"[Title/Abstract])) OR ("cardiovascular risk"[Title/Abstract] OR "Cardiovascular Diseases"[Title/Abstract] OR "Cardiovascular Risk Factors"[Title/Abstract] OR "Risk Factors for Heart Disease"[Title/Abstract] OR "Cardiovascular Risk Score"[Title/Abstract] OR "Artery Disease Coronary"[Title/Abstract] OR "coronary disease"[Title/Abstract] OR "Coronary Atherosclerosis"[Title/Abstract] OR "stroke"[Title/Abstract] OR "Cerebrovascular Accident"[Title/Abstract] OR "CVA"[Title/Abstract] OR "Cerebrovascular Apoplexy"[Title/Abstract] OR "Brain Vascular Accident"[Title/Abstract] OR "Cerebrovascular Stroke"[Title/Abstract] OR obesity[Title/Abstract] OR Hypertension[Title/Abstract] OR "High Blood Pressure"[Title/Abstract] OR Dyslipidemia[Title/Abstract] OR Dyslipoproteinemia[Title/Abstract] OR Hypercholesterolemia[Title/Abstract] OR "High Cholesterol Level"[Title/Abstract] OR Hyperglycemia[Title/Abstract] OR "Postprandial Hyperglycemia"[Title/Abstract] OR "Glucose Metabolism Disorders"[Title/Abstract] OR "Glucose Metabolic Disorder"[Title/Abstract] OR "Insulin Resistance"[Title/Abstract] OR "Insulin Sensitivity"[Title/Abstract] OR "Diabetes Mellitus Type 2"[Title/Abstract] OR "Diabetes Mellitus, Non Insulin Dependent"[Title/Abstract] OR "Diabetes Mellitus Type II"[Title/Abstract] OR "NIDDM"[Title/Abstract] OR "neoplas*"[Title/Abstract] OR "cancer"[Title/Abstract] OR "tumor"[Title/Abstract])) OR ("multiple sclerosis"[Title/Abstract]))) AND ("Muscular fitness"[Title/Abstract] OR "Muscle Strength"[Title/Abstract] OR "Muscular strength"[Title/Abstract] OR "Musculoskeletal fitness"[Title/Abstract] OR "muscular endurance"[Title/Abstract] OR "muscular power"[Title/Abstract] OR "Muscle Strength Dynamometer"[Title/Abstract] OR "handgrip strength"[Title/Abstract] OR "back-leg strength"[Title/Abstract] OR "isometric leg strength"[Title/Abstract] OR "Biering-Sorensen test"[Title/Abstract] OR "Sorensen test"[Title/Abstract] OR "trunk flexion"[Title/Abstract] OR "chair-rise"[Title/Abstract] OR "sit-to-stand test"[Title/Abstract] OR "chair-stand"[Title/Abstract] OR "prone bridge"[Title/Abstract] OR "prone bridging"[Title/Abstract] OR "prone plank"[Title/Abstract] OR "sit-up"[Title/Abstract] OR "partial curl-up"[Title/Abstract] OR "flexion rotation trunk"[Title/Abstract] OR "timed stair ascent"[Title/Abstract] OR "pull-up test"[Title/Abstract] OR "bent-arm hang"[Title/Abstract] OR "flexed arm hang"[Title/Abstract] OR "standing broad jump"[Title/Abstract] OR "standing long jump"[Title/Abstract] OR "trunk lift"[Title/Abstract])) AND ("Cohort Study"[Title/Abstract] OR "Cohort Studies"[Title/Abstract] OR "Longitudinal Studies"[Title/Abstract] OR "Longitudinal Study"[Title/Abstract] OR "Studies, Longitudinal"[Title/Abstract] OR "Study, Longitudinal"[Title/Abstract] OR "Longitudinal Survey"[Title/Abstract] OR "Longitudinal Surveys"[Title/Abstract] OR "Survey, Longitudinal"[Title/Abstract] OR "Surveys, Longitudinal"[Title/Abstract] OR "Prospective study"[Title/Abstract] OR "Retrospective study"[Title/Abstract] OR "Follow Up Study"[Title/Abstract] OR "Follow Up"[Title/Abstract] "Follow-Up").

**Complete search strategy for Web of Science**

TS=(Adult* OR "old adult*" OR "Middle aged" OR aged OR elder*) AND TS=("bone health" OR "Bone Diseases" OR "Bone Density" OR "bone quality" OR osteopo* OR "mineral density" OR "bone content" OR "Bone Mineral Content" OR "bone strength" OR "bone mass" OR "Accidental Falls" OR Falls OR Rheumat* OR Rheumatism OR "rheumatic diseases" OR "Disease Rheumatic" OR "Chronic Fatigue Syndrome" OR osteoarth* OR arthritis OR arthrosis OR fibromyalgia OR "multiple sclerosis" OR Parkinson OR Dementia* OR Amentia* OR Alzheimer OR "Mental Health" OR "Self Concept*" OR "self-perception*" OR "Self-esteem*" OR "Self esteem*" OR "Body Image*" OR "Body representation*" OR "Body Schema" OR "Self-efficacy*" OR "Happiness*" OR "Positive affect*" OR Mood* OR "Optimism" OR Anxiet* OR Hypervigilance OR Nervousness OR Depression* OR "Depressive Symptom" OR Pessimism OR "Psychological stress" OR stress* OR "Negative affect*" OR "quality of life" OR "Life Quality" OR "QOL" OR "HRQOL" OR "Mobility Limitation*" OR "Ambulation Difficult*" OR "Ambulatory Difficult*" OR "Difficulty Walking" OR frailt* OR "Frail Elderly" OR "Functionally-Impaired Elderly" OR "Frail Older Adults" OR "ADL" OR "Activities, Daily Living" OR "Chronic Limitation of Activity" OR "Low Back Pain" OR Lumbago OR "Lower Back Pain*" OR "Low Back Ache*" OR "Low Backache" OR sciatica OR "Sciatic Neuralgia" OR "neck pain" OR Cervicalgia OR neckache OR "Cervical Pain" OR "cardiovascular risk" OR "Cardiovascular Diseases" OR "Cardiovascular Risk Factors" OR "Risk Factors for Heart Disease" OR "Cardiovascular Risk Score" OR "Artery Disease Coronary" OR "coronary disease" OR "Coronary Atherosclerosis" OR cancer OR "Cerebrovascular Accident" OR "Cerebrovascular Apoplexy" OR "Brain Vascular Accident" OR "Cerebrovascular Stroke" OR "Metabolic Syndrome" OR "Metabolic Syndrome X" OR "Insulin Resistance Syndrome X" OR "Dysmetabolic Syndrome X" OR "Metabolic Cardiovascular Syndrome" OR "Cardiometabolic Syndrome" OR obesity OR Hypertension OR "High Blood Pressure" OR Dyslipidemia OR Dyslipoproteinemia OR Hypercholesterolemia OR "High Cholesterol Level" OR Hyperglycemia OR "Postprandial Hyperglycemia" OR "Glucose Metabolism Disorders" OR "Glucose Metabolic Disorder" OR "Insulin Resistance" OR "Insulin Sensitivity" OR "Diabetes Mellitus Type 2" OR "Diabetes Mellitus, Non Insulin Dependent" OR "Diabetes Mellitus Type II" OR "NIDDM" OR neoplas* OR "cancer" OR "tumor") AND TS=("Muscular fitness" OR "Muscle Strength" OR "Muscular strength" OR "Musculoskeletal fitness" OR "muscular endurance" OR "muscular power" OR "Muscle Strength Dynamometer" OR "handgrip strength" OR "back-leg strength" OR "isometric leg strength" OR "Biering-Sorensen test" OR "Sorensen test" OR "trunk flexion" OR "chair-rise" OR "sit-to-stand test" OR "chair-stand" OR "prone bridge" OR "prone bridging" OR "prone plank" OR "sit-up" OR "partial curl-up" OR "flexion rotation trunk" OR "timed stair ascent" OR "pull-up test" OR "bent-arm hang" OR "flexed arm hang" OR "standing broad jump" OR "standing long jump" OR "trunk lift") AND TS=("Cohort Study" OR "Cohort Studies" OR "Longitudinal Studies" OR "Longitudinal Study" OR "Studies, Longitudinal" OR "Study, Longitudinal" OR "Longitudinal Survey" OR "Longitudinal Surveys" OR "Survey, Longitudinal" OR "Surveys, Longitudinal" OR "Prospective study" OR "Retrospective study" OR "Follow Up Study" OR "Follow Up" OR "Follow-Up")

**Complete search strategy for SPORTDiscus**

(("old" AND adult*) OR "Adult*" OR "Middle aged" OR aged OR elder*)

AND

("bone health" OR "Bone Diseases" OR "Bone Density" OR "bone quality" OR osteop* OR "mineral density" OR "bone content" OR "Bone Mineral Content" OR "bone strength" OR "bone mass" OR "Accidental Falls" OR Falls OR Rheumat* OR Rheumatism OR "rheumatic diseases" OR "Disease Rheumatic" OR "Chronic Fatigue Syndrome" OR osteoarth* OR arthritis OR arthrosis OR fibromyalgia OR Parkinson OR Dementia* OR Amentia* OR Alzheimer OR "Mental Health" OR "Self Concept*" OR "self-perception*" OR "Self-esteem*" OR "Body Image*" OR "Body representation*" OR "Body Schema" OR Self efficacy* OR Happiness* OR "Positive affect*" OR Mood* OR Optimis* OR Anxiet* OR Hypervigilance OR Nervousness OR Depression* OR "Depressive Symptom" OR Pessimism OR "Psychological stress" OR stress* OR "Negative affect*" OR "quality of life" OR "Life Quality" OR "QOL" OR "HRQOL" OR "Mobility Limitation*" OR "Ambulation Difficult*" OR "Ambulatory Difficult*" OR "Difficulty Walking" OR frailt* OR "Frail Elderly" OR "Functionally-Impaired Elderly" OR "Frail Older Adults" OR "ADL" OR "Activities, Daily Living" OR "Chronic Limitation of Activity" OR "Low Back Pain" OR Lumbago OR "Lower Back Pain*" OR "Low Back Ache*" OR "Low Backache" OR sciatica OR "Sciatic Neuralgia" OR "neck pain" OR Cervicalgia OR Neckache OR "Cervical Pain" OR "cardiovascular risk" OR "Cardiovascular Diseases" OR "Cardiovascular Risk Factors" OR "Risk Factors for Heart Disease" OR "Cardiovascular Risk Score" OR "Artery Disease Coronary" OR "coronary disease" OR "Coronary Atherosclerosis" OR stroke OR "Cerebrovascular Accident" OR CVA OR "Cerebrovascular Apoplexy" OR "Brain Vascular Accident" OR "Cerebrovascular Stroke" OR obesity OR Hypertension OR "High Blood Pressure" OR Dyslipidemia OR Dyslipoproteinemia OR Hypercholesterolemia OR "High Cholesterol Level" OR Hyperglycemia OR "Postprandial Hyperglycemia" OR "Glucose Metabolism Disorders" OR "Glucose Metabolic Disorder" OR "Insulin Resistance" OR "Insulin Sensitivity" OR "Diabetes Mellitus Type 2" OR "Diabetes Mellitus, Non Insulin Dependent" OR "Diabetes Mellitus Type II" OR "NIDDM" OR neoplas* OR cancer OR tumor OR "multiple sclerosis")

AND

("Muscular fitness" OR "Muscle Strength" OR "Muscular strength" OR "Musculoskeletal fitness" OR "muscular endurance" OR "muscular power" OR "Muscle Strength Dynamometer" OR "handgrip strength" OR "back-leg strength" OR "isometric leg strength" OR "Biering-Sorensen test" OR "Sorensen test" OR "trunk flexion" OR "chair-rise" OR "sit-to-stand test" OR "chair-stand" OR "prone bridge" OR "prone bridging" OR "prone plank" OR "sit-up" OR "partial curl-up" OR "flexion rotation trunk" OR "timed stair ascent" OR "pull-up test" OR "bent-arm hang" OR "flexed arm hang" OR "standing broad jump" OR "standing long jump" OR "trunk lift")

AND

("Cohort Study" OR "Cohort Studies" OR "Longitudinal Studies" OR "Longitudinal Study" OR "Studies, Longitudinal" OR "Study, Longitudinal" OR "Longitudinal Survey" OR "Longitudinal Surveys" OR "Survey, Longitudinal" OR "Surveys, Longitudinal" OR "Prospective study" OR "Retrospective study" OR "Follow Up Study" OR "Follow Up" OR "Follow-Up")

**Complete search strategy for SCOPUS**

(("old" AND adult*) OR "Adult*" OR "Middle aged" OR aged OR elder*)

AND

("bone health" OR "Bone Diseases" OR "Bone Density" OR "bone quality" OR osteop* OR "mineral density" OR "bone content" OR "Bone Mineral Content" OR "bone strength" OR "bone mass" OR "Accidental Falls" OR Falls OR Rheumat* OR Rheumatism OR "rheumatic diseases" OR "Disease Rheumatic" OR "Chronic Fatigue Syndrome" OR osteoarth* OR arthritis OR arthrosis OR fibromyalgia OR Parkinson OR Dementia* OR Amentia* OR Alzheimer OR "Mental Health" OR "Self Concept*" OR "self-perception*" OR "Self-esteem*" OR "Body Image*" OR "Body representation*" OR "Body Schema" OR Self efficacy* OR Happiness* OR "Positive affect*" OR Mood* OR Optimis* OR Anxiet* OR Hypervigilance OR Nervousness OR Depression* OR "Depressive Symptom" OR Pessimism OR "Psychological stress" OR stress* OR "Negative affect*" OR "quality of life" OR "Life Quality" OR "QOL" OR "HRQOL" OR "Mobility Limitation*" OR "Ambulation Difficult*" OR "Ambulatory Difficult*" OR "Difficulty Walking" OR frailt* OR "Frail Elderly" OR "Functionally-Impaired Elderly" OR "Frail Older Adults" OR "ADL" OR "Activities, Daily Living" OR "Chronic Limitation of Activity" OR "Low Back Pain" OR Lumbago OR "Lower Back Pain*" OR "Low Back Ache*" OR "Low Backache" OR sciatica OR "Sciatic Neuralgia" OR "neck pain" OR Cervicalgia OR Neckache OR "Cervical Pain" OR "cardiovascular risk" OR "Cardiovascular Diseases" OR "Cardiovascular Risk Factors" OR "Risk Factors for Heart Disease" OR "Cardiovascular Risk Score" OR "Artery Disease Coronary" OR "coronary disease" OR "Coronary Atherosclerosis" OR stroke OR "Cerebrovascular Accident" OR CVA OR "Cerebrovascular Apoplexy" OR "Brain Vascular Accident" OR "Cerebrovascular Stroke" OR obesity OR Hypertension OR "High Blood Pressure" OR Dyslipidemia OR Dyslipoproteinemia OR Hypercholesterolemia OR "High Cholesterol Level" OR Hyperglycemia OR "Postprandial Hyperglycemia" OR "Glucose Metabolism Disorders" OR "Glucose Metabolic Disorder" OR "Insulin Resistance" OR "Insulin Sensitivity" OR "Diabetes Mellitus Type 2" OR "Diabetes Mellitus, Non Insulin Dependent" OR "Diabetes Mellitus Type II" OR "NIDDM" OR neoplas* OR cancer OR tumor OR "multiple sclerosis")

AND

("Muscular fitness" OR "Muscle Strength" OR "Muscular strength" OR "Musculoskeletal fitness" OR "muscular endurance" OR "muscular power" OR "Muscle Strength Dynamometer" OR "handgrip strength" OR "back-leg strength" OR "isometric leg strength" OR "Biering-Sorensen test" OR "Sorensen test" OR "trunk flexion" OR "chair-rise" OR "sit-to-stand test" OR "chair-stand" OR "prone bridge" OR "prone bridging" OR "prone plank" OR "sit-up" OR "partial curl-up" OR "flexion rotation trunk" OR "timed stair ascent" OR "pull-up test" OR "bent-arm hang" OR "flexed arm hang" OR "standing broad jump" OR "standing long jump" OR "trunk lift")

AND

("Cohort Study" OR "Cohort Studies" OR "Longitudinal Studies" OR "Longitudinal Study" OR "Studies, Longitudinal" OR "Study, Longitudinal" OR "Longitudinal Survey" OR "Longitudinal Surveys" OR "Survey, Longitudinal" OR "Surveys, Longitudinal" OR "Prospective study" OR "Retrospective study" OR "Follow Up Study" OR "Follow Up" OR "Follow-Up")

**Complete search strategy for CINAHL**

(("old" AND adult*) OR "Adult*" OR "Middle aged" OR aged OR elder*)

AND

("bone health" OR "Bone Diseases" OR "Bone Density" OR "bone quality" OR osteop* OR "mineral density" OR "bone content" OR "Bone Mineral Content" OR "bone strength" OR "bone mass" OR "Accidental Falls" OR Falls OR Rheumat* OR Rheumatism OR "rheumatic diseases" OR "Disease Rheumatic" OR "Chronic Fatigue Syndrome" OR osteoarth* OR arthritis OR arthrosis OR fibromyalgia OR Parkinson OR Dementia* OR Amentia* OR Alzheimer OR "Mental Health" OR "Self Concept*" OR "self-perception*" OR "Self-esteem*" OR "Body Image*" OR "Body representation*" OR "Body Schema" OR Self efficacy* OR Happiness* OR "Positive affect*" OR Mood* OR Optimis* OR Anxiet* OR Hypervigilance OR Nervousness OR Depression* OR "Depressive Symptom" OR Pessimism OR "Psychological stress" OR stress* OR "Negative affect*" OR "quality of life" OR "Life Quality" OR "QOL" OR "HRQOL" OR "Mobility Limitation*" OR "Ambulation Difficult*" OR "Ambulatory Difficult*" OR "Difficulty Walking" OR frailt* OR "Frail Elderly" OR "Functionally-Impaired Elderly" OR "Frail Older Adults" OR "ADL" OR "Activities, Daily Living" OR "Chronic Limitation of Activity" OR "Low Back Pain" OR Lumbago OR "Lower Back Pain*" OR "Low Back Ache*" OR "Low Backache" OR sciatica OR "Sciatic Neuralgia" OR "neck pain" OR Cervicalgia OR Neckache OR "Cervical Pain" OR "cardiovascular risk" OR "Cardiovascular Diseases" OR "Cardiovascular Risk Factors" OR "Risk Factors for Heart Disease" OR "Cardiovascular Risk Score" OR "Artery Disease Coronary" OR "coronary disease" OR "Coronary Atherosclerosis" OR stroke OR "Cerebrovascular Accident" OR CVA OR "Cerebrovascular Apoplexy" OR "Brain Vascular Accident" OR "Cerebrovascular Stroke" OR obesity OR Hypertension OR "High Blood Pressure" OR Dyslipidemia OR Dyslipoproteinemia OR Hypercholesterolemia OR "High Cholesterol Level" OR Hyperglycemia OR "Postprandial Hyperglycemia" OR "Glucose Metabolism Disorders" OR "Glucose Metabolic Disorder" OR "Insulin Resistance" OR "Insulin Sensitivity" OR "Diabetes Mellitus Type 2" OR "Diabetes Mellitus, Non Insulin Dependent" OR "Diabetes Mellitus Type II" OR "NIDDM" OR neoplas* OR cancer OR tumor OR "multiple sclerosis")

AND

("Muscular fitness" OR "Muscle Strength" OR "Muscular strength" OR "Musculoskeletal fitness" OR "muscular endurance" OR "muscular power" OR "Muscle Strength Dynamometer" OR "handgrip strength" OR "back-leg strength" OR "isometric leg strength" OR "Biering-Sorensen test" OR "Sorensen test" OR "trunk flexion" OR "chair-rise" OR "sit-to-stand test" OR "chair-stand" OR "prone bridge" OR "prone bridging" OR "prone plank" OR "sit-up" OR "partial curl-up" OR "flexion rotation trunk" OR "timed stair ascent" OR "pull-up test" OR "bent-arm hang" OR "flexed arm hang" OR "standing broad jump" OR "standing long jump" OR "trunk lift")

AND

("Cohort Study" OR "Cohort Studies" OR "Longitudinal Studies" OR "Longitudinal Study" OR "Studies, Longitudinal" OR "Study, Longitudinal" OR "Longitudinal Survey" OR "Longitudinal Surveys" OR "Survey, Longitudinal" OR "Surveys, Longitudinal" OR "Prospective study" OR "Retrospective study" OR "Follow Up Study" OR "Follow Up" OR "Follow-Up")

**Complete search strategy for EPISTEMONIKOS**

(("old" AND adult*) OR "Adult*" OR "Middle aged" OR aged OR elder*)

AND

("bone health" OR "Bone Diseases" OR "Bone Density" OR "bone quality" OR osteop* OR "mineral density" OR "bone content" OR "Bone Mineral Content" OR "bone strength" OR "bone mass" OR "Accidental Falls" OR Falls OR Rheumat* OR Rheumatism OR "rheumatic diseases" OR "Disease Rheumatic" OR "Chronic Fatigue Syndrome" OR osteoarth* OR arthritis OR arthrosis OR fibromyalgia OR Parkinson OR Dementia* OR Amentia* OR Alzheimer OR "Mental Health" OR "Self Concept*" OR "self-perception*" OR "Self-esteem*" OR "Body Image*" OR "Body representation*" OR "Body Schema" OR Self efficacy* OR Happiness* OR "Positive affect*" OR Mood* OR Optimis* OR Anxiet* OR Hypervigilance OR Nervousness OR Depression* OR "Depressive Symptom" OR Pessimism OR "Psychological stress" OR stress* OR "Negative affect*" OR "quality of life" OR "Life Quality" OR "QOL" OR "HRQOL" OR "Mobility Limitation*" OR "Ambulation Difficult*" OR "Ambulatory Difficult*" OR "Difficulty Walking" OR frailt* OR "Frail Elderly" OR "Functionally-Impaired Elderly" OR "Frail Older Adults" OR "ADL" OR "Activities, Daily Living" OR "Chronic Limitation of Activity" OR "Low Back Pain" OR Lumbago OR "Lower Back Pain*" OR "Low Back Ache*" OR "Low Backache" OR sciatica OR "Sciatic Neuralgia" OR "neck pain" OR Cervicalgia OR Neckache OR "Cervical Pain" OR "cardiovascular risk" OR "Cardiovascular Diseases" OR "Cardiovascular Risk Factors" OR "Risk Factors for Heart Disease" OR "Cardiovascular Risk Score" OR "Artery Disease Coronary" OR "coronary disease" OR "Coronary Atherosclerosis" OR stroke OR "Cerebrovascular Accident" OR CVA OR "Cerebrovascular Apoplexy" OR "Brain Vascular Accident" OR "Cerebrovascular Stroke" OR obesity OR Hypertension OR "High Blood Pressure" OR Dyslipidemia OR Dyslipoproteinemia OR Hypercholesterolemia OR "High Cholesterol Level" OR Hyperglycemia OR "Postprandial Hyperglycemia" OR "Glucose Metabolism Disorders" OR "Glucose Metabolic Disorder" OR "Insulin Resistance" OR "Insulin Sensitivity" OR "Diabetes Mellitus Type 2" OR "Diabetes Mellitus, Non Insulin Dependent" OR "Diabetes Mellitus Type II" OR "NIDDM" OR neoplas* OR cancer OR tumor OR "multiple sclerosis")

AND

("Muscular fitness" OR "Muscle Strength" OR "Muscular strength" OR "Musculoskeletal fitness" OR "muscular endurance" OR "muscular power" OR "Muscle Strength Dynamometer" OR "handgrip strength" OR "back-leg strength" OR "isometric leg strength" OR "Biering-Sorensen test" OR "Sorensen test" OR "trunk flexion" OR "chair-rise" OR "sit-to-stand test" OR "chair-stand" OR "prone bridge" OR "prone bridging" OR "prone plank" OR "sit-up" OR "partial curl-up" OR "flexion rotation trunk" OR "timed stair ascent" OR "pull-up test" OR "bent-arm hang" OR "flexed arm hang" OR "standing broad jump" OR "standing long jump" OR "trunk lift")

AND

("Cohort Study" OR "Cohort Studies" OR "Longitudinal Studies" OR "Longitudinal Study" OR "Studies, Longitudinal" OR "Study, Longitudinal" OR "Longitudinal Survey" OR "Longitudinal Surveys" OR "Survey, Longitudinal" OR "Surveys, Longitudinal" OR "Prospective study" OR "Retrospective study" OR "Follow Up Study" OR "Follow Up" OR "Follow-Up")

**Complete search strategy for Google Scholar**

Adult AND (bone *|* osteoporosis *|* "mineral density" *| "*bone content” *|* fall) AND ("Muscle Strength"*|* “Muscular strength”) AND (cohort *|* follow-up *|* longitudinal)

Adult AND (rheumatic | Rheumatism | "Chronic Fatigue Syndrome" | osteoarthritis| arthritis | arthrosis | fibromyalgia) AND ("Muscle Strength"| “Muscular strength”) AND (cohort | follow-up | longitudinal)

Adult AND “multiple sclerosis” AND ("Muscle Strength" *|* “Muscular strength”) AND (cohort *|*follow-up *|* longitudinal)

Adult AND Parkinson AND ("Muscle Strength" *|* “Muscular strength”) AND (cohort *|*follow-up *|* longitudinal)

Adult AND (Dementia *|* Amentia *|* Alzheimer) AND ("Muscle Strength"*|* “Muscular strength”) AND (cohort *|* follow-up *|* longitudinal)

Adult AND ("Mental Health" | "Self-concept” | “self-perception” | “Self-esteem” | “Self-efficacy” | Happiness | “Positive affect” | Mood | Optimism) AND ("Muscle Strength"| “Muscular strength”) AND (cohort | follow-up | longitudinal)

Adult AND (Anxiety | Hypervigilance | Nervousness | Depression | "Depressive Symptom" | Pessimism | "Psychological stress" | stress | "Negative affect") AND ("Muscle Strength"| “Muscular strength”) AND (cohort | follow-up | longitudinal)

Adult AND ("quality of life”) AND ("Muscle Strength"| “Muscular strength”) AND (cohort | follow-up | longitudinal)

Adult AND ("mobility limitation” | difficulty walking” | frailty | frail | “functionally-impaired elderly” | ADL | “Activities Daily Living”) AND ("Muscle Strength"| “Muscular strength”) AND (cohort | follow-up | longitudinal)

Adult AND ("Low Back Pain" | Lumbago | “Low Back Ache” | “Low Backache” | sciatica | “neck pain” | Cervicalgia | Neckache | “Cervical Pain”) AND ("Muscle Strength"| “Muscular strength”) AND (cohort | follow-up | longitudinal)

Adult AND ("cardiovascular risk" | "cardiovascular diseases" | “cardiovascular risk factors” | “coronary disease” | “coronary atherosclerosis”) AND ("Muscle Strength"| “Muscular strength”)

Adult AND (“stroke” | “cerebrovascular accident” | “brain vascular accident” | “cerebrovascular stroke”) AND ("Muscle Strength"| “Muscular strength”) AND (cohort | follow-up | longitudinal)

Adult AND (“Glucose Metabolism Disorders” | “Glucose Metabolic Disorder” | “Insulin Resistance” | “Insulin Sensitivity” | “Diabetes Mellitus Type 2” | “Diabetes Mellitus Type II”) AND (cohort | follow-up | longitudinal)

Adult AND (neoplasia | cancer | tumor) AND ("Muscle Strength"| “Muscular strength”) AND (cohort | follow-up | longitudinal)

**Long-term health conditions**

Specific long-term health conditions were classified and defined according to Medical Subject Headings (MeSH). **Cardiovascular diseases, “**pathological conditions involving the cardiovascular system including the heart; the blood vessels; or the pericardium”. **Cancer (*classified as Neoplasms*),** “new abnormal growth of tissue. Malignant neoplasms show a greater degree of anaplasia and have the properties of invasion and metastasis, compared to benign neoplasms”; **Diabetes Mellitus,** “a heterogeneous group of disorders characterized by hyperglycemia and glucose intolerance”, and **Type 2 Diabetes Mellitus**, “a subclass of Diabetes Mellitus that is not insulin-responsive or dependent”; **Respiratory diseases (*classified as Respiratory Tract Diseases*)**, “diseases involving the respiratory system”; **Musculoskeletal impairment *(classified as Musculoskeletal Diseases*)** “diseases of the muscles and their associated ligaments and other connective tissue and of the bones and cartilage viewed collectively”; **Disability (*classified as Mobility Limitation*)**, “difficulty in walking from place to place”; **Anxiety (diagnosed cases or subclinical symptoms)**, “persistent and disabling anxiety, or feelings or emotions of dread, apprehension, and impending disaster”; **Depression (diagnosed cases or subclinical symptoms),** “depressive states usually of moderate intensity, in contrast with major depressive disorder present in neurotic and psychotic disorders; an affective disorder manifested by either a dysphoric mood or loss of interest or pleasure in usual activities, in which the mood disturbance is prominent and relatively persistent, with major depression defined as a disorder in which five (or more) symptoms have been present during the same 2-week period and represent a change from previous functioning, with at least one symptom being depressed mood or loss of interest or pleasure”; **Cognitive decline, (*classified as*** ***Cognitive Dysfunction*)** “diminished or impaired mental and/or intellectual function”; **Dementia**, “an acquired organic mental disorder with loss of intellectual abilities of sufficient severity to interfere with social or occupational functioning. The dysfunction is multifaceted and involves memory, behaviour, personality, judgment, attention, spatial relations, language, abstract thought, and other executive functions. The intellectual decline is usually progressive and initially spares the level of consciousness”; and **Parkinson's disease**, “a progressive, degenerative neurologic disease characterized by a tremor that is maximal at rest, retropulsion (i.e. a tendency to fall backwards), rigidity, stooped posture, slowness of voluntary movements, and a masklike facial expression”.

**SUPPLEMENTARY RESULTS**

**List of Excluded Studies (n=292)**

- **Wrong outcomes (n=118)**

1. Abizanda, P., Romero, L., Sánchez-Jurado, P. M., Atienzar-Núñez, P., Esquinas-Requena, J. L., & García-Nogueras, I. (2012). Association between functional assessment instruments and frailty in older adults: the FRADEA study. *J frailty aging*, *1*(4), 162-168.
2. Ahmad, R., & Bath, P. A. (2005). Identification of risk factors for 15-year mortality among community-dwelling older people using Cox regression and a genetic algorithm. *The Journals of Gerontology Series A: Biological Sciences and Medical Sciences*, *60*(8), 1052-1058.
3. Al Snih, S., Markides, K. S., Ray, L., Ostir, G. V., & Goodwin, J. S. (2002). Handgrip strength and mortality in older Mexican Americans. *Journal of the American Geriatrics Society*, *50*(7), 1250-1256.
4. Andersen, K., Rasmussen, F., Held, C., Neovius, M., Tynelius, P., & Sundström, J. (2015). Exercise capacity and muscle strength and risk of vascular disease and arrhythmia in 1.1 million young Swedish men: cohort study. *Bmj*, *351*.
5. Antony, B., Jones, G., Stannus, O., Blizzard, L., & Ding, C. (2013). Body fat predicts an increase and limb muscle strength predicts a decrease in leptin in older adults over 2· 6 years. *Clinical Endocrinology*, *79*(5), 652-660.
6. Arvandi, M., Strasser, B., Meisinger, C., Volaklis, K., Gothe, R. M., Siebert, U., ... & Thorand, B. (2016). Gender differences in the association between grip strength and mortality in older adults: results from the KORA-age study. *BMC geriatrics*, *16*, 1-8.
7. Atlantis, E., Martin, S. A., Haren, M. T., Taylor, A. W., & Wittert, G. A. (2009). Inverse associations between muscle mass, strength, and the metabolic syndrome. *Metabolism*, *58*(7), 1013-1022.
8. Bae, K. H., Jo, Y. H., Lee, D. R., & Lee, J. (2020). Trajectories of handgrip strength and their associations with mortality among older adults in Korea: analysis of the Korean Longitudinal Study of Aging. *Korean Journal of Family Medicine*, *42*(1), 38.
9. Bites, A. C., Bunout, D., Barrera, G., Hirsch, S., Leiva, L., & de la Maza, M. P. (2013). Association between functional measures and mortality in older persons. *International journal of gerontology*, *7*(1), 17-21.
10. Brill, P. A., Macera, C. A., Davis, D. R., Blair, S. N., & Gordon, N. E. I. L. (2000). Muscular strength and physical function. *Medicine & Science in Sports & Exercise*, *32*(2), 412.
11. Buchman, A. S., Boyle, P. A., Wilson, R. S., Gu, L., Bienias, J. L., & Bennett, D. A. (2008). Pulmonary function, muscle strength and mortality in old age. *Mechanisms of ageing and development*, *129*(11), 625-631.
12. Buchman, A. S., Wilson, R. S., Boyle, P. A., Bienias, J. L., & Bennett, D. A. (2007). Change in motor function and risk of mortality in older persons. *Journal of the American Geriatrics Society*, *55*(1), 11-19.
13. Byeon, J. Y., Lee, M. K., Yu, M. S., Kang, M. J., Lee, D. H., Kim, K. C., ... & Jeon, J. Y. (2019). Lower relative handgrip strength is significantly associated with a higher prevalence of the metabolic syndrome in adults. *Metabolic syndrome and related disorders*, *17*(5), 280-288.
14. Cesari, M., Onder, G., Zamboni, V., Manini, T., Shorr, R. I., Russo, A., ... & Landi, F. (2008). Physical function and self-rated health status as predictors of mortality: results from longitudinal analysis in the ilSIRENTE study. *BMC geriatrics*, *8*, 1-9.
15. Chen, P. J., Lin, M. H., Peng, L. N., Liu, C. L., Chang, C. W., Lin, Y. T., & Chen, L. K. (2012). Predicting cause-specific mortality of older men living in the Veterans home by handgrip strength and walking speed: a 3-year, prospective cohort study in Taiwan. *Journal of the American Medical Directors Association*, *13*(6), 517-521.
16. Cho, J., Yoon, E., & Park, S. H. (2019). Association of relative handgrip strength with the incidence of metabolic syndrome in korean adults: a community based cohort study. *Exercise Science*, *28*(3), 303-310.
17. De Brito, L. B. B., Ricardo, D. R., de Araújo, D. S. M. S., Ramos, P. S., Myers, J., & de Araújo, C. G. S. (2014). Ability to sit and rise from the floor as a predictor of all-cause mortality. *European journal of preventive cardiology*, *21*(7), 892-898.
18. De Brito, L. B. B., Ricardo, D. R., de Araújo, D. S. M. S., Ramos, P. S., Myers, J., & de Araújo, C. G. S. (2014). Ability to sit and rise from the floor as a predictor of all-cause mortality. *European journal of preventive cardiology*, *21*(7), 892-898.
19. Edwards, M. K., & Loprinzi, P. D. (2018). Adequate muscular strength may help to reduce risk of residual-specific mortality: findings from the National Health and Nutrition Examination Survey. *Journal of Physical Activity and Health*, *15*(5), 369-373.
20. Fraser, B. J., Blizzard, L., Buscot, M. J., Schmidt, M. D., Dwyer, T., Venn, A. J., & Magnussen, C. G. (2022). Muscular strength measured across the life-course and the metabolic syndrome. *Nutrition, Metabolism and Cardiovascular Diseases*, *32*(5), 1131-1137.
21. Fujita, Y., Nakamura, Y., Hiraoka, J., Kobayashi, K., Sakata, K., Nagai, M., & Yanagawa, H. (1995). Physical-strength tests and mortality among visitors to health-promotion centers in Japan. *Journal of clinical epidemiology*, *48*(11), 1349-1359.
22. Gale, C. R., Martyn, C. N., Cooper, C., & Sayer, A. A. (2007). Grip strength, body composition, and mortality. *International journal of epidemiology*, *36*(1), 228-235.
23. Gao, J., Qiu, Y., Hou, Y., Zhang, L., Wang, K., Chen, Z., ... & Lin, J. (2022). Influencing factors for the decline of limb muscle strength and the association with all-cause mortality: evidence from a nationwide population-based cohort study. *Aging Clinical and Experimental Research*, 1-9.
24. Gu, Y., Dong, J., Meng, G., Zhang, Q., Liu, L., Wu, H., ... & Niu, K. (2021). Handgrip strength as a predictor of incident hypertension in the middle-aged and older population: the TCLSIH cohort study. *Maturitas*, *150*, 7-13.
25. Guadalupe-Grau, A., Carnicero, J. A., Gómez-Cabello, A., Gutiérrez Avila, G., Humanes, S., Alegre, L. M., ... & García-García, F. J. (2015). Association of regional muscle strength with mortality and hospitalisation in older people. *Age and ageing*, *44*(5), 790-795.
26. He, P., Gan, X., Ye, Z., Liu, M., Zhou, C., Wu, Q., ... & Qin, X. (2023). Combined handgrip strength and walking pace, genetic susceptibility, and incident hypertension: A prospective study in UK Biobank. *Scandinavian Journal of Medicine & Science in Sports*, *33*(6), 989-999.
27. Hong, S. (2019). Association of relative handgrip strength and metabolic syndrome in Korean older adults: Korea National Health and Nutrition Examination Survey VII-1. *Journal of obesity & metabolic syndrome*, *28*(1), 53.
28. Ikezoe, T., Tsuboyama, T., Tabara, Y., Matsuda, F., Ichihashi, N., & Nagahama Study group. (2021). Weak hip flexor strength predicts progression of functional capacity decline due to locomotor system dysfunction in community-dwelling older adults: a longitudinal cohort study. *Archives of Gerontology and Geriatrics*, *97*, 104499.
29. Imaeva, A. E., Kapustina, A. V., Shalnova, S. A., Balanova, Y. A., & Shkolnikov, V. M. (2019). Role of cognitive impairments and decreased muscle strength in cardiovascular mortality of 55 years and older population. *Russian Journal of Cardiology*, (6), 61-65.
30. Imagama, S., Ando, K., Kobayashi, K., Nakashima, H., Seki, T., Hamada, T., ... & Hasegawa, Y. (2020). Risk factors for neuropathic pain in middle-aged and elderly people: a five-year longitudinal cohort in the Yakumo study. *Pain Medicine*, *21*(8), 1604-1610.
31. Jackson, A. W., Lee, D. C., Sui, X., Morrow Jr, J. R., Church, T. S., Maslow, A. L., & Blair, S. N. (2010). Muscular strength is inversely related to prevalence and incidence of obesity in adult men. *Obesity*, *18*(10), 1988-1995.
32. Jeon, Y. J., Lee, S. K., & Shin, C. (2021). Relative hand grip and back muscle strength, but not mean muscle strength, as risk factors for incident metabolic syndrome and its metabolic components: 16 years of follow-up in a population-based cohort study. *Applied Sciences*, *11*(11), 5198.
33. Ji, C., Xia, Y., Tong, S., Wu, Q., & Zhao, Y. (2020). Association of handgrip strength with the prevalence of metabolic syndrome in US adults: the national health and nutrition examination survey. *Aging (Albany NY)*, *12*(9), 7818.
34. Jurca, R., Lamonte, M. J., Church, T. S., Earnest, C. P., Fitzgerald, S. J., Barlow, C. E., ... & Blair, S. N. (2004). Associations of muscle strength and fitness with metabolic syndrome in men. *Medicine & Science in Sports & Exercise*, *36*(8), 1301-1307.
35. Karlsen, T., Nauman, J., Dalen, H., Langhammer, A., & Wisløff, U. (2017, May). The combined association of skeletal muscle strength and physical activity on mortality in older women: the HUNT2 study. In *Mayo Clinic Proceedings* (Vol. 92, No. 5, pp. 710-718). Elsevier.
36. Kawamoto, R., Kikuchi, A., Akase, T., Ninomiya, D., & Kumagi, T. (2021). Handgrip Strength is Associated with Hypertension among Middle-Aged and Older Community-Dwelling Persons. *International Journal of Gerontology*, *15*(3).
37. Kawamoto, R., Kikuchi, A., Akase, T., Ninomiya, D., & Kumagi, T. (2021). Thigh circumference and handgrip strength are significantly associated with all-cause mortality: findings from a study on Japanese community-dwelling persons. *European geriatric medicine*, *12*, 1191-1200.
38. Kawamoto, R., Ninomiya, D., Kasai, Y., Kusunoki, T., Ohtsuka, N., Kumagi, T., & Abe, M. (2016). Handgrip strength is associated with metabolic syndrome among middle-aged and elderly community-dwelling persons. *Clinical and Experimental Hypertension*, *38*(2), 245-251.
39. Kim, G. R., Sun, J., Han, M., Park, S., & Nam, C. M. (2019). Impact of handgrip strength on cardiovascular, cancer and all-cause mortality in the Korean longitudinal study of ageing. *BMJ open*, *9*(5), e027019.
40. Kim, J. (2021). Handgrip strength to predict the risk of all-cause and premature mortality in Korean adults: a 10-year cohort study. *International journal of environmental research and public health*, *19*(1), 39.
41. Kim, J. H., & Kim, J. M. (2019). Association of cognitive impairment and grip strength trajectories with mortality among middle-aged and elderly adults. *International psychogeriatrics*, *31*(5), 723-734.
42. Kim, J. H., Lim, S., Choi, S. H., Kim, K. M., Yoon, J. W., Kim, K. W., ... & Kritchevsky, S. (2014). Sarcopenia: an independent predictor of mortality in community-dwelling older Korean men. *Journals of Gerontology Series A: Biomedical Sciences and Medical Sciences*, *69*(10), 1244-1252.
43. Kim, K., & Ho, J. H. (2020). Handgrip strength and mortality in elderly Koreans: Evidence from the Korea longitudinal study of ageing. *Asia Pacific Journal of Public Health*, *32*(6-7), 302-309.
44. Kim, Y., White, T., Wijndaele, K., Westgate, K., Sharp, S. J., Helge, J. W., ... & Brage, S. (2018). The combination of cardiorespiratory fitness and muscle strength, and mortality risk. *European Journal of Epidemiology*, *33*, 953-964.
45. Kim, Y., Wijndaele, K., Lee, D. C., Sharp, S. J., Wareham, N., & Brage, S. (2017). Independent and joint associations of grip strength and adiposity with all-cause and cardiovascular disease mortality in 403,199 adults: the UK Biobank study. *The American journal of clinical nutrition*, *106*(3), 773-782.
46. Kishimoto, H., Hata, J., Ninomiya, T., Nemeth, H., Hirakawa, Y., Yoshida, D., ... & Kiyohara, Y. (2014). Midlife and late-life handgrip strength and risk of cause-specific death in a general Japanese population: the Hisayama Study. *J Epidemiol Community Health*, *68*(7), 663-668.
47. Ko, K. J., Kang, S. J., & Lee, K. S. (2019). Association between cardiorespiratory, muscular fitness and metabolic syndrome in Korean men. *Diabetes & Metabolic Syndrome: Clinical Research & Reviews*, *13*(1), 536-541.
48. Kobayashi, K., Ando, K., Tsushima, M., Machino, M., Ota, K., Morozumi, M., ... & Imagama, S. (2019). Predictors of locomotive syndrome in community-living people: a prospective five-year longitudinal study. *Modern Rheumatology*, *29*(4), 669-675.
49. Kobayashi, K., Ando, K., Tsushima, M., Machino, M., Ota, K., Morozumi, M., ... & Imagama, S. (2019). Predictors of presarcopenia in community-dwelling older adults: A 5-year longitudinal study. *Modern rheumatology*, *29*(6), 1053-1058.
50. Kobayashi, K., Imagama, S., Ando, K., Tsushima, M., Machino, M., Ota, K., ... & Hasegawa, Y. (2020). Weakness of grip strength reflects future locomotive syndrome and progression of locomotive risk stage: a 10-year longitudinal cohort study. *Modern Rheumatology*, *30*(3), 573-579.
51. Kolbaşı, E. N., & Demirdağ, F. (2020). Prevalence of osteosarcopenic obesity in community-dwelling older adults: a cross-sectional retrospective study. *Archives of osteoporosis*, *15*, 1-9.
52. Kozicka, I., & Kostka, T. (2016). Handgrip strength, quadriceps muscle power, and optimal shortening velocity roles in maintaining functional abilities in older adults living in a long-term care home: a 1-year follow-up study. *Clinical interventions in aging*, 739-747.
53. Kunutsor, S. K., Mäkikallio, T. H., Voutilainen, A., Hupin, D., & Laukkanen, J. A. (2021). Normalized handgrip strength and future risk of hypertension: findings from a prospective cohort study. *Scandinavian Cardiovascular Journal*, *55*(6), 336-339.
54. Laddu, D. R., Kim, H., Cawthon, P. M., LaMonte, M. J., Phillips, S. A., Ma, J., & Stefanick, M. L. (2024). Physical performance changes as clues to late-life blood pressure changes with advanced age: the osteoporotic fractures in men study. *The Journal of nutrition, health and aging*, *28*(9), 100317.
55. Laukkanen, P. I. A., Heikkinen, E., & Kauppinen, M. (1995). Muscle strength and mobility as predictors of survival in 75–84-year-old people. *Age and ageing*, *24*(6), 468-473.
56. Lee, J., Hong, Y. P., Shin, H. J., & Lee, W. (2015). Associations of sarcopenia and sarcopenic obesity with metabolic syndrome considering both muscle mass and muscle strength. *Journal of Preventive Medicine and Public Health*, *49*(1), 35.
57. Lee, W. J., Peng, L. N., Chiou, S. T., & Chen, L. K. (2017). Physical health indicators improve prediction of cardiovascular and all-cause mortality among middle-aged and older people: a national population-based study. *Scientific reports*, *7*(1), 40427.
58. Leknessund, O. G., Morelli, V. M., Strand, B. H., Hansen, J. B., & Brækkan, S. K. (2022). Hand grip strength and risk of incident venous thromboembolism: The Tromsø study. *Research and Practice in Thrombosis and Haemostasis*, *6*(7), e12833.
59. Li, R., Xia, J., Zhang, X. I., Gathirua-Mwangi, W. G., Guo, J., Li, Y., ... & Song, Y. (2018). Associations of muscle mass and strength with all-cause mortality among US older adults. *Medicine and science in sports and exercise*, *50*(3), 458.
60. López-Bueno, R., Andersen, L. L., Calatayud, J., Casaña, J., Smith, L., Jacob, L., ... & del Pozo Cruz, B. (2022). Longitudinal association of handgrip strength with all-cause and cardiovascular mortality in older adults using a causal framework. *Experimental Gerontology*, *168*, 111951.
61. Lopez-Lopez, J. P., Cohen, D. D., Ney-Salazar, D., Martinez, D., Otero, J., Gomez-Arbelaez, D., ... & Lopez-Jaramillo, P. (2021). The prediction of metabolic syndrome alterations is improved by combining waist circumference and handgrip strength measurements compared to either alone. *Cardiovascular diabetology*, *20*, 1-11.
62. Lopez-Lopez, J. P., Ney-Salazar, D., Parra-Gomez, L. A., Camacho, P. A., Rangarajan, S., Yusuf, S., & Lopez-Jaramillo, P. (2021). Ethnic differences in the association between handgrip strength and the prevalence of hypertension. *Journal of Hypertension*, *39*, e238.
63. López-Martínez, S., Sánchez-López, M., Solera-Martinez, M., Arias-Palencia, N., Fuentes-Chacón, R. M., & Martínez-Vizcaíno, V. (2013). Physical activity, fitness, and metabolic syndrome in young adults. *International journal of sport nutrition and exercise metabolism*, *23*(4), 312-321.
64. Losa-Reyna, J., Alcazar, J., Carnicero, J., Alfaro-Acha, A., Castillo-Gallego, C., Rosado-Artalejo, C., ... & García-García, F. J. (2022). Impact of relative muscle power on hospitalization and all-cause mortality in older adults. *The Journals of Gerontology: Series A*, *77*(4), 781-789.
65. Lovesley, D., John, S., Khakhar, A., Ramakrishnan, B., & Ramamurthy, A. (2022). Handgrip strength: A simple and effective tool to predict mortality after liver transplantation. *Clinical Nutrition ESPEN*, *51*, 323-335.
66. Lu, Y., Li, G., Ferrari, P., Freisling, H., Qiao, Y., Wu, L., ... & Ke, C. (2022). Associations of handgrip strength with morbidity and all-cause mortality of cardiometabolic multimorbidity. *BMC medicine*, *20*(1), 191.
67. Luo, J. H., Zhang, T. M., Yang, L. L., Cai, Y. Y., & Yang, Y. (2023). Association between relative muscle strength and hypertension in middle-aged and older Chinese adults. *BMC Public Health*, *23*(1), 2087.
68. Lyyra, T. M., Leskinen, E., & Heikkinen, E. (2005). A cohort study found good respiratory, sensory and motor functions decreased mortality risk in older people. *Journal of clinical epidemiology*, *58*(5), 509-516.
69. Mänty, M., Mendes de Leon, C. F., Rantanen, T., Era, P., Pedersen, A. N., Ekmann, A., ... & Avlund, K. (2012). Mobility-related fatigue, walking speed, and muscle strength in older people. *Journals of Gerontology Series A: Biomedical Sciences and Medical Sciences*, *67*(5), 523-529.
70. Maslow, A. L., Sui, X., Colabianchi, N., Hussey, J., & Blair, S. N. (2010). Muscular strength and incident hypertension in normotensive and prehypertensive men. *Medicine and science in sports and exercise*, *42*(2), 288.
71. Maslow, A. L., Sui, X., Colabianchi, N., Hussey, J., & Blair, S. N. (2010). Muscular strength and incident hypertension in normotensive and prehypertensive men. *Medicine and science in sports and exercise*, *42*(2), 288.
72. Mattila, V. M., Niva, M., Kiuru, M., & Pihlajamäki, H. (2007). Risk factors for bone stress injuries: a follow-up study of 102,515 person-years. *Medicine and science in sports and exercise*, *39*(7), 1061-1066.
73. McGrath, R. P., Kraemer, W. J., Snih, S. A., & Peterson, M. D. (2018). Handgrip strength and health in aging adults. *Sports medicine*, *48*, 1993-2000.
74. McGrath, R., Vincent, B. M., Peterson, M. D., Jurivich, D. A., Dahl, L. J., Hackney, K. J., & Clark, B. C. (2020). Weakness may have a causal association with early mortality in older Americans: a matched cohort analysis. *Journal of the American Medical Directors Association*, *21*(5), 621-626.
75. McLeod, M., Breen, L., Hamilton, D. L., & Philp, A. (2016). Live strong and prosper: the importance of skeletal muscle strength for healthy ageing. *Biogerontology*, *17*, 497-510.
76. Momma, H., Kato, K., Sawada, S. S., Gando, Y., Kawakami, R., Miyachi, M., ... & Sone, H. (2021). Physical fitness and dyslipidemia among Japanese: a cohort study from the Niigata Wellness Study. *Journal of epidemiology*, *31*(4), 287-296.
77. Neumann, R. J., Ahrens, K. F., Kollmann, B., Goldbach, N., Chmitorz, A., Weichert, D., ... & Matura, S. (2022). The impact of physical fitness on resilience to modern life stress and the mediating role of general self-efficacy. *European archives of psychiatry and clinical neuroscience*, 1-14.
78. Newman, A. B., Kupelian, V., Visser, M., Simonsick, E. M., Goodpaster, B. H., Kritchevsky, S. B., ... & Harris, T. B. (2006). Strength, but not muscle mass, is associated with mortality in the health, aging and body composition study cohort. *The Journals of Gerontology Series A: Biological Sciences and Medical Sciences*, *61*(1), 72-77.
79. Nofuji, Y., Shinkai, S., Taniguchi, Y., Amano, H., Nishi, M., Murayama, H., ... & Suzuki, T. (2016). Associations of walking speed, grip strength, and standing balance with total and cause-specific mortality in a general population of Japanese elders. *Journal of the American Medical Directors Association*, *17*(2), 184-e1.
80. Nomura, Y., Shimada, M., Kakuta, E., Okada, A., Otsuka, R., Tomizawa, Y., ... & Hanada, N. (2020). Mortality-and health-related factors in a community-dwelling of oldest-older adults at the age of 90: a 10-year follow-up study. *International journal of environmental research and public health*, *17*(24), 9584.
81. Oksuzyan, A., Demakakos, P., Shkolnikova, M., Thinggaard, M., Vaupel, J. W., Christensen, K., & Shkolnikov, V. M. (2017). Handgrip strength and its prognostic value for mortality in Moscow, Denmark, and England. *PloS one*, *12*(9), e0182684.
82. Park, D., Lim, B., & Lee, O. (2024). Association Between Relative Grip Strength, Insulin Resistance, and Nonalcoholic Fatty Liver Disease Among Middle-Aged and Older Adults: A Prospective Cohort Study. *Metabolic Syndrome and Related Disorders*.
83. Park, S., Cho, J., Kim, D., Jin, Y., Lee, I., Hong, H., & Kang, H. (2019). Handgrip strength, depression, and all-cause mortality in Korean older adults. *BMC geriatrics*, *19*, 1-8.
84. Peterson, M. D., Duchowny, K., Meng, Q., Wang, Y., Chen, X., & Zhao, Y. (2017). Low normalized grip strength is a biomarker for cardiometabolic disease and physical disabilities among US and Chinese adults. *Journals of Gerontology Series A: Biomedical Sciences and Medical Sciences*, *72*(11), 1525-1531.
85. Polo-López, A., Calatayud, J., Núñez-Cortés, R., Andersen, L. L., Moya-Ramón, M., & López-Bueno, R. (2023). Dose-response association between handgrip strength and hypertension: a longitudinal study of 76,503 European older adults. *Current Problems in Cardiology*, *48*(9), 101813.
86. Rantanen, T., Harris, T., Leveille, S. G., Visser, M., Foley, D., Masaki, K., & Guralnik, J. M. (2000). Muscle strength and body mass index as long-term predictors of mortality in initially healthy men. *The Journals of Gerontology Series A: Biological Sciences and Medical Sciences*, *55*(3), M168-M173.
87. Rantanen, T., Masaki, K., He, Q., Ross, G. W., Willcox, B. J., & White, L. (2012). Midlife muscle strength and human longevity up to age 100 years: a 44-year prospective study among a decedent cohort. *Age*, *34*, 563-570.
88. Rey-Lopez, J. P., Frederick, K. W., Foster, H. M., Petermann-Rocha, F., Sattar, N., Pell, J. P., ... & Celis-Morales, C. A. (2020). Does the association between physical capability and mortality differ by deprivation? Findings from the UK Biobank population-based cohort study. *Journal of Sports Sciences*, *38*(23), 2732-2739.
89. Rolland, Y., Lauwers-Cances, V., Cesari, M., Vellas, B., Pahor, M., & Grandjean, H. (2006). Physical performance measures as predictors of mortality in a cohort of community-dwelling older French women. *European journal of epidemiology*, *21*, 113-122.
90. Ruiz, J. R., Sui, X., Lobelo, F., Lee, D. C., Morrow Jr, J. R., Jackson, A. W., ... & Blair, S. N. (2009). Muscular strength and adiposity as predictors of adulthood cancer mortality in men. *Cancer Epidemiology Biomarkers & Prevention*, *18*(5), 1468-1476.
91. Ruiz, J. R., Sui, X., Lobelo, F., Morrow, J. R., Jackson, A. W., Sjöström, M., & Blair, S. N. (2008). Association between muscular strength and mortality in men: prospective cohort study. *Bmj*, *337*.
92. Sagarra-Romero L, Gonzalez-Aguero A, Navarrete-Villanueva D, et al. (2017). Associations between lean mass, strength and mortality in the elderly: The EXERNET study. *Journal of cachexia, sarcopenia and muscle.* *8*(6):1005-1006.
93. Samuel, D., Rowe, P., Hood, V., & Nicol, A. (2012). The relationships between muscle strength, biomechanical functional moments and health-related quality of life in non-elite older adults. *Age and ageing*, *41*(2), 224-230.
94. Sasaki, H., Kasagi, F., Yamada, M., & Fujita, S. (2007). Grip strength predicts cause-specific mortality in middle-aged and elderly persons. *The American journal of medicine*, *120*(4), 337-342.
95. Sayer, A. A., Syddall, H. E., Dennison, E. M., Martin, H. J., Phillips, D. I. W., Cooper, C., & Byrne, C. D. (2007). Grip strength and the metabolic syndrome: findings from the Hertfordshire Cohort Study. *QJM: An International Journal of Medicine*, *100*(11), 707-713.
96. Scott, D., Park, M. S., Kim, T. N., Ryu, J. Y., Hong, H. C., Yoo, H. J., ... & Choi, K. M. (2016). Associations of low muscle mass and the metabolic syndrome in Caucasian and Asian middle-aged and older adults. *The Journal of nutrition, health and aging*, *20*(3), 248-255.
97. Shen, C., Lu, J., Xu, Z., Xu, Y., & Yang, Y. (2020). Association between handgrip strength and the risk of new-onset metabolic syndrome: A population-based cohort study. *BMJ open*, *10*(10), e041384.
98. Shen, C., Lu, J., Xu, Z., Xu, Y., & Yang, Y. (2020). Association between handgrip strength and the risk of new-onset metabolic syndrome: A population-based cohort study. *BMJ open*, *10*(10), e041384.
99. Shen, C., Lu, J., Xu, Z., Xu, Y., & Yang, Y. (2020). Association between handgrip strength and the risk of new-onset metabolic syndrome: A population-based cohort study. *BMJ open*, *10*(10), e041384.
100. Shrier, I. (2010). Muscle strength and body size and later cerebrovascular and coronary heart disease. *Clinical Journal of Sport Medicine*, *20*(2), 131.
101. Song, P., Han, P., Zhao, Y., Zhang, Y., Wang, L., Tao, Z., ... & Guo, Q. (2021). Muscle mass rather than muscle strength or physical performance is associated with metabolic syndrome in community-dwelling older Chinese adults. *BMC geriatrics*, *21*, 1-9.
102. Stenholm, S., Mehta, N. K., Elo, I. T., Heliövaara, M., Koskinen, S., & Aromaa, A. (2014). Obesity and muscle strength as long-term determinants of all-cause mortality—a 33-year follow-up of the Mini-Finland Health Examination Survey. *International journal of obesity*, *38*(8), 1126-1132.
103. Stevenson, J. M., Weber, C. L., Smith, J. T., Dumas, G. A., & Albert, W. J. (2001). A longitudinal study of the development of low back pain in an industrial population. *Spine*, *26*(12), 1370-1377.
104. Strand, B. H., Cooper, R., Bergland, A., Jørgensen, L., Schirmer, H., Skirbekk, V., & Emaus, N. (2016). The association of grip strength from midlife onwards with all-cause and cause-specific mortality over 17 years of follow-up in the Tromsø Study. *J Epidemiol Community Health*, *70*(12), 1214-1221.
105. Sugai, K., Michikawa, T., Takebayashi, T., & Nishiwaki, Y. (2019). Association between muscle strength, mobility, and the progression of hyperkyphosis in the elderly: the Kurabuchi Cohort Study. *The Journals of Gerontology: Series A*, *74*(12), 1987-1992.
106. Takata, Y., Ansai, T., Akifusa, S., Soh, I., Yoshitake, Y., Kimura, Y., ... & Takehara, T. (2007). Physical fitness and 4-year mortality in an 80-year-old population. *The Journals of Gerontology Series A: Biological Sciences and Medical Sciences*, *62*(8), 851-858.
107. Takata, Y., Ansai, T., Soh, I., Awano, S., Yoshitake, Y., Kimura, Y., ... & Nishihara, T. (2012). Physical fitness and 6.5-year mortality in an 85-year-old community-dwelling population. *Archives of gerontology and geriatrics*, *54*(1), 28-33.
108. Takata, Y., Shimada, M., Ansai, T., Yoshitake, Y., Nishimuta, M., Nakagawa, N., ... & Miyazaki, H. (2012). Physical performance and 10-year mortality in a 70-year-old community-dwelling population. *Aging clinical and experimental research*, *24*, 257-264.
109. Van Ancum, J. M., Pijnappels, M., Jonkman, N. H., Scheerman, K., Verlaan, S., Meskers, C. G., & Maier, A. B. (2018). Muscle mass and muscle strength are associated with pre-and post-hospitalization falls in older male inpatients: a longitudinal cohort study. *BMC geriatrics*, *18*, 1-7.
110. Veronese, N., Stubbs, B., Fontana, L., Trevisan, C., Bolzetta, F., Rui, M. D., ... & Sergi, G. (2017). A comparison of objective physical performance tests and future mortality in the elderly people. *Journals of Gerontology Series A: Biomedical Sciences and Medical Sciences*, *72*(3), 362-368.
111. Wang, J., Yang, Y., Su, Q., Wang, J., Zeng, H., Chen, Y., ... & Wang, Y. (2024). Association between muscle strength and cardiometabolic multimorbidity risk among middle-aged and older Chinese adults: a nationwide longitudinal cohort study. *BMC Public Health*, *24*(1), 2012.
112. Wang, Y. C., Liang, C. K., Hsu, Y. H., Peng, L. N., Chu, C. S., Liao, M. C., ... & Lin, Y. T. (2019). Synergistic effect of low handgrip strength and malnutrition on 4-year all-cause mortality in older males: A prospective longitudinal cohort study. *Archives of Gerontology and Geriatrics*, *83*, 217-222.
113. Wijndaele, K., Duvigneaud, N., Matton, L., Duquet, W., Thomis, M., Beunen, G., ... & Philippaerts, R. M. (2007). Muscular strength, aerobic fitness, and metabolic syndrome risk in Flemish adults. *Medicine and science in sports and exercise*, *39*(2), 233.
114. Wu, H., Liu, M., Chi, V. T. Q., Wang, J., Zhang, Q., Liu, L., ... & Niu, K. (2019). Handgrip strength is inversely associated with metabolic syndrome and its separate components in middle aged and older adults: a large-scale population-based study. *Metabolism*, *93*, 61-67.
115. Wu, Y., Wang, W., Liu, T., & Zhang, D. (2017). Association of grip strength with risk of all-cause mortality, cardiovascular diseases, and cancer in community-dwelling populations: a meta-analysis of prospective cohort studies. *Journal of the American Medical Directors Association*, *18*(6), 551-e17.
116. Yang, E. J., Lim, S., Lim, J. Y., Kim, K. W., Jang, H. C., & Paik, N. J. (2012). Association between muscle strength and metabolic syndrome in older Korean men and women: the Korean Longitudinal Study on Health and Aging. *Metabolism*, *61*(3), 317-324.
117. Yi, D., Khang, A. R., Lee, H. W., Son, S. M., & Kang, Y. H. (2018). Relative handgrip strength as a marker of metabolic syndrome: the Korea National Health and Nutrition Examination Survey (KNHANES) VI (2014–2015). *Diabetes, metabolic syndrome and obesity: targets and therapy*, 227-240.
118. Zhang, W., Zhao, Z., Sun, X., & Tian, X. (2021). Prevalence of metabolic syndrome according to absolute and relative values of muscle strength in middle-aged and elderly women. *International journal of environmental research and public health*, *18*(17), 9073.

- **Wrong study design (n=75)**

1. Ashdown-Franks, G., Stubbs, B., Koyanagi, A., Schuch, F., Firth, J., Veronese, N., & Vancampfort, D. (2019). Handgrip strength and depression among 34,129 adults aged 50 years and older in six low-and middle-income countries. *Journal of affective disorders*, *243*, 448-454.
2. Bertoni, M., Maggi, S., Manzato, E., Veronese, N., & Weber, G. (2018). Depressive symptoms and muscle weakness: A two-way relation? *Experimental gerontology*, *108*, 87-91.
3. Bing, W. U., Lyu, Y. B., Cao, Z. J., Yuan, W. E. I., Shi, W. Y., Xiang, G. A. O., ... & Shi, X. M. (2021). Associations of sarcopenia, handgrip strength and calf circumference with cognitive impairment among Chinese older adults. *Biomedical and Environmental Sciences*, *34*(11), 859-870.
4. Buckinx, F., Croisier, J. L., Charles, A., Petermans, J., Reginster, J. Y., Rygaert, X., & Bruyere, O. (2019). Normative data for isometric strength of 8 different muscle groups and their usefulness as a predictor of loss of autonomy among physically active nursing home residents: the SENIOR cohort. *Journal of Musculoskeletal & Neuronal Interactions*, *19*(3), 258.
5. Cao, J., Zhao, F., & Ren, Z. (2021). Association between changes in muscle strength and risk of depressive symptoms among chinese female College students: a prospective cohort study. *Frontiers in public health*, *9*, 616750.
6. Cawthon, P. M., Fox, K. M., Gandra, S. R., Delmonico, M. J., Chiou, C. F., Anthony, M. S., ... & Health, Aging and Body Composition Study. (2009). Do muscle mass, muscle density, strength, and physical function similarly influence risk of hospitalization in older adults? *Journal of the American Geriatrics Society*, *57*(8), 1411-1419.
7. Chen, W. L., Peng, T. C., Sun, Y. S., Yang, H. F., Liaw, F. Y., Wu, L. W., ... & Kao, T. W. (2015). Examining the association between quadriceps strength and cognitive performance in the elderly. *Medicine*, *94*(32), e1335.
8. Ciccolo, J. T., SantaBarbara, N. J., Dunsiger, S. I., Busch, A. M., & Bartholomew, J. B. (2016). Muscular strength is associated with self-esteem in college men but not women. *Journal of health psychology*, *21*(12), 3072-3078.
9. Crump, C., Sundquist, J., Winkleby, M. A., & Sundquist, K. (2017). Aerobic fitness, muscular strength and obesity in relation to risk of heart failure. *Heart*, *103*(22), 1780-1787.
10. Cui, M., Zhang, S., Liu, Y., Gang, X., & Wang, G. (2021). Grip strength and the risk of cognitive decline and dementia: a systematic review and meta-analysis of longitudinal cohort studies. *Frontiers in aging neuroscience*, *13*, 625551.
11. de Asteasu, M. L. S., Steffens, T., Ramirez-Velez, R., Cadore, E. L., Izquierdo, M., & Pietta-Dias, C. (2022). Low handgrip strength is associated with higher cancer prevalence in frail nonagenarians and centenarians. *Experimental Gerontology*, *165*, 111862.
12. De Lima, T. R., Martins, P. C., Guerra, P. H., & Santos Silva, D. A. (2021). Muscular strength and cardiovascular risk factors in adults: A systematic review. *The Physician and Sportsmedicine*, *49*(1), 18-30.
13. Delinocente, M. L. B., de Carvalho, D. H. T., de Oliveira Máximo, R., Chagas, M. H. N., Santos, J. L. F., de Oliveira Duarte, Y. A., ... & da Silva Alexandre, T. (2021). Accuracy of different handgrip values to identify mobility limitation in older adults. *Archives of gerontology and geriatrics*, *94*, 104347.
14. den Ouden, M. E., Schuurmans, M. J., Arts, I. E., & van der Schouw, Y. T. (2011). Physical performance characteristics related to disability in older persons: a systematic review. *Maturitas*, *69*(3), 208-219.
15. Dixe, M. D. A., Madeira, C., Alves, S., Henriques, M. A., & Baixinho, C. L. (2021). Gait ability and muscle strength in institutionalized older persons with and without cognitive decline and association with falls. *International journal of environmental research and public health*, *18*(21), 11543.
16. Ensrud, K. E., Nevitt, M. C., Yunis, C., Cauley, J. A., Seeley, D. G., Fox, K. M., & Cummings, S. R. (1994). Correlates of impaired function in older women. *Journal of the American Geriatrics Society*, *42*(5), 481-489.
17. Frith, E., & Loprinzi, P. D. (2018). The association between lower extremity muscular strength and cognitive function in a national sample of older adults. *Journal of lifestyle medicine*, *8*(2), 99.
18. García-Hermoso, A., Cavero-Redondo, I., Ramírez-Vélez, R., Ruiz, J. R., Ortega, F. B., Lee, D. C., & Martínez-Vizcaíno, V. (2018). Muscular strength as a predictor of all-cause mortality in an apparently healthy population: a systematic review and meta-analysis of data from approximately 2 million men and women. *Archives of physical medicine and rehabilitation*, *99*(10), 2100-2113.
19. García‐Hermoso, A., Ramírez‐Vélez, R., Peterson, M. D., Lobelo, F., Cavero‐Redondo, I., Correa‐Bautista, J. E., & Martínez‐Vizcaíno, V. (2018). Handgrip and knee extension strength as predictors of cancer mortality: a systematic review and meta‐analysis. *Scandinavian journal of medicine & science in sports*, *28*(8), 1852-1858.
20. Ghiotto, L., Muollo, V., Tatangelo, T., Schena, F., & Rossi, A. P. (2022). Exercise and physical performance in older adults with sarcopenic obesity: A systematic review. *Frontiers in endocrinology*, *13*, 913953.
21. Gonzalez-Bautista, E., de Souto Barreto, P., Salinas-Rodriguez, A., Manrique-Espinoza, B., Sourdet, S., Rolland, Y., ... & Vellas, B. (2023). Development and validation of a cutoff for the chair stand test as a screening for mobility impairment in the context of the integrated care for older people program. *The Journals of Gerontology: Series A*, *78*(1), 104-110.
22. Gopinath, B., Kifley, A., Liew, G., & Mitchell, P. (2017). Handgrip strength and its association with functional independence, depressive symptoms and quality of life in older adults. *Maturitas*, *106*, 92-94.
23. Gordon, B. R., McDowell, C. P., Lyons, M., & Herring, M. P. (2019). Associations between grip strength and generalized anxiety disorder in older adults: results from the Irish longitudinal study on ageing. *Journal of affective disorders*, *255*, 136-141.
24. Granacher, U., Gollhofer, A., Hortobágyi, T., Kressig, R. W., & Muehlbauer, T. (2013). The importance of trunk muscle strength for balance, functional performance, and fall prevention in seniors: a systematic review. *Sports medicine*, *43*, 627-641.
25. Gu, Y., Li, X., Zhang, Q., Liu, L., Meng, G., Wu, H., ... & Niu, K. (2021). Grip strength and depressive symptoms in a large-scale adult population: the TCLSIH cohort study. *Journal of affective disorders*, *279*, 222-228.
26. Gubelmann, C., Vollenweider, P., & Marques-Vidal, P. (2017). Association of grip strength with cardiovascular risk markers. *European journal of preventive cardiology*, *24*(5), 514-521.¡.
27. Huang, X., Ma, J., Ying, Y., Liu, K., Jing, C., & Hao, G. (2021). The handgrip strength and risk of depressive symptoms: a meta-analysis of prospective cohort studies. *Quality of Life Research*, *30*(9), 2467-2474.
28. Ji, J. J., Zhao, M. J., Xiao, M. L., Zhang, H. E., Tan, Q., Cheng, Y. R., & Lu, F. (2024). Association between relative muscle strength and cardiovascular disease among middle-aged and older adults in China. *BMC Public Health*, *24*(1), 1928.
29. Jiang, D., Chen, X., Huang, J., Wu, L., Chen, Y., Feng, H., & Hu, M. (2023). Associations of sarcopenia, sarcopenia parameters and motoric cognitive risk syndrome in Chinese older adults. *Frontiers in Aging Neuroscience*, *15*, 1302879.
30. Jin, Y. L., Xu, L., Jiang, C. Q., Zhang, W. S., Pan, J., Zhu, F., ... & Lam, T. H. (2022). Association of hand grip strength with mild cognitive impairment in middle-aged and older people in guangzhou biobank cohort study. *International Journal of Environmental Research and Public Health*, *19*(11), 6464.
31. Jonkman NH, Colpo M, Klenk J, et al. (2018). Predicting the onset of functional decline in people aged 65-75 years old: Pooled analysis of four European cohort studies. *European Geriatric Medicine*, *9*(1), 11-S12.
32. Kato, S., Murakami, H., Demura, S., Yoshioka, K., Shinmura, K., Yokogawa, N., ... & Tsuchiya, H. (2019). Abdominal trunk muscle weakness and its association with chronic low back pain and risk of falling in older women. *BMC musculoskeletal disorders*, *20*, 1-8.
33. Kim, H., Jeong, W., Kim, S. H., Park, Y. S., Jang, S. I., & Park, E. C. (2022). Association between changes in handgrip strength and depression in Korean adults: a longitudinal panel study. *Scientific reports*, *12*(1), 13643.
34. Kim, H., Kim, S. H., Jeong, W., Jang, S. I., Park, E. C., & Kim, Y. (2021). Association between change in handgrip strength and cognitive function in Korean adults: a longitudinal panel study. *BMC geriatrics*, *21*, 1-8.
35. Kim, J., Kang, S., Hong, H., Kang, H., Kim, J. H., & Woo, S. K. (2022). Muscle strength moderates the relationship between nutritional health risk and depression in Korean older adults. *Nutrients*, *14*(3), 665.
36. Kim, K. M., Lim, S., Oh, T. J., Moon, J. H., Choi, S. H., Lim, J. Y., ... & Jang, H. C. (2018). Longitudinal changes in muscle mass and strength, and bone mass in older adults: gender-specific associations between muscle and bone losses. *The Journals of Gerontology: Series A*, *73*(8), 1062-1069.
37. Kim, Y., Gonzales, J. U., & Reddy, P. H. (2020). An Investigation of Short-Term Longitudinal Associations Between Handgrip Strength and Cardiovascular Disease Biomarkers Among Middle-Aged to Older Adults: A Project FRONTIER Study. *Journal of Aging and Physical Activity*, *28*(1), 9-17.
38. Kobayashi-Cuya, K. E., Sakurai, R., Suzuki, H., Ogawa, S., Takebayashi, T., & Fujiwara, Y. (2018). Observational evidence of the association between handgrip strength, hand dexterity, and cognitive performance in community-dwelling older adults: a systematic review. *Journal of epidemiology*, *28*(9), 373-381.
39. Lawman, H. G., Troiano, R. P., Perna, F. M., Wang, C. Y., Fryar, C. D., & Ogden, C. L. (2016). Associations of relative handgrip strength and cardiovascular disease biomarkers in US adults, 2011–2012. *American journal of preventive medicine*, *50*(6), 677-683.
40. Lee, M. R., Jung, S. M., Kim, H. S., & Kim, Y. B. (2018). Association of muscle strength with cardiovascular risk in Korean adults: Findings from the Korea National Health and Nutrition Examination Survey (KNHANES) VI to VII (2014–2016). *Medicine*, *97*(47), e13240.
41. Lee, S., Oh, J. W., Son, N. H., & Chung, W. (2022). Association between handgrip strength and cognitive function in older adults: Korean Longitudinal Study of Aging (2006–2018). *International Journal of Environmental Research and Public Health*, *19*(3), 1048.
42. Lin, P. S., Hsieh, C. C., Cheng, H. S., Tseng, T. J., & Su, S. C. (2016). Association between physical fitness and successful aging in Taiwanese older adults. *PloS one*, *11*(3), e0150389.
43. Lipsitz, L. A., Nakajima, I., Gagnon, M., Hirayama, T., Connelly, C. M., Izumo, H., & Hirayama, T. (1994). Muscle strength and fall rates among residents of Japanese and American nursing homes: An international cross‐cultural study. *Journal of the American Geriatrics Society*, *42*(9), 953-959.
44. Makizako, H., Shimada, H., Doi, T., Tsutsumimoto, K., Nakakubo, S., Hotta, R., & Suzuki, T. (2017). Predictive cutoff values of the five-times sit-to-stand test and the timed “up & go” test for disability incidence in older people dwelling in the community. *Physical therapy*, *97*(4), 417-424.
45. Marques, A., Gomez-Baya, D., Peralta, M., Frasquilho, D., Santos, T., Martins, J., ... & Gaspar de Matos, M. (2020). The effect of muscular strength on depression symptoms in adults: a systematic review and meta-analysis. *International journal of environmental research and public health*, *17*(16), 5674.
46. Moreland, J. D., Richardson, J. A., Goldsmith, C. H., & Clase, C. M. (2004). Muscle weakness and falls in older adults: a systematic review and meta‐analysis. *Journal of the american Geriatrics Society*, *52*(7), 1121-1129.
47. Muraki, S. (2013). Fall risk and fracture. The association of physical performance with falls in the elderly. *Clinical calcium*, *23*(5), 713-718.
48. Noh, H. M., & Park, Y. S. (2020). Handgrip strength, dynapenia, and mental health in older Koreans. *Scientific reports*, *10*(1), 4004.
49. Øiestad, B. E., Juhl, C. B., Culvenor, A. G., Berg, B., & Thorlund, J. B. (2022). Knee extensor muscle weakness is a risk factor for the development of knee osteoarthritis: an updated systematic review and meta-analysis including 46 819 men and women. *British journal of sports medicine*, *56*(6), 349-355.
50. Ottenbacher, K. J., Ostir, G. V., Peek, M. K., Snih, S. A., Raji, M. A., & Markides, K. S. (2005). Frailty in older mexican americans. *Journal of the American Geriatrics Society*, *53*(9), 1524-1531.
51. Pan, F., Tian, J., Cicuttini, F., & Jones, G. (2020). Muscle function and power but not mass protect against more severe knee pain trajectories. *Osteoarthritis and Cartilage*, *28*, S145-S146.
52. Park, D., Jo, H., Yoon, C. H., Lee, E. S., Oh, M. K., & Lee, C. H. (2019). Fall risk assessment of rural elderly population in Korea. *Annals of rehabilitation medicine*, *43*(3), 269-278.
53. Parsons, C. M., Edwards, M. H., Cooper, C., Dennison, E. M., & Ward, K. A. (2020). Are jumping mechanography assessed muscle force and power, and traditional physical capability measures associated with falls in older adults? Results from the Hertfordshire cohort study. *Journal of Musculoskeletal & Neuronal Interactions*, *20*(2), 168.
54. Pasdar, Y., Darbandi, M., Mirtaher, E., Rezaeian, S., Najafi, F., & Hamzeh, B. (2019). Associations between muscle strength with different measures of obesity and lipid profiles in men and women: results from RaNCD cohort study. *Clinical nutrition research*, *8*(2), 148-158.
55. Rijk, J. M., Roos, P. R., Deckx, L., van den Akker, M., & Buntinx, F. (2016). Prognostic value of handgrip strength in people aged 60 years and older: a systematic review and meta‐analysis. *Geriatrics & gerontology international*, *16*(1), 5-20.
56. Sayer, A. A., Syddall, H. E., Martin, H. J., Dennison, E. M., Roberts, H. C., & Cooper, C. (2006). Is grip strength associated with health-related quality of life? Findings from the Hertfordshire Cohort Study. *Age and ageing*, *35*(4), 409-415.
57. Schneider, P., Schwab, M., & Hänscheid, H. (2011). Identification of factors associated with risk of fall using a force platform and power spectrum analysis technique. *Journal of biomechanics*, *44*(10), 2008-2012.
58. Shaughnessy, K. A., Hackney, K. J., Clark, B. C., Kraemer, W. J., Terbizan, D. J., Bailey, R. R., & McGrath, R. (2020). A narrative review of handgrip strength and cognitive functioning: bringing a new characteristic to muscle memory. *Journal of Alzheimer's Disease*, *73*(4), 1265-1278.
59. Silventoinen, K., Magnusson, P. K., Tynelius, P., Batty, G. D., & Rasmussen, F. (2009). Association of body size and muscle strength with incidence of coronary heart disease and cerebrovascular diseases: a population-based cohort study of one million Swedish men. *International journal of epidemiology*, *38*(1), 110-118.
60. Sirola, J., Tuppurainen, M., Honkanen, R., Jurvelin, J. S., & Kröger, H. (2005). Associations between grip strength change and axial postmenopausal bone loss—a 10-year population-based follow-up study. *Osteoporosis international*, *16*, 1841-1848.
61. Søgaard, A. J., Magnus, J. H., Bjørnerem, Å., Holvik, K., Ranhoff, A. H., Emaus, N., ... & Strand, B. H. (2020). Grip strength in men and women aged 50–79 years is associated with non-vertebral osteoporotic fracture during 15 years follow-up: The Tromsø Study 1994–1995. *Osteoporosis International*, *31*, 131-140.
62. Stel, V. S., Pluijm, S. M., Deeg, D. J., Smit, J. H., Bouter, L. M., & Lips, P. (2003). A classification tree for predicting recurrent falling in community‐dwelling older persons. *Journal of the American Geriatrics Society*, *51*(10), 1356-1364.
63. Sui, S. X., Holloway-Kew, K. L., Hyde, N. K., Williams, L. J., Leach, S., & Pasco, J. A. (2020). Muscle strength and gait speed rather than lean mass are better indicators for poor cognitive function in older men. *Scientific Reports*, *10*(1), 10367.
64. Suija, K., Timonen, M., Suviola, M., Jokelainen, J., Järvelin, M. R., & Tammelin, T. (2013). The association between physical fitness and depressive symptoms among young adults: results of the Northern Finland 1966 birth cohort study. *BMC Public Health*, *13*, 1-7.
65. Tainaka, K., & Aoki, J. (2007). Fitness-related factors associated with falling in older women. *Japanese Journal of Physical Fitness and Sports Medicine*, 279-286.
66. Triana-Reina, H. R., & Ramírez-Vélez, R. (2013). Association of muscle strength with early markers of cardiovascular risk in sedentary adults. *Endocrinología y Nutrición (English Edition)*, *60*(8), 433-438.
67. Ueno, M., Kawai, S., Mino, T., & Kamoshita, H. (2006). Systematic review of fall-related factors among the house-dwelling elderly in Japan. *Nihon Ronen Igakkai zasshi. Japanese journal of geriatrics*, *43*(1), 92-101.
68. Vancampfort, D., Stubbs, B., Firth, J., Smith, L., Swinnen, N., & Koyanagi, A. (2019). Associations between handgrip strength and mild cognitive impairment in middle‐aged and older adults in six low‐and middle‐income countries. *International journal of geriatric psychiatry*, *34*(4), 609-616.
69. Villamizar-Pita, P. C., Angarita-Fonseca, A., de Souza, H. C. D., Martínez-Rueda, R., Villamizar García, M. C., & Sánchez-Delgado, J. C. (2022). Handgrip strength is associated with risk of falls in physically active older women. *Health care for women international*, *43*(10-11), 1301-1314.
70. Vodička, T., Bozděch, M., Vespalec, T., Piler, P., & Paludo, A. C. (2024). Association between muscle strength and depression in a cohort of young adults. *Plos one*, *19*(5), e0303925.
71. Volaklis, K. A., Halle, M., & Meisinger, C. (2015). Muscular strength as a strong predictor of mortality: a narrative review. *European journal of internal medicine*, *26*(5), 303-310.
72. Volaklis, K., Mamadjanov, T., Meisinger, C., & Linseisen, J. (2019). Association between muscular strength and depressive symptoms: a narrative review. *Wiener Klinische Wochenschrift*, *131*(11), 255-264.
73. Wagner, P., Chapurlat, R., Ecochard, R., & Szulc, P. (2018). Low muscle strength and mass is associated with the accelerated decline of bone microarchitecture at the distal radius in older men: the prospective STRAMBO study. *Journal of Bone and Mineral Research*, *33*(9), 1630-1640.
74. Wang, D. X., Yao, J., Zirek, Y., Reijnierse, E. M., & Maier, A. B. (2020). Muscle mass, strength, and physical performance predicting activities of daily living: a meta‐analysis. *Journal of cachexia, sarcopenia and muscle*, *11*(1), 3-25.
75. Wang, Y., Lee, D. C., Brellenthin, A. G., Sui, X., Church, T. S., Lavie, C. J., & Blair, S. N. (2019, April). Association of muscular strength and incidence of type 2 diabetes. In *Mayo Clinic Proceedings* (Vol. 94, No. 4, pp. 643-651). Elsevier.

- **No original paper (n=43)**

1. Alajlouni, D., Bliuc, D., Tran, T., Nguyen, T., Eisman, J., & Center, J. (2019, May). Role of individual components of sarcopenia in fracture risk prediction in elderly women and men. In *osteoporosis international* (vol. 30, pp. S91-s91). 236 Grays Inn Rd, 6th Floor, London Wc1x 8hl, England: Springer London Ltd.
2. Anderson-Ranberg, K., Ryg, J., Vestergaard, S., Eriksen, M. L., & Masud, T. (2013, August). Decreased grip strength, slower walking speed and inability to stand from chair without using arms predict falls in a large european ageing cohort. In *Age and Ageing* (Vol. 42, pp. 17-17). Great Clarendon St, Oxford Ox2 6dp, England: Oxford Univ Press.
3. Arai T. (2017, September) Factors affecting the subjective well-being of Japanese community-dwelling elderly at one-year follow-up: A preliminary study. *European Geriatric Medicine*, *8*(1):S101-S102.
4. Bahat-Öztürk, G., Erbaş Saçar, D., Kılıç, C., Karan, M., & Cruz-Jentoft, A. Prediction of reduced function and physical performance in older adults with low grip strength.
5. Bullain, S., Corrada, M., & Kawas, C. (2013). Poor Physical Performance Increases the Risk of Dementia in the Oldest-Old (P03. 096).
6. Camargo, E., Beiser, A., Tan, Z., Au, R., DeCarli, C., Pikula, A., ... & Seshadri, S. (2012). Walking speed, handgrip strength and risk of dementia and stroke: The Framingham Offspring Study (S24. 003).
7. Chen, T., Narazaki, K., Chen, S., Haeuchi, Y., & Kumagai, S. (2016). P1‐029: The Dynamic Association Between Physical and Cognitive Functions in Non‐Demented Community‐Dwelling Older Adults: A 2‐YEAR Longitudinal Analysis. *Alzheimer's & Dementia*, *12*, P412-P412.
8. Conway, A. T. (1998). *Relationship of strength and balance impairments to function in the elderly*. MGH Institute of Health Professions.
9. Cooper, R., Ward, K. A., & Sayer, A. A. (2023). A Life Course Approach to Musculoskeletal Ageing. *A Life Course Approach to Women's Health*, 115.
10. De Buyser, S., Petrovic, M., Taes, Y., Lapauw, B., Toye, K., Kaufman, J., & Goemaere, S. (2015). Persistent decline over 3 years in physical function predicts 15-year mortality in ambulatory older men. In *11th International congress of the European Union Geriatric Medicine Society* (Vol. 6, No. suppl. 1, pp. S8-S8).
11. Ekstubbe, H., Bartosch, P., McGuigan, F., & Akesson, K. E. (2020, November). A Longitudinal Study of Sarcopenia in Older Community Dwelling Women. In *JOURNAL OF BONE AND MINERAL RESEARCH* (Vol. 35, pp. 316-316). 111 RIVER ST, HOBOKEN 07030-5774, NJ USA: WILEY.
12. Fuggle, N. R., Jameson, K. A., Edwards, M. H., Dennison, E. M., & Cooper, C. (2018, April). Relationships between muscle size, strength and function and the risk of falls and fractures: findings from the hertfordshire cohort study. In *osteoporosis international* (vol. 29, pp. S66-s67). 236 grays inn rd, 6th floor, London wc1x 8hl, england: springer London ltd.
13. Fuggle, N. R., Westbury, L. D., Jameson, K. A., Syddall, H. E., Edwards, M. H., Ward, K. A., ... & Cooper, C. (2019, July). Which muscle parameters are longitudinally associated with future knee osteoarthritis outcomes? Findings from the hertfordshire cohort study. In *osteoporosis international* (vol. 30, pp. S171-s171). 236 grays inn rd, 6th floor, London wc1x 8hl, England: springer London ltd.
14. Greslou, F., Baptista, G., Cristol, J. P., & Jeandel, C. (2012). The insulin resistance paradox in elderly people. A new case of reverse epidemiology? *European Geriatric Medicine*, (3), S85.
15. Hastings, E., Chen, R., Ross, G. W., Abbott, R., Petrovitch, H., Wen, A. B., ... & Masaki, K. (2017, May). Late-Life Handgrip Strength Predicts Incident Stroke: The Kuakini Honolulu Heart Program. In *Journal of The American Geriatrics Society* (Vol. 65, Pp. S115-S115). 111 River St, Hoboken 07030-5774, Nj Usa: Wiley.
16. Hovda III, T. J. (2002). Lower extremity strength and its association with physical function and disability.
17. Iki, M., Fujita, Y., Tamaki, J., Kouda, K., Yura, A., Sato, Y., ... & Kurumatani, N. (2015, February). Incident clinical fractures were associated with increased risk of death after adjustment for frailty indices in community-dwelling elderly japanese men: a cohort study. In *Osteoporosis International* (Vol. 26, pp. S91-S92). 236 Grays Inn Rd, 6th Floor, London Wc1x 8hl, England: Springer London Ltd.
18. Imaeva, A., Shalnova, S., Kapustina, A., Balanova, Y., & Shkolnikov, V. (2020, February). Associations between handgrip strength, diabetes mellitus, and all-cause mortality among the population of 55 years and older. In *Diabetes Technology & Therapeutics* (Vol. 22, pp. A205-A205). 140 HUGUENOT Street, 3rd Fl, New Rochelle, Ny 10801 Usa: Mary Ann Liebert, Inc.
19. Jhee, J. H., Lee, G. Y., Yoon, S., Choi, H. Y., & Park, H. C. (2020, June). Greater muscle strength is associated with lower risk of chronic kidney disease. In *nephrology dialysis transplantation* (Vol. 35, pp. 1010-1010). Great Clarendon St, Oxford Ox2 6dp, England: Oxford Univ Press.
20. Kim, J. E., Kang, J. H., Lee, K. E., Park, D. J., & Lee, S. S. (2017). SAT0518 Association between grip strength and hand and knee radiographic osteoarthritis in older adults: data from the dong-gu study.
21. Kouvari, M., Panagiotakos, D. D. B., Chrysohoou, C., Georgousopoulou, E., Notara, V., Tousoulis, D., & Pitsavos, C. T Toril Dammen1, C Papageorgiou2, E Sverre3, J Munkhaugen3 1University of Oslo, Oslo, Norway, 2Priory Hospital, Liverpool, United Kingdom of Great Britain & Northern Ireland, 3Drammen Hospital, Drammen, Norway.
22. Lee, D. C., Brellenthin, A., Sui, X., & Blair, S. (2018). Abstract MP32: muscular strength and type 2 diabetes prevention. *Circulation*, *137*(suppl_1), AMP32-AMP32.
23. Lobo, D., Alvarenga, J. C., Domiciano, D. S., Benatti, F., Gualano, B., & Pereira, R. M. (2017). Associations between muscle mass and function with bone microarchitecture and bone strength in postmenopausal women. In *Arthritis & Rheumatology*. WILEY.
24. Locquet, M., Beaudart, C., Croisier, J. L., Reginster, J. Y., & Bruyère, O. (2019). Determinants and health consequences of a rapid muscle health decline in older adults from the SacoPhAge study. *Osteoporosis International*, *30*(S2).
25. Manda, C., Nakanga, W. P., Mkandawire, J., Muula, A. S., Nyirenda, M. J., Crampin, A. C., & Wagatsuma, Y. (2021, September). Muscle strength as a simple measure for screening prediabetes and type 2 diabetes mellitus risk among adults in Malawi. In *TROPICAL MEDICINE & INTERNATIONAL HEALTH* (Vol. 26, Pp. 167-168). 111 RIVER ST, HOBOKEN 07030-5774, NJ USA: WILEY.
26. Marko, M. (2012). Lower extremities muscle strength associated with early signs of mobility dependence among older adults age 65 years and older.
27. Matsushita, K., Ding, N., Ballew, S. H., Palta, P., Schrack, J. A., Windham, B. G., & Coresh, J. (2017, March). Muscle strength and Short-term Risk of Cardiovascular Outcomes in Community-dwelling Older Adults: The Atherosclerosis Risk in Communities (ARIC) Study. In *Circulation* (Vol. 135). Two Commerce Sq, 2001 Market St, Philadelphia, Pa 19103 Usa: Lippincott Williams & Wilkins.
28. Moretti, A., De Sire, A., Giamattei, M., Gimigliano, F., & Iolascon, G. (2015). Dynapenic skeletal muscle function deficit as determinant of skeletal fragility: a retrospective analysis. In *WCO-IOF-ESCEO World Congress on Osteoporosis, Osteoarthritis and Musculoskeletal Diseases*.
29. Muraki S, Akune T, Oka H, et al. (2010, December). Gender differences in factors associated with falls in a population-based cohort study in Japan: The ROAR study. . In *osteoporosis international* (vol. 21, pp. S671-s672). 236 grays inn rd, 6th floor, London wc1x 8hl, England: Springer London ltd.
30. Muraki, S., Akune, T., Oka, H., Nakamura, K., Kawaguchi, H., & Yoshimura, N. (2010, December). Gender differences in incidence of falls and its associated factors in a population-based cohort study in Japan: the ROAR study. In *osteoporosis international* (vol. 21, pp. S671-s672). 236 grays inn rd, 6th floor, London wc1x 8hl, England: Springer London ltd.
31. Nordström, P., Sievänen, H., Gustafson, Y., Pedersen, N., & Nordström, A. (2012). High physical capacity in young adulthood reduces the risk of fracture later in life. *Bone*, (50), S55-S56.
32. Park, D. J., Wen, L., Kang, J. H., Yim, Y. R., Kim, J. E., Lee, J. W., ... & Lee, S. S. (2016, October). Association Between Grip Strength and Hand and Knee Radiographic Osteoarthritis in Older Adults: Data from the Dong-Gu Study. In *ARTHRITIS & RHEUMATOLOGY* (Vol. 68). 111 RIVER ST, HOBOKEN 07030-5774, NJ USA: WILEY.
33. Parsons, C., Edwards, M. H., Cooper, C., Dennison, E. M., & Ward, K. A. (2017, July). Does physical performance testing predict falls? Results from the hertfordshire cohort study. In *osteoporosis international* (vol. 28, pp. S488-s489). 236 grays inn rd, 6th floor, London wc1x 8hl, England: springer London ltd.
34. Pietarila-Heikkinen, T., Korpelainen, R., Pietarila-Heikkinen, T., Keinanen-Kiukaanniemi, S., Nieminen, P., Heikkinen, J., ... & Korpelainen, J. (2010, December). Long-term effect of exercise on extraskeletal risk factors for fractures in elderly high-risk women-follow-up of a population-based randomised controlled trial. In *OSTEOPOROSIS INTERNATIONAL* (Vol. 21, Pp. S712-S712). 236 GRAYS INN RD, 6TH FLOOR, LONDON WC1X 8HL, ENGLAND: SPRINGER LONDON LTD.
35. Rosengren, B., Nilsson, J. Å., Ohlsson, C., Mellstrom, D., Lorentzon, M., Ljunggren, O., ... & Karlsson, M. (2010). Fall in elderly men can be predicted by physical ability tests-the mr os international study. In *iof World Congress on Osteoporosis/10th European Congress on Clinical and Economic Aspects of Osteoporosis and Osteoarthritis* (Vol. 21, No. Suppl. 1, pp. 272-273). Springer.
36. Safonova, J. A. (2016). Assessment methods risk of falls in people over age 65. *Advances in Gerontology= Uspekhi Gerontologii*, *29*(3), 517-522.
37. Scott, D., Sanders, K., Laslett, L., Hayes, A., Ebeling, P., & Jones, G. (2022). Sarcopenia and health-related quality of life over five years in community-dwelling older adults.
38. Shalnova, S. (2011). Association between cognitive function and grip strength in Muscovites 55 year and older. *The SAHR study*.
39. Tomson, C., Al Snih, S., & Sodhi, J. (2020, April). Effects of Muscle Strength and Physical Function on Hospitalization Risk in Older Mexican Americans. In *Journal of The American Geriatrics Society* (Vol. 68, Pp. S293-S293). 111 River St, Hoboken 07030-5774, Nj Usa: Wiley.
40. Wai-Keong H, Tsai-Chun C, Ming-Hisa H. (2012). Performance measures as predictors of frailty in older people. *Neurorehabilitation and Neural Repair.* *26*(6):800.
41. Wang, M., Wu, F., Callisaya, M., Jones, G., & Winzenberg, T. (2020, December). Baseline and change in leg muscle strength and balance are not associated with the incidence of falls in middle-aged women: a 5-year population-based prospective study. In *osteoporosis international* (vol. 31, no. Suppl 1, pp. S521-s521). 236 grays inn rd, 6th floor, London wc1x 8hl, England: Springer London ltd.
42. Ward, K., Muthuri, S., Moore, A., Adams, J., Cooper, C., Kuh, D., & Cooper, R. (2017, December). Changes in muscle strength and physical performance from midlife and bone health in early old-age: a 7-year follow up study of the MRC National Survey of Health and Development. In *journal of bone and mineral research* (Vol. 32, pp. S218-S218). 111 river st, hoboken 07030-5774, nj usa: wiley.
43. Westbury, L. D., Syddall, H. E., Fuggle, N. R., Dennison, E. M., Cauley, J. A., Shiroma, E. J., ... & Cooper, C. (2020, December). Level and change in sarcopenia components predict adverse health outcomes: findings from the health, aging and body composition study. In *Osteoporosis International* (Vol. 31, No. Suppl 1, Pp. S48-S48). 236 Grays Inn Rd, 6th Floor, London Wc1x 8hl, England: Springer London Ltd.

- **Wrong comparator (n=54)**

1. Aoyama, M., Suzuki, Y., Onishi, J., & Kuzuya, M. (2011). Physical and functional factors in activities of daily living that predict falls in community‐dwelling older women. *Geriatrics & gerontology international*, *11*(3), 348-357.
2. Artero, E. G., Lee, D. C., Lavie, C. J., España-Romero, V., Sui, X., Church, T. S., & Blair, S. N. (2012). Effects of muscular strength on cardiovascular risk factors and prognosis. *Journal of cardiopulmonary rehabilitation and prevention*, *32*(6), 351-358.
3. Arvandi, M., Strasser, B., Volaklis, K., Ladwig, K. H., Grill, E., Matteucci Gothe, R., ... & Meisinger, C. (2018). Mediator effect of balance problems on association between grip strength and falls in older adults: results from the KORA-age study. *Gerontology and geriatric medicine*, *4*, 2333721418760122.
4. Bindawas S, Kuo YF, Al Snih S, Protas E, Ottenbacher KJ. (2011). The effects of lower extremity performance on health-related quality of in older Mexican-Americans. *Physiotherapy*, *97*:eS131.
5. Bird, M. L., Pittaway, J. K., Cuisick, I., Rattray, M., & Ahuja, K. D. (2013). Age-related changes in physical fall risk factors: results from a 3 year follow-up of community dwelling older adults in Tasmania, Australia. *International journal of environmental research and public health*, *10*(11), 5989-5997.
6. Bleicher, K., Cumming, R. G., Naganathan, V., Seibel, M. J., Blyth, F. M., Le Couteur, D. G., ... & Waite, L. M. (2013). Predictors of the rate of BMD loss in older men: findings from the CHAMP study. *Osteoporosis International*, *24*, 1951-1963.
7. Brooks, J. M., Titus, A. J., Bruce, M. L., Orzechowski, N. M., Mackenzie, T. A., Bartels, S. J., & Batsis, J. A. (2018). Depression and Handgrip Strength Among US Adults Aged 60 Years and Older from NHANES 2011-2014. *The Journal of nutrition, health and aging*, *22*(8), 938-943.
8. Buchman, A. S., Boyle, P. A., Leurgans, S. E., Evans, D. A., & Bennett, D. A. (2009). Pulmonary function, muscle strength, and incident mobility disability in elders. *Proceedings of the American Thoracic Society*, *6*(7), 581-587.
9. Buckinx, F., Croisier, J. L., Reginster, J. Y., Lenaerts, C., Brunois, T., Rygaert, X., ... & Bruyere, O. (2018). Prediction of the incidence of falls and deaths among elderly nursing home residents: the SENIOR study. *Journal of the American Medical Directors Association*, *19*(1), 18-24.
10. Bullain, S. S., Corrada, M. M., Perry, S. M., & Kawas, C. H. (2016). Sound body sound mind? Physical performance and the risk of dementia in the oldest‐old: the 90+ study. *Journal of the American Geriatrics Society*, *64*(7), 1408-1415.
11. Cardon-Verbecq, C., Loustau, M., Guitard, E., Bonduelle, M., Delahaye, E., Koskas, P., & Raynaud-Simon, A. (2017). Predicting falls with the cognitive timed up-and-go dual task in frail older patients. *Annals of physical and rehabilitation medicine*, *60*(2), 83-86.
12. Chen, Y., Liu, Y., Han, P., Zhang, H., Mei, Y., Wang, Y., ... & Wang, H. (2022). Mobility speed predicts new-onset hypertension: a longitudinal study. *Blood Pressure Monitoring*, *27*(1), 22-26.
13. Delvaux, K. A. T. R. I. E. N., Lefevre, J. O. H. A. N., Philippaerts, R. E. N. A. A. T., Dequeker, J., Thomis, M. A. R. T. I. N. E., Vanreusel, B. A. R. T., ... & Lysens, R. O. E. L. A. N. D. (2001). Bone mass and lifetime physical activity in Flemish males: a 27-year follow-up study. *Medicine and science in sports and exercise*, *33*(11), 1868-1875.
14. den Ouden, M. E., Schuurmans, M. J., Mueller-Schotte, S., & Van der Schouw, Y. T. (2013). Identification of high-risk individuals for the development of disability in activities of daily living. A ten-year follow-up study. *Experimental gerontology*, *48*(4), 437-443.
15. Deshpande, N., Metter, J. E., Guralnik, J., Bandinelli, S., & Ferrucci, L. (2014). Sensorimotor and psychosocial determinants of 3-year incident mobility disability in middle-aged and older adults. *Age and ageing*, *43*(1), 64-69.
16. Doi, T., Makizako, H., Tsutsumimoto, K., Nakakubo, S., Kim, M. J., Kurita, S., ... & Shimada, H. (2018). Transitional status and modifiable risk of frailty in Japanese older adults: A prospective cohort study. *Geriatrics & gerontology international*, *18*(11), 1562-1566.
17. Ericsson, Y. B., McGuigan, F. E., & Akesson, K. E. (2021). Knee pain in young adult women-associations with muscle strength, body composition and physical activity. *BMC Musculoskeletal Disorders*, *22*(1), 715.
18. Foldvari, M., Clark, M., Laviolette, L. C., Bernstein, M. A., Kaliton, D., Castaneda, C., ... & Singh, M. A. F. (2000). Association of muscle power with functional status in community-dwelling elderly women. *The Journals of Gerontology Series A: Biological Sciences and Medical Sciences*, *55*(4), M192-M199.
19. Graafmans, W. C., Ooms, M. E., Hofstee, H. M. A., Bezemer, P. D., Bouter, L. M., & Lips, P. T. A. M. (1996). Falls in the elderly: a prospective study of risk factors and risk profiles. *American journal of epidemiology*, *143*(11), 1129-1136.
20. Hatayama, T., Nagano, M., Une, H., Yoshitake, Y., Kimura, Y., Momose, Y., ... & Kumagai, S. (2008). The association between incidence of falls and physical fitness in community-dwelling elderly. *Japanese Journal of Physical Fitness and Sports Medicine*, 503-510.
21. Heidari, B., Muhammadi, A., Javadian, Y., Bijani, A., Hosseini, R., & Babaei, M. (2016). Associated factors of bone mineral density and osteoporosis in elderly males. *International Journal of Endocrinology and Metabolism*, *15*(1), e39662.
22. Hootman, J. M., FitzGerald, S., Macera, C. A., & Blair, S. N. (2004). Lower extremity muscle strength and risk of self-reported hip or knee osteoarthritis. *Journal of Physical Activity and Health*, *1*(4), 321-330.
23. Hori Y, Hoshino M, Takahashi S, et al. (2018). Related factors of neck disability index (NDI) among elderly people in suburban area: Shiraniwa cohort study. *European Spine Journal, 27*(5):S671-S672.
24. Huang, C., Sun, S., Tian, X., Wang, T., Wang, T., Duan, H., & Wu, Y. (2019). Age modify the associations of obesity, physical activity, vision and grip strength with functional mobility in Irish aged 50 and older. *Archives of gerontology and geriatrics*, *84*, 103895.
25. Imagama, S., Matsuyama, Y., Hasegawa, Y., Sakai, Y., Ito, Z., Ishiguro, N., & Hamajima, N. (2011). Back muscle strength and spinal mobility are predictors of quality of life in middle-aged and elderly males. *European Spine Journal*, *20*, 954-961.
26. Jackson, A. W., Morrow Jr, J. R., Brill, P. A., Kohl III, H. W., Gordon, N. F., & Blair, S. N. (1998). Relations of sit-up and sit-and-reach tests to low back pain in adults. *Journal of Orthopaedic & Sports Physical Therapy*, *27*(1), 22-26.
27. Lin, C., Wu, X., Liu, Q., Cheng, X., Chen, Y., Ke, Y., & Lin, J. (2018). Incidence and related risk factors of radiographic knee osteoarthritis in a Chinese suburban area: Shunyi osteoarthritis study. *Osteoarthritis and Cartilage*, *26*, S218-S219.
28. Lord, S. R., & Clark, R. D. (1996). Simple physiological and clinical tests for the accurate prediction of falling in older people. *Gerontology*, *42*(4), 199-203.
29. Magnusson, K., Turkiewicz, A., Timpka, S., & Englund, M. (2018). A prediction model for hand osteoarthritis in middle-aged men–data from the Swedish conscription register. *Osteoarthritis and Cartilage*, *26*, S212.
30. Mengist, B., Lotfaliany, M., Pasco, J. A., Agustini, B., Berk, M., Williams, L. J., ... & Mohebbi, M. (2025). Gait speed, handgrip strength, and their combination, and risk of depression in later life: Evidence from a prospective study of community-dwelling older adults. *Journal of Affective Disorders*, *369*, 218-226.
31. Michel, E., Zory, R., Guerin, O., Prate, F., Sacco, G., & Chorin, F. (2024). Assessing muscle quality as a key predictor to differentiate fallers from non-fallers in older adults. *European Geriatric Medicine*, 1-11.
32. Oh, B., Cho, B., Choi, H. C., Son, K. Y., Park, S. M., Chun, S., & Cho, S. I. (2014). The influence of lower-extremity function in elderly individuals’ quality of life (QOL): an analysis of the correlation between SPPB and EQ-5D. *Archives of gerontology and geriatrics*, *58*(2), 278-282.
33. Pham, H. M., Nguyen, N. D., Center, J. R., Eisman, J. A., & Nguyen, T. V. (2016). Contribution of quadriceps weakness to fragility fracture: a prospective study. *Journal of Bone and Mineral Research*, *31*(1), 208-214.
34. Piirtola, M., Vahlberg, T., Isoaho, R., Aarnio, P., & Kivelä, S. L. (2008). Predictors of fractures among the aged: a population-based study with 12-year follow-up in a Finnish municipality. *Aging clinical and experimental research*, *20*, 242-252.
35. Prieto‐Alhambra, D., Turkiewicz, A., Reyes, C., Timpka, S., Rosengren, B., & Englund, M. (2020). Smoking and alcohol intake but not muscle strength in young men increase fracture risk at middle age: a cohort study linked to the Swedish national patient registry. *Journal of Bone and Mineral Research*, *35*(3), 498-504.
36. Qiu, Y., Li, Z., Lin, C., Liu, Q., Lin, J., & Zhang, Y. (2019). Incidence, related risk factors, imaging characteristics analysis and prediction model of radiographic patellofemoral osteoarthritis in a Chinese suburban area: Shunyi osteoarthritis study. *Osteoarthritis and Cartilage*, *27*, S246.
37. Rantanen, T., Avlund, K., Suominen, H., Schroll, M., Frändin, K., & Pertti, E. (2002). Muscle strength as a predictor of onset of ADL dependence in people aged 75 years. *Aging clinical and experimental research*, *14*(3 Suppl), 10-15.
38. Sayer, A. A., Dennison, E. M., Syddall, H. E., Gilbody, H. J., Phillips, D. I., & Cooper, C. (2005). Type 2 Diabetes, Muscle Strength, and Impaired Physical Function. *Diabetes care*, *28*(10).
39. Scronce, G., Zhang, W., Smith, M. L., & Mercer, V. S. (2020). Characteristics associated with improved physical performance among community-dwelling older adults in a community-based falls prevention program. *International journal of environmental research and public health*, *17*(7), 2509.
40. Segal, N. A., & Glass, N. A. (2011). Is quadriceps muscle weakness a risk factor for incident or progressive knee osteoarthritis? *The Physician and Sportsmedicine*, *39*(4), 44-50.
41. Seko, T., Akasaka, H., Koyama, M., Himuro, N., Saitoh, S., Miura, T., ... & Ohnishi, H. (2023). Preserved lower limb muscle mass prevents insulin resistance development in nondiabetic older adults. *Journal of the American Medical Directors Association*, *24*(3), 376-381.
42. Shafiee, G., Ostovar, A., Heshmat, R., Darabi, H., Sharifi, F., Raeisi, A., ... & Larijani, B. (2017). Bushehr Elderly Health (BEH) programme: study protocol and design of musculoskeletal system and cognitive function (stage II). *BMJ open*, *7*(8), e013606.
43. Stenholm, S., Alley, D., Bandinelli, S., Griswold, M. E., Koskinen, S., Rantanen, T., ... & Ferrucci, L. (2009). The effect of obesity combined with low muscle strength on decline in mobility in older persons: results from the InCHIANTI study. *International journal of obesity*, *33*(6), 635-644.
44. Tainaka, K., Takizawa, T., Katamoto, S., & Aoki, J. (2009). Six‐year prospective study of physical fitness and incidence of disability among community‐dwelling Japanese elderly women. *Geriatrics & gerontology international*, *9*(1), 21-28.
45. Takagi, S., Omori, G., Koga, H., Endo, K., Koga, Y., Nawata, A., & Endo, N. (2018). Quadriceps muscle weakness is related to increased risk of radiographic knee OA but not its progression in both women and men: the Matsudai Knee Osteoarthritis Survey. *Knee Surgery, Sports Traumatology, Arthroscopy*, *26*, 2607-2614.
46. Tembo, M. C., Mohebbi, M., Holloway-Kew, K. L., Gaston, J., Brennan-Olsen, S. L., Williams, L. J., ... & Pasco, J. A. (2021). The predictability of frailty associated with musculoskeletal deficits: a longitudinal study. *Calcified Tissue International*, *109*(5), 525-533.
47. Van Dam, R., Van Ancum, J. M., Verlaan, S., Scheerman, K., Meskers, C. G., & Maier, A. B. (2018). Lower cognitive function in older patients with lower muscle strength and muscle mass. *Dementia and geriatric cognitive disorders*, *45*(3-4), 243-250.
48. Veronese, N., Stefanac, S., Koyanagi, A., Al-Daghri, N. M., Sabico, S., Cooper, C., ... & Maggi, S. (2021). Lower limb muscle strength and muscle mass are associated with incident symptomatic knee osteoarthritis: A longitudinal cohort study. *Frontiers in Endocrinology*, *12*, 804560.
49. Villa, M. L., Marcus, R., Delay, R. R., & Kelsey, J. L. (1995). Factors contributing to skeletal health of postmenopausal Mexican‐American women. *Journal of Bone and Mineral Research*, *10*(8), 1233-1242.
50. Wang M, Wu F, Callisaya M, Jones G, Winzenberg TM. (2021). Neither Baseline Nor Change in Leg Muscle Strength and Balance Are Associated with the Incidence of Falls in Middle-aged Women: A 5-year Population-based Prospective Study. *Journal of Bone and Mineral Research*, *35*(1):318.
51. Wang, X., Wu, L., Zhou, H., & He, J. (2024). Grip strength and depressive symptoms in Chinese middle-aged and older adults: the mediating effects of cognitive function. *Frontiers in Aging Neuroscience*, *16*, 1455546.
52. Wang, Y., Huang, Y., & Chen, X. (2023). The relationship between low handgrip strength with or without asymmetry and fall risk among middle-aged and older males in China: evidence from the China Health and Retirement Longitudinal Study. *Postgraduate medical journal*, *99*(1178), 1246-1252.
53. Wilson, P. W., Meigs, J. B., Sullivan, L., Fox, C. S., Nathan, D. M., & D’Agostino, R. B. (2007). Prediction of incident diabetes mellitus in middle-aged adults: the Framingham Offspring Study. *Archives of internal medicine*, *167*(10), 1068-1074.
54. Zhang, L., Liu, S., Li, Y., Li, S., & Wu, Y. (2022). Associations of sleep quality with gait speed and falls in older adults: The mediating effect of muscle strength and the gender difference. *Gerontology*, *68*(1), 1-7.

- **Wrong population (n=2)**

1. Åberg, N. D., Kuhn, H. G., Nyberg, J., Waern, M., Friberg, P., Svensson, J., ... & Nilsson, M. (2015). Influence of cardiovascular fitness and muscle strength in early adulthood on long-term risk of stroke in Swedish men. *Stroke*, *46*(7), 1769-1776.
2. Grøntved, A., Ried-Larsen, M., Ekelund, U., Froberg, K., Brage, S., & Andersen, L. B. (2013). Independent and combined association of muscle strength and cardiorespiratory fitness in youth with insulin resistance and β-cell function in young adulthood: the European Youth Heart Study. *Diabetes Care*, *36*(9), 2575-2581.

**Meta-analysis**

**Highest (vs. lowest) category of handgrip strength test**

Fifty-eight comparisons between the highest (vs. lowest) category of HGS test and long-term health conditions from 64 cohort studies (mean age range: 50.0–84.7 years) were included in the meta-analysis (**figure 2**). The studies for each long-term health condition along with their applied cut-off points for defining the highest and lowest levels of HGS test are displayed in **online supplemental appendix 3 (figure S13)**. All included studies provided data for HGS test categories by standardizing the cut-off point for high and low levels according to the participant’s sex or adjusting for this variable. The pooled ORs comparing prospective associations between the highest (vs. lowest) category of HGS test and the risk of long-term health conditions were (i) 0.73 (95% CI:0.67–0.80; I^2^=50.2%; n=15) for cardiovascular diseases (n=568,975; incident cases: 44,496); (ii) 0.79 (95% CI:0.68–0.91; I^2^=46.3%; n=5) for T2DM (n=248,804; incident cases: 10,336); (iii) 0.65 (95% CI:0.56–0.76; I^2^=51.6%; n=10) for musculoskeletal impairment (n=68,777; incident cases: 3,197); (iv) 0.57 (95% CI:0.47–0.70; I^2^=42.6%; n=13) for disability (n=88,458; incident cases: 15,406 [no data available for all studies]); (v) 0.79 (95% CI:0.63–0.99; I^2^=0%; n=3) for anxiety (n=167,438; incident cases: 6,742); (vi) 0.70 (95% CI:0.63–0.78; I^2^=52.1%; n=9) for depression (n=453,401; incident cases: 18,079 [no data available for all studies]); (vii) 0.57 (95% CI:0.44–0.75; I^2^=73.5%; n=8) for cognitive decline (n=25,691; incident cases: 1,570 [no data available for all studies]); (viii) 0.62 (95% CI:0.53–0.73; I^2^=0%; n=5) for dementia (n=251,626; incident cases: 2,905); and (ix) 0.53 (95% CI:0.31–0.91; I^2^=89.0%; n=3) for Parkinson's disease (n=358,791; incident cases: 1,810). Pooled ORs for the risk of both cancer and respiratory diseases were not performed because only one study examined the prospective role of HGS test levels.

**Per 5-kg increase in handgrip strength test**

Forty-four comparisons between a 5-kg increase in HGS test and long-term health conditions from 41 cohort studies (mean age range: 37.2–85.0 years) were included in the meta-analysis (**figure 2**). The studies within each long-term health conditions are detailed in **online supplemental appendix 3 (figure S14)**. The pooled ORs for the prospective associations between a 5-kg increase in HGS test and the incidence of long-term health conditions were (i) 0.93 (95% CI:0.90–0.96; I^2^=58.2%; n=8) for cardiovascular diseases (n=685,229; incident cases: 45,976); (ii) 1.03 (95% CI:0.96–1.10; I^2^=47.6%; n=3) for cancer (n=580,162; incident cases: 30,985); (iii) 0.95 (95% CI:0.90–1.00; I^2^=27.8%; n=8) for T2DM (n=316,875; incident cases: 8,289); (iv) 0.91 (95% CI:0.78–1.07; I^2^=97.0%; n=2) for respiratory diseases (n=593,329; incident cases: 12,700); (v) 0.92 (95% CI:0.85–1.00; I^2^=94.2%; n=6) for musculoskeletal impairment (n=174,583; incident cases: 9,912); (vi) 0.79 (95% CI:0.67–0.95; I^2^=75.9%; n=6) for disability (n=4,549; incident cases: 1,145 [no data available for all studies]); (vii) 0.94 (95% CI:0.90–0.98; I^2^=85.5%; n=5) for depression (n=193,009; incident cases: 7,695 [no data available for all studies]); (viii) 0.93 (95% CI:0.87–0.99; I^2^=1.2%; n=5) for cognitive decline (n=16,355; incident cases: 593 [no data available for all studies]); (ix) 0.87 (95% CI:0.84–0.91; I^2^=0%; n=5) for dementia (n=469,524; incident cases: 4,253); and (x) 0.86 (95% CI:0.79–0.95; I^2^=63.2%; n=3) for Parkinson's disease (n=494,233; incident cases: 2,432). No study examined the association between a 5-kg increase in HGS test and the risk of anxiety.

**Chair-stand test performance**

The results of the meta-analysis according to 5-CST are shown in **figure 3.** Sixteen cohort studies comparing the best (vs. worst) category of 5-CST performance and long-term health conditions (mean age range: 57.6–84.2 years) were included in the meta-analysis. The studies within each health long-term health condition along with their applied cut-off points for defining the best and worst levels of 5-CTS are displayed in **online supplemental appendix 3 (figure S15)**. The studies applied different cut-off points for defining the best (i.e., ranging from <6.8 to <17.0 seconds) and worst (i.e., ranging from ≥11.2 to ≥17.0 seconds) levels of 5-CST. The pooled ORs comparing prospective associations between the best (vs. worst) category of 5-CST performance and the risk of long-term health conditions were (i) 0.80 (95% CI:0.72–0.88; I^2^=0%; n=2) for T2DM (n=23,578; incident cases: 1,866); (ii) 0.52 (95% CI:0.37–0.74; I^2^=0%; n=6) for musculoskeletal impairment (n=16,172; incident cases: 2,325); (iii) 0.58 (95% CI:0.41–0.82; I^2^=44.1%; n=5) for disability (n=5,561; incident cases: 2112); (iv) 0.63 (95% CI:0.42–0.95; I^2^=72.0%; n=2) for depression (n=35,78; incident cases: 983); and (v) 0.68 (95% CI:0.54–0.85; I^2^=0%; n=2) for dementia (n=7,753; incident cases: 569). The prospective association between 5-CST levels and cognitive decline was only analysed in a single study. When comparing 5-CST performance as continuous exposure, a lower risk of musculoskeletal impairment was observed per 1-second decrease (OR: 0.94, 95% CI: 0.89–1.00; I^2^=92.1%) in 4 cohort studies (n=14,068; incident cases: 1,986; mean age range: 63.8–73.5 years) (**figure 3** and **figure S16 in online supplemental appendix 3**).

**Subgroup analyses**

Subgroup analyses according to sex are shown in **table 3.** When comparing the highest (vs. lowest) HGS test in at least two cohort studies, the pooled ORs remained significant in reducing the risk of most long-term health conditions in both females and males. When comparing HGS test as continuous exposure, the inverse association between a 5-kg increase and a lower risk of musculoskeletal impairment, depression and Parkinson’s disease remained significant for both females and males. In turn, a 5-kg increase was non-significantly and significantly associated with a lower risk of T2DM in females (OR: 0.97, 95% CI: 0.87–1.08; I^2^=56.8%; n=3) and males (OR: 0.92, 95% CI: 0.87–0.97; I^2^=7.8%; n=4), respectively. Moreover, a 5-kg increase was associated with a non-significant reduction in the risk of cardiovascular diseases and cognitive decline for both females and males.

Subgroup analyses according to adult age group are displayed in **table 4.** When comparing the highest (vs. lowest) HGS test in at least two studies, the pooled ORs remained significant in reducing the risk of most long-term health conditions for which data were reported for both middle-aged and older adults. Conversely, while a 5-kg increase in HGS test was associated as a protective factor for the risk of developing cardiovascular diseases and dementia in both middle-aged and older adults, the estimates from the meta-analyses of other long-term health conditions yielded mixed results. Specifically, significant protective associations for depression were observed among middle-aged adults (OR: 0.92, 95% CI: 0.90–0.95; I²=17.7%; n=4) compared to older adults (OR: 0.94, 95% CI: 0.82–1.08; I²=95.2%; n=2). In contrast, significant associations for musculoskeletal impairment (OR: 0.95, 95% CI: 0.91–0.98; I²=63.8%; n=3 for older adults vs. OR: 0.92, 95% CI: 0.80–1.04; I²=94.3%; n=4 for middle-aged) and disability (OR: 0.79, 95% CI: 0.66–0.96; I²=76.6%; n=4 for older adults vs. OR: 0.76, 95% CI: 0.46–1.26; n=2 for middle-aged) were found in older populations.

Subgroup analyses according to the geographical region are detailed in **table S6** (**online supplemental appendix 2**). When comparing the highest (vs. lowest) HGS test in at least two studies, pooled ORs remained significant in reducing the risk of cardiovascular diseases in Asia, Europe, and North America. In turn, cognitive decline was examined only in Asia and North America and T2DM and depression only in Asia and Europe, with a significant protective association for the respective regions. Instead, the highest (vs. lowest) HGS test was significantly associated with a lower risk of disability in Asia (OR: 0.57, 95% CI: 0.40–0.80; I^2^=63.8%; n=5) and North America (OR: 0.52, 95% CI: 0.43–0.63; I^2^=0%; n=2), and non-significantly associated in Europe (OR: 0.61, 95% CI: 0.36–1.01; I^2^=50.7%; n=5), respectively. When comparing HGS as continuous exposure, while a significant protective association was found per 5-kg increase and cardiovascular diseases for Asia (OR: 0.92, 95% CI: 0.88–0.96; I^2^=0%; n=2) and North America (OR: 0.95, 95% CI: 0.91–1.00; I^2^=0%; n=2), the decrease in the incidence was not significant for Europe (OR: 0.92, 95% CI: 0.83–1.02; I^2^=89.6%; n=4). In turn, a 5-kg increase was significantly and non-significantly associated with a lower risk of T2DM in Europe (OR: 0.93, 95% CI: 0.86–0.99; I^2^=58.0%; n=2) and North America (OR: 0.91, 95% CI: 0.78–1.06; I^2^=21.0%; n=3), respectively. Finally, while a 5-kg increase was significantly associated with a lower risk of disability in both Asia (OR: 0.59, 95% CI: 0.48–0.73; I^2^=0%; n=2) and North America (OR: 0.81, 95% CI: 0.74–0.89; I^2^=0%; n=2), the reduction in the incidence was not significant for the European population (OR: 0.96, 95% CI: 0.88–1.06; I^2^=0%; n=2).

**Sensitivity analyses**

The pooled ORs for cardiovascular diseases, cancer, T2DM, respiratory diseases, depression, and Parkinson’s disease were not modified by removing studies that analyzed the increased or decreased HGS test with values other than 5-kg (**table S8 in online supplemental appendix 2**). Furthermore, participants without muscular weakness (i.e., ≥31 kg for males and ≥21 kg for females) were associated with a lower risk of cardiovascular diseases (OR: 0.77, 95% CI: 0.69–0.86; I^2^=39.0%; n=6), musculoskeletal impairment (OR: 0.68, 95% CI: 0.54–0.87; I^2^=11.2%; n=4), disability (OR: 0.59, 95% CI: 0.41–0.86; I^2^=46.9%; n=6), anxiety (OR: 0.79, 95% CI: 0.63–0.99; I^2^=0%; n=3), depression (OR: 0.78, 95% CI: 0.69–0.89; I^2^=43.3%; n=5), and cognitive decline (OR: 0.52, 95% CI: 0.30–0.91; I^2^=80.0%; n=3) than participants reporting muscular weakness (**table S8 in online supplemental appendix 2)**.

The leave-one-out method showed that the pooled ORs remained significant upon removal of individual studies in most sensitivity analyses (i.e., 13 out of 17), indicating the robustness of our results, except for T2DM (in the continuous HGS test analysis), musculoskeletal impairment (in both the continuous HGS test and 5-CST analyses) and cognitive decline (in the continuous HGS test analysis) (**figures S17-S33 in online supplemental appendix 3**). This suggests that the overall findings for these long-term health conditions may be sensitive to individual study effects and should therefore be interpreted with caution. Additional high-quality studies may help to strengthen the evidence base and confirm these associations. The leave-one-out analysis also revealed that heterogeneity was substantially modified when certain studies were removed (**figures S17-S33 in online supplemental appendix 3**). This indicates that specific studies may be major contributors to between-study variability, possibly due to methodological differences or population characteristics.

**Other data synthesis**

The results from relative HGS on long-term health conditions are displayed in **table S9** (**online supplemental appendix 2**). Although two or more studies analyzed the prospective associations between relative HGS test and specific long-term health conditions (i.e., cardiovascular diseases, T2DM, dementia, and Parkinson’s disease),^1-27^ meta-analytical approaches were not performed due to heterogeneity of HGS test representations (i.e., kg divided by body mass index, body weight, arm lean mass, or total lean mass) and exposure harmonizations. In summary, the highest (vs. lowest) level of relative HGS test was associated with a significantly reduced incidence of cardiovascular diseases (n=6), T2DM (n=12) and Parkinson’s disease (n=2) in most cohort studies. This protective role does not differ by changing the way in which the relative HGS test is expressed. In turn, relative HGS as a continuous variable was associated with a significantly lower risk of cardiovascular diseases (n=5) and T2DM (n=8) in all included cohort studies across the different proxies of HGS test (i.e., kg or kPa by body mass index, body weight, arm lean mass, or total lean mass).

**REFERENCES**

1. Jang S-k, Kim J-h, Lee Y. Effect of relative handgrip strength on cardiovascular disease among Korean adults aged 45 years and older: results from the Korean Longitudinal Study of Aging (2006–2016). *Archives of Gerontology and Geriatrics* 2020;86:103937.

2. Kim Y, Hwang S, Sharp SJ, et al. Genetic Risk, Muscle Strength, and Incident Stroke: Findings From the UK Biobank Study. *MAYO CLINIC PROCEEDINGS* 2021;96(7):1746-57. doi: 10.1016/j.mayocp.2021.01.034

3. Boonpor J, Parra-Soto S, Petermann-Rocha F, et al. Associations between grip strength and incident type 2 diabetes: findings from the UK Biobank prospective cohort study. *BMJ Open Diabetes Research and Care* 2021;9(1):e001865.

4. Hao G, Chen H, Ying Y, et al. The Relative Handgrip Strength and Risk of Cardiometabolic Disorders: A Prospective Study. *Frontiers in Physiology* 2020;11((Hao, Ying, Wu, Jing) Department of Epidemiology, School of Medicine, Jinan University, Guangzhou, China(Chen) Department of Endemic Disease, Guangzhou Center for Disease Control and Prevention, Guangzhou, China(Yang) Department of Pathogen Biology, Schoo):719. doi: 10.3389/fphys.2020.00719

5. Jeon Y-J, Lee SK, Shin C. Normalized hand grip and back muscle strength as risk factors for incident type 2 diabetes mellitus: 16 years of follow-up in a population-based cohort study. *Diabetes, Metabolic Syndrome and Obesity* 2021:741-50.

6. Karvonen-Gutierrez CA, Peng Q, Peterson M, et al. Low grip strength predicts incident diabetes among mid-life women: the Michigan Study of Women’s Health Across the Nation. *Age and ageing* 2018;47(5):685-91.

7. Li G, Qiao Y, Lu Y, et al. Role of handgrip strength in predicting new-onset diabetes: findings from the survey of health, ageing and retirement in Europe. *BMC geriatrics* 2021;21(1):445-45. doi: 10.1186/s12877-021-02382-9

8. Li JJ, Wittert GA, Vincent A, et al. Muscle grip strength predicts incident type 2 diabetes: Population-based cohort study. *Metabolism: clinical and experimental* 2016;65(6):883-92. doi: 10.1016/j.metabol.2016.03.011

9. Qiu S, Cai X, Liang Y, et al. Cumulative muscle strength and risk of diabetes: A prospective cohort study with mediation analysis. *Diabetes Research and Clinical Practice* 2023;197:110562.

10. Manda CM, Hokimoto T, Okura T, et al. Handgrip strength predicts new prediabetes cases among adults: A prospective cohort study. *Preventive medicine reports* 2020;17:101056.

11. Momma H, Sawada SS, Kato K, et al. Physical fitness tests and type 2 diabetes among Japanese: a longitudinal study from the Niigata Wellness Study. *Journal of epidemiology* 2019;29(4):139-46.

12. McGrath R, Vincent BM, Al Snih S, et al. The association between muscle weakness and incident diabetes in older Mexican Americans. *Journal of the American Medical Directors Association* 2017;18(5):452. e7-52. e12.

13. Peterson MD, Casten K, Collins S, et al. Muscle weakness is a prognostic indicator of disability and chronic disease multimorbidity. *Experimental gerontology* 2021;152:111462.

14. Zheng J, Zhang L, Jiang M. Lower handgrip strength levels probably precede triglyceride glucose index and associated with diabetes in men not in women. *Journal of Diabetes Investigation* 2022;13(1):148-55.

15. Kunutsor SK, Voutilainen A, Laukkanen JA. Handgrip strength improves prediction of type 2 diabetes: a prospective cohort study. *ANNALS OF MEDICINE* 2020;52(8):471-78. doi: 10.1080/07853890.2020.1815078

16. Li G, Lu Y, Shao L, et al. Handgrip strength is associated with risks of new-onset stroke and heart disease: results from 3 prospective cohorts. *BMC geriatrics* 2023;23(1):268. doi: 10.1186/s12877-023-03953-8

17. Liu DQ, Yang CX, Liu G, et al. Association between grip strength, walking pace and incident peripheral artery disease: A prospective study of 430,886 UK biobank participants. *International Journal of Cardiology Cardiovascular Risk and Prevention* 2024;23 doi: 10.1016/j.ijcrp.2024.200330

18. Liu MY, He PP, Ye ZL, et al. Association of handgrip strength and walking pace with incident Parkinson's disease. *Journal of Cachexia Sarcopenia and Muscle* 2024;15(1):198-207. doi: 10.1002/jcsm.13366

19. Qi K-j, Li Q, Lu G-l, et al. The combined effect of handgrip strength and obesity phenotype on the risk of stroke in Chinese middle-aged and elderly: A cohort study. *Archives of Gerontology & Geriatrics* 2024;124:N.PAG-N.PAG. doi: 10.1016/j.archger.2024.105481

20. Zhang F, Luo B, Bai Y, et al. Association of handgrip strength and risk of cardiovascular disease: a population-based cohort study. *Aging Clin Exp Res* 2024;36(1):207. doi: 10.1007/s40520-024-02856-x

21. He YY, Jin ML, Fang XY, et al. Associations of muscle mass and strength with new-onset diabetes among middle-aged and older adults: evidence from the China health and retirement longitudinal study (CHARLS). *Acta Diabetologica* 2024;61(7):869-78. doi: 10.1007/s00592-024-02265-6

22. Lee SB, Jo MK, Moon JE, et al. Relationship between Handgrip Strength and Incident Diabetes in Korean Adults According to Gender: A Population-Based Prospective Cohort Study. *Journal of Clinical Medicine* 2024;13(2) doi: 10.3390/jcm13020627

23. Park D, Rho J, Kim E, et al. Comparison of Absolute and Relative Grip Strength to Predict Incidence of Diabetes Mellitus in Korea: A Prospective Cohort Study. *Metabolic Syndrome and Related Disorders* 2024;22(6):463-70. doi: 10.1089/met.2024.0006

24. Sohn YJ, Lee HS, Bae H, et al. Association of relative handgrip strength on the development of diabetes mellitus in elderly Koreans. *PLoS One* 2024;19(10):e0309558. doi: 10.1371/journal.pone.0309558

25. Yan LJ, Ge HY, Wang ZM, et al. Roles of low muscle strength and sarcopenic obesity on incident symptomatic knee osteoarthritis: A longitudinal cohort study. *Plos One* 2024;19(10) doi: 10.1371/journal.pone.0311423

26. Kuo K, Zhang YR, Chen SD, et al. Associations of grip strength, walking pace, and the risk of incident dementia: A prospective cohort study of 340212 participants. *Alzheimers & Dementia* 2023;19(4):1415-27. doi: 10.1002/alz.12793

27. Wu KM, Kuo K, Deng YT, et al. Association of grip strength and walking pace with the risk of incident Parkinson's disease: a prospective cohort study of 422,531 participants. *J Neurol* 2024;271(5):2529-38. doi: 10.1007/s00415-024-12194-7
