# Supplementary material for: Clinical importance of simple muscular fitness tests to predict long-term health conditions: a systematic review and meta-analysis of 94 cohort studies
Source: Br J Sports Med. 2026 Feb 10;60(6):e109173. doi: 10.1136/bjsports-2024-109173 (PMC13018823; doi:10.1136/bjsports-2024-109173)
Supplement: online supplemental appendix 2 [file bjsports-60-6-s002.docx]

# **Appendix 2. Online Supplemental Tables**

**Table S1**. Consensus-based criteria for downgrading or upgrading GRADE domains mains.

| **Domain** | **Judgment** | **Scoring** | **Criteria** |
| --- | --- | --- | --- |
| **Risk of Bias** | No serious ROB | 0 | - For each outcome, most (≥80%) studies included in the meta-analysis are assessed as having an overall low risk of bias. The remaining the studies are assessed to be at overall moderate risk of bias and they don´t have substantial statistical weight. |
|  | Serious ROB | –1 point | - For each outcome, <80% of studies included in the meta-analysis are assessed to be at low risk of bias, and the rest of the studies are assessed to be at overall moderate risk of bias, due to bias from study participation, study attrition, prognostic factor measurement, outcome measurement, study confounding and statistical analysis and reporting. |
|  | Very serious ROB | –2 points | - For each outcome, at least one of the primary studies included in the meta-analysis is assessed to be of overall high risk of bias, due to bias from study participation, study attrition, prognostic factor measurement, outcome measurement, study confounding and statistical analysis and reporting. But the relative contribution of each study to the overall results will be considered for assessing the overall level of bias. - Risk of biases include: 1) Study participation: not having appropriate eligibility criteria and the populations are not generalizable; 2) Study attrition: high loss to follow-up, significant differences between participants who completed the study and those who did not and lack of methods for handling missing data; 3) Prognostic factor measurement: lack of a clear description of the prognostic factor, lack of validity and reliability of the measurement, the method and environment for measuring the prognostic factor are not the same for all study participants, and there is a lack of standardized measurement and quantification; 4) Outcome measurement: lack of a clear description of the outcome, lack of validity and reliability of the outcome measurement, the method and environment for measuring the outcome are not the same for all study participants, and there is a lack of standardized measurement and quantification; 5) Study confounding: lack of identification, control and measurement of important confounders, lack of appropriate statistical; 6) Statistical analysis and reporting: the statistical analysis is inadequate, and not all primary outcomes are reported. |
| **Inconsistency** | No serious inconsistency | 0 | - Results consistent in direction and magnitude. - Point estimates are similar across studies with overlap of confidence intervals. - Statistical tests for heterogeneity are not significant and the I^2^ is low to moderate (i.e., <50%). |
|  | Serious inconsistency | –1 point | - Heterogeneity is substantial and significant (i.e., ≥50%, p<0.05). |
|  | Very serious inconsistency | –2 points | - Point estimates vary widely across studies. - Confidence intervals show minimal or no overlap. - Heterogeneity is high and significant ((i.e., ≥75%, p<0.05). |
| **Indirectness** | No serious indirectness | 0 | For each outcome, most (≥80%) studies included in the meta-analysis, must meet the following criteria:   - There was good global representation within the primary studies (e.g., variety of populations, sex and age distribution). - Both tests used to assess muscle strength follow standardized and validated protocols. - The outcome is clearly defined, adequately measuring the final result without substitutes. - The follow-up reasonably allows for the observation of new cases. - The remaining studies (<20%) can be assessed as having serious indirectness, but they don´t have substantial statistical weight. |
|  | Serious indirectness | –1 point | For each outcome, the studies included in the meta-analysis were limited by having one of the following criteria:   - Limited global representation of primary studies with consideration for their relative contribution to the estimate. - Both tests used to assess muscle strength don´t follow standardized and validated protocols. - The outcome is not clearly defined, inadequately measuring the final result with substitutes. - The follow-up doesn’t allow for the observation of new cases. |
|  | Very serious indirectness | –2 points | For each outcome, the studies included in the meta-analysis were limited by having two or more of the following criteria:   - Limited global representation of primary studies with consideration for their relative contribution to the estimate. - Both tests used to assess muscle strength don´t follow standardized and validated protocols. - The outcome is not clearly defined, inadequately measuring the final result with substitutes. - The follow-up doesn’t allow for the observation of new cases. |
| **Imprecision** | No serious imprecision | 0 | Each outcome must meet the following criteria:   - Total number of participants was ≥4000. - The 95% CIs for the pooled estimates include no effect, but the sample size is ≥4000 and the CIs exclude important benefit or harm (i.e., 10%). |
|  | Serious imprecision | –1 point | Each outcome was limited by having one of the following criteria:   - Total number of participants was <4000. - The 95% CIs for the pooled estimate do not include 1.0 but are very wide despite a large sample size. - The 95% CIs for the pooled estimates include 1.0 and the sample size is ≥4000, but the CIs include important benefit or harm (i.e., 10%). For example, sample >900,000, 0R = 1.03, 95% CI: 0.96, 1.10. |
|  | Very serious imprecision | –2 points | - Each outcome was limited by have two of the criteria established in serious imprecision. - There are very few events and the CIs around the estimates of effect include both appreciable benefit and appreciable harm. |
| **Publication Bias**^5^ | No serious publication bias | 0 | For each outcome, most (≥80%) studies included in the meta-analysis, must meet the following criteria:   - There is no suspected evidence of publication bias as reported in the systematic review based on Egger’s test and/or visual inspection of funnel plots. - There were an insufficient number of included studies to assess publication bias (<10 studies). - The search strategy appears comprehensive. - The remaining studies (<20%) can be assessed as having serious publication bias, but they don´t have substantial statistical weight. |
|  | Serious publication bias | –1 point | For each outcome, the studies included in the meta-analysis were limited by having one of the following criteria:   - There is evidence of publication bias arising from asymmetrical rather than symmetrical funnel plots, and/or statistical tests of asymmetry are positive. - Publication bias was not assessed and there were ≥10 studies. - The search strategy is not comprehensive. |
|  | Very serious publication bias | –2 points | For each outcome, the studies included in the meta-analysis were limited by having two or more of the following criteria:   - There is evidence of publication bias arising from asymmetrical rather than symmetrical funnel plots, and/or statistical tests of asymmetry are positive. - Publication bias was not assessed and there were ≥10 studies. - The search strategy is not comprehensive. |
| **Large magnitude of an effect** |  | 0 points | - OR≥0.5, no large effect. |
|  |  | +1 point | - OR<0.5, large effect. |
|  |  | +2 points | - OR<0.2, very large effect. |
| **Dose-response gradient** |  | 0 points | - Absence of a dose-response gradient. |
|  |  | +1 point | - Presence of a dose-response gradient. |
| **Effect of plausible residual confounding** |  | 0 points | - Residual confounding could plausibly explain part of the observed association. |
|  |  | +1 point | - Residual confounding would likely attenuate rather than explain the observed association. |

# **Table S2.** Study quality ratings based on the Newcastle Ottawa Scale for cohort studies.

| Author,  publication year | 1)  Representativeness of the exposed cohort | 2) Selection of the non-exposed cohort | 3) Ascertainment of exposure | 4) Demonstration that outcome of interest was not present at start of study | 5) Comparability of cohorts based on the design or analysis* | 6) Assessment of outcome | 7) Was follow-up long enough for outcomes to occur | 8) Adequacy of follow up of cohorts | **Overall score** |
| --- | --- | --- | --- | --- | --- | --- | --- | --- | --- |
| **CARDIOVASCULAR DISEASES (n=18)** | | | | | | | | | |
| **Camargo et al. 2016^1^** | 1 | 1 | 1 | 1 | 2 | 1 | 1 | 1 | **9** |
| **Celis-Morales et al. 2018^2^** | 1 | 1 | 1 | 1 | 2 | 1 | 1 | 1 | **9** |
| **Gubelmann et al. 2017^3^** | 1 | 1 | 1 | 1 | 2 | 1 | 1 | 1 | **9** |
| Ho et al. 2019^4^ | 1 | 1 | 1 | 1 | 2 | 0 | 1 | 1 | **8** |
| **Jang et al. 2020^5^** | 1 | 1 | 1 | 1 | 2 | 1 | 1 | 1 | **9** |
| **Klinpudtan et al. 2021^6^** | 1 | 1 | 1 | 1 | 1 | 1 | 1 | 1 | **8** |
| Kim et al. 2021^7^ | 1 | 1 | 1 | 1 | 2 | 1 | 1 | 1 | **9** |
| **Leong et al. 2015^8^** | 1 | 1 | 1 | 1 | 2 | 1 | 1 | 1 | **9** |
| **Li et al. 2023^9^** | 1 | 1 | 1 | 1 | 2 | 1 | 1 | 1 | **9** |
| **Liu et al. 2021^10^** | 1 | 1 | 1 | 1 | 2 | 1 | 1 | 1 | **9** |
| **Liu et al. 2024a^11^** | 1 | 1 | 1 | 1 | 2 | 1 | 1 | 1 | **9** |
| **McGrath et al. 2020^12^** | 1 | 1 | 1 | 1 | 2 | 1 | 1 | 1 | **9** |
| **Peralta et al. 2023^13^** | 1 | 1 | 1 | 1 | 2 | 1 | 0 | 1 | **8** |
| Qi et al. 2024^14^ | 1 | 1 | 1 | 1 | 2 | 1 | 1 | 1 | **9** |
| **Sillars et al. 2019^15^** | 1 | 1 | 1 | 1 | 2 | 1 | 1 | 1 | **9** |
| Yang et al. 2024^16^ | 1 | 1 | 1 | 1 | 2 | 1 | 1 | 1 | **9** |
| **Zhang et al. 2023^17^** | 1 | 1 | 1 | 1 | 2 | 1 | 1 | 1 | **9** |
| **Zhang et al. 2024^18^** | 1 | 1 | 1 | 1 | 2 | 1 | 1 | 1 | **9** |
| **CANCER (n=6)** | | | | | | | | | |
| **Celis-Morales et al. 2018^2^** | 1 | 1 | 1 | 1 | 2 | 1 | 1 | 1 | **9** |
| Ho et al. 2019^4^ | 1 | 1 | 1 | 1 | 2 | 0 | 1 | 1 | **8** |
| **Leong et al. 2015^8^** | 1 | 1 | 1 | 1 | 2 | 1 | 1 | 1 | **9** |
| Parra-Soto et al. 2022^19^ | 1 | 1 | 1 | 1 | 2 | 1 | 1 | 1 | **9** |
| Parra-Soto et al. 2021^20^ | 1 | 1 | 1 | 1 | 2 | 1 | 1 | 1 | **9** |
| **Weber et al. 2021^21^** | 1 | 1 | 1 | 1 | 2 | 1 | 1 | 1 | **9** |
| **TYPE 2 DIABETES MELLITUS (n=22)** | | | | | | | | | |
| **Boonpor et al. 2021^22^** | 1 | 1 | 1 | 1 | 2 | 1 | 1 | 1 | **9** |
| **Hao et al. 2020^23^** | 1 | 1 | 1 | 1 | 2 | 1 | 1 | 1 | **9** |
| **He et al. 2024^24^** | 1 | 1 | 1 | 1 | 2 | 1 | 1 | 1 | **9** |
| **Jeon et al. 2021^25^** | 1 | 1 | 1 | 1 | 2 | 1 | 1 | 1 | **9** |
| Karvonen-Gutierrez et al. 2018^26^ | 0 | 1 | 1 | 1 | 2 | 1 | 1 | 1 | **8** |
| **Katzmarzyk et al. 2007^27^** | 1 | 1 | 1 | 1 | 2 | 1 | 1 | 1 | **9** |
| **Kowall et al. 2022^28^** | 1 | 1 | 1 | 1 | 2 | 1 | 1 | 1 | **9** |
| Kunutsor et al. 2021^29^ | 1 | 1 | 1 | 1 | 2 | 1 | 1 | 1 | **9** |
| **Larsen et al. 2016^30^** | 0 | 1 | 1 | 1 | 1 | 1 | 1 | 1 | **7** |
| Lee et al. 2024^31^ | 1 | 1 | 1 | 1 | 2 | 1 | 1 | 1 | **9** |
| **Leong et al. 2015^8^** | 1 | 1 | 1 | 1 | 2 | 1 | 1 | 1 | **9** |
| **Li et al. 2016^32^** | 1 | 1 | 1 | 1 | 2 | 1 | 1 | 1 | **9** |
| **Li et al. 2021^33^** | 1 | 1 | 1 | 1 | 2 | 1 | 1 | 1 | **9** |
| Mahala Manda et al. 2020^34^ | 0 | 1 | 1 | 1 | 2 | 1 | 1 | 1 | **8** |
| **Marques-Vidal et al. 2017^35^** | 1 | 1 | 1 | 1 | 2 | 1 | 1 | 1 | **9** |
| McGrath et al. 2017^36^ | 1 | 1 | 1 | 1 | 1 | 0 | 1 | 1 | **7** |
| **Momma et al. 2019^37^** | 1 | 1 | 1 | 1 | 2 | 1 | 1 | 1 | **9** |
| **Park et al. 2024^38^** | 1 | 1 | 1 | 1 | 2 | 1 | 1 | 1 | **9** |
| Qiu et al. 2023^39^ | 1 | 1 | 1 | 1 | 2 | 1 | 1 | 1 | **9** |
| Sohn et al. 2024^40^ | 1 | 1 | 1 | 1 | 2 | 1 | 1 | 1 | **9** |
| **Wander et al. 2011^41^** | 1 | 1 | 1 | 1 | 2 | 1 | 1 | 1 | **9** |
| Zheng et al. 2022^42^ | 1 | 1 | 1 | 1 | 2 | 1 | 1 | 1 | **9** |
| **RESPIRATORY DISEASES (n=3)** | | | | | | | | | |
| **Celis-Morales et al. 2018^2^** | 1 | 1 | 1 | 1 | 2 | 1 | 1 | 1 | **9** |
| Ho et al. 2019^4^ | 1 | 1 | 1 | 1 | 2 | 0 | 1 | 1 | **8** |
| **Leong et al. 2015^8^** | 1 | 1 | 1 | 1 | 2 | 1 | 1 | 1 | **9** |
| **MUSCULOSKELETAL IMPAIRMENT (n=30)** | | | | | | | | | |
| **Alajlouni et al. 2021^43^** | 0 | 1 | 1 | 1 | 0 | 0 | 1 | 1 | **5** |
| Albrand et al. 2003^44^ | 0 | 1 | 1 | 1 | 2 | 1 | 1 | 1 | **8** |
| Beauchamp et al. 2022^45^ | 1 | 1 | 1 | 1 | 2 | 1 | 1 | 1 | **9** |
| **Cawthon et al. 2008^46^** | 0 | 1 | 1 | 1 | 2 | 1 | 1 | 1 | **8** |
| Cheung et al. 2012^47^ | 1 | 1 | 1 | 1 | 2 | 1 | 1 | 1 | **9** |
| Delbaere et al. 2006^48^ | 1 | 1 | 1 | 1 | 0 | 0 | 1 | 1 | **6** |
| Finigan et al. 2008^49^ | 0 | 1 | 1 | 1 | 1 | 1 | 1 | 1 | **8** |
| **Guo et al. 2024^50^** | 1 | 1 | 1 | 1 | 2 | 0 | 1 | 1 | **8** |
| **Hussain et al. 2024^51^** | 1 | 1 | 1 | 1 | 2 | 1 | 1 | 1 | **9** |
| **Kamiya 2019^52^** | 1 | 1 | 1 | 1 | 2 | 1 | 1 | 1 | **9** |
| Kärkkäinen et al. 2008^53^ | 1 | 1 | 1 | 1 | 2 | 0 | 1 | 1 | **8** |
| **Leong et al. 2015^8^** | 1 | 1 | 1 | 1 | 2 | 1 | 1 | 1 | **9** |
| Minneci et al. 2015^54^ | 0 | 1 | 1 | 1 | 1 | 0 | 1 | 1 | **6** |
| **Muraki et al. 2013^55^** | 1 | 1 | 1 | 1 | 2 | 1 | 1 | 1 | **9** |
| Ooi et al. 2021^56^ | 1 | 1 | 1 | 1 | 2 | 1 | 1 | 1 | **9** |
| **Pham et al. 2023^57^** | 1 | 1 | 1 | 1 | 2 | 1 | 1 | 1 | **9** |
| Rikkonen et al. 2012^58^ | 1 | 1 | 1 | 1 | 0 | 1 | 1 | 1 | **7** |
| Roongbenjawan et al. 2020^59^ | 0 | 1 | 1 | 1 | 2 | 1 | 0 | 1 | **7** |
| **Rouzi et al. 2015^60^** | 0 | 1 | 1 | 1 | 2 | 1 | 1 | 1 | **8** |
| **Schaap et al. 2017^61^** | 0 | 1 | 1 | 1 | 1 | 0 | 1 | 1 | **6** |
| Søgaard et al. 2020^62^ | 1 | 1 | 1 | 1 | 2 | 1 | 1 | 1 | **9** |
| **Stel et al. 2003^63^** | 0 | 1 | 1 | 1 | 1 | 0 | 1 | 1 | **6** |
| **Stel et al. 2004^64^** | 0 | 1 | 1 | 1 | 1 | 1 | 1 | 1 | **7** |
| Valenzuela et al. 2020^65^ | 0 | 1 | 1 | 1 | 1 | 0 | 1 | 1 | **6** |
| Wang et al. 2024^66^ | 1 | 1 | 1 | 1 | 2 | 1 | 1 | 1 | **9** |
| **Welmer et al. 2017^67^** | 1 | 1 | 1 | 1 | 2 | 1 | 1 | 1 | **9** |
| **Yan et al. 2024^68^** | 1 | 1 | 1 | 1 | 2 | 1 | 1 | 1 | **9** |
| **Zhang et al. 2013^69^** | 0 | 1 | 1 | 1 | 0 | 0 | 1 | 1 | **5** |
| **Zhong et al. 2021^70^** | 1 | 1 | 1 | 1 | 1 | 0 | 1 | 1 | **7** |
| Zhou et al. 2022^71^ | 1 | 1 | 1 | 1 | 2 | 1 | 1 | 1 | **9** |
| **DISABILITY (disability in activities of daily living, functional mobility, ambulatory status, n=31)** | | | | | | | | | |
| **Al Snih et al. 2004^72^** | 1 | 1 | 1 | 1 | 2 | 1 | 1 | 1 | **9** |
| Burbank et al. 2023^73^ | 1 | 1 | 1 | 1 | 2 | 1 | 1 | 1 | **9** |
| **Carrière et al. 2005^74^** | 0 | 1 | 1 | 1 | 0 | 1 | 1 | 1 | **6** |
| **Cesari et al. 2009^75^** | 1 | 1 | 1 | 1 | 2 | 1 | 1 | 1 | **9** |
| Chan et al. 2014^76^ | 1 | 1 | 1 | 1 | 1 | 1 | 1 | 1 | **8** |
| **Coelho-Júnior et al. 2024^77^** | 0 | 1 | 1 | 1 | 2 | 0 | 0 | 1 | **6** |
| **Dai et al. 2023^78^** | 1 | 1 | 1 | 1 | 2 | 0 | 1 | 1 | **8** |
| **den Ouden et al. 2013^79^** | 1 | 1 | 1 | 1 | 2 | 1 | 1 | 1 | **9** |
| **Dodds et al. 2018^80^** | 1 | 1 | 1 | 1 | 2 | 0 | 1 | 1 | **8** |
| Giampaoli et al. 1999^81^ | 0 | 1 | 1 | 1 | 0 | 1 | 1 | 1 | **6** |
| Gill et al. 1995^82^ | 1 | 1 | 1 | 1 | 2 | 1 | 1 | 1 | **9** |
| Gonzalez-Bautista et al. 2022^83^ | 1 | 1 | 1 | 1 | 2 | 1 | 1 | 1 | **9** |
| Hicks et al. 2012^84^ | 1 | 1 | 1 | 1 | 0 | 0 | 1 | 1 | **6** |
| **Huang et al. 2024^85^** | 1 | 1 | 1 | 1 | 2 | 1 | 1 | 1 | **9** |
| **Ishizaki et al. 1999^86^** | 1 | 1 | 1 | 1 | 0 | 1 | 1 | 1 | **7** |
| **Jung et al. 2016^87^** | 0 | 1 | 1 | 1 | 2 | 0 | 1 | 1 | **7** |
| **Legrand et al. 2014^88^** | 1 | 1 | 1 | 1 | 2 | 1 | 1 | 1 | **9** |
| **Lopez-Teros et al. 2022^89^** | 1 | 1 | 1 | 1 | 2 | 1 | 1 | 1 | **9** |
| **Marincolo et al. 2024^90^** | 1 | 1 | 1 | 1 | 2 | 1 | 1 | 1 | **9** |
| Minneci et al. 2015^54^ | 0 | 1 | 1 | 1 | 1 | 0 | 1 | 1 | **6** |
| **Morera et al. 2023^91^** | 1 | 1 | 1 | 1 | 2 | 1 | 1 | 1 | **9** |
| **Okabe et al. 2017^92^** | 1 | 1 | 1 | 1 | 2 | 1 | 1 | 1 | **9** |
| Peterson et al. 2021^93^ | 1 | 1 | 1 | 1 | 2 | 1 | 1 | 1 | **9** |
| **Rantanen et al. 1999^94^** | 1 | 1 | 1 | 1 | 2 | 1 | 1 | 1 | **9** |
| **Seidel et al 2011^95^** | 1 | 1 | 1 | 1 | 2 | 1 | 1 | 1 | **9** |
| Shinkai et al. 2003^96^ | 1 | 1 | 1 | 1 | 0 | 1 | 1 | 1 | **7** |
| Sirola et al. 2010^97^ | 1 | 1 | 1 | 1 | 0 | 0 | 1 | 1 | **6** |
| **Song et al. 2024^98^** | 1 | 1 | 1 | 1 | 2 | 1 | 1 | 1 | **9** |
| **Stessman et al. 2017^99^** | 1 | 1 | 1 | 1 | 1 | 1 | 1 | 1 | **8** |
| **Taekema et al. 2010^100^** | 0 | 1 | 1 | 1 | 1 | 1 | 1 | 1 | **7** |
| Zhang et al. 2022^101^ | 1 | 1 | 1 | 1 | 2 | 1 | 1 | 1 | **9** |
| **ANXIETY (diagnosis or moderate to severe symptoms, n=3)** | | | | | | | | | |
| **Cabanas-Sánchez et al. 2022^102^** | 1 | 1 | 1 | 1 | 2 | 1 | 1 | 1 | **9** |
| **Carvalho 2019^103^** | 1 | 1 | 1 | 1 | 2 | 1 | 1 | 1 | **9** |
| **Gordon et al. 2019^104^** | 1 | 1 | 1 | 1 | 2 | 1 | 1 | 1 | **9** |
| **DEPRESSION (diagnosis or moderate to severe symptoms, n=17)** | | | | | | | | | |
| **Bao et al. 2022^105^** | 1 | 1 | 1 | 1 | 2 | 1 | 1 | 1 | **9** |
| **Cabanas-Sánchez et al. 2022^102^** | 1 | 1 | 1 | 1 | 2 | 1 | 1 | 1 | **9** |
| **Carvalho 2019^103^** | 1 | 1 | 1 | 1 | 2 | 1 | 1 | 1 | **9** |
| Chan et al. 2024^106^ | 1 | 1 | 1 | 1 | 2 | 1 | 1 | 1 | **9** |
| **Gu et al. 2023^107^** | 1 | 1 | 1 | 1 | 2 | 1 | 1 | 1 | **9** |
| **Hamer, Batty and Kivimaki 2015^108^** | 1 | 1 | 1 | 1 | 2 | 1 | 1 | 1 | **9** |
| Lian et al. 2021^109^ | 1 | 1 | 1 | 1 | 2 | 1 | 1 | 1 | **9** |
| **López-Bueno et al. 2023^110^** | 1 | 1 | 1 | 1 | 2 | 1 | 1 | 1 | **9** |
| **Luo et al. 2022^111^** | 1 | 1 | 1 | 1 | 2 | 1 | 1 | 1 | **9** |
| **McDowell, Gordon and Herring 2018^112^** | 1 | 1 | 1 | 1 | 2 | 1 | 1 | 1 | **9** |
| Mendorf et al. 2023^113^ | 1 | 1 | 1 | 1 | 2 | 1 | 1 | 1 | **9** |
| **Stessman et al. 2017^99^** | 1 | 1 | 1 | 1 | 1 | 1 | 1 | 1 | **8** |
| Song et al. 2024^114^ | 1 | 1 | 1 | 1 | 2 | 1 | 1 | 1 | **9** |
| **Taekema et al. 2010^100^** | 0 | 1 | 1 | 1 | 1 | 1 | 1 | 1 | **7** |
| **Veronese et al. 2017^115^** | 1 | 1 | 1 | 1 | 1 | 1 | 1 | 1 | **8** |
| **Zhao et al. 2020^116^** | 1 | 1 | 1 | 1 | 2 | 1 | 1 | 1 | **9** |
| Zheng et al. 2022^117^ | 1 | 1 | 1 | 1 | 2 | 1 | 1 | 1 | **9** |
| **HEALTH-RELATED QUALITY OF LIFE (n=4)** | | | | | | | | | |
| Balogun et al. 2019^118^ | 1 | 1 | 1 | 1 | 2 | 1 | 1 | 1 | **9** |
| Chan et al. 2014^76^ | 1 | 1 | 1 | 1 | 1 | 1 | 1 | 1 | **8** |
| Gomez-Bruton et al. 2021^119^ | 1 | 1 | 1 | 1 | 2 | 1 | 1 | 1 | **9** |
| Gum et al. 2018^120^ | 0 | 1 | 1 | 0 | 2 | 1 | 1 | 1 | **7** |
| **COGNITIVE DECLINE (n=26)** | | | | | | | | | |
| **Alfaro-Acha et al. 2006^121^** | 1 | 1 | 1 | 1 | 2 | 1 | 1 | 1 | **9** |
| Auyeung et al. 2011^122^ | 1 | 1 | 1 | 1 | 2 | 1 | 1 | 1 | **9** |
| Boyle et al. 2009^123^ | 1 | 1 | 1 | 1 | 2 | 1 | 1 | 1 | **9** |
| **Buchman et al. 2007^124^** | 0 | 1 | 1 | 1 | 2 | 1 | 1 | 1 | **8** |
| Chen et al. 2022^125^ | 1 | 1 | 1 | 1 | 2 | 0 | 1 | 1 | **8** |
| **Chou et al. 2019^126^** | 1 | 1 | 1 | 1 | 2 | 1 | 1 | 1 | **9** |
| **Feng et al. 2023^127^** | 0 | 1 | 1 | 1 | 1 | 0 | 0 | 1 | **5** |
| **Heward et al. 2018^128^** | 1 | 1 | 1 | 1 | 0 | 1 | 1 | 1 | **7** |
| **Jeong and Kim 2018^129^** | 1 | 1 | 1 | 1 | 2 | 1 | 1 | 1 | **9** |
| Jeong et al. 2018^130^ | 0 | 1 | 1 | 1 | 2 | 1 | 1 | 1 | **8** |
| Jian et al. 2022^131^ | 1 | 1 | 1 | 1 | 1 | 1 | 1 | 1 | **8** |
| **Kang et al. 2021^132^** | 1 | 1 | 1 | 1 | 2 | 1 | 1 | 1 | **9** |
| Kim 2019^133^ | 1 | 1 | 1 | 1 | 2 | 1 | 1 | 1 | **9** |
| Kim and Kim 2022^134^ | 1 | 1 | 1 | 1 | 2 | 1 | 1 | 1 | **9** |
| Kim et al. 2019a^135^ | 1 | 1 | 1 | 1 | 1 | 1 | 1 | 1 | **8** |
| Kim et al. 2019b^136^ | 1 | 1 | 1 | 1 | 2 | 1 | 1 | 1 | **9** |
| **McGrath et al. 2019a^137^** | 1 | 1 | 1 | 1 | 2 | 1 | 1 | 1 | **9** |
| **McGrath et al. 2019b^138^** | 1 | 1 | 1 | 1 | 2 | 1 | 1 | 1 | **9** |
| Peng et al. 2023^139^ | 0 | 1 | 1 | 1 | 2 | 1 | 0 | 1 | **7** |
| Peng et al. 2024^140^ | 1 | 1 | 1 | 1 | 2 | 1 | 1 | 1 | **9** |
| Sattler et al. 2011^141^ | 0 | 1 | 1 | 1 | 1 | 1 | 1 | 1 | **7** |
| **Stessman et al. 2017^99^** | 1 | 1 | 1 | 1 | 1 | 1 | 1 | 1 | **8** |
| Stijntjes et al. 2017^142^ | 1 | 1 | 1 | 1 | 2 | 1 | 1 | 1 | **9** |
| **Taekema et al. 2010^100^** | 0 | 1 | 1 | 1 | 1 | 1 | 1 | 1 | **7** |
| **Veronese et al. 2016^143^** | 1 | 1 | 1 | 1 | 1 | 1 | 1 | 1 | **8** |
| Werneck et al. 2023^144^ | 1 | 1 | 1 | 1 | 2 | 1 | 1 | 1 | **9** |
| **DEMENTIA (n=12)** | | | | | | | | | |
| **Camargo et al. 2016^1^** | 1 | 1 | 1 | 1 | 2 | 1 | 1 | 1 | **9** |
| **Doi et al. 2019^145^** | 1 | 1 | 1 | 1 | 2 | 1 | 1 | 1 | **9** |
| **Duchowny et al. 2022^146^** | 1 | 1 | 1 | 1 | 2 | 1 | 1 | 1 | **9** |
| **Esteban-Cornejo et al. 2022^147^** | 1 | 1 | 1 | 1 | 2 | 1 | 1 | 1 | **9** |
| **Hatabe et al. 2020^148^** | 1 | 1 | 1 | 1 | 2 | 1 | 1 | 1 | **9** |
| **He et al. 2023^149^** | 1 | 1 | 1 | 1 | 2 | 1 | 1 | 1 | **9** |
| **Kuo et al. 2022^150^** | 1 | 1 | 1 | 1 | 2 | 1 | 1 | 1 | **9** |
| Peterson et al. 2021^93^ | 1 | 1 | 1 | 1 | 2 | 1 | 1 | 1 | **9** |
| Sattler et al. 2011^141^ | 0 | 1 | 1 | 1 | 1 | 1 | 1 | 1 | **7** |
| Sibbett et al. 2018^151^ | 0 | 1 | 1 | 1 | 1 | 1 | 1 | 1 | **7** |
| **Stephan et al. 2024^152^** | 1 | 1 | 1 | 1 | 2 | 1 | 1 | 1 | **9** |
| Werneck et al. 2023^144^ | 1 | 1 | 1 | 1 | 2 | 1 | 1 | 1 | **9** |
| **PARKINSON (n=3)** | | | | | | | | | |
| **Liu et al. 2024b^153^** | 1 | 1 | 1 | 1 | 2 | 1 | 1 | 1 | **9** |
| **Mey et al. 2023^154^** | 1 | 1 | 1 | 1 | 2 | 1 | 1 | 1 | **9** |
| **Wu et al. 2024^155^** | 1 | 1 | 1 | 1 | 2 | 1 | 1 | 1 | **9** |

*Note:* *Comparability of cohorts based on the design or analysis can be allotted with a maximum of 2 points. The maximum score for the rest of the studies is 1 point.

Bolds indicate studies included in the meta-analysis.

**Table S3**. Certainty of the evidence for the handgrip strength test assessed using the GRADE tool, explained by the downgrade and upgrade domains.

| **Long-term health conditions** | **Certainty (quality) of evidence. Traditional GRADE** |
| --- | --- |
| **Cardiovascular disease** | 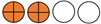  **Low certainty**  RoB: 0 points, ≥ 80% studies included were assessed an overall low RoB, and no studies as high RoB.  Inconsistency: -1 point, I^2^ = 50.9% and CIs overlap.  Indirectness: 0 points, good global representation, standardized and validated protocols to asses HG, no surrogate outcome.  Imprecision: 0 points, sample size > 4000, CIs do not include 1.  Publication bias: 0 points, funnel plot symmetry and Egger’s test p = 0.08.  Large magnitude of an effect: 0 points, large effect ≥ 0.5.  Dose-response gradient: +1 point, presence of a dose-response gradient.  Effect of plausible residual confounding: 0 points, residual confounding could plausibly explain part of the observed association. |
| **Cancer** | 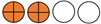  **Low certainty**  RoB: 0 points, ≥ 80% studies included were assessed an overall low RoB, and no studies as high RoB.  Inconsistency: 0 points, I^2^ < 50% and CIs overlap.  Indirectness: 0 points, good global representation, standardized and validated protocols to asses HG, no surrogate outcome.  Imprecision: -1 point, sample size > 4000, CIs include 1, but exclude important harm (i.e., 10%).  Publications bias: 0 points, insufficient number of studies to assess, search strategy appears comprehensive.  Large magnitude of an effect: 0 points, large effect ≥ 0.5.  Dose-response gradient: +1 point, presence of a dose-response gradient.  Effect of plausible residual confounding: 0 points, residual confounding could plausibly explain part of the observed association. |
| **Type 2 Diabetes Mellitus** | 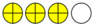  **Moderate certainty**  RoB: 0 points, ≥ 80% studies included were assessed an overall low RoB, and no studies as high RoB.  Inconsistency: 0 points, I^2^ < 50%, results are consistent in direction and magnitude and Cis overlap.  Indirectness: 0 points, good global representation, standardized and validated protocols to asses HG, no surrogate outcome.  Imprecision: 0 points, sample size > 4000, Cis do not include 1.  Publications bias: 0 points, insufficient number of studies to assess, search strategy appears comprehensive.  Large magnitude of an effect: 0 points, large effect ≥ 0.5.  Dose-response gradient: +1 point, presence of a dose-response gradient.  Effect of plausible residual confounding: 0 points, residual confounding could plausibly explain part of the observed association. |
| **Respiratory diseases** | 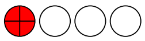  **Very low certainty**  RoB: 0 points, ≥ 80% studies included were assessed an overall low RoB, and no studies as high RoB.  Inconsistency: -2 point, I^2^ = 97%, point estimates vary widely across studies and CIs no overlap.  Indirectness: 0 points, good global representation, standardized and validated protocols to asses HG, no surrogate outcome.  Imprecision: -1 point, sample size > 4000, CIs include 1, but exclude important harm (i.e., 10%).  Publications bias: 0 points, insufficient number of studies to assess, search strategy appears comprehensive.  Large magnitude of an effect: 0 points, large effect ≥ 0.5.  Dose-response gradient: +1 point, presence of a dose-response gradient.  Effect of plausible residual confounding: 0 points, residual confounding could plausibly explain part of the observed association. |
| **Musculoskeletal impairment** | 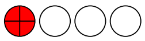  **Very low certainty**  RoB: -1 point, > 20% studies included were assessed an overall moderate RoB, and no studies as high RoB.  Inconsistency: -1 point, I^2^ = 51.6, results are consistent in direction and CIs minimal overlap.  Indirectness: 0 points, good global representation, standardized and validated protocols to asses HG, no surrogate outcome.  Imprecision: 0 points, sample size > 4000, CIs do not include 1.  Publication bias: 0 points, funnel plot symmetry and Egger’s test p = 0.22.  Large magnitude of an effect: 0 points, large effect ≥ 0.5.  Dose-response gradient: +1 point, presence of a dose-response gradient.  Effect of plausible residual confounding: 0 points, residual confounding could plausibly explain part of the observed association. |
| **Disability (disability in activities of daily living, functional mobility, ambulatory status)** | 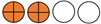  **Low certainty**  RoB: 0 points, ≥ 80% studies included were assessed an overall low RoB, and no studies as high RoB.  Inconsistency: 0 points, I^2^ < 50%, results are consistent in direction and CIs overlap.  Indirectness: -1 point, good global representation, standardized and validated protocols to assess HG, but surrogate outcome.  Imprecision: 0 points, sample size > 4000, CIs do not include 1.  Publication bias: 0 points, funnel plot symmetry and Egger’s test p = 0.34.  Large magnitude of an effect: 0 points, large effect ≥ 0.5.  Dose-response gradient: +1 point, presence of a dose-response gradient.  Effect of plausible residual confounding: 0 points, residual confounding could plausibly explain part of the observed association. |
| **Anxiety (diagnosis or moderate to severe symptoms)** | 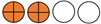  **Low certainty**  RoB: 0 points, ≥ 80% studies included were assessed an overall low RoB, and no studies as high RoB.  Inconsistency: 0 points, I^2^ = 0%, results are consistent in direction and CIs overlap.  Indirectness: -1 point, good global representation, standardized and validated protocols to asses HG, but surrogate outcome.  Imprecision: 0 points, sample size > 4000, CIs do not include 1.  Publications bias: 0 points, insufficient number of studies to assess, search strategy appears comprehensive.  Large magnitude of an effect: 0 points, large effect ≥ 0.5.  Dose-response gradient: +1 point, presence of a dose-response gradient.  Effect of plausible residual confounding: 0 points, residual confounding could plausibly explain part of the observed association. |
| **Depression (diagnosis or mild to severe symptoms)** | 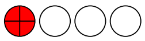  **Very low certainty**  RoB: 0 points, ≥ 80% studies included were assessed an overall low RoB, and no studies as high RoB.  Inconsistency: -1 point, I^2^ = 52.1%, results are consistent in direction and magnitude, and CIs overlap.  Indirectness: -1 point, good global representation, standardized and validated protocols to asses HG, but surrogate outcome.  Imprecision: 0 points, sample size > 4000, CIs do not include 1.  Publications bias: 0 points, insufficient number of studies to assess, search strategy appears comprehensive.  Large magnitude of an effect: 0 points, large effect ≥ 0.5.  Dose-response gradient: +1 point, presence of a dose-response gradient.  Effect of plausible residual confounding: 0 points, residual confounding could plausibly explain part of the observed association. |
| **Cognitive decline** | 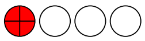  **Very low certainty**  RoB: 0 points, ≥ 80% studies included were assessed an overall low RoB, and no studies as high RoB.  Inconsistency: -1 point, I^2^ = 73.5%, results are consistent in direction and CIs minimal overlap.  Indirectness: -1 point, good global representation, standardized and validated protocols to asses HG, but surrogate outcome.  Imprecision: 0 points, sample size > 4000, CIs do not include 1.  Publications bias: 0 points, insufficient number of studies to assess, search strategy appears comprehensive.  Large magnitude of an effect: 0 points, large effect ≥ 0.5.  Dose-response gradient: +1 point, presence of a dose-response gradient.  Effect of plausible residual confounding: 0 points, residual confounding could plausibly explain part of the observed association. |
| **Dementia** | 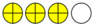  **Moderate certainty**  RoB: 0 points, ≥ 80% studies included were assessed an overall low RoB, and no studies as high RoB.  Inconsistency: 0 point, I^2^ = 0%, results are consistent in direction and CIs overlap.  Indirectness: 0 points, good global representation, standardized and validated protocols to asses HG, no surrogate outcome.  Imprecision: 0 points, sample size > 4000, CIs do not include 1.  Publications bias: 0 points, insufficient number of studies to assess, search strategy appears comprehensive.  Large magnitude of an effect: 0 points, large effect ≥ 0.5.  Dose-response gradient: +1 point, presence of a dose-response gradient.  Effect of plausible residual confounding: 0 points, residual confounding could plausibly explain part of the observed association. |
| **Parkinson´s disease** | 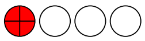  **Very low certainty**  RoB: 0 points, ≥ 80% studies included were assessed an overall low RoB, and no studies as high RoB.  Inconsistency: -2 point, I^2^ = 89%, point estimates vary widely across studies and CIs no overlap.  Indirectness: 0 points, good global representation, standardized and validated protocols to asses HG, no surrogate outcome.  Imprecision: -1 point, sample size > 4000, CIs do not include 1, but large 95%CI (upper/lower > 2).  Publications bias: 0 points, insufficient number of studies to assess, search strategy appears comprehensive.  Large magnitude of an effect: 0 points, large effect ≥ 0.5.  Dose-response gradient: +1 point, presence of a dose-response gradient.  Effect of plausible residual confounding: 0 points, residual confounding could plausibly explain part of the observed association. |

*Abbreviations:* CIs, confidence interval; HG, handgrip; RoB, risk of bias

**Table S4**. Certainty of the evidence for the handgrip strength test assessed using the GRADE tool, explained by the downgrade and upgrade domains.

| **Long-term health conditions** | **Certainty (quality) of evidence. Traditional GRADE** |
| --- | --- |
| **Type 2 Diabetes Mellitus** | 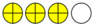  **Moderate certainty**  RoB: 0 points, ≥ 80% studies included were assessed an overall low RoB and no studies as high RoB.  Inconsistency: 0 points, I^2^ = 0% and results are consistent in direction and magnitude and CIs overlap.  Indirectness: 0 points, good global representation, standardized and validated protocols to asses HG, no surrogate outcome.  Imprecision: 0 points, sample size > 4000 and CIs do not include 1.  Publications bias: 0 points, insufficient number of studies to assess, search strategy appears comprehensive.  Large magnitude of an effect: 0 points, large effect ≥ 0.5.  Dose-response gradient: +1point, presence of a dose-response gradient.  Effect of plausible residual confounding: 0 points, residual confounding could plausibly explain part of the observed association. |
| **Musculoskeletal impairment** | 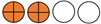  **Low certainty**  RoB: -1 point, > 20% studies included were assessed an overall moderate RoB and no studies as high RoB.  Inconsistency: 0 points, I^2^ = 0 and results are consistent in direction and CIs overlap.  Indirectness: 0 points, good global representation, standardized and validated protocols to asses HG, no surrogate outcome.  Imprecision: 0 points, sample size > 4000 and CIs do not include 1.  Publications bias: 0 points, insufficient number of studies to assess, search strategy appears comprehensive.  Large magnitude of an effect: 0 points, large effect ≥ 0.5.  Dose-response gradient: +1 point, presence of a dose-response gradient.  Effect of plausible residual confounding: 0 points, residual confounding could plausibly explain part of the observed association. |
| **Disability (disability in activities of daily living, functional mobility, ambulatory status)** | 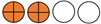  **Low certainty**  RoB: 0 points, ≥ 80% studies included were assessed an overall low RoB and no studies as high RoB.  Inconsistency: 0 points, I^2^ < 50% and results are consistent in direction and CIs overlap.  Indirectness: -1 point, good global representation, standardized and validated protocols to asses HG, but surrogate outcome.  Imprecision: 0 points, sample size > 4000 and CIs do not include 1.  Publications bias: 0 points, insufficient number of studies to assess, search strategy appears comprehensive.  Large magnitude of an effect: 0 points, large effect ≥ 0.5.  Dose-response gradient: +1 point, presence of a dose-response gradient.  Effect of plausible residual confounding: 0 points, residual confounding could plausibly explain part of the observed association. |
| **Depression (diagnosis or mild to severe symptoms)** | 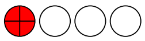  **Very low certainty**  RoB: 0 points, ≥ 80% studies included were assessed an overall low RoB, and no studies as high RoB.  Inconsistency: -1 point, I^2^ = 72% and results are consistent in direction and CIs overlap.  Indirectness: -1 point, good global representation, standardized and validated protocols to assess HG, but surrogate outcome.  Imprecision: -2 points, sample size < 4000 and large 95%CI (upper/lower > 2).  Publications bias: 0 points, insufficient number of studies to assess, search strategy appears comprehensive.  Large magnitude of an effect: 0 points, large effect ≥ 0.5.  Dose-response gradient: +1 point, presence of a dose-response gradient.  Effect of plausible residual confounding: 0 points, residual confounding could plausibly explain part of the observed association. |
| **Dementia** | 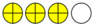  **Moderate certainty**  RoB: 0 points, ≥ 80% studies included were assessed an overall low RoB, and no studies as high RoB.  Inconsistency: 0 point, I^2^ = 0%, results are consistent in direction and CIs overlap.  Indirectness: 0 points, good global representation, standardized and validated protocols to asses HG, no surrogate outcome.  Imprecision: 0 points, sample size > 4000 and CIs do not include 1.  Publications bias: 0 points, insufficient number of studies to assess, search strategy appears comprehensive.  Large magnitude of an effect: 0 points, large effect ≥ 0.5.  Dose-response gradient: +1 point, presence of a dose-response gradient.  Effect of plausible residual confounding: 0 points, residual confounding could plausibly explain part of the observed association. |

*Abbreviations:* CIs, confidence interval; HG, handgrip; RoB, risk of bias

**Table S5.** Characteristics of the studies included in the systematic review.

| **Author, year** | **Cohort / Project** | **Participants, size, sex** | **Sample age, years (range or mean)** | **Follow-up, year** | **Fitness test (absolute/ relative)** | **Measurement tool** | **Sample categorization** | **Long-term health condition** | **Outcome measure** |
| --- | --- | --- | --- | --- | --- | --- | --- | --- | --- |
| **CARDIOVASCULAR DISEASES (n=18)** | | | | | | | | | |
| **Camargo et al. 2016^1^** | The Framingham Offspring Cohort | 2176  Females=1175  Males=1001 | 35-84  62±8 | 11-year | Handgrip Strength (absolute) | Dynamometer (Jamar Hydraulic Hand Dynamometer; Lafayette) | - Cut-off points:  ≤10th sex-specific percentile versus >10th percentile  Females=15 kg  Males=30 kg | Cardiovascular diseases: Stroke | Defined by clinical criteria. |
| **Celis-Morales et al. 2018^2^** | UK Biobank | 502293  Females=271238  Males=231055 | 40-69  56.5±8.1 | 7.1-year | Handgrip Strength (absolute) | Dynamometer (*N/A*) | - Sex-age specific HGS quartiles:  Females, <56 years, Q1=<21 kg; Q2=21-25 kg; Q3=26-29 kg; Q4=>29 kg. 56-65 years, Q1=<19 kg; Q2=18-22 kg; Q3=23-26 kg; Q4=>26 kg.  Males, <56 years, Q1=<36 kg; Q2=36-42 kg; Q3=43-48 kg; Q4=>48 kg. 56-65 years, Q1=<33 kg; Q2=33-38 kg; Q3=39-44 kg; Q4=>44 kg  - Change in HGS:  Per 5 kg decrement in HGS | Cardiovascular disease | Hospital admissions, with the International Classification of Diseases, 10th revision. |
| **Gubelmann et al. 2017^3^** | CoLaus (Cohorte Lausannoise) study | 2707  Females=1483  Males=1224 | 50-75 | 5.4-year | Handgrip Strength (absolute) | Dynamometer (Baseline®) | - Cut-off point for weakness=  21.7±6.5 kg (Fried et al. 2002 criterion) | Cardiovascular disease | Three equations: European Society of Cardiology SCORE, Framingham-2001 and PROCAM-2007. |
| Ho et al. 2019^4^ | UK Biobank | 356721  Females=194540  Males=162181 | 37-73 | 2-year | Handgrip Strength (absolute, and relative to height, weight, fat-free mass, BMI, fat-free mass index and fat-free mass) | Dynamometer (Jamar J00105) | Sex-age-specific quintiles:  Q1=≤21.5 kg; Q2=>21.5-27 kg; Q3=>27-33 kg; Q4=>33-41.5 kg; Q5=>41.5 kg | Cardiovascular disease | Medical history and medications for CVD were collected from the self-completed, baseline assessment questionnaire. |
| **Jang et al. 2020^5^** | Korean Longitudinal Study of Aging (KLoSA) | 5492  Females=3039  Males=2453 | 45-65  60.7±10.6 | 10-year | Handgrip Strength (absolute and relative by BMI) | Dynamometer (TANITA, Japan) | - Cut-off points for weakness:  Females=<16.0 kg  Males=<26.1 kg | Cardiovascular diseases | Diagnosed by a doctor. |
| Kim et al. 2021^7^ | UK Biobank | 284767  Females=154348  Males=130419 | 40-69 | 11.5-year | Handgrip Strength (absolute and normalized by fat-free mass) | Dynamometer (Jamar J00105) | - Sex-age-specific HGS tertiles (normalized, kg/kg):  Females, <50 years, T1=<0.53; T2=0.53-0.64; T3=>0.64. 50-59 years, T1=<0.49; T2=0.49-0.59; T3=>0.59. ≥60 years, T1=<0.44; T2=0.44-0.55; T3=>0.55.  Males, <50 years, T1=<0.61; T2=0.61-0.72; T3=>0.72. 50-59 years, T1=<0.58; T2=0.58-0.69; T3=>0.69. ≥60 years, T1=0.55; T2=0.55-0.66; T3=>0.66. | Stroke or myocardial infarction | Hospital admission records. |
| **Klinpudtan et al. 2021^6^** | The SONIC (Septuagenarians, Octogenarian, Nonagenarian, and Investigation with Centenarians) study (Japan) | 1272  *N/A* | ≥70 | 7-year | Handgrip Strength (absolute) | Dynamometer (*N/A*) | - HGS cut-off:  Females, HGS=<18 kg  Males, HGS=<26 kg | Coronary heart disease | Doctor’s diagnosis. |
| **Leong et al. 2015^8^** | Prospective Urban Rural Epidemiology (PURE) study | 139691  Females=81039  Males=58652 | 35-70 | 4-year | Handgrip Strength (absolute) | Dynamometer (Jamar) | - Sex-specific tertiles:  *N/A*  - Change in HGS:  Per 5 kg decrement in HGS | Myocardial infarction, stroke | Hospital admission. |
| **Li et al. 2023^9^** | - SHARE (Europe)  - HRS (US)  - CHARLS (China) | 75292 (Females=40659; Males=34633)  7790 (Females=4 384; Males=3406)  13181 (Females=6886; Males=6295) | ≥50 (±63.7 years)  ≥50 (±64.2 years)  ≥45 (±58.7 years) | 4.67-year  8.0-year  4.0-year | Handgrip Strength (bsolute and relative HGS/BMI) | Dynamometer (Smedley, TTM, Tokyo) | - Sex-age specific HGS quartiles:  Females, Q1=≤0.85; Q4=>1.17  Males, Q1=≤1.40; Q4=>1.84 | Stroke, Heart Disease | Self-reported physician diagnosis. |
| **Liu et al. 2021^10^** | China Health and Retirement Longitudinal Study (CHARLS) | 8871  Females=6350  Males=5887 | 45-95  59.0±9.6 | 4-year | Handgrip Strength (absolute) | Dynamometer (electronic dynamometer similar to Jamar) | - Cut-off points for weakness:  Females, HGS=<16.0 kg  Males, HGS <26.0 kg | Stroke | Self-reported. |
| **Liu et al. 2024a^11^** | UK biobank | 430886  Females=238280  Males=192606 | 56.44 | 13.81-year | Handgrip Strength (absolute and relative by body weigh) | Dynamometer (Jamar J00105) | - Sex-age specific HGS quartiles:  *N/A*  - Change in HGS:  Per 1 kg increment in absolute HGS  Per 0.01 kg/kg increase in relative HGS | Peripheral artery disease | Hospital admissions, with International Classification of Disease (10th revision) and the Office of Population Censuses and Surveys Classification of Interventions and Procedures (4th revision). |
| **McGrath et al. 2020^12^** | Health and Retirement Study (HRS) | 17431  Females=10226  Males=7187 | >50 | 4.7-year | Handgrip Strength (absolute) | Dynamometer (Smedley, Denmark) | - Sex-race-specific maximal HGS cut-off points, weakness:  Black females=<31 kg; White females=<22 kg  Black and White males=<35 kg | Chronic heart failure (CHF) | Doctor diagnosis. |
| **Peralta et al. 2023^13^** | Survey of Health, Aging and Retirement in Europe (SHARE) | 20829  Females=11489  Males=9340 | 63.1±9.6 | 13-year | Handgrip Strength (absolute) | Dynamometer (Smedley, Tokyo) | - Sex-age-specific quartiles:  Females, 50-54 years, Q1=<25 kg; Q2=26-29 kg; Q3=30-34 kg; Q4=>35 kg. 55-59 years, Q1=<24 kg; Q2=25-28 kg; Q3=28-32 kg; Q4=>33 kg. 60-64 years, Q1=<23 kg; Q2=24-27 kg; Q3=28-31 kg; Q4=>32 kg  Males, 50-54 years, Q1=<44 kg; Q2=45-49 kg; Q3=50-55 kg; Q4=>56 kg. 55-59 years, Q1=<41 kg; Q2=42-47 kg; Q3=48-53 kg; Q4=>54 kg. 60-64 years, Q1=<39 kg; Q2=40-44 kg; Q3=45-50 kg; Q4=>51 kg | Cardiovascular diseases | Self-reported. |
| Qi et al. 2024^14^ | CHARLS (China Health and Retirement Longitudinal Study) | 7904  Females=3659  Males=4245 | 45–85  58.89±9.08 | 7-year | Handgrip Strength (absolute, normalized by body weight) | Dynamometer (Yuejian WL-1000, Nantong, China) | - HGS tertiles:  Females, T1=<20.5 kg; T2=20.5–24.0 kg; T3=>24.0 kg  Males, T1=<32.5 kg; T2=32.5–38.0 kg; T3=>38.0 kg | Stroke Risk | Self-reported stroke diagnosis. |
| **Sillars et al. 2019^15^** | UK Biobank | 374493  *N/A* | 40-69 | 4.1-year | Handgrip Strength (absolute) | Dynamometer (Jamar J00105 hydraulic hand dynamometer) | - Quartiles:  *N/A*  - Change in HGS:  Per 5 kg increment in HGS | Incident heart failure | Hospital admission. International Classification of Diseases ,10th revision. Codes I50.0, I50.2, and I50.9. |
| Yang et al. 2024^16^ | CHARLS (China Health and Retirement Longitudinal Study) | 6608  Females=3471 Males=3137 | ≥45  58.6±9.1 | 5-year | - Handgrip Strength (absolute and normalized)  - Chair stand (5 reps, seconds) | - Dynamometer (Yuejian WL-1000, Nantong, China)  - Chair stand (Chair rise speed) | - HGS cut-offs for weakness:  Females=<18 kg Males=<28 kg  - Chair-Stand thresholds:  Low=<10 sec High=≥12 sec | Cardiovascular Disease | Incident CVD (heart disease or stroke) defined via self-reported diagnosis. |
| **Zhang et al. 2023^17^** | TCLSIH (Tianjin Chronic Low-Grade Systemic Inflammation and Health Cohort) | 1258  Females=562 Males=696 | 45–85  52.5±7.9 | 4-year | Handgrip Strength (absolute and weight-adjusted) | Dynamometer (EH101, CAMRY, Guangdong, China) | - Weight-adjusted HGS Quartiles:  Females, Q1=0.24–0.36; Q4=0.46–0.66  Males, Q1=0.28–0.50; Q4=0.62–0.84 | Incident Carotid Atherosclerosis | Clinically confirmed Incident Carotid Atherosclerosis based on CIMT ≥1.0 mm or plaque. |
| **Zhang et al. 2024^18^** | CHARLS (China Health and Retirement Longitudinal Study) | 9369  Females=4708 Males=4661 | ≥45  56.69±9.0 | 3-year | Handgrip Strength (absolute) | Dynamometer (Yuejian WL-1000, Nantong, China) | - HGS Quartiles:  Females, Q1=<24.15 kg; Q4=≥38.50 kg  Males, Q1=<30.50 kg; Q4=≥47.00 kg | Cardiovascular Disease | Incident CVD (self-reported and verified heart disease or stroke). |
| **CANCER (n=6)** | | | | | | | | | |
| **Celis-Morales et al. 2018^2^** | UK Biobank | 502293  Females=271238  Males=231055 | 40-69  56.5±8.1 | 7.1-year | Handgrip Strength (absolute) | Dynamometer (*N/A*) | - Sex-age specific HGS quartiles:  Females, <56 years, Q1=<21 kg; Q2=21-25 kg; Q3=26-29 kg; Q4=>29 kg. 56-65 years, Q1=<19 kg; Q2=18-22 kg; Q3=23-26 kg; Q4=>26 kg  Males, <56 years, Q1=<36 kg; Q2=36-42 kg; Q3=43-48 kg; Q4=>48 kg. 56-65 years, Q1=<33 kg; Q2=33-38 kg; Q3=39-44 kg; Q4=>44 kg  - Change in HGS:  Per 5 kg decrement in HGS | Cancer (all cancer, colorectal, lung, breast, and prostate) | Hospital admissions, with the International Classification of Diseases, 10th revision. |
| Ho et al. 2019^4^ | UK Biobank | 356721  Females=194540  Males=162181 | 37-73  53.7±8.12 | 2-year | Handgrip Strength (absolute, and relative to height, weight, fat-free mass, BMI, fat-free mass index and fat-free mass) | Dynamometer (Jamar J00105) | Sex-age-specific quintiles:  Q1=≤21.5 kg; Q2=>21.5-27 kg; Q3=>27-33 kg; Q4=>33-41.5 kg; Q5=>41.5 kg | Cancer | Hospital admissions, with the International Classification of Diseases, 10th revision. |
| **Leong et al. 2015^8^** | Prospective Urban Rural Epidemiology (PURE) study | 139691  Females=81039  Males=58652 | 35-70 | 4-year | Handgrip Strength (absolute) | Dynamometer (Jamar dynamometer) | - Sex-specific tertiles:  *N/A*  - Change in HGS:  Per 5 kg decrement in HGS | Cancer | Hospital admission. |
| Parra-Soto et al. 2022^19^ | UK Biobank | 445552  Females=239838  Males=205714 | ±56 | 9-year | Handgrip Strength (absolute, and relative: divided by height, divided by weight, divided by BMI, and divided by body fat mass) | Dynamometer (Jamar, UK) | - Sex-specific HGS tertiles:  *N/A* | Cancer | The International Classification of Diseases, 10th revision (Hospital admissions). |
| Parra-Soto et al. 2021^20^ | UK Biobank | 445555  Females=239316  Males=205239 | ±56 | 9-year | Handgrip Strength (relative, divided by BMI) | Dynamometer (Jamar, UK) | - Sex-specific HGS quartiles:  *N/A*  - HGS change:  Per 1 SD increment in HGS | Cancer | The International Classification of Diseases, 10th revision (Hospital admissions). |
| **Weber et al. 2021^21^** | UK Biobank | 350512  Females=178340  Males=172172 | 38-73 | 7-year | Handgrip Strength (absolute) | Dynamometer (Jamar, Lafayette Instrument, USA) | - Sex-specific HGS quartiles of PA activity:  *N/A*  - HGS change:  Per 1 kg increment in HGS | Malignant melanoma | Depending on the diagnosis date, classification of cancer was conducted with the International Classification of Diseases 9th or 10th Revision (Hospital admissions). |
| **TYPE 2 DIABETES MELLITUS (n=22)** | | | | | | | | | |
| **Boonpor et al. 2021^22^** | UK Biobank | 166894  Females=93794  Males=73100 | 37-73  55.8±8.1 | 5.3-year | Handgrip Strength (absolute and relative by body weight) | Dynamometer (Jamar J00105 hydraulic hand dynamometer) | - Age-specific and sex-specific HGS quintiles:  *N/A*  - HGS change:  Per 5 kg decrement in HGS and 0.05 kg/ kg decrement in relative HGS | Type 2 Diabetes Mellitus (T2DM) | Primary care diagnosis, with the International Classification of Diseases, 10th revision. |
| **Hao et al. 2020^23^** | Chinese Health and Retirement Prospective Cohort Study (CHARLS) | 5271  Females=2810  Males=2461 | >45  Group 1: 45-55  Group 2: 55-65 | 3.7-year | Handgrip Strength (absolute and relative by BMI) | Dynamometer (NA) | - Sex-specific HGS tertiles:  Females: T1=19.9±4.8 kg; T2=26.5±3.7kg; T3=32.5±5.7 kg  Males: T1=30.3±6.6 kg; T2=38.6±5.1 kg; T3=45.8±7.8 kg | T2DM | Fasting glucose ≥7.0 mmol/L (≥126 mg/dL), glycohemoglobin (HbA1c) ≥ 6.5%, or using antidiabetic drugs. |
| **He et al. 2024^24^** | China Health and Retirement Longitudinal Study  (CHARLS) | 6033  Females=3286  Males= 2747 | 58.4 ± 8.7 | 7-year | - Handgrip Strength (and relative by body weight)  - Chair stand (5 reps, seconds) | - Dynamometer (Jamar. Sammons Preston Rolyan, Bolingbrook, IL, USA)  - Chair stand (Chair rise speed) | - Sex- specific HGS tertiles:  Males, T1=<0.60; T2=0.60–0.71; T3=≥0.71  Females, T1=<0.44; T2=0.44–0.55; T3=≥0.55  - Chair stand tertiles:  T1=<8.65 s; T2=8.65–11.45 s; T3=≥11.45 s | T2DM | Fasting glucose ≥ 126 mg/dL, glycohemoglobin (HbA1c) ≥ 6.5%, or self-reported history of diabetes. |
| **Jeon et al. 2021^25^** | Korean Genome and Epidemiology Study (KoGES) | 2699  Females=1313  Males=1386 | 40-69 | 16-year | Handgrip Strength (absolute and relative by BMI, Weight-normalized HGS, WC-normalized HGS, WHR-normalized HGS) | Dynamometer (Grip-D T.K.K.5401 and T.K.K.5102, TAKEI Science Instruments Co., Ltd, Nigata, Japan) | - Sex-specific HGS quartiles:  *N/A*  - Absolute cut-off point for Incident T2DM:  Females, HGS=21.5±3.9 kg  Males, HGS=35.0±5.6 kg  - HGS change:  Per one SD increment in HGS | T2DM | T2DM was defined as a fasting plasma glucose concentration of ≥126 mg/dL, or a postprandial 2-h glucose concentration of ≥200 mg/dL, or current treatment with oral anti-diabetic drugs or insulin. |
| Karvonen-Gutierrez et al. 2018^26^ | The Study of Women’s Health Across the Nation (SWAN) | Females=424 | 42-52  46.4±2.8 | 16-year | Handgrip Strength (relative by body weight) | Dynamometer (Baseline hydraulic hand dynamometer) | - HGS change:  Per 0.1 kg increment in HGS | T2DM | Diabetes was defined as: (1) self-reported doctor’s diagnosis of diabetes; (2) self-reported use of anti-diabetic medications (oral medications or insulin) or (3) fasting blood glucose ≥126 mg/dl or haemoglobin A1c (HbA1c) ≥6.5% |
| **Katzmarzyk et al. 2007^27^** | The Physical Activity Longitudinal Study (PALS) | 1543  Females=834  Males=709 | 18-69  Females: 37.5±12.1  Males: 36.8±12.4 | 15.5-year | Handgrip Strength (absolute) | Dynamometer (Stoelting) | *N/A* | T2DM | Questionnaire survey. |
| **Kowall et al. 2022^28^** | Survey of Health, Ageing and Retirement in Europe (SHARE) | 46119 Females=26514  Males: 19605 | ≥50  ±63.5 | 5.3-year | -Handgrip Strength (absolute)  - Chair stand (5 reps, seconds) | - Dynamometer (Hand Grip Meter 6103, Tanita)  - Chair stand (Chair rise speed) | - CST-5 Quartiles:  Q1 (fastest)=≤10.5 s;  Q2=10.6–12.5 s;  Q3=12.6–14.5 s; Q4 (slowest)=≥14.6 s  - Change in HGS:  Per 1 SD decrement in HGS (SD=8.5 kg)  - Change in Chair stand:  Per 1 SD increment in Chair stand (SD=1.3 sec) | T2DM | Doctor-diagnosed diabetes or use of diabetes medication. |
| Kunutsor et al. 2021^29^ | The Kuopio Ischemic Heart Disease (KIHD) study | 776  Females=410  Males=366 | 61-72 | 18-year | Handgrip Strength (relative by body weight) | Dynamometer (Martin Vigorimeter, Germany) | - HGS change:  Per SD increment in HGS | T2DM | Fasting plasma glucose (FPG) ≥7.0 mmol/L, a 2 h glucose tolerance test plasma glucose ≥11.1 mmol/L, or use of glucose-lowering medication according to self-report at re-examination and by record linkage to the national hospital discharge registry and to the Social Insurance Institution of Finland register for reimbursement of medicine expenses. |
| **Larsen et al. 2016^30^** | Health, Aging, and Body Composition Study | 2166  Females=1146  Males=1020 | 70-79 | 11.3-year | Handgrip Strength (absolute) | Dynamometer (Jaymar; JLW Instruments) | - HGS change:  *N/A* | T2DM | Fasting glucose levels ≥126 mg/dL and/or reporting a physician’s diagnosis of diabetes and/or use of hypoglycaemic medication. |
| **Li et al. 2016^32^** | Men Androgen Inflammation Lifestyle Environment and Stress (MAILES) Study | Males=1632 | >35 | 5-year | Handgrip Strength (relative by lean mass) | Dynamometer (Jamar) | - HGS change:  Per 5 kg increment in HGS | T2DM | Previous doctor diagnosis, diabetes medication use, fasting plasma glucose (FPG) ≥7.0 mmol/L (≥126 mg/dl), or glycated haemoglobin (HbA1c) ≥6.5% (48 mmol/mol). |
| **Li et al. 2021^33^** | Survey of Health, Ageing and Retirement in Europe (SHARE) | 66100  Females=36315  Males=29785 | >50 | 2-year | Handgrip Strength (absolute and relative by BMI) | Dynamometer (Smedley, Tokyo) | - Sex-age-specific quartiles (dominant HGS hand):  Females, HGS: Q1=<22 kg; Q2=22-27 kg: Q3=27-31 kg; Q4=≥31 kg  Males, HGS: Q1=<37 kg; Q2=37-44 kg: Q3=44-51 kg; Q4=≥51 kg | T2DM | Doctor diagnosis or high blood sugar. |
| Lee et al. 2024^31^ | Korean Genome and Epidemiology Study (KoGES) | 33326 Females= 22589  Males=10737 | 40–80  ±54.8 | 4.1-year | Handgrip Strength (absolute an relative) | Dynamometer (T.K.K. 5401, Takei Scientific) | - HGS Quartiles:  Females, Q1=≤0.85; Q4=>1.17  Males, Q1=≤1.40; Q4=>1.84 | T2DM | Diagnosed per ADA criteria (fasting plasma glucose ≥126 mg/dL or HbA1c ≥6.5%, or treatment history). |
| **Leong et al. 2015^8^** | Prospective Urban Rural Epidemiology (PURE) study | 139691  Females=81039  Males=58652 | 35-70 | 4-year | Handgrip Strength (absolute) | Dynamometer (Jamar) | - Sex-specific tertiles:  *N/A*  - Change in HGS:  Per 5 kg decrement in HGS | T2DM | Hospital admission. |
| Mahala Manda et al. 2020^34^ | The Japan Agriculture Cooperative of Ibaraki (JA Ibaraki) | 1075  Females=482  Males=593 | 20-75 | 2-year | Handgrip Strength (absolute and relative by BMI) | Dynamometer (Takei, Japan) | - Sex-age-specific relative HGS tertiles:  *N/A* | T2DM | Prediabetes: HbA1c of 5.7% to 6.4% or FPG of 110 to 125 mg/dL (Heianza et al., 2011). Type 2 diabetes mellitus: physician’s diagnosis, use of antidiabetic medication, FPG ≥ 126 mg/dL, or HbA1c ≥6.5%. |
| **Marques-Vidal et al. 2017^35^** | CoLaus (Cohorte Lausannoise) study | 2318  Females=1354  Males=964 | 50-75  ±60.2 | 10.7-year | Handgrip Strength (absolute) | Dynamometer (Baseline Hydraulic Hand Dynamometer, NY, USA) | - Cut-off points for T2DM:  HGS=35.6±10.5 kg  - HGS change:  Per 5 kg increment in HGS | T2DM | T2DM was defined as stated by the American Diabetes Association using fasting plasma glucose. |
| McGrath et al. 2017^36^ | Mexican Americans | 1903  Females=1102  Males=801 | ≥65  73.30±6.50 | 19-years | Handgrip Strength (normalized by body weight) | Dynamometer (Jamar Hydraulic Dynamometer; J.A. Preston Corporation, Clifton, NJ) | - Normalized HGS cut-off for weakness:  Females, HGS=<30 kg  Males, HGS=<46 kg | T2DM | Self-reported (doctor diagnosis). |
| **Momma et al. 2019^37^** | The Niigata Association of Occupational Health | 21802  Females=6649  Males=15153 | 20-92  ±50 | 5-year | Handgrip Strength (absolute and relative by body weight) | Dynamometer (Takei) | - Sex-specific HGS quartiles:  *N/A* | T2DM | Fasting glucose level of ≥126 mg=dL (7.0 mmol=L), HbA1c ≥48 mmol=mol (6.5%), self-reported history of previously diagnosed diabetes or current medication for diabetes. |
| **Park et al. 2024^38^** | KoGES (Korean Genome and Epidemiology Study) | 1935  Females=1102 Males=833 | 51–81  68.7±7.4 | 6.5 years | Handgrip Strength (Absolute and relative) | Dynamometer (JAMA-5030J1, SAEHAN, Korea) | - HGS tertiles:  Absolute HGS:  T1=<0.86; T2=0.86–1.12; T3=>1.12  - Relative HGS (kg/kg):  T1=<0.35; T2=0.35–0.44; T3=>0.44 | T2DM | Incident T2DM defined by fasting glucose ≥126 mg/dL, HbA1c ≥6.5%, or self-reported. |
| Qiu et al. 2023^39^ | The China Health and Retirement Longitudinal Study (CHARLS) | 3731  Females=1963  Males=1768 | ≥45 years | 4-year | - Handgrip Strength (relative by body weight)  - Chair stand (5 reps, seconds) | - Dynamometer (YuejianTM WL-1000)  - Chair stand (Chair rise speed) | - HGS tertiles:  Cumulative strength (measured at the baseline, 2013–2014, and 2015 surveys) | T2DM | Fasting plasma glucose (FPG) ≥7.0 mmol/L (126 mg/dL), random plasma glucose ≥11.1 mmol/L (200 mg/dL), or HbA1c ≥6.5% (48 mmol/mol). Medical history and the use of antidiabetic medications were additionally employed to ascertain diabetes prior to the 2015 survey. |
| Sohn et al. 2024^40^ | KLoSA (Korean Longitudinal Study of Ageing) | 22016  Females=12114  Males=9902 | ≥65  73.8±6.4 | 14-year | Relative Handgrip Strength (HGS/BMI) | Dynamometer (Tanita 6103, Japan) | - Relative HGS Tertiles:  Females, T1=0–0.68; T2=0.68–0.86; T3=0.86–2.39  Males, T1=0–1.13; T2=1.13–1.39; T3=1.39–4.30 | New-Onset T2DM | Diagnosed by doctor or treatment report via survey. |
| **Wander et al. 2011^41^** | Japanese American Community Diabetes Study | 394  *N/A* | ±52 | 10-year | Handgrip Strength (absolute) | Dynamometer (Harpenden R, England) | - Cut-off points for T2DM:  HGS=50.0±1.6 lbs | T2DM | Oral glucose tolerance test (75 g load) and defined as fasting glucose  ≥126 mg/dl and/or 2-  h glucose ≥200 mg/dl, or use of diabetes medication. |
| Zheng et al. 2022^42^ | The China Health and Retirement Longitudinal Study (CHARLS) | 4561  Females=2416  Males=2145 | ≥45  58.7±8.7 | 4-year | Handgrip strength (relative by weight) | Dynamometer (WL-1000 Mechanical Handgrip Meter; Nantong, China) | *N/A* | T2DM | Doctor diagnosis with and individual’s interview or fasting plasma glucose ≥126 mg/dL (7.0 mmol/mol), or glycated haemoglobin ≥6.5% (48 mmol/mol) from blood test reports. |
| **RESPIRATORY DISEASES (n=3)** | | | | | | | | | |
| **Celis-Morales et al. 2018^2^** | UK Biobank | 502293  Females=271238  Males=231055 | 40-69  56.5±8.1 | 7.1-year | Handgrip Strength (absolute) | Dynamometer (*N/A*) | - Sex-age specific HGS quartiles:  Females, <56 years, Q1=<21 kg; Q2=21-25 kg; Q3=26-29 kg; Q4=>29 kg. 56-65 years, Q1=<19 kg; Q2=18-22 kg; Q3=23-26 kg; Q4=>26 kg  Males, <56 years, Q1=<36 kg; Q2=36-42 kg; Q3=43-48 kg; Q4=>48 kg. 56-65 years, Q1=<33 kg; Q2=33-38 kg; Q3=39-44 kg; Q4=>44 kg  - Change in HGS:  Per 5 kg decrement in HGS | Respiratory disease, chronic obstructive pulmonary disease | Hospital admissions, with the International Classification of Diseases, 10th revision. |
| Ho et al. 2019^4^ | UK Biobank | 356721  Females=194540  Males=162181 | 37-73  53.7±8.12 | 2-year | Handgrip Strength (absolute, and relative to height, weight, fat-free mass, BMI, fat-free mass index and fat-free mass) | Dynamometer (Jamar J00105) | Sex-age-specific quintiles:  Q1=≤21.5 kg; Q2=>21.5-27 kg; Q3=>27-33 kg; Q4=>33-41.5 kg; Q5=>41.5 kg | Respiratory disease, chronic obstructive pulmonary disease | Hospital admissions, with the International Classification of Diseases, 10th revision. |
| **Leong et al. 2015^8^** | Prospective Urban Rural Epidemiology (PURE) study | 139691  Females=81039  Males=58652 | 35-70 | 4-year | Handgrip Strength (absolute) | Dynamometer (Jamar) | - Sex-specific tertiles:  *N/A*  - Change in HGS:  Per 5 kg decrement in HGS | Any respiratory disease (including COPD, asthma, tuberculosis, and pneumonia) | Hospital admission. |
| **MUSCULOSKELETAL IMPAIRMENT (n=30)** | | | | | | | | | |
| **Alajlouni et al. 2021^43^** | Osteoporotic Fractures in Men (MrOS) Study | Men=5665 | >65  73.5±5.78 | 13-year | - Handgrip Strength (absolute)  - Chair stand (5 reps, seconds) | - Dynamometer (Jamar. Sammons Preston Rolyan, Bolingbrook, IL, USA)  - Chair stand (Chair rise speed) | - Change in HGS:  Per 1 SD decrement in HGS (SD=8.5 kg)  - Change in Chair stand:  Per 1 SD increment in Chair stand (SD=1.3 sec) | Fractures | Telephone questionnaire. |
| Albrand et al. 2003^44^ | The OFELY study | Females=672 | 59.1±9.8 | 5.3-year | - Handgrip Strength (absolute)  - Chair stand (5 reps, seconds) | - Dynamometer (Vigorimeter Martin)  - Chair stand (Chair rise speed) | - HGS cut-off:  HGS=<0.60 bar  - Chair stand cut-off:  Chair stand=>8.3 sec | Fractures | Radiography. |
| Beauchamp et al. 2022^45^ | Canadian Longitudinal Study on Aging (CLSA) | 1121  Females=747  Males=374 | 75.2±5.9 | 18-months | Chair stand (5 reps, seconds) | Chair stand (Chair rise speed) | *N/A* | Falls | Self-reported. |
| **Cawthon et al. 2008^46^** | MrOS study (USA) | Males=5902 | ≥65 | 5.3-year | - Handgrip Strength (absolute)  - Chair stand (5 reps, seconds) | - Dynamometer (Jamar. Sammons Preston Rolyan, Bolingbrook, IL, USA)  - Chair stand (Chair rise speed) | - HGS quartiles:  Q1=<36 kg; Q2=≥36 to <42.0 kg; Q3=≥42.0 to <48.0 kg; Q4=≥48 kg  - Chair stand quartiles:  Q1=<9.0 sec; Q2=≥9.0 to <10.5 sec; Q3=≥10.5 to <12.6 sec; Q4=≥12.6 sec. | Hip fractures | Radiographic diagnosis. |
| Cheung et al. 2012^47^ | The Hong Kong Osteoporosis  Study | 1702  Females=820  Males=882 | >50 | 4.9-year | Handgrip Strength (absolute) and T-score (corrected by body weight) | Dynamometer (Smedley Hand Dynamometer, Stoelting Co, Wood Dale, IL) | - HGS change:  Decrement in HGS T-score | Fractures (osteoporosis risk) | Dual-energy X-ray absorptiometry. |
| Delbaere et al. 2006^48^ | *N/A* | 1259  Females=725  Males=534 | ≥60  72.1±5.5 | 1-year | - Handgrip Strength (absolute)  - Chair stand (1 rep, seconds) | - Dynamometer (Baseline, Fabrication Enterprises Inc., New York, USA)  - Chair stand (Chair rise speed) | - HGS change:  Per decrement (*N/A*) in HGS  - Chair stand change:  Per increment (*N/A*) in Chair stand | Falls | Falls calendar. |
| Finigan et al. 2008^49^ | United Kingdom | Women=367 | 50-85  64.6±9.1 | 10-year | Handgrip Strength (absolute) | - Dynamometer (Accoson, London, UK) | Weaker HGS:  *N/A* | Fractures | Radiographic diagnosis. |
| **Guo et al. 2024^50^** | China Health and Retirement Longitudinal Study (CHARLS) | 10,092  F: 5621  M: 4471 | >45  58.6 ± 9.1 years | 4-years | Handgrip Strength (absolute) | Handgrip dynamometer (YuejianTM WL-1000, Nantong, China) | Sex-specific Quintiles *(N/A)* | Hip fractures and falls | Self-reported. |
| **Hussain et al. 2024^51^** | SHARE (Survey of Health, Aging, and Retirement in Europe) | 48533  Females=26441  Males=20962 | 50–99  67.4±9.1 | 7-year | Handgrip Strength (absolute) | Dynamometer (Smedley, TTM Tokyo, 100 kg capacity) | - HGS cut-offs for weakness:  Females=<16 kg Males=<27 kg | Hip Fracture Risk | Clinically verified hip fractures through survey waves. |
| **Kamiya 2019^52^** | Japanese Population-based Osteoporosis Cohort Study | Females=1342 | >50  63.4±8.5 | 15.2-year | Handgrip Strength (absolute) | Dynamometer (TKK-5101, Japan) | - HGS tertiles:  T1=≤22.2 kg; T2=22.3-26.2 kg; T3=≥26.3 kg | Fractures (osteoporosis risk) | Radiographic diagnosis. |
| Kärkkäinen et al. 2008^53^ | The Osteoporosis Risk Factor and Prevention Study (OSTPRE) | Females=2928 | (47-56)  ±59 | 8-year | Handgrip Strength (absolute) | Dynamometer (Martin Vigorimeter, Tuttlingen, Germany) | - HGS change:  Per 10 Nm decrement in HGS | Fractures (osteoporosis risk) | Questionnaires. |
| **Leong et al. 2015^8^** | Prospective Urban Rural Epidemiology (PURE) study | 139691  Females=81039  Males=58652 | 35-70 | 4-year | Handgrip Strength (absolute) | Dynamometer (Jamar) | - Sex-specific tertiles:  *N/A*  - Change in HGS:  Per 5 kg decrement in HGS | Falls, fractures | Hospital admission. |
| Minneci et al. 2015^54^ | Insufficienza Cardiaca negli Anziani Residenti a Dicomano (ICARe Dicomano) Study (Italy) | 561  Females=323  Males=238 | ≥65  72.90±0.30 | 3-year | Handgrip Strength (absolute) | Dynamometer (*N/A*) | *N/A* | Falls | Self-reported. |
| **Muraki et al. 2013^55^** | Research on Osteoarthritis / Osteoporosis Against Disability (ROAD) | 1348  Females=896  Males=452 | ±64 | 3-year | - Handgrip Strength (absolute)  - Chair stand (5 reps, seconds) | - Dynamometer (TOEI LIGHT CO., LTD, Saitama, Japan)  - Chair stand (Chair rise speed) | - Strength performance change:  Per 5 kg increment in HGS; per 1 second increment in Chair stand | Falls | Interview. |
| Ooi et al. 2021^56^ | The Long-term Research Grant Scheme - Towards Useful Ageing (LRGS-TUA) study | 523  *Sex by age-group no reported* | 60-64 | 18-months | - Handgrip Strength (absolute)  - Chair stand (30 seconds, reps) | - Dynamometer (Jamar Plus+ Hand, Australia)  - Chair Stand (Chair rise speed) | - Cut-off points for falls:  Occasional fallers:  HGS: 20.82±7.27 kg; Chair Stand: 9.60±3.28 reps  Recurrent fallers: HGS: 19.57±6.69 kg; Chair Stand: 8.83±3.07 reps | Falls (Occasional and recurrent falls) | Interview: falls occurring within 12-months before the baseline examination. |
| **Pham et al. 2023^57^** | ASPREE (ASPirin in Reducing Events in the Elderly) | 16445  Females=9015  Males=7430 | ≥65  75.3±4.4 years | 4-years | Handgrip Strength (absolute) | Dynamometer (Jamar Plus+, Lafayette, USA) | - HGS Quintiles:  Females, T1=<20 kg; T2-T4=20–28 kg; T5=>28 kg  Males, T1=<30 kg; T2-T4=30–40 kg; T5=>40 kg | Serious Falls | Hospital presentations due to falls (clinically verified). |
| Rikkonen et al. 2012^58^ | Kuopio Osteoporosis Risk Factor and Prevention Study (OSTPRE), Filand | Females=979 | 63-75  68.1±2.4 | 20-year | Handgrip Strength (absolute) | Dynamometer  (Jamar; Sammons Preston, Bolingbrook, IL) | *N/A* | Osteoporosis | Dual X-ray absorptiometry. |
| Roongbenjawan et al. 2020^59^ | *N/A* | 73  Females=52  Males=21 | ≥65 | 6-months | Chair stand (30 sec) | Chair stand (number of repetitions) | - Chair stand cut-off:  < 11.25 rep | Fall Risk | Self-reported. |
| **Rouzi et al. 2015^60^** | Center of Excellence for Osteoporosis Research (CEOR) Study | Females=707 | >50 | 5.2-years | - Handgrip Strength (absolute)  - Chair stand (5 reps, seconds) | Dynamometer (Jamar-Plus, Sammons Preston, Bolingbrook, IL)  - Chair stand (Chair rise speed) | - Quartiles:  *N/A*  - Cut-off points:  HGS=≤13.88 kg (the lowest quartile); Chair stand=≥15.26 seconds (the lowest quartile) | Falls | Incidence asked by a doctor. |
| **Schaap et al. 2017^61^** | Longitudinal Aging Study Amsterdam (LASA; The Netherland) | 496  Females=248  Males=248 | ≥65  75.20±6.40 | 10-year | Handgrip Strength (absolute) | Dynamometer (Takei TKK 5001, Takei Scientific Instruments Co. Ltd., Tokyo, Japan) | - HGS cut-off for weakness, EWGSOP definition:  Females, HGS=<20 kg  Males, HGS=<30 kg  - HGS cut-off for weakness, FNIH definition:  Females, HGS=<26 kg  Males, HGS=<16 kg | - Falls  - Fractures | - Falls: self-reported  - Fractures: medical interview. |
| Søgaard et al. 2020^62^ | The Tromsø Study | 6893  Females=4002  Males=2891 | 50-79  Females=61.0±7.4  Males=62.9±6.5 | 15-year | Handgrip Strength (absolute) | Dynamometer (Martin Vigorimeter) | - Sex-age-specific HGS quintiles:  Females: Q1=0.46 bar; Q2=0.63 bar; Q3=0.72 bar; Q4=0.81 bar; Q5=0.98 bar  Males: Q1=0.57 bar; Q2=0.73 bar; Q3=0.83 bar; Q4=0.94 bar; Q5=1.1 bar  - HGS change:  Per 1 SD decrement in HGS | Fractures (osteoporosis risk) | X-ray archives. |
| **Stel et al. 2003^63^** | Longitudinal Aging Study Amsterdam (LASA; The Netherland) | 435  *N/A* | 55-85  78.30±6.20 | 3-year | Handgrip Strength (absolute) | Dynamometer (Takei TKK 5001, Takei Scientific Instruments Co. Ltd., Tokyo, Japan) | - HGS cut-off for weakness:  Females, HGS=<35 kgF  Males, HGS=<58 kgF | Falls | Self-reported. |
| **Stel et al. 2004^64^** | Longitudinal Aging Study Amsterdam (LASA; The Netherland) | 1477  Females=762  Males=715 | ≥65  75.80±6.6 | 3-year | Handgrip Strength (absolute) | Dynamometer (Takei TKK 5001, Takei Scien- tific Instruments Co. Ltd., Tokyo, Japan) | - HGS cut-off for weakness:  Females, HGS=<15 kgF  Males, HGS=<27 kgF | Fractures | Questionnaire. |
| Valenzuela et al. 2020^65^ | StrongAge Olten Cohort study (Switzerland) | 24  Females=19  Males=5 | ≥70  88±7 | 1-year | Handgrip Strength (absolute) | Dynamometer (Jamar Plus+, Sammons Preston Rolyan, Chicago, USA), and Chronometer | *N/A* | Falls | Personal interview. |
| Wang et al. 2024^66^ | CHARLS (China Health and Retirement Longitudinal Study) | 9627  Females=4960 Males=4667 | ≥50  63.8±8.2 | 2-year | Handgrip Strength (asymmetry: HGSmax and HGSave criteria) | Dynamometer (EH101, CAMRY, Guangdong, China) | - HGSmax Asymmetry Thresholds:  Symmetrical: 0.9–1.1 % Moderate Asymmetry: 10.1–30.0%  Severe Asymmetry: >30% | Fall Risk | Self-reported. |
| **Welmer et al. 2017^67^** | The Swedish National Study on Aging and Care in Kungsholmen (SNAC-K) | 2495  Females=1545  Males=950 | ≥60  72.00±9.80 | 3-year | - Handgrip Strength (absolute)  - Chair stand (5 reps, seconds) | - Dynamometer (Grippit)  - Chair stand (Chair rise speed) | - Cut-off points for falls:  HGS=217.0±100.2 Newtons; Chair Stand=29.1±25.8 seconds | Falls | Interview: falls occurring within 3 years before the baseline examination. |
| **Yan et al. 2024^68^** | CHARLS (China Health and Retirement Longitudinal Study) | 12043  Females=6230  Males=5813 | 45–85  58.66±9.85 | 7-year | - Handgrip Strength (absolute and normalized by body weight)  - Chair stand (5 reps, seconds) | - Dynamometer (Yuejian WL-1000, Nantong, China)  - Chair stand (Chair rise speed) | - HGS cut-offs for weakness:  Females=<18 kg; Males=<28 kg  - Chair-Stand thresholds:  Low=<10 sec; High=≥12.3 sec | Knee Osteoarthritis (KOA) | Symptomatic KOA defined as joint pain with physician-diagnosed arthritis. |
| **Zhang et al. 2013^69^** | InCHIANTI study (Italy) | 948  Females=522  Males=426 | ≥60  73.19±6.84 | 3-year | Chair stand (5 reps, seconds) | Chair stand (Chair rise speed) | STS Quintiles:  Q5, (worst performance)=inability to complete the test;  Q4=> 16.6 s; Q3=13.7–16.6 s; Q2=11.2–13.6 s; Q1=<11.2 s | Falls and fall-related fractures | Interview: falls occurring within 3 years before the baseline examination. |
| **Zhong et al. 2021^70^** | The China Health and Retirement Longitudinal Study (CHARLS) | 5958  Females=3000  Males=2958 | ≥60  67.49±6.69 | 4-year | - Handgrip Strength (absolute)  - Chair stand (5 reps, seconds) | - Dynamometer (YuejianTM WL-1000 dynamometer)  - Chair stand (Chair rise speed) | - HGS (newton/meters), quartiles: *N/A*  - STS, quintiles:  Q1=≥16.7 s; Q2=13.7-16.6 s; Q3=11.2-13.6 s; Q4=≤11.1 s; Q5=unable to perform the test | Hip fractures | Self-reported. |
| Zhou et al. 2022^71^ | CHARLS (China Health and Retirement Longitudinal Study) | 4857  Females=2190 Males=2667 | 60–102  67.16±5.78 | 3-year | - Handgrip Strength (absolute)  - Chair stand (5 reps, seconds) | - Dynamometer (Yuejian WL-1000, Nantong, China)  - Chair stand (Chair rise speed) | - HGS cut-offs:  Low=<26 kg; High=≥26 kg  - FTSST cut-offs: Pass=<12 sec; Fail=>12 sec | Fall Risk | Self-reported. |
| **DISABILITY (disability in activities of daily living, functional mobility, ambulatory status, n=31)** | | | | | | | | | |
| **Al Snih et al. 2004^72^** | Hispanic Established Populations for the Epidemiological Study of the Elderly (EPESE) | 2593  Females=1443  Males=1050 | ≥65  Females=72.3±6.2  Males=72.5±6.2 | 7-year | Handgrip Strength (absolute) | Hand-held dynamometer (Jamar Hydraulic Dynamo-meter model #5030J1- J.A. Corp) | - Sex-specific HGS quartiles:  HGS, females: Q1=<22 kg; Q2=22.01-30.00 kg; Q3=30.01-35.00 kg; Q4=≥35.01 kg  HGS, males: Q1=<14 kg; Q2=14.01-18.20 kg; Q3=18.21-22.50 kg; Q4=≥22.51 kg | ADL | Modified Katz ADL questionnaire (removing continence, and by adding grooming and ability to walk across a small room). |
| Burbank et al. 2023^73^ | National Health and Aging Trends Study (NHATS) | 4803  Females=2639  Males=2164 | ≥65  74.0±6.2 | 4-year | Chair stand (5 reps, seconds) | Chair stand (power by Alcazar et al. equation 2019) | Chair stand power, Cut-point median =231.2W (low vs. high muscle power) | Frailty | Frailty status based on 5 criteria: exhaustion (self-report), low physical activity (self-report), unintentional weight loss (self-report), low walking speed (3-meter walk), and weakness (HGS). |
| **Carrière et al. 2005^74^** | French EPIDOS study | Females=545 | ≥75  ±79 | 7-year | - Handgrip Strength (absolute)  - Chair stand (5 reps, seconds) | - Dynamometer (*N/A*)  - Chair stand (Chair rise speed) | - HGS tertiles:  T1=<47 kPa; T2=47-61 kPa; T3=>61 kPa  - Chair stand tertiles:  T1=<9.6 sec; T2=9.6-13 sec; T3=>13 sec | ADL | ADL questionnaire. |
| **Cesari et al. 2009^75^** | Health, Aging and Body Composition (Health ABC) Study | 3024  Females=1560  Males=1464 | 70-79  73.6±2.9 | 6.9-year | Chair stand (5 reps, seconds) | Chair stand (Chair rise speed) | Chair stand, cut-off point: High-risk group ≥17.1 seconds | - Persistent lower extremity limitation  - Persistent severe lower extremity limitation | Hospital records:  - Two consecutive semi-annual reports of having any difficulty walking one-quarter of a mile or climbing up 10 steps without resting.  - Two consecutive semi-annual reports of having a lot of difficulty or not being able to walk one-quarter of a mile or to climb up 10 steps without resting. |
| Chan et al. 2014^76^ | Integrated Systemic Care for Older People (ISCOPE) study | 764  Females=521  Males=243 | ≥75  ±83 | 1-year | Handgrip Strength (absolute) | Dynamometer (Jamar. Sammons Preston Inc., Bolingbrook, IL) | - Cut-off point for weakness:  Females, HGS=<18 kg  Males, HGS=<30 kg  - HGS change:  Per unit decrement in HGS | - ADL  - Basic ADL | The Groningen Activities Restriction Scale. |
| **Coelho-Júnior et al. 2024^77^** | Aging and Longevity Study in the Sirente Geographic Area (ilSIRENTE) | 255  F:161  M:94 | 84.2 ± 5.1 yrs | 2-year | - Handgrip Strength (absolute)  - Chair stand (5 reps, seconds) | - Hydraulic dynamometer (North Coast Medical, Inc., Morgan Hill, CA, USA)  - Chair stand (Chair rise speed) | ROC curve analysis was performed to identify cutoff points | Incident disability and Falls | Incapacity to independently perform one or more ADLs;  Fall history and incident falls. |
| **Dai et al. 2023^78^** | China Health and Retirement Longitudinal Study (CHARLS) | 4,902  F: 3,020  M: 1,882 | < 45 years  60.24 ± 9.31yrs | 4-years | Handgrip Strength (absolute) | Isometric dynamometer (YuejianTM WL-1000, Nantong, China) | Sex-specific points from Asian Working Group for Sarcopenia (AWGS):  Female, Low=<18 kg  Male, Low=<28 kg | ADL disability | Lack of complete independence in any BADL/IADL item. |
| **den Ouden et al. 2013^79^** | Preservation of Function in ELderly (PROFIEL) study | 625  Females=306  Males=319 | 40-80  62.3±8.9 | 10-year | Handgrip Strength (absolute) | Dynamometer (Jamar, Lafayette Instrument, USA) | - HGS change:  Per 10 kg increment in HGS | - ADL  - Impaired physical ability | Katz ADL questionnaire, with a score of 15 representing dependencies in all ADL (8 functions). |
| **Dodds et al. 2018^80^** | Medical Research Council National Survey of Health and Development (NSHD) | 1885  Females=984  Males=901 | 53 | 16-year | - Handgrip Strength (absolute)  - Chair stand (10 reps, seconds) | - Dynamometer (electronic dynamometer, *NA*)  - Chair stand (Chair rise speed) | - Sex-specific HGS quintiles:  HGS, females: Q1=≥34.1 kg; Q5=≤21.0 kg  HGS, males: Q1=≥57.4 kg; Q5=≤38.1 kg  Chair stand, females: Q1=≥0.63 rise/second; Q5=≤0.37 rise/second  Chair stand, males: Q1=≥0.67 rise/second; Q5=≤0.37 rise/second | Disability | Office of Population Censuses and Surveys (OPCS) of Disability in Great Britain. |
| Giampaoli et al. 1999^81^ | FINE Study (Finland, Italy, Netherlands Elderly) | Males=427 | 71-91 | 4-year | Handgrip Strength (absolute) | Dynamometer (Martin dynamometer) | - HGS quartiles:  Q1=20-68 kPa; Q2=70-78 kPa; Q3=80-96 kPa; Q4=98-150 kPa | - ADL  - IADL | World Health Organization 14-item scale. |
| Gill et al. 1995^82^ | Project Safety cohort (USA) | 563  Females=415  Males=148 | ≥72  79.1 ± 4.7 | 1-year | Chair stand (3 reps, seconds) | Chair stand (Chair rise speed) | - Chair stand quartiles:  Q1=2.3-5.4 sec; Q2=5.5-6.8 sec; Q3=6.9-8.7 sec; Q4=8.8-30 sec. | ADL | Modified Katz instrument. |
| Gonzalez-Bautista et al. 2022^83^ | Multidomain Alzheimer Preventive Trial (MAPT), France | 1573  Females=1019  Males=554 | ≥70 | 4.8-year | Chair stand (5 reps, seconds) | Chair stand (Chair rise speed) | - Chair stand age-specific cut-off points:  70-79 years=14 sec; ≥80=16 sec | ADL | Katz scale. |
| **Huang et al. 2024^85^** | China Health and Retirement Longitudinal Study (CHARLS) | 3768  Females=1727  Males=2041 | 67.69±6.69  67.33±6.02 | 7-year | - Handgrip Strength (absolute)  - Chair stand (5 reps, seconds) | Mechanical dynamometer (N/A)  Chair stand (Chair rise speed) | - Age-specific and sex-specific HGS quintiles and unable group:  *N/A*  - Age-specific and sex-specific Chair stand quintiles and unable group:  *N/A* | Activities of Daily Living (ADL) | Katz ADL questionnaire. |
| Hicks et al. 2012^84^ | Invecchiare in Chianti (InCHIANTI) study | 934  Females=515  Males=419 | ≥65 | 3-year | Handgrip Strength (absolute) | Dynamometer (Baseline. Smith & Nephew, Milan, Italy) | - Cut-off point for mobility decline:  HGS=<39 kg | Mobility decline | Self-reported: any difficulty walking 1 km or climbing a flight of stairs. |
| **Ishizaki et al. 1999^86^** | Longitudinal Interdisciplinary Study on Aging, Japan | 583  Females=326  Males=257 | 65-89  70.9±4.9 | 3-year | Handgrip Strength (absolute) | Dynamometer (*N/A*) | - HGS change:  Per 1 kg decrement in HGS | - Basic ADL  - ADL | Basic ADL questionnaire, Five item subscale of Instrumental Self-Maintenance of the Tokyo Metropolitan Institute of Gerontology Index for Competence. |
| **Jung et al. 2016^87^** | Japan | Females=283 | 65-87  72.2±5.0 | 2-year | Handgrip Strength (absolute) | Dynamometer (GRIP-D, T.K.K. 5401; Takei Scientific Instruments, Tokyo, Japan) | - HGS cut-off points:  Weak=≤19.6 kg; Strong=>19.6 kg | Mobility limitation | Self-reported questionnaire. |
| **Legrand et al. 2014^88^** | BELFRAIL study (BFC80+), Belgium | 560  Females=351  Males=209 | ≥80  84.7±3.7 | 2.8-year | Handgrip Strength (absolute) | Dynamometer (Jamar Plus, Sammons Preston Rolyan, Bolingbrook, IL) | - Sex-specific HGS tertiles:  HGS, females: T1=<15.0 kg; T2=15.1-20.0 kg; T3=>20.1 kg  HGS, males: T1=<25.3 kg; T2=25.4-33.2 kg; T3=>33.3 kg | ADL | Edwards-Nunnally index. |
| **Lopez-Teros et al. 2022^89^** | Mexican Study of Nutritional and Psychosocial Markers of Frailty among Community-Dwelling Elderly | 133  Females=71  Males=62 | ≥70  75.5±4.7 | 1-year | Handgrip Strength (absolute) | Dynamometer (Takei Ltd., Tokyo, Japan) | *N/A* | ADL | ADL questionnaire. |
| **Marincolo et al. 2024^90^** | FIBRA (Frailty in Older Brazilians Study) | 390  Females=261  Males=129 | ≥65  71.7±5.07 | 9-year | Handgrip Strength (absolute) | Dynamometer (Jamar, Lafayette Instruments, USA) | - HGS cut-offs for weakness:  Females=<16 kg  Males=<27 kg | Dependence in basic ADLs | Transition from independence to dependence in ≥1 ADL (Katz Index). |
| Minneci et al. 2015^54^ | Insufficienza Cardiaca negli Anziani Residentia Dicomano (ICARe Dicomano) Study (Italy) | 561  Females=323  Males=238 | ≥65  72.90±0.30 | 3-year | Handgrip Strength (absolute) | Dynamometer (*N/A*) | *N/A* | Disability in ADL | Number of tasks in which the participant reported complete inability or need for help. |
| **Morera et al. 2023^91^** | Survey of Health, Ageing and Retirement in Europe (SHARE) | 70820  Females=38448  Males=32372 | ≥50  61.0±7.7 | 3.8-year | Handgrip Strength (absolute) | Dynamometer (Smedley, S Dynamometer, TTM, Tokyo, 100 kg) | - Cut-off point for weakness:  Females, HGS=<16 kg  Males, HGS=<27 kg | Work limitations | Question designed by SHARE “Do you have any health problem or disability that limits the kind or amount of paid work you can do?”. |
| **Okabe et al. 2017^92^** | Hizen-Oshima Study | Females=264 | >40 | 9-year | - Handgrip Strength (absolute)  - Chair stand (5 reps, seconds) | - Dynamometer (Jamar, Lafayette Instrument, USA)  - Chair stand (Chair rise speed) | - Age-specific HGS change:  40-64 years and ≥65 year-groups lower HGS | ADL | Specific ADL questionnaire survey. ADL disability was defined as difficulty carrying out three or more ADL. |
| Peterson et al. 2021^93^ | The Health and Retirement Study (HRS) | 12618  Females=7652  Males=4966 | >50 | 8-year | Handgrip Strength (relative by BMI) | Dynamometer (Smedley spring-type, Scandidact, Odder, Denmark) | - HGS change:  Per each 0.05-unit decrement in HGS | ADL | Specific ADL questionnaire survey (6 self-care activities). Presence of 2 or more limitations to any ADL (i.e., ADL summary score ≥ 2) was considered a physical disability. |
| **Rantanen et al. 1999^94^** | The Honolulu Heart Program and the Honolulu-Asia Aging Study | Males=3218 | 45-68  54.0±5.5 | 25.3-year | Handgrip Strength (absolute) | Dynamometer (Smedley) | - HGS tertiles:  T1=37.0 kg; T2=39.2 kg; T3=42.0 kg | Functional limitations | Walking speed ≤0.4 and inability to from chair. |
| **Seidel et al 2011^95^** | Survey of Health, Ageing and Retirement in Europe (SHARE) | 6841  Females=3570  Males=3271 | ≥65  72±6 | 2-year | Handgrip Strength (absolute) | Dynamometer (*N/A*) | - HGS tertiles:  T1=<25.0 kg; T2=26.0-36.0 kg; T3=>37.1 kg | ADL | ADL questionnaire. |
| Shinkai et al. 2003^96^ | Tokyo Metropolitan Institute of Gerontology Longitudinal Interdisciplinary Study on Aging (TMIG-LISA) | 601  Females=337  Males=264 | ≥65  70.9±4.9 | 6-year | Handgrip Strength (absolute) | Dynamometer (*N/A*) | - HGS change:  Per a decrement by a HGS quartile | - ADL  - Basic BADL | ADL questionnaire. |
| Sirola et al. 2010^97^ | The prospective Kuopio Osteoporosis Risk Factor and Prevention (OSTPRE) study | Females=1620 | 53–66 years | 10-year | Handgrip Strength (absolute) | Dynamometer ((Martin Vigorimeter, Germany) | *N/A* | Ambulatory status | Specific interview about health and ambulatory status. |
| **Song et al. 2024^98^** | CHARLS (China Health and Retirement Longitudinal Study) | 7540  Females=3763  Males=3777 | ≥45  ≥45 | 4-year | - Handgrip Strength (absolute)  - Handgrip Strength Asymmetry | Dynamometer (Yuejian WL-1000, China) | - HGS cut-offs for weakness:  Females=<18 kg Males=<28 kg  - HGS asymmetry: <0.90 or >1.10 ratio | Functional Disability | ADL/IADL disability and functional dependency. |
| **Stessman et al. 2017^99^** | Jerusalem Longitudinal Study | 3015  Females=1214  Males=1801 | 70-90 | 20-year | Handgrip Strength (absolute) | Dynamometer (5001 Grip-A; Takei, Niigata City, Japan) | - Sex-age specific HGS quartiles:  Q4 the lowest | Disability in ADL | Defined as difficulty or dependence on another person in performing one or more of six ADL (transferring, dressing, bathing, using the toilet, eating, remaining continent). |
| **Taekema et al. 2010^100^** | Leiden 85-plus Study (The Netherlands) | 555  Females=361  Males=194 | 85 | 2-year | Handgrip strength (absolute) | Dynamometer (Jamar, Sammons Preston Inc., Boling- brook, IL) | - Sex-age-specific HGS tertiles:  Females, T1=<21-32 kg; T2=17-20 kg; T3=1-16 kg.  Males, T1=34-54 kg; T2=20-33 kg; T3=10-27 kg. | Functional limitation in ADL | Groningen Activity Restriction Scale. |
| **Zhang et al. 2013^69^** | InCHIANTI study (Italy) | 948  Females=522  Males=426 | ≥60  73.19±6.84 | 3-year | Chair stand (5 reps, seconds) | Chair stand (Chair rise speed) | STS Quintiles:  Q5, (worst performance)=inability to complete the test;  Q4=> 16.6 s; Q3=13.7–16.6 s; Q2=11.2–13.6 s; Q1=<11.2 s | ADL | Questionnaires: to report the number of ADL (including bathing, dressing, eating, getting into and out of bed or chair, walking across a room, and using the toilet) for which they needed help. |
| Zhang et al. 2022^101^ | The China Health and Retirement Longitudinal Study (CHARLS) | 2192  Females=1300  Males=892 | 60-64 | 4-year | - Handgrip Strength (absolute)  - Chair stand (5 reps, seconds) | - Dynamometer (*N/A*)  - Chair stand (Chair rise speed) | *N/A* | ADL | Katz ADL questionnaire, with having ADL disability if they reported needing any help in at least one of these ADL items. |
| **ANXIETY (diagnosis or moderate to severe symptoms, n=3)** | | | | | | | | | |
| **Cabanas-Sánchez et al. 2022^102^** | UK Biobank | 162167  Females=89567  Males=72600 | 38-70 | 10-year | Handgrip Strength (absolute) | Dynamometer (Jamar J00105) | - Sex-age-specific tertiles:  *N/A*  - Cut-off point for weakness:  Females, HGS=<16 kg  Males, HGS=<26 kg  - HGS change:  Per 5 kg decrement in HGS | Anxiety | National Institute for Health and Care Excellence, International Classification of Diseases, 10th revision. Primary care and hospital admission records. |
| **Carvalho 2019^103^** | The Irish Longitudinal Study on Aging (TILDA), Ireland | 5271  Females=2693  Males=2578 | ≥50  63.2±9.0 | 2-year | Handgrip strength (absolute) | Dynamometer (Baseline. Fabrication Enterprises Inc, White Plains, NY) | - HGS cut-off:  Females, HGS=<20 kg  Males, HGS=<30 kg | Incidence and Persistent Anxiety symptoms | Hospital Anxiety and Depression Scale. |
| **Gordon et al. 2019^104^** | The Irish Longitudinal Study on Ageing (TILDA) | 3952 | ≥50 | 2-years | Handgrip strength (absolute) | Dynamometer (Fabrication Enterprises Inc, White Plains, NY) | - Sex-specific HGS tertiles:  Females, T1=0–17.5kg; T2=18.0–22kg; T3= 22.5–38.5 kg  Males, T1=0.0–29.5kg; T2=30.0–37.0kg; T3=37.5–65.0 kg | Incidence and Persistent of Generalized Anxiety Disorder | Penn State Worry Questionnaire (PSWQ) and Composite International Diagnostic Interview – Short Form (CIDI-SF). |
| **DEPRESSION (diagnosis or mild to severe symptoms, n=17)** | | | | | | | | | |
| **Bao et al. 2022^105^** | The China Health and Retirement Longitudinal Study (CHARLS) | 5228  Females=2519  Males=2709 | >45  57.58±8.13 | 7-year | - Handgrip Strength (relative by BMI)  - Chair stand (5 reps, seconds) | - Dynamometer (Yuejian WL-1000, Nantong Yuejian Physical Measurement Equipment Co., LTD)  - Chair stand (Chair rise speed) | - Quartiles:  - HGS, Q1=the weakest (*N/A*)  - Chair Stand, Q1=the strongest (*N/A*) | Depression | 10-item Center for Epidemiological Studies-Depression Scale. |
| **Cabanas-Sánchez et al. 2022^102^** | UK Biobank | 162167  Females=89567  Males=72600 | 38-70 | 10-year | Handgrip Strength (absolute) | Dynamometer (Jamar J00105) | - Sex-age-specific tertiles:  *N/A*  - Cut-off point for weakness:  Females, HGS=<16 kg  Males, HGS=<26 kg  - HGS change:  Per 5 kg decrement in HGS | Depression | National Institute for Health and Care Excellence, International Classification of Diseases, 10th revision. Primary care and hospital admission records. |
| **Carvalho 2019^103^** | The Irish Longitudinal Study on Aging (TILDA), Ireland | 5271  Females=2693  Males=2578 | ≥50  63.2±9.0 | 2-year | Handgrip strength (absolute) | Dynamometer (Baseline. Fabrication Enterprises Inc, White Plains, NY) | - HGS cut-off:  Females, HGS=<20 kg  Males, HGS=<30 kg | Incidence and Persistent Depressive | 20-item Center for Epidemiologic Studies-Depression Scale. |
| Chan et al. 2024^106^ | The Sydney Memory and Ageing Study (MAS) | 553 | 70–90 | 6-year | - Handgrip Strength (absolute)  - Chair stand (5 reps, seconds) | -Dynamometer (Jamar, Lafayette Instrument Company, USA)  - Chair stand (Chair rise speed) | *N/A* | Depression | 15-item Geriatric Depression Scale (GDS-15). |
| **Gu et al. 2023^107^** | UK Biobank | 345621  Females=187611  Males=158010 | >50 years  56.69 ± 7.90 yrs | 8-years | Handgrip Strength adjusted by BMI | JamarJ00105hydraulichand dynamometer | BMI-specific tertiles *(N/A)* | Serious mood disorder | Hospital inpatient records: One or more episodes of depression, or Reduced pleasure ≥2 weeks with irritability, fatigue, sleep issues, weight changes, guilt, or suicidal thoughts. |
| **Hamer, Batty and Kivimaki 2015^108^** | The English Longitudinal Study of Ageing | 3862  Females=2083  Males=1779 | 64.6±8.3 | 6-year | Handgrip strength (absolute) | Dynamometer (Stoelting Co, IL, USA) | - HGS cut-off:  Females, HGS=<19.6 kg  Males, HGS=<35.3 kg  - Change in HGS:  Per 1 SD decrement in HGS | Depressive symptoms | 8-item Center for Epidemiologic Studies-Depression Scale. |
| Lian et al. 2021^109^ | The China Health and Retirement Longitudinal Study (CHARLS) | 13208  Females=7089  Males=6119 | >45 | 4-year | Handgrip Strength (absolute) | Dynamometer (Yuejian WL-1000; Nantong, Jiangsu, China) | Sex-age-specific HGS quartiles:  *N/A* | Depressive Symptoms | 10-item Center for Epidemiological Studies-Depression Scale. |
| **López-Bueno et al. 2023^110^** | SHARE (24 countries and Israel) | 115601  Females=62743  Males=52858 | ≥50  64.3±9.9 | 7.3-year | Handgrip Strength (absolute) | Dynamometer (Smedley TTM Tokyo) | - HGS Tertiles:  Females, T=<21 kg; T2=21–26 kg; T3=≥26 kg.  Males, T1=<36 kg T2=36–43 kg; T3=≥43 kg | Risk of depression | EURO-D 12-item scale (cutoff ≥4 symptoms). |
| **Luo et al. 2022^111^** | HRS (Health and Retirement Study) | 17713  Females=9964 Males=7749 | ≥50  64.3±10.0 | 4.2 years | Handgrip Strength (absolute) | Dynamometer (Smedley spring-type, USA) | - Weakness cut-offs (Fried criteria):  Age-adjusted HGS thresholds *(N/A)* | Depression | CES-D. |
| **McDowell, Gordon and Herring 2018^112^** | Irish Longitudinal Study on Ageing (TILDA) | 4505  Females=1961  Males=2544 | >50 | 2-year | Handgrip Strength (absolute) | Dynamometer (Baseline, NY) | Sex-specific tertiles:  Females, T1=0.0–17.5 kg; T2=18.0–21.5 kg; T3=22.0–38.5 kg  Males, T1=0.0–29.5 kg,; T2=30.0–37.0 kg; T3=37.5–65.0 kg | Depressive Symptoms | Center for Epidemiological Studies Depression Scale. |
| Mendorf et al. 2023^113^ | SHARE (Survey of Health, Aging, and Retirement in Europe) | 39572  Females=21485  Males=18087 | ≥50  65.9±10.3 | 7.3-year | Handgrip Strength (absolute) | Dynamometer (Smedley TTM Tokyo) | - HGS cut-off:  Females=<16 kg  Males=<27 kg | Depressive Symptoms | EURO-D scale (score ≥4 indicates depression). |
| **Stessman et al. 2017^99^** | Jerusalem Longitudinal Study | 3015  Females=1214  Males=1801 | 70-90 | 20-year | Handgrip Strength (absolute) | Dynamometer (5001 Grip-A; Takei, Niigata City, Japan) | - Sex-age specific HGS quartiles:  Q4 the lowest | Depression | Brief Symptom Inventory. |
| Song et al. 2024^114^ | China Health and Retirement Longitudinal Study (CHARLS) | 8700  Females=4230  Males= 4470 | 59.2 (9.1) | 3-year | Handgrip Strength (absolute) | - Dynamometer (Yuejian WL-1000, Nantong, China) | - Groups HGS:  (1) normal and symmetric HGS, (2) asymmetry only, (3) weakness only, and (4) both weakness and asymmetry.  - Sex-specific HGS weakness:  Females=*<*18 kg  Males*=<*28 kg  - HGS asymmetry (Ratio (kg)) = maximal nondominant HGS / maximal dominant HGS) = *<* 0.90 or *>*1.10 (10 % rule) and *<*0.80 or *>*1.20 (20 % rule) | Depression | The 10-item Center for Epidemiological Studies-Depression (CESD-10) Scale. |
| **Taekema et al. 2010^100^** | Leiden 85-plus Study (The Netherlands) | 555  Females=361  Males=194 | 85 | 2-year | Handgrip strength (absolute) | Dynamometer (Jamar, Sammons Preston Inc., Boling- brook, IL) | - Sex-age-specific HGS tertiles:  Females, T1=<21-32 kg; T2=17-20 kg; T3=1-16 kg.  Males, T1=34-54 kg; T2=20-33 kg; T3=10-27 kg. | Depression | 15-items Geriatric Depression Scale. |
| **Veronese et al. 2017^115^** | Progetto Veneto Anziani study (Italy) | 3099  Females=1854  Males=1245 | ≥65 | 4-year | - Handgrip Strength (absolute)  - Chair stand (5 reps, seconds) | - Dynamometer (Jamar. Sammons Preston INC, Bolingbrook, IL, USA)  - Chair stand (Chair rise speed) | Sex-age specific tertiles:  T1 the lowest | Depression | 15-item Geriatric Depression Scale. |
| **Zhao et al. 2020^116^** | The China Health and Retirement Longitudinal Study (CHARLS) | 8470  *N/A* | ±58 | 3.75-year | Handgrip Strength (absolute and relative by weight) | Dynamometer (*N/A*) | HGS quartiles:  *N/A* | Depressive Symptoms | Center for Epidemiological Studies Depression Scale. |
| Zheng et al. 2022^117^ | SHARE (Survey of Health, Ageing, and Retirement in Europe) | 14098  Females=7517 Males=6581 | ≥50  63.5±9.8 | 9.25 years | Handgrip Strength (absolute) | Dynamometer (Smedley Handheld, 100 kg) | - HGS tertiles:  T1=<26.0 kg; T2=26.0–35.2 kg; T3=>35.2 kg | Depressive Symptoms | Depression via EURO-D Scale (score >3). |
| **HEALTH-RELATED QUALITY OF LIFE (n=4)** | | | | | | | | | |
| Balogun et al. 2019^118^ | The Tasmanian Older Adult Cohort (TASOAC) study | 818  Females=511  Males=307 | >50 | 10-year | Handgrip Strength (absolute) | Dynamometer (North Coast™ bulb dynamometer; adult 0–30 psi, model no. 70154) | - Sex-specific distribution:  The lowest 20% for HGS at baseline =low HGS | Health-related quality of life | The Assessment of Quality of life questionnaire. |
| Chan et al. 2014^76^ | Integrated Systemic Care for Older People (ISCOPE) study | 764  Females=521  Males=243 | ≥75  ±83 | 1-year | Handgrip Strength (absolute) | Dynamometer (Jamar. Sammons Preston Inc., Bolingbrook, IL) | - Cut-off point for weakness:  Females, HGS=<18 kg  Males, HGS=<30 kg  - HGS change:  Per unit decrement in HGS | Health-related quality of life | - EQ5D+c questionnaire.  - Visual Analogue Scale. |
| Gomez-Bruton et al. 2021^119^ | EXERNET Study (Exernet Elder 3.0) | 617  Females=460  Males=157 | ≥65 | 8-year | Chair stand (30 seconds) | Chair stand (Chair rise repetitions) | *N/A* | Health-related quality of life | EuroQol Research Foundation questionnaire, plus visual analogy scale. |
| Gum et al. 2018^120^ | Israeli component of the Survey of Health Ageing and Retirement in Europe (SHARE) | 344  F: 193  M: 151 | ≥65  71.67±5.47 | 4-years | Handgrip Strength (absolute) | Dynamometer (Smedley, S Dynamometer, TTM, Tokyo | *N/A* | Health-related quality of life | Control, Autonomy, Self-Realization, and Pleasure (CASP-12). |
| **COGNITIVE DECLINE (n=27)** | | | | | | | | | |
| **Alfaro-Acha et al. 2006^121^** | Hispanic Established Populations for the Epidemiological Study of the Elderly (EPESE) | 2160  Females=1242  Males=918 | ≥65  71.9±5.9 | 7-year | Handgrip Strength (absolute) | Hand-held dynamometer (Jaymar Hydraulic Dynamo-meter model #5030J1- J.A. Corp) | - Sex-specific HGS quartiles:  HGS, females: Q1=<22 kg; Q2=22.01-30.00 kg; Q3=30.01-35.00 kg; Q4=≥35.01 kg  HGS, males: Q1=<14 kg; Q2=14.01-18.20 kg; Q3=18.21-22.50 kg; Q4=≥22.51 kg | Cognitive function | MMSE |
| Auyeung et al. 2011^122^ | School of Public Health of the Chinese university of Hong Kong (China) | 2737  Females=1223  Males=1514 | ≥65  71.6±4.6 | 4-year | - Handgrip Strength (absolute)  - Chair stand (5 reps, seconds) | - Dynamometer (Jamar. Sammons Preston INC, Bolingbrook, IL, USA)  - Chair stand (Chair rise speed) | - Change in HGS:  Per 1 SD decrement in HGS  Females, SD=4.12 kg  Males, SD=6.14 kg  - Change in Chair stand:  Per 1 SD increment in Chair stand  Females, SD=4.57 sec  Males, SD=3.69 sec | Cognitive decline | MMSE. |
| Boyle et al. 2009^123^ | Rush Memory and Aging Project (USA) | 970  Females=729  Males=241 | 54-100  80.3±7.5 | 3.6-year | Handgrip Strength (absolute) | Hydraulic hand and pinch dynamometer (Jamar; Lafayette Instrument Co USA) | *N/A* | - Mild cognitive impairment  - Alzheimer disease | - MMSE  - NINCDS-ADRDA diagnostic criteria. |
| **Buchman et al. 2007^124^** | Religious Orders Study (USA) | 877  Females=363  Males=514 | ±75 | 6-year | Handgrip Strength (absolute) | Dynamometer (Lafayette Instruments, Lafayette, Ind., USA) | - HGS change:  Slope estimate from the ordinary least squares regression model (1-lb increment) | Alzheimer disease | - MMSE  - NINCDS-ADRDA diagnostic criteria. |
| Chen et al. 2022^125^ | China Health and Retirement Longitudinal Study (CHARLS) | 4925  Females=2446  Males=2479 | ≥60  68.1 ± 6.68 years | 4-years | Handgrip Strength (absolute) | Mechanical dynamometer (YuejianTM WL-1000, Nantong, China) | - Sex-specific HGS low and high categories:  Females, low=<18kg  Males, low=<28kg | Neurodegenerative disorders | Physician-diagnosed through self-reported questionnaires. |
| **Chou et al. 2019^126^** | National Institute for Longevity Science –Longitudinal Study of Aging (NILS-LSA) | 1096  Females=538  Males=558 | ≥60  69.4 ± 5.8 | 10-year | Handgrip Strength (absolute) | Dynamometer (Takei Co., Niigata, Japan) | - Sex-specific HGS quintiles:  HGS, females: Q1=≤18.50 kg; Q2=18.51-20.90 kg; Q3=20.91-23.10 kg; Q4=23.11-25.70; Q5=>25.70 kg  HGS, males: Q1=≤30.70 kg; Q2=30.71-33.90 kg; Q3=33.91-37.40 kg; Q4=37.41-41.25; Q5=>41.25 kg | Cognitive decline | MMSE, Digit Symbol Substitution Test. |
| **Feng et al. 2023^127^** | Cohort from Fuxin Mongolia Liaoning Province, China | 743  F: 530  M: 213 | Aged 35- 85  55.9 ± 9.6 years | 2-years | Handgrip Strength (absolute) | Dynamome- ter (Jamar Plus+, Patterson Medical, USA) | *N/A* | Mild cognitive impairment | Montreal Cognitive Assess- ment-Basic (MoCA-BC). |
| **Heward et al. 2018^128^** | Identification and Intervention for Dementia in Elderly Africans (IDEA) study. Tanzania | 327  Females=186  Males=141 | ≥65 | 2-year | Handgrip Strength (absolute) | Dynamometer (Jamar hydraulic hand dynamometer. Model J000105, Lafayette Instruments, Lafayette, IN, USA) | - Sex and age-specific change in HGS:  Above or below the median normative value (Leong *et al.*, 2016). | Cognitive decline | IDEA cognitive screen. |
| **Jeong and Kim 2018^129^** | The Korean Longitudinal Study of Ageing (KLoSA) | 6435  Females=3215  Males=3220 | >45 | 6-year | Handgrip Strength (absolute) | Dynamometer (*N/A*) | - Cut-off points:  Females: <14.5 kg (low), 14.5- <17.5 kg (normal-low), 17.5- <20.0 kg (normal-high), and ≥20.0 kg (high)  Males: <25.0 kg (low), 25.0- <29.0 kg (normal-low), 29.0- <32.5 kg (normal-high), and ≥32.5 kg (high) | Cognitive function | K-MMSE. |
| Jeong et al. 2018^130^ | Korean Longitudinal Study of Aging (KLoSA) | Females=544 | ≥65 | 8-year | Handgrip Strength (absolute) | Dynamometer (Model number: NO6103, Manufacturer: TANITA, Japan) | - HGS cut-off points:  Weak=≤18 kgF; Strong=>18.5 kgF  - HGS tertiles:  T1=4.5-17.5 kgF; T2=18.0-20.5 kgF; T3=20.8-27.5 kgF | Cognitive impairment | K-MMSE. |
| Jian et al. 2022^131^ | UK Biobank | 3152–40784  Females=1513-19576  Males=1639-21208 | 55.25±7.54  40–70 | 9-year | Handgrip Strength (absolute) | Dynamometer (Jamar J00105) | Sex-specific HGS as continuous variable | 15 mental health behavioral phenotypes  Grey matter volume | Developed for the UK Biobank and from commonly used tests  Magnetic resonance imaging |
| **Kang et al. 2021^132^** | Korean Longitudinal Study of Aging (KLoSA) | 727  Females=330  Males=397 | ≥65  69.3±3.7 | 12-year | Handgrip Strength (absolute) | Dynamometer (TANITA No. 6103, Tokyo, Japan) | Change in HGS:  Per kg decrement in HGS | Cognitive decline | K-MMSE. |
| Kim 2019^133^ | The Korean Longitudinal Study of Ageing (KLoSA) | 9279  Females=5148  Males=4131 | >45 | 10-year | Handgrip Strength (absolute) | Dynamometer (Hand Grip Meter 6103, Tanita, Tokyo, Japan) | - HGS quintiles:  *N/A* (SAS Rank function) | Cognitive function | MMSE. |
| Kim and Kim 2022^134^ | The Korean Longitudinal Study of Ageing (KLoSA) | 9621  Females=5402  Males=4219 | >45 | 10-year | Handgrip Strength (absolute) | Dynamometer Dynamometer (Hand Grip Meter 6103, Tanita, Tokyo, Japan) | - Sex-specific HGS quartiles:  Females: Q1=<14.5 kg; Q2=14.5-17.5 kg; Q3=17.5-20.0 kg; Q4=>20.0 kg  Males: Q1=<25.0 kg; Q2=25.0-29.0 kg; Q3=29.0-32.5 kg; Q4=>32.5 kg | Cognitive function | MMSE. |
| Kim et al. 2019^135^ | The Korean Longitudinal Study of Ageing (KLoSA) | 2378  Females=1138  Males=1240 | ≥65  71.1 ± 5.0 | 8-year | Handgrip Strength (absolute) | Dynamometer ((TANITA Hand Grip Meter Blue 6103; Tanita Co., Tokyo, Japan) | *N/A* | Cognitive function | K-MMSE. |
| Kim et al. 2019b^136^ | The Korean Longitudinal Study of Ageing (KLoSA) | 5995  Females=3268  Males=2727 | >45 | 8-year | Handgrip Strength (absolute) | Dynamometer (Hand Grip Meter 6103, Tanita, Tokyo, Japan) | - HGS quartiles:  Q1=0-18.8 kg; Q2=18.9-23.4 kg; Q3=23.5-30.9 kg; Q4=31.0-60.3 kg | Cognitive function | MMSE. |
| **McGrath et al. 2019a^137^** | Health and Retirement Study (HRS) | 14775  Females=8554  Males=6221 | >50  64.1±9.5 | 2.1-year | Handgrip Strength (absolute) | Dynamometer (Smedley) | - Cut-off points for weakness:  Females=<16 kg  Males=26 kg | Cognitive function:  - Any cognitive impairment (CI)  - Severe CI  - Poorer cognitive functioning | MMSE, Telephone Interview of Cognitive Status |
| **McGrath et al. 2019b^138^** | Health and Retirement Study (HRS) | 13828  *N/A* | >50 | 8-year | Handgrip Strength (absolute) | Dynamometer (Smedley) | - Change in HGS:  Per 5 kg decrement in HGS | Cognitive function:  - Any CI  - Severe CI  - Poorer cognitive functioning | MMSE, Telephone Interview of Cognitive Status, MMSE. |
| Peng et al. 2024^140^ | Taiwan  Initiative of Geriatric Epidemiological Research (TIGER) | 392  Females=213  Males=179 | ≥65  75.77±4.85  68–90 | 4-year | Handgrip Strength (absolute) | Hydraulic hand dynamometer  (Lafayette Instruments, Lafayette, Ind., USA) | - HGS asymmetry (ratio left handgrip strength (kg)/right handgrip strength (kg):  Normal (<15%difference); Mild (15-25% difference); Moderate (>25% difference) | Cognitive impairment | The Wechsler Memory Scale-Third Edition (WMS-III), The Trail Making Tests A and B, animal fluency task, and the digit span-forward and backward  tests. Global cognitive function was assessed using the MoCA-T score. |
| Sattler et al. 2011^141^ | German Interdisciplinary Longitudinal Study on Adult Development and Aging (ILSE; Germany) | 381  *N/A* | 75.92±1.09 | 12-year | Handgrip Strength (absolute) | Dynamometer (Martin-Vigorimeter) | *N/A* | Mild Cognitive Impairment | International Classification of Diseases, 10th revision. |
| **Stessman et al. 2017^99^** | Jerusalem Longitudinal Study | 3015  Females=1214  Males=1801 | 70-90 | 20-year | Handgrip Strength (absolute) | Dynamometer (5001 Grip-A; Takei, Niigata City, Japan) | - Sex-age specific HGS quartiles:  Q4 the lowest | Cognitive status | MMSE. |
| Stijntjes et al. 2017^142^ | - The Longitudinal Aging Study Amsterdam (LASA)  - The Leiden 85-plus Study | 2545  Females=1358; Males=1187  434  Females=289; Males=145 | - 55-85  - 85 | 5-12-year | Handgrip Strength (absolute) | Dynamometer (*N/A*) | Change in HGS:  *N/A* | Cognitive decline | MMSE. |
| **Taekema et al. 2010^100^** | Leiden 85-plus Study (The Netherlands) | 555  Females=361  Males=194 | 85 | 2-year | Handgrip strength (absolute) | Dynamometer (Jamar, Sammons Preston Inc., Boling- brook, IL) | - Sex-age-specific HGS tertiles:  Females, T1=<21-32 kg; T2=17-20 kg; T3=1-16 kg.  Males, T1=34-54 kg; T2=20-33 kg; T3=10-27 kg. | Cognitive decline | MMSE. |
| Peng et al. 2023^139^ | Taiwan Initiatives for Geriatric Epidemiological Research (TIGER) | 394  F: 214  M: 180 | ≥65  75.8 ± 4.8 years | 2-years | Handgrip Strength (absolute) | Hand-held dynamometer (Lafayette Hydraulic Hand Dynamometer) | - Sex-specific categories:  Females=<16 kg  Males=<27 kg | Cognitive impairment | Z scores of several tests: MoCA-T, Wechsler Memory Scale-III, and Trail Making Tests |
| **Veronese et al. 2016^143^** | Progetto Veneto Anziani study (Italy) | 1249  Females=743  Males=506 | ≥65  72.2 ± 5.8 | 4-year | - Handgrip Strength (absolute)  - Chair stand (5 reps, seconds) | - Dynamometer (Jamar. Sammons Preston INC, Bolingbrook, IL, USA)  - Chair stand (Chair rise speed) | Sex-age specific tertiles:  T1 the lowest | Cognitive decline/impairment | MMSE |
| Werneck et al. 2023^144^ | Survey of Health, Aging and Retirement in Europe (SHARE) | 19686  Females=11459  Males=8227 | 64.9±8.7 | 10.2-year | Handgrip Strength (absolute) | Dynamometer (Smedley, S Dynamometer, TTM, Tokyo, 100 kg) | Change in HGS:  Per 10% increment in HGS | Mild cognitive impairment | Rey's Auditory Verbal Learning Test and animal fluency task. |
| **DEMENTIA (n=12)** | | | | | | | | | |
| **Camargo et al. 2016^1^** | The Framingham Offspring Cohort | 2176  Females=1175  Males=1001 | 35-84  62±8 | 11-year | Handgrip Strength (absolute) | Dynamometer (Jamar Hydraulic Hand Dynamometer; Lafayette) | - Cut-off points:  ≤10th sex-specific percentile versus >10th percentile  Females=15 kg  Males=30 kg | - Alzheimer  - Dementia | MMSE, Physical examination. |
| **Doi et al. 2019^145^** | National Center for Geriatrics and Gerontology Study of  Geriatric Syndromes (Japan) | 4086  Females=2125  Males=1961 | ±72.0 | 3.6-year | - Handgrip Strength (absolute)  - Chair stand (5 reps, seconds) | - Dynamometer (Smedley-type handheld dynamometer. GRIP-D; Takei Ltd, Niigata, Japan)  - Chair stand (Chair rise speed) | - Sex-specific HGS quartiles:  HGS, females: Q1=>23.7 kg; Q2=≤23.7-21.0 kg; Q3=≤21.0-18.3 kg; Q4=≤18.3  HGS, males: Q1=>37.3 kg; Q2=≤37.3-33.1 kg; Q3=≤33.1-29.2 kg; Q4=≤29.2 kg.  Chair stand, females: Q1=≤6.82 sec; Q2=>6.82-8.15 sec; Q3=>8.15-9.84 sec; Q4=>9.84 sec  Chair stand, males: Q1=≤6.92 sec; Q2=>6.92-8.25 sec; Q3=>8.25-9.80 sec; Q4=>9.80 sec. | Dementia | Medical records. |
| **Duchowny et al. 2022^146^** | UK Biobank | 190406  Females=102735  Males=87671 | 39-73 | 11.7-year | Handgrip Strength (absolute) | Dynamometer (Jamar) | - Sex-specific HGS change:  5 kg decrement in HGS | - Dementia Risk  - Cognition | Diagnoses obtained from primary care, hospital inpatient, and death registry records. |
| **Esteban-Cornejo et al. 2022^147^** | UK Biobank | 466788  Females=254535  Males=212253 | 37-73  56.51±8.08 | 9.1-year | Handgrip Strength (absolute) | Dynamometer (Jamar) | - Sex-age-specific HGS quintiles:  Q5=40.82±10.64 kg; Q4=35.05±8.87 kg; Q3=30.83±8.20 kg; Q2=27.00±7.73 kg; Q1=21.12±7.85 kg  - Sex-age-specific HGS change:  Per 5 kg decrement in HGS | Dementia | International Classification of Diseases, codes F00, F01, F02 and F03. |
| **Hatabe et al. 2020^148^** | Hisayama study | 835  Females=485  Males=350 | 45-64 | 14.6-year | Handgrip Strength (absolute) | Dynamometer (Smedley, Japan) | - Sex-age specific tertiles:  *N/A*  - Cut-off sex-age specific:  Females, Low HGS:  45-49 years=<22 kg; 50-54 years=<20.5kg; 55-59 years=<20kg; 60-64 years=<19 kg  Males, Low HGS:  45-49 years=<36 kg; 50-54 years=<33.5kg; 55-59 years=<33.5kg; 60-64 years=<30.5 kg | - Dementia  - Alzheimer  - Vascular dementia | Diagnostic and Statistical Manual of Mental Disorders, NINCDS-ADRDA, and NINDS-AIREN diagnostic criteria. |
| **He et al. 2023^149^** | UK Biobank | 495700  Females=269839  Males=225861 | 56.5±8.1 | 12-year | Handgrip Strength (absolute) | Dynamometer (Jamar J00105 hydraulic hand dynamometer) | - Sex-specific HGS quartiles:  Females: Q1=<19.5 kg; Q2=19.5 to <23.0 kg; Q3=23.0 to <27.5kg; Q4=>27.5 kg  Males: Q1=<34.0 kg; Q2=34.0 to <39.5 kg; Q3=39.5 to <45.0 kg; Q4=>45.0 kg | Dementia | Diagnoses were recorded using the International Classification of Diseases (9th and 10th revisions) coding system. |
| **Kuo et al. 2022^150^** | UK Biobank | 340212  Females=198535  Males=141677 | 40-69 | 8.51 ± 2.68-year | Handgrip Strength (absolute and relative by body weight) | Dynamometer (Jamar Hydraulic Hand 100105) | - HGS increments:  Absolute (+5 kg): reduced dementia risk  Relative (+0.05 kg/kg): reduced risk | - Risks of all-cause dementia  - Alzheimer  - Vascular dementia | International Classification of Diseases (ICD)-9 and ICD-10 coding systems. |
| Peterson et al. 2021^93^ | The Health and Retirement Study (HRS) | 12618  Females=7652  Males=4966 | >50 | 8-year | Handgrip Strength (relative by BMI) | Dynamometer (Smedley spring-type, Scandidact, Odder, Denmark) | - HGS change:  Per each 0.05-unit decrement in HGS | Dementia | Different tests adapted from the Telephone Interview for Cognitive Status. |
| Sattler et al. 2011^141^ | German Interdisciplinary Longitudinal Study on Adult Development and Aging (ILSE; Germany) | 381  *N/A* | 75.92±1.09 | 12-year | Handgrip Strength (absolute) | Dynamometer (Martin-Vigorimeter) | *N/A* | Alzheimer Disease | NINCDS-ADRDA and NINDS-AIREN diagnostic criteria. |
| Sibbett et al. 2018^151^ | Lothian Birth Cohort 1921 (LBC1921) | 488  Females=280  Males=208 | 79 | 9-year | Handgrip Strength (absolute) | Dynamometer (Jamar) | *N/A* | Dementia | Medical consensus criteria. |
| **Stephan et al. 2024^152^** | ELSA (UK) | 3667  Females=2006 Males=1661 | ≥60  69.90±7.02 | 10.17 years  11.75 years | Chair Stand (5 reps, seconds) | Chair stand (Chair rise speed) | - Chair Stand cut-offs:  Fail=>15 sec | Dementia Risk | Dementia diagnosed through validated cognitive assessments (TICSm in HRS, IQCODE in ELSA). |
| Werneck et al. 2023^144^ | Survey of Health, Aging and Retirement in Europe (SHARE) | 19686  Females=11459  Males=8227 | 64.9±8.7 | 10.2-year | Handgrip Strength (absolute) | Dynamometer (Smedley, S Dynamometer, TTM, Tokyo, 100 kg) | - Change in HGS:  Per 10% increment in HGS | Dementia | Question reported by participants. |
| **PARKINSON (n=3)** | | | | | | | | | |
| **Liu et al. 2024b^153^** | UK Biobank | 419 572 Females=226811  Males=192761 | 37–73  56.1±8.2 | 12.5-year | Handgrip Strength (absolute) | Dynamometer (Jamar J00105 hydraulic) | - HGS Tertiles:  Females, T1=<21 kg; T2=21–26 kg; T3=≥26 kg  Males, T1=<36 kg; T2=36–43 kg; T3=≥43 kg | Incident Parkinson's Disease | Verified hospital admissions, death registries, self-reported diagnosis |
| **Mey et al. 2023^154^** | SHARE (Survey of Health, Aging, and Retirement in Europe) | 71702  Females=39617  Males=32085 | ≥50  65.2±9.7 | 5.0-year | Handgrip Strength (absolute) | Dynamometer (Smedley S Dynamometer, TTM, Tokyo, 100 kg) | - HGS Tertiles:  Females, T1=<24 kg; T2=24–38 kg; T3=≥38 kg  Males, T1=<27 kg; T2=27–59 kg; T3=≥59 kg | Parkinson's Disease | Doctor-diagnosed PD via self-report. |
| **Wu et al. 2024^155^** | UK Biobank | 422,531  Males= 193,078  Females= 229,453 | 37–73 | 9.23 years | Handgrip Strength (absolute and relative by body weight) | Dynamometer (Jamar Hydraulic Hand J00105) | - Change HGS:  Per 5 kg increment  Per 0.05 kg/kg increment | Parkinson's Disease | Diagnoses obtained from primary care, hospital inpatient, and death registry records. |

*Abbreviations:* ADL, Activities of Daily Living; BMI, Body Mass Index; CHARLS, Chinese Population-Representative Sample; CI, Cognitive Impairment; HGS, Handgrip Strength; IADL, Instrumental Activities of Daily Living; K-MMSE, Korean mini-mental state examination; MMSE, Mini-Mental State Examination; NHANES, U.S. Population-Representative Sample; NINCDS-ADRDA, National Institute of Neurological and Communicative Disorders and Stroke and the Alzheimer's Disease and Related Disorders Association; NINDS-AIREN, National Institute of Neurological Disorders and Stroke and Association Internationale pour la Recherche et l’Enseignement en Neurosciences; PA, Physical Activity; PD, Parkinson’s Disease; T2DM, Type 2 diabetes mellitus.

*Notes:* N/A indicates Not Applicable because that information was not reported. Bolds indicate studies included in the meta-analysis.

**Table S6**. Subgroup analyses according to geographical region for the prospective associations between handgrip strength and long-term health conditions.

| ***Highest vs. lowest category*** *^1^* | ***Asia*** | | |  |  | ***Europe*** | | | | | | ***North America*** | | | |
| --- | --- | --- | --- | --- | --- | --- | --- | --- | --- | --- | --- | --- | --- | --- | --- |
|  | N | OR (95% CI) | p-value | *I*^2^ (%) |  | N | OR (95% CI) | p-value | *I*^2^ (%) |  | N | | OR (95% CI) | p-value | *I*^2^ (%) |
| Cardiovascular diseases | 6 | **0.82 (0.76, 0.88)** | <0.001 | 0 |  | 6 | **0.65 (0.58, 0.74)** | <0.001 | 0 |  | 3 | | **0.71 (0.58, 0.86)** | 0.001 | 0 |
| Cancer | 0 | NA | NA | NA |  | 1 | **0.73 (0.70, 0.76)** | NA | NA |  | 0 | | NA | NA | NA |
| Type 2 Diabetes Mellitus | 3 | **0.75 (0.57, 0.99)** | 0.042 | 53.5 |  | 2 | **0.81 (0.74, 0.90)** | <0.001 | 45.3 |  | 0 | | NA | NA | NA |
| Respiratory diseases | 0 | NA | NA | NA |  | 1 | **0.53 (0.50, 0.57)** | NA | NA |  | 0 | | NA | NA | NA |
| Musculoskeletal impairment | 3 | **0.63 (0.50, 0.81)** | <0.001 | 59.7 |  | 5 | **0.71 (0.52, 0.98)** | 0.037 | 57.5 |  | 1 | | 0.61 (0.24, 1.55) | NA | NA |
| Disability (disability ADL, functional mobility, ambulatory status) | 5 | **0.57 (0.40, 0.80)** | 0.001 | 63.8 |  | 5 | 0.61 (0.36, 1.01) | 0.056 | 50.7 |  | 2 | | **0.52 (0.43, 0.63)** | <0.001 | 0 |
| Anxiety (diagnosis or moderate to severe symptoms) | 0 | NA | NA | NA |  | 3 | **0.79 (0.63, 0.99)** | 0.040 | 0 |  | 0 | | NA | NA | NA |
| Depression (diagnosis or mild to severe symptoms) | 2 | **0.71 (0.54, 0.93)** | 0.013 | 0 |  | 6 | **0.64 (0.59, 0.70)** | <0.001 | 0 |  | 1 | | **0.82 (0.73, 0.92)** | NA | NA |
| Cognitive decline | 3 | **0.75 (0.64, 0.87)** | <0.001 | 0 |  | 1 | 0.70 (0.45, 1.10) | NA | NA |  | 2 | | **0.45 (0.31, 0.65)** | <0.001 | 74.1 |
| Dementia | 2 | **0.69 (0.56, 0.86)** | 0.001 | 0 |  | 2^2^ | **0.52 (0.40, 0.69)** | NA | NA |  | 1 | | **0.46 (0.21, 1.00)** | NA | NA |
| Parkinson's disease | 0 | NA | NA | NA |  | 3 | **0.53 (0.31, 0.91)** | 0.022 | 89.0 |  | 0 | | NA | NA | NA |
| **Random effects model** | 24 | **0.71 (0.64, 0.77)** | <0.001 | 41.3 |  | 33 | **0.66 (0.60, 0.72)** | <0.001 | 84.6 |  | 10 | | **0.58 (0.48, 0.69)** | <0.001 | 80.4 |
| **Per 5-kg increment** ^3^ | n | OR (95% CI) | p-value | *I*^2^ (%) |  | n | OR (95% CI) | p-value | *I*^2^ (%) |  | n | | OR (95% CI) | p-value | *I*^2^ (%) |
| Cardiovascular diseases | 2 | **0.92 (0.88, 0.96)** | <0.001 | 0 |  | 4 | 0.92 (0.83, 1.02) | 0.106 | 89.6 |  | 2 | | **0.95 (0.91, 1.00)** | 0.044 | 0 |
| Cancer | 0 | NA | NA | NA |  | 1 | 0.98 (0.88, 1.08) | NA | NA |  | 0 | | NA | NA | NA |
| Type 2 Diabetes Mellitus | 1 | 0.94 (0.88, 1.01) | NA | NA |  | 2 | **0.93 (0.86, 1.00)** | 0.049 | 58.0 |  | 3 | | 0.91 (0.78, 1.06) | 0.233 | 21.0 |
| Respiratory diseases | 0 | NA | NA | NA |  | 1 | **0.84 (0.81, 0.87)** | NA | NA |  | 0 | | NA | NA | NA |
| Musculoskeletal impairment | 3 | 0.87 (0.76, 1.00) | 0.056 | 65.1 |  | 0 | NA | NA | NA |  | 1 | | **0.92 (0.88, 0.96)** | NA | NA |
| Disability (disability ADL, functional mobility, ambulatory status) | 2 | **0.59 (0.48, 0.73)** | <0.001 | 0 |  | 2 | 0.96 (0.88, 1.06) | 0.421 | 0 |  | 2 | | **0.81 (0.74, 0.89)** | <0.001 | 0 |
| Depression (diagnosis or mild to severe symptoms) | 1 | **0.90 (0.85, 0.94)** | NA | NA |  | 3 | 0.95 (0.89, 1.02) | 0.137 | 90.0 |  | 1 | | **0.94 (0.92, 0.97)** | NA | NA |
| Cognitive decline | 2 | **0.83 (0.71, 0.96)** | 0.015 | 0 |  | 1 | **0.95 (0.91, 0.99)** | NA | NA |  | 2^2^ | | 0.94 (0.88, 1.00) | NA | NA |
| Dementia | 0 | NA | NA | NA |  | 3^2^ | **0.87 (0.84, 0.91)** | NA | NA |  | 2 | | **0.86 (0.77, 0.97)** | 0.012 | 0 |
| Parkinson's disease | 0 | NA | NA | NA |  | 3 | **0.89 (0.87, 0.91)** | <0.001 | 0 |  | 0 | | NA | NA | NA |
| **Random effects model** | 11 | **0.85 (0.77, 0.94)** | 0.001 | 64.0 |  | 20 | **0.92 (0.89, 0.95)** | <0.001 | 87.4 |  | 13 | | **0.91 (0.87, 0.95)** | <0.001 | 37.6 |

Bold values indicate a statistically significant association (*p* < 0.05). N represents the number of studies included in each subgroup analysis.

**Abbreviations:** **CI**, confidence interval; **ADL,** activities of daily living activities; **NA**, not applicable; **OR**, odds ratio.

^1^ Only one study was conducted in Africa, Oceania and South America, and no studies were reported from the Caribbean; therefore, these regions were not included in the subgroup analyses. ^2^ A meta-analysis was not conducted on studies from the same cohort (n=1 comparison). ^3^ Only one study was conducted in Oceania, and no studies were reported from Africa, South America and the Caribbean; therefore, these regions were not included in the subgroup analyses.

**Table S7**. Subgroup analyses according to clinical complexity (participants with co-existing long-term health conditions at baseline) for the prospective associations between handgrip strength and long-term health conditions.

| ***Highest vs. lowest category*** | **<50% ^1^** | | |  |  | **≥50% ^1^** | | |  | |  |
| --- | --- | --- | --- | --- | --- | --- | --- | --- | --- | --- | --- |
|  | N | OR (95% CI) | p-value | *I*^2^ (%) |  | N | OR (95% CI) | p-value | | *I*^2^ (%) | |
| Cardiovascular diseases | 4 | **0.82 (0.75, 0.89)** | <0.001 | 0 |  | 11 | **0.67 (0.60, 0.74)** | <0.001 | | 0 | |
| Cancer | 0 | NA | NA | NA |  | 1 | **0.73 (0.70, 0.76)** | NA | | NA | |
| Type 2 Diabetes Mellitus | 1 | **0.64 (0.50, 0.81)** | NA | NA |  | 4 | **0.82 (0.72, 0.93)** | 0.003 | | 16.0 | |
| Respiratory diseases | 0 | NA | NA | NA |  | 1 | **0.53 (0.50, 0.57)** | NA | | NA | |
| Musculoskeletal impairment | 4 | **0.64 (0.52, 0.77)** | <0.001 | 53.1 |  | 6 | **0.67 (0.53, 0.86)** | 0.001 | | 60.7 | |
| Disability (disability ADL, functional mobility, ambulatory status) | 3 | **0.49 (0.40, 0.62)** | <0.001 | 0 |  | 9 | **0.62 (0.44, 0.87)** | 0.006 | | 46.6 | |
| Anxiety (diagnosis or moderate to severe symptoms) | 0 | NA | NA | NA |  | 3 | **0.79 (0.63, 0.99)** | 0.040 | | 0 | |
| Depression (diagnosis or mild to severe symptoms) | 3 | **0.70 (0.58, 0.84)** | <0.001 | 0 |  | 6 | **0.70 (0.62, 0.80)** | <0.001 | | 70.0 | |
| Cognitive decline | 4 | **0.60 (0.40, 0.83)** | 0.003 | 81.5 |  | 4 | **0.55 (0.36, 0.86)** | 0.008 | | 70.2 | |
| Dementia | 3 | **0.66 (0.51, 0.87)** | 0.003 | 0 |  | 2^2^ | **0.59 (0.51, 0.64)** | NA | | NA | |
| Parkinson's disease | 0 | NA | NA | NA |  | 3 | **0.53 (0.31, 0.91)** | 0.022 | | 89.0 | |
| **Random effects model** | 22 | **0.64 (0.58, 0.71)** | <0.001 | 67.0 |  | 53 | **0.67 (0.61, 0.73)** | <0.001 | | 82.5 | |

Bold values indicate a statistically significant association (*p* < 0.05). N represents the number of studies included in each subgroup analysis.

**Abbreviations:** **CI**, confidence interval; **ADL,** activities of daily living activities; **NA**, not applicable; **OR**, odds ratio.

^1^ Percentage of participants with at least one co-existing long-term health condition at baseline as reported by the included studies. ^2^ A meta-analysis was not conducted on studies from the same cohort (n=1 comparison).

**Table S8**. Sensitivity analyses for handgrip strength including studies that specifically analysed 5-kg increments and that applied sex-specific cut-off points for clinically relevant muscular weakness.

| **Long-term health condition** | **No muscular weakness vs. muscular weakness ^1^** | | | |  | **Per 5-kg increment ^2^** | | | |
| --- | --- | --- | --- | --- | --- | --- | --- | --- | --- |
|  | N | OR (95% CI) | p-value | *I*^2^ (%) |  | N | OR (95% CI) | p-value | *I*^2^ (%) |
| Cardiovascular diseases | 6 | **0.77 (0.69, 0.86)** | <0.001 | 39.0 |  | 4 | **0.91 (0.86, 0.96)** | <0.001 | 42.9 |
| Cancer | 1 | **0.82 (0.79, 0.86)** | NA | NA |  | 2 | 0.99 (0.87, 1.12) | 0.842 | 96.2 |
| Type 2 Diabetes Mellitus | 0 | NA | NA | NA |  | 4 | **0.93 (0.88, 0.99)** | 0.019 | 55.0 |
| Respiratory diseases | 1 | **0.61 (0.57, 0.64)** | NA | NA |  | 2 | 0.91 (0.78, 1.07) | 0.256 | 97.0 |
| Musculoskeletal impairment | 4 | **0.68 (0.54, 0.87)** | 0.002 | 11.2 |  | 3 | 0.93 (0.77,1.11) | 0.427 | 83.4 |
| Disability (disability ADL, functional mobility, ambulatory status) | 6 | **0.59 (0.41, 0.86)** | 0.005 | 46.9 |  | 1 | **0.55 (0.35, 0.85)** | NA | NA |
| Anxiety (diagnosis or moderate to severe symptoms) | 3 | **0.79 (0.63, 0.99)** | 0.040 | 0 |  | 0 | NA | NA | NA |
| Depression (diagnosis or mild to severe symptoms) | 5 | **0.78 (0.69, 0.89)** | <0.001 | 43.3 |  | 2 | **0.94 (0.92, 0.96)** | <0.001 | 0 |
| Cognitive decline | 3 | **0.52 (0.30, 0.91)** | 0.021 | 80.0 |  | 3 | 0.94 (0.88, 1.01) | 0.080 | 0 |
| Dementia | 1 | **0.46 (0.21, 1.00)** | NA | NA |  | 3^3^ | **0.87 (0.84, 0.91)** | NA | NA |
| Parkinson's disease | 0 | NA | NA | NA |  | 2 | **0.86 (0.78, 0.96)** | 0.005 | 65.9 |
| **Random effects model** | 30 | **0.69 (0.62, 0.77)** | <0.001 | 79.1 |  | 26 | **0.92 (0.88, 0.96)** | <0.001 | 89.6 |

Bold values indicate a statistically significant association (*p* < 0.05). N represents the number of studies included in each subgroup analysis.

**Abbreviations:** **CI**, confidence interval; **ADL,** activities of daily living activities; **NA**, not applicable; **OR**, odds ratio.

^1^ Muscular weakness was defined according to the Foundation for the National Institutes of Health Sarcopenia Project (handgrip strength <26 kg for men and <16 kg for women) ^156^ and to other specific criteria that applied similar cut-off points (<31 kg for men and <21 kg for women). ^2^ Cohort studies that analysed the increase or decrease in handgrip strength with values other than 5-kg were excluded.^3^ A meta-analysis was not conducted on studies from the same cohort (n=1 comparison).

**Table S9**. Cohort studies reporting prospective associations between relative handgrip strength and long-term health conditions.

| **Highest vs. lowest category** | **Mean age** | **n, total** | **n, cases** | **HGS metric** | **High level** | **Low level** | **Outcome** | **OR ^1^** | **LL** | **UL** |
| --- | --- | --- | --- | --- | --- | --- | --- | --- | --- | --- |
| Jang et al. (2020)^5^ | 60.7 | 8494 | 688 | kg/BMI (kg/m^2^) | ≥2.05♀ ≥3.19♂ | <1.42♀ <2.37♂ | CVD | **0.76** | **0.70** | **0.84** |
| Kim et al. (2021)^7^ | 56.7 | 189,846 | 2766 | kg/TLM (kg) | ≥0.55♀ ≥0.66♂ | <0.53♀ <0.61♂ | CVD | **0.76** | **0.71** | **0.82** |
| Li et al. (2023)^9^ | 63.0 | 96,263 | 13,916 | kg/BMI (kg/m^2^) | ≥2.04♀ ≥3.33♂ | <1.72♀ <2.78♂ | CVD | **0.70** | **0.62** | **0.79** |
| Liu et al. (2024a)^11^ | 56.4 | 430,886 | 5,661 | kg/BM (kg) | ≥0.38♀ ≥0.53♂ | <0.39♀ <0.52♂ | CVD | **0.80** | **0.73** | **0.88** |
| Qi et al. (2024)^14^ | 58.9 | 7904 | 532 | kg/BM (kg) | NR | NR | CVD | **0.79** | **0.63** | **0.98** |
| Zhang et al. (2024)^18^ | 52.5 | 626 | 228 | kg/BM (kg) | ≥0.46♀ ≥0.62♂ | <0.36♀ <0.50♂ | CVD | **0.69** | **0.52** | **0.91** |
| Parra-Soto et al. (2022)^19^ | 56.3 | 438,871 | 37,297 | kg/BMI (kg/m^2^) | ≥1.27♀ ≥1.90♂ | <0.53♀ <0.97♂ | Cancer | **0.96** | **0.93** | **0.99** |
| Boonpor et al. (2021)^22^ | 55.8 | 166,894 | 3713 | kg/BM (kg) | ≥0.37♀ ≥0.51♂ | <0.28♀ <0.40♂ | T2DM | **0.53** | **0.45** | **0.63** |
| Hao et al. (2020)^23^ | 57.6 | 3514 | 333 | kg/BMI (kg/m^2^) | 1.50♀ 2.20♂ | 0.80♀ 1.30♂ | T2DM | 0.47 | 0.13 | 1.75 |
| He et al. (2024)^24^ | 58.4 | 4027 | 563 | kg/BM (kg) | ≥0.55♀ ≥0.71♂ | <0.44♀ <0.60♂ | T2DM | **0.77** | **0.64** | **0.93** |
| Jeon et al. (2021) ^25^ | 48.0 | 2699 | 976 | kg/BMI (kg/m^2^) | NR | NR | T2DM | **0.72** | **0.59** | **0.88** |
| Lee et al. (2024)^31^ | 53.5 | 33,326 | 1473 | kg/BMI (kg/m^2^) | >0.30♀ >1.17♂ | ≤0.85♀ ≤1.40♂ | T2DM | **0.66** | **0.51** | **0.84** |
| Li et al. (2021)^33^ | 61.2 | 66,100 | 5661 | kg/BMI (kg/m^2^) | ≥2.40♀ ≥3.70♂ | <1.60♀ <2.60♂ | T2DM | **0.60** | **0.54** | **0.67** |
| Mahala Manda et al. (2020)^34^ | 42.2 | 1027 | 165 | kg/BMI (kg/m^2^) | NR | NR | T2DM | **0.59** | **0.40** | **0.86** |
| McGrath et al. (2017)^36^ | 73.3 | 1903 | . | kg/BM (kg) | >0.30♀ >0.46♂ | ≤0.30♀ ≤0.46♂ | T2DM | **0.81** | **0.79** | **0.83** |
| Momma et al. (2019)^37^ | 50.0 | 10,893 | 496 | kg/BM (kg) | 0.62♀ 0.79♂ | 0.41♀ 0.56♂ | T2DM | **0.64** | **0.51** | **0.81** |
| Park et al. (2024)^38^ | 68.0 | 1935 | 203 | kg/BMI (kg/m^2^) | 1.37♀♂ | 0.86♀♂ | T2DM | **0.54** | **0.36** | **0.80** |
|  |  |  |  | kg/BM (kg) | 0.51♀♂ | 0.35♀♂ |  | **0.50** | **0.34** | **0.73** |
| Qiu et al. (2023)^39^ | 58.7 | 2487 | 196 | kg/BM (kg).year | ≥2.29♀♂ | <1.81♀♂ | T2DM | **0.60** | **0.41** | **0.88** |
| Sohn et al. (2024)^40^ | 73.8 | 22,016 | 1351 | kg/BMI (kg/m^2^) | 1.21♀ 1.40♂ | 0.61♀ 0.65♂ | T2DM | 0.89 | 0.69 | 1.13 |
| Yan et al. (2024)^68^ | 58.7 | 12,043 | 2008 | kg/BM (kg) | ≥0.65♀♂ | <0.45♀♂ | MSI | **0.82** | **0.72** | **0.94** |
| Bao et al. (2022) ^105^ | 57.6 | 2644 | 756 | kg/BMI (kg/m^2^) | NR | NR | Depression | **0.58** | **0.43** | **0.77** |
| Kuo et al. (2022)^150^ | 56.0 | 340,212 | 2424 | kg/BM (kg) | NR | NR | Dementia | **0.77** | **0.67** | **0.87** |
| Liu et al. (2024b)^153^ | 56.1 | 279,233 | 1473 | kg/BMI (kg/m^2^) | NR | NR | Parkinson’s disease | **0.67** | **0.59** | **0.75** |
| Wu et al. (2024)^155^ | 56.8 | 167,576 | 747 | kg/BM (kg) | >0.37♀ >0.52♂ | 0.27-0.39♀ 0.40-0.52♂ | Parkinson’s disease | **0.75** | **0.65** | **0.87** |
| **Continuous exposure** | **Mean age** | **n, total** | **n, cases** | **HGS metric** | **HGS increment** | | **Outcome** | **OR ^1^** | **LL** | **UL** |
| Jang et al. (2020)^5^ | 60.7 | 8494 | 688 | kg/BMI (kg/m^2^) | ↑0.28 ♀ / ↑0.44 ♂ | | CVD | **0.82** | **0.77** | **0.87** |
| Kim et al. (2021)^7^ | 56.7 | 284,767 | 4008 | kg/TLM (kg) | ↑0.10 ♀♂ | | CVD | **0.88** | **0.85** | **0.91** |
| Li et al. (2023)^9^ | 63.0 | 96,263 | 13,916 | kg/BMI (kg/m^2^) | ↑0.8-1.0 ♀♂ | | CVD | **0.83** | **0.78** | **0.89** |
| Liu et al. (2024a)^11^ | 56.4 | 430,886 | 5,661 | kg/BM (kg) | ↑0.01 ♀♂ | | CVD | **0.17** | **0.13** | **0.23** |
| Zhang et al. (2024)^18^ | 52.5 | 1258 | 462 | kg/BM (kg) | ↑1.00 ♀♂ | | CVD | **0.16** | **0.05** | **0.52** |
| Parra-Soto et al. (2022)^20^ | 56.3 | 438,792 | 37,291 | kg/BMI (kg/m^2^) | ↑0.29 ♀ / ↑0.37 ♂ | | Cancer | 0.98 | 0.97 | 1.00 |
| Boonpor et al. (2021)^22^ | 55.8 | 166,894 | 3713 | kg/BM (kg) | ↑0.10 ♀♂ | | T2DM | **0.86** | **0.81** | **0.92** |
| Jeon et al. (2021)^25^ | 48.0 | 2699 | 976 | kg/BMI (kg/m^2^) | ↑0.19 ♀ / ↑0.26 ♂ | | T2DM | **0.88** | **0.82** | **0.94** |
| Karvonen-Gutierrez et al. (2018)^26^ | 46.4 | 424 | 157 | kg/BM (kg) | ↑0.10 ♀ | | T2DM | **0.81** | **0.70** | **0.94** |
| Kunutsor et al. (2020)^29^ | 69.0 | 776 | 59 | kPa/BM (kg) | ↑0.49 ♀♂ | | T2DM | **0.53** | **0.29** | **0.97** |
| Lee et al. (2024)^31^ | 53.5 | 33,326 | 1473 | kg/BMI (kg/m^2^) | ↑0.01 ♀♂ | | T2DM | **0.62** | **0.48** | **0.80** |
| Li et al. (2016)^32^ | 54.1 | 1632 | 146 | kg/ALM (kg) | ↑1.00 ♂ | | T2DM | **0.84** | **0.75** | **0.94** |
| Li et al. (2021)^33^ | 61.2 | 66,100 | 5661 | kg/BMI (kg/m^2^) | ↑0.78 ♀ / ↑1.09 ♂ | | T2DM | **0.81** | **0.77** | **0.85** |
| Qiu et al. (2023)^39^ | 58.7 | 3731 | 300 | kg/BM (kg) year | ↑0.53 ♀♂ | | T2DM | **0.79** | **0.68** | **0.93** |
| Cheung et al. (2012)^47^ | 64.1 | 1702 | 43 | kg/BM (kg) | ↑1.10 ♀ / ↑0.95 ♂ | | MSI | **0.64** | **0.43** | **0.94** |
| Peterson et al. (2021)^93^ | 66.8 | 7739 | NR | kg/BM (kg) | ↑0.05 ♀♂ | | Disability | **0.78** | **0.75** | **0.82** |
| Zhao et al. (2020)^116^ | 57.7 | 8470 | 2027 | kg/BM (kg) | ↑0.06 ♀♂ | | Depression | **0.93** | **0.87** | **0.98** |
| Kuo et al. (2022)^150^ | 56.0 | 340,212 | 2424 | kg/BM (kg) | ↑0.05 ♀♂ | | Dementia | **0.92** | **0.90** | **0.94** |
| Peterson et al. (2021)^93^ | 66.8 | 7606 | NR | kg/BM (kg) | ↑0.05 ♀♂ | | Dementia | 0.94 | 0.88 | 1.02 |
| Wu et al. (2024)^155^ | 56.8 | 422,531 | 2118 | kg/BM (kg) | ↑0.05 ♀♂ | | Parkinson’s disease | **0.91** | **0.89** | **0.93** |

Bold values indicate a statistically significant association (*p* < 0.05). **Abbreviations:** **ALM**, arm lean mass; **BM**, body mass; **BMI**; body mass index; **CI**, confidence interval; **CVD**, cardiovascular diseases; **T2DM**, Type 2 diabetes mellitus; **HGS**, handgrip strength; **kg**, kilograms; **kPa**, kilopascal; **LL**, lower limit; **MSI**, musculoskeletal impairment; **NR**, not reported; **OR**, odds ratio; **TLM**, total lean mass; **UL**, upper limit.

^1^ The effect sizes provided in the included studies (hazard ratios, odds ratios, or risk ratios) were reported in this systematic review and meta-analysis as equivalent to the odds ratio.

**REFERENCES**

1. Camargo EC, Weinstein G, Beiser AS, et al. Association of physical function with clinical and subclinical brain disease: the Framingham Offspring Study. *Journal of Alzheimer's Disease* 2016;53(4):1597-608.

2. Celis-Morales CA, Welsh P, Lyall DM, et al. Associations of grip strength with cardiovascular, respiratory, and cancer outcomes and all cause mortality: prospective cohort study of half a million UK. *bmjcom* 2018

3. Gubelmann C, … PVIjo, undefined. No association between grip strength and cardiovascular risk: the CoLaus population-based study. *Elsevier* 2017

4. Ho FKW, Celis-Morales CA, Petermann-Rocha F, et al. The association of grip strength with health outcomes does not differ if grip strength is used in absolute or relative terms: a prospective cohort study. *Age and ageing* 2019;48(5):684-91. doi: 10.1093/ageing/afz068

5. Jang S-k, Kim J-h, Lee Y. Effect of relative handgrip strength on cardiovascular disease among Korean adults aged 45 years and older: results from the Korean Longitudinal Study of Aging (2006–2016). *Archives of Gerontology and Geriatrics* 2020;86:103937.

6. Klinpudtan N, Kabayama M, Godai K, et al. Association between physical function and onset of coronary heart disease in a cohort of community-dwelling older populations: The SONIC study. *Archives of gerontology and geriatrics* 2021;95:104386.

7. Kim Y, Hwang S, Sharp SJ, et al. Genetic Risk, Muscle Strength, and Incident Stroke: Findings From the UK Biobank Study. *MAYO CLINIC PROCEEDINGS* 2021;96(7):1746-57. doi: 10.1016/j.mayocp.2021.01.034

8. Leong DP, Teo KK, Rangarajan S, et al. Prognostic value of grip strength: findings from the Prospective Urban Rural Epidemiology (PURE) study. *The lancet* 2015;386(9990):266-73.

9. Li G, Lu Y, Shao L, et al. Handgrip strength is associated with risks of new-onset stroke and heart disease: results from 3 prospective cohorts. *BMC geriatrics* 2023;23(1):268. doi: 10.1186/s12877-023-03953-8

10. Liu G, Xue Y, Wang S, et al. Association between hand grip strength and stroke in China: a prospective cohort study. *Aging (albany NY)* 2021;13(6):8204.

11. Liu DQ, Yang CX, Liu G, et al. Association between grip strength, walking pace and incident peripheral artery disease: A prospective study of 430,886 UK biobank participants. *International Journal of Cardiology Cardiovascular Risk and Prevention* 2024;23 doi: 10.1016/j.ijcrp.2024.200330

12. McGrath R, Vincent BM, Hackney KJ, et al. The longitudinal associations of handgrip strength and cognitive function in aging Americans. *Journal of the American Medical Directors Association* 2020;21(5):634-39. e1.

13. Peralta M, Dias CM, Marques A, et al. Longitudinal association between grip strength and the risk of heart diseases among European middle-aged and older adults. *Experimental Gerontology* 2023;171 doi: 10.1016/j.exger.2022.112014

14. Qi K-j, Li Q, Lu G-l, et al. The combined effect of handgrip strength and obesity phenotype on the risk of stroke in Chinese middle-aged and elderly: A cohort study. *Archives of Gerontology & Geriatrics* 2024;124:N.PAG-N.PAG. doi: 10.1016/j.archger.2024.105481

15. Association of fitness and grip strength with heart failure: findings from the UK Biobank population-based study. Mayo Clinic Proceedings; 2019. Elsevier.

16. Yang Z, Wei J, Liu H, et al. Changes in muscle strength and risk of cardiovascular disease among middle-aged and older adults in China: Evidence from a prospective cohort study. *Chinese Medical Journal* 2024;137(11):1343-50.

17. Zhang B, Wang XA, Gu YQ, et al. The association between grip strength and incident carotid atherosclerosis in middle-aged and older adults: The TCLSIH cohort study. *Maturitas* 2023;167:53-59. doi: 10.1016/j.maturitas.2022.09.008

18. Zhang F, Luo B, Bai Y, et al. Association of handgrip strength and risk of cardiovascular disease: a population-based cohort study. *Aging Clin Exp Res* 2024;36(1):207. doi: 10.1007/s40520-024-02856-x

19. Parra-Soto S, Tumblety C, Ho FK, et al. Associations Between Relative Grip Strength and the Risk of 15 Cancer Sites. *AMERICAN JOURNAL OF PREVENTIVE MEDICINE* 2022;62(2):E87-E95. doi: 10.1016/j.amepre.2021.07.015

20. Parra-Soto S, Pell JP, Celis-Morales C, et al. Absolute and relative grip strength as predictors of cancer: prospective cohort study of 445 552 participants in UK Biobank. *JOURNAL OF CACHEXIA SARCOPENIA AND MUSCLE* 2021. doi: 10.1002/jcsm.12863

21. Weber A, Leitzmann MF, Sedlmeier AM, et al. Association between physical activity, grip strength and sedentary behaviour with incidence of malignant melanoma: results from the UK Biobank. *BRITISH JOURNAL OF CANCER* 2021;125(4):593-600. doi: 10.1038/s41416-021-01443-5

22. Boonpor J, Parra-Soto S, Petermann-Rocha F, et al. Associations between grip strength and incident type 2 diabetes: findings from the UK Biobank prospective cohort study. *BMJ Open Diabetes Research and Care* 2021;9(1):e001865.

23. Hao G, Chen H, Ying Y, et al. The Relative Handgrip Strength and Risk of Cardiometabolic Disorders: A Prospective Study. *Frontiers in Physiology* 2020;11((Hao, Ying, Wu, Jing) Department of Epidemiology, School of Medicine, Jinan University, Guangzhou, China(Chen) Department of Endemic Disease, Guangzhou Center for Disease Control and Prevention, Guangzhou, China(Yang) Department of Pathogen Biology, Schoo):719. doi: 10.3389/fphys.2020.00719

24. He YY, Jin ML, Fang XY, et al. Associations of muscle mass and strength with new-onset diabetes among middle-aged and older adults: evidence from the China health and retirement longitudinal study (CHARLS). *Acta Diabetologica* 2024;61(7):869-78. doi: 10.1007/s00592-024-02265-6

25. Jeon Y-J, Lee SK, Shin C. Normalized hand grip and back muscle strength as risk factors for incident type 2 diabetes mellitus: 16 years of follow-up in a population-based cohort study. *Diabetes, Metabolic Syndrome and Obesity* 2021:741-50.

26. Karvonen-Gutierrez CA, Peng Q, Peterson M, et al. Low grip strength predicts incident diabetes among mid-life women: the Michigan Study of Women’s Health Across the Nation. *Age and ageing* 2018;47(5):685-91.

27. Katzmarzyk PT, Craig CL, Gauvin L. Adiposity, physical fitness and incident diabetes: the physical activity longitudinal study. *DIABETOLOGIA* 2007;50(3):538-44. doi: 10.1007/s00125-006-0554-3

28. Kowall B. Lower body muscle strength, dynapenic obesity and risk of type 2 diabetes -longitudinal results on the chair-stand test from the Survey of Health, Ageing and Retirement in Europe (SHARE). *Bmc Geriatrics* 2022;22(1) doi: 10.1186/s12877-022-03647-7

29. Kunutsor SK, Voutilainen A, Laukkanen JA. Handgrip strength improves prediction of type 2 diabetes: a prospective cohort study. *ANNALS OF MEDICINE* 2020;52(8):471-78. doi: 10.1080/07853890.2020.1815078

30. Larsen BA, Wassel CL, Kritchevsky SB, et al. Association of muscle mass, area, and strength with incident diabetes in older adults: the health ABC study. *The Journal of Clinical Endocrinology & Metabolism* 2016;101(4):1847-55.

31. Lee SB, Jo MK, Moon JE, et al. Relationship between Handgrip Strength and Incident Diabetes in Korean Adults According to Gender: A Population-Based Prospective Cohort Study. *Journal of Clinical Medicine* 2024;13(2) doi: 10.3390/jcm13020627

32. Li JJ, Wittert GA, Vincent A, et al. Muscle grip strength predicts incident type 2 diabetes: Population-based cohort study. *Metabolism: clinical and experimental* 2016;65(6):883-92. doi: 10.1016/j.metabol.2016.03.011

33. Li G, Qiao Y, Lu Y, et al. Role of handgrip strength in predicting new-onset diabetes: findings from the survey of health, ageing and retirement in Europe. *BMC geriatrics* 2021;21(1):445-45. doi: 10.1186/s12877-021-02382-9

34. Manda CM, Hokimoto T, Okura T, et al. Handgrip strength predicts new prediabetes cases among adults: A prospective cohort study. *Preventive medicine reports* 2020;17:101056.

35. Marques-Vidal P, Vollenweider P, Waeber G, et al. Grip strength is not associated with incident type 2 diabetes mellitus in healthy adults: The CoLaus study. *Diabetes research and clinical practice* 2017;132:144-48.

36. McGrath R, Vincent BM, Al Snih S, et al. The association between muscle weakness and incident diabetes in older Mexican Americans. *Journal of the American Medical Directors Association* 2017;18(5):452. e7-52. e12.

37. Momma H, Sawada SS, Kato K, et al. Physical fitness tests and type 2 diabetes among Japanese: a longitudinal study from the Niigata Wellness Study. *Journal of epidemiology* 2019;29(4):139-46.

38. Park D, Rho J, Kim E, et al. Comparison of Absolute and Relative Grip Strength to Predict Incidence of Diabetes Mellitus in Korea: A Prospective Cohort Study. *Metabolic Syndrome and Related Disorders* 2024;22(6):463-70. doi: 10.1089/met.2024.0006

39. Qiu S, Cai X, Liang Y, et al. Cumulative muscle strength and risk of diabetes: A prospective cohort study with mediation analysis. *Diabetes Research and Clinical Practice* 2023;197:110562.

40. Sohn YJ, Lee HS, Bae H, et al. Association of relative handgrip strength on the development of diabetes mellitus in elderly Koreans. *PLoS One* 2024;19(10):e0309558. doi: 10.1371/journal.pone.0309558

41. Wander PL, Boyko EJ, Leonetti DL, et al. Greater hand-grip strength predicts a lower risk of developing type 2 diabetes over 10 years in leaner Japanese Americans. *Diabetes research and clinical practice* 2011;92(2):261-64.

42. Zheng J, Zhang L, Jiang M. Lower handgrip strength levels probably precede triglyceride glucose index and associated with diabetes in men not in women. *Journal of Diabetes Investigation* 2022;13(1):148-55.

43. Alajlouni D, Tran T, Bliuc D, et al. Muscle strength and physical performance improve fracture risk prediction beyond Garvan and FRAX: the Osteoporotic Fractures in Men (MrOS) Study. *Journal of Bone and Mineral Research* 2020;37(3):411-19.

44. Albrand G, Munoz F, Sornay-Rendu E, et al. Independent predictors of all osteoporosis-related fractures in healthy postmenopausal women: the OFELY study. *Bone* 2003;32(1):78-85.

45. Beauchamp MK, Kuspinar A, Sohel N, et al. Mobility screening for fall prediction in the Canadian Longitudinal Study on Aging (CLSA): implications for fall prevention in the decade of healthy ageing. *Age and Ageing* 2022;51(5) doi: 10.1093/ageing/afac095

46. Cawthon PM, Fullman RL, Marshall L, et al. Physical performance and risk of hip fractures in older men. *Journal of Bone and Mineral Research* 2008;23(7):1037-44.

47. Cheung C-L, Tan KC, Bow CH, et al. Low handgrip strength is a predictor of osteoporotic fractures: cross-sectional and prospective evidence from the Hong Kong Osteoporosis Study. *Age* 2012;34:1239-48.

48. Delbaere K, Van den Noortgate N, Bourgois J, et al. The Physical Performance Test as a predictor of frequent fallers: a prospective community-based cohort study. *Clinical rehabilitation* 2006;20(1):83-90.

49. Finigan J, Greenfield DM, Blumsohn A, et al. Risk factors for vertebral and nonvertebral fracture over 10 years: a population‐based study in women. *Journal of Bone and Mineral Research* 2008;23(1):75-85.

50. Guo T, Zhang F, Xiong L, et al. Association of Handgrip Strength with Hip Fracture and Falls in Community-dwelling Middle-aged and Older Adults: A 4-Year Longitudinal Study. *Orthop Surg* 2024;16(5):1051-63. doi: 10.1111/os.14029

51. Hussain MA, Qaisar R, Karim A, et al. Predictors of hip fracture in 15 European countries: a longitudinal study of 48,533 geriatric adults using SHARE dataset. *Archives of Osteoporosis* 2024;19(1) doi: 10.1007/s11657-024-01420-4

52. Kamiya K, Kajita E, Tachiki T, et al. Association between hand-grip strength and site-specific risks of major osteoporotic fracture: Results from the Japanese Population-based Osteoporosis Cohort Study. *Maturitas* 2019;130:13-20. doi: 10.1016/j.maturitas.2019.09.008

53. Karkkainen M, Rikkonen T, Kroger H, et al. Association between functional capacity tests and fractures: An eight-year prospective population-based cohort study. *OSTEOPOROSIS INTERNATIONAL* 2008;19(8):1203-10. doi: 10.1007/s00198-008-0561-y

54. Minneci C, Mello AM, Mossello E, et al. Comparative study of four physical performance measures as predictors of death, incident disability, and falls in unselected older persons: the insufficienza Cardiaca negli Anziani Residenti a Dicomano Study. *Journal of the American Geriatrics Society* 2015;63(1):136-41.

55. Muraki S, Akune T, Ishimoto Y, et al. Risk factors for falls in a longitudinal population-based cohort study of Japanese men and women: The ROAD Study. *BONE* 2013;52(1):516-23. doi: 10.1016/j.bone.2012.10.020

56. Ooi TC, Singh DKA, Shahar S, et al. Incidence and multidimensional predictors of occasional and recurrent falls among Malaysian community-dwelling older persons. *BMC geriatrics* 2021;21(1):154-54. doi: 10.1186/s12877-021-02103-2

57. Pham T, McNeil JJ, Barker AL, et al. Longitudinal association between handgrip strength, gait speed and risk of serious falls in a community-dwelling older population. *Plos One* 2023;18(5) doi: 10.1371/journal.pone.0285530

58. Rikkonen T, Sirola J, Salovaara K, et al. Muscle strength and body composition are clinical indicators of osteoporosis. *Calcified tissue international* 2012;91:131-38.

59. Roongbenjawan N, Siriphorn A. Accuracy of modified 30-s chair-stand test for predicting falls in older adults. *Annals of Physical and Rehabilitation Medicine* 2020;63(4):309-15. doi: 10.1016/j.rehab.2019.08.003

60. Rouzi AA, Ardawi M-SM, Qari MH, et al. Risk factors for falls in a longitudinal cohort study of Saudi postmenopausal women: the Center of Excellence for Osteoporosis Research Study. *Menopause (New York, NY)* 2015;22(9):1012-20. doi: 10.1097/GME.0000000000000418

61. Schaap LA, Van Schoor NM, Lips P, et al. Associations of sarcopenia definitions, and their components, with the incidence of recurrent falling and fractures: the longitudinal aging study Amsterdam. *The Journals of Gerontology: Series A* 2018;73(9):1199-204.

62. Sogaard AJ, Magnus JH, Bjornerem A, et al. Grip strength in men and women aged 50-79 years is associated with non-vertebral osteoporotic fracture during 15 years follow-up: The Tromso Study 1994-1995. *OSTEOPOROSIS INTERNATIONAL* 2020;31(1):131-40. doi: 10.1007/s00198-019-05191-4

63. Stel VS, Smit JH, Pluijm SM, et al. Balance and mobility performance as treatable risk factors for recurrent falling in older persons. *Journal of clinical epidemiology* 2003;56(7):659-68.

64. Stel V, Pluijm S, Deeg D, et al. Functional limitations and poor physical performance as independent risk factors for self-reported fractures in older persons. *Osteoporosis international* 2004;15:742-50.

65. Valenzuela PL, Maffiuletti NA, Saner H, et al. Isometric strength measures are superior to the timed up and go test for fall prediction in older adults: results from a prospective cohort study. *Clinical interventions in aging* 2020:2001-08.

66. Wang Y, Li Z, Li M, et al. The relationship between asymmetrical grip strength criteria and fall incidence among middle-aged and older Chinese adults. *Eur Geriatr Med* 2024 doi: 10.1007/s41999-024-01011-z

67. Welmer A-K, Rizzuto D, Laukka EJ, et al. Cognitive and Physical Function in Relation to the Risk of Injurious Falls in Older Adults: A Population-Based Study. *The journals of gerontology Series A, Biological sciences and medical sciences* 2017;72(5):669-75. doi: 10.1093/gerona/glw141

68. Yan LJ, Ge HY, Wang ZM, et al. Roles of low muscle strength and sarcopenic obesity on incident symptomatic knee osteoarthritis: A longitudinal cohort study. *Plos One* 2024;19(10) doi: 10.1371/journal.pone.0311423

69. Zhang F, Ferrucci L, Culham E, et al. Performance on five times sit-to-stand task as a predictor of subsequent falls and disability in older persons. *Journal of aging and health* 2013;25(3):478-92.

70. Zhong B-X, Zhong H-L, Zhou G-Q, et al. Physical performance and risk of hip fracture in community-dwelling elderly people in China: a 4-year longitudinal cohort study. *Maturitas* 2021;146:26-33.

71. Zhou R, Li JY, Chen ML. The Value of Cognitive and Physical Function Tests in Predicting Falls in Older Adults: A Prospective Study. *Frontiers in Medicine* 2022;9 doi: 10.3389/fmed.2022.900488

72. Snih SA, Markides KS, Ottenbacher KJ, et al. Hand grip strength and incident ADL disability in elderly Mexican Americans over a seven-year period. *Aging clinical and experimental research* 2004;16:481-86.

73. Burbank CM, Branscum A, Bovbjerg ML, et al. Muscle power predicts frailty status over four years: A retrospective cohort study of the National Health and Aging Trends Study. *Journal of frailty, sarcopenia and falls* 2023;8(1):1-8. doi: 10.22540/JFSF-08-001

74. Carrière I, Colvez A, Favier F, et al. Hierarchical components of physical frailty predicted incidence of dependency in a cohort of elderly women. *Journal of clinical epidemiology* 2005;58(11):1180-87.

75. Cesari M, Kritchevsky SB, Newman AB, et al. Added value of physical performance measures in predicting adverse health‐related events: results from the Health, Aging and Body Composition Study. *Journal of the American Geriatrics Society* 2009;57(2):251-59.

76. Chan OYA, van Houwelingen AH, Gussekloo J, et al. Comparison of quadriceps strength and handgrip strength in their association with health outcomes in older adults in primary care. *Age* 2014;36:1-13.

77. Coelho-Júnior HJ, Calvani R, Álvarez-Bustos A, et al. Physical performance and negative events in very old adults: a longitudinal study examining the ilSIRENTE cohort. *Aging Clinical & Experimental Research* 2024;36(1):1-12. doi: 10.1007/s40520-024-02693-y

78. Dai SJ, Wang SH, Jiang SY, et al. Bidirectional association between handgrip strength and ADLs disability: a prospective cohort study. *Frontiers in Public Health* 2023;11 doi: 10.3389/fpubh.2023.1200821

79. den Ouden MEM, Schuurmans MJ, Brand JS, et al. Physical functioning is related to both an impaired physical ability and ADL disability: a ten year follow-up study in middle-aged and older persons. *Maturitas* 2013;74(1):89-94. doi: 10.1016/j.maturitas.2012.10.011

80. Dodds RM, Kuh D, Sayer AA, et al. Can measures of physical performance in mid-life improve the clinical prediction of disability in early old age? Findings from a British birth cohort study. *EXPERIMENTAL GERONTOLOGY* 2018;110:118-24. doi: 10.1016/j.exger.2018.06.001

81. Giampaoli S, Ferrucci L, Cecchi F, et al. Hand-grip strength predicts incident disability in non-disabled older men. *Age and ageing* 1999;28(3):283-88.

82. Gill TM, Williams CS, Tinetti ME. Assessing risk for the onset of functional dependence among older adults: the role of physical performance. *Journal of the American Geriatrics Society* 1995;43(6):603-09.

83. Gonzalez-Bautista E, de Souto Barreto P, Salinas-Rodriguez A, et al. Development and validation of a cutoff for the chair stand test as a screening for mobility impairment in the context of the integrated care for older people program. *The Journals of Gerontology: Series A* 2023;78(1):104-10.

84. Hicks GE, Shardell M, Alley DE, et al. Absolute strength and loss of strength as predictors of mobility decline in older adults: the InCHIANTI study. *Journals of Gerontology: Series A: Biomedical Sciences and Medical Sciences* 2012;67(1):66-73.

85. Huang YC, Dong Y, Tang CM, et al. Mortality and disability risk among older adults unable to complete grip strength and physical performance tests: a population-based cohort study from China. *Bmc Public Health* 2024;24(1) doi: 10.1186/s12889-024-18258-7

86. Ishizaki T, Watanabe S, Suzuki T, et al. Predictors for functional decline among nondisabled older Japanese living in a community during a 3‐year follow‐up. *Journal of the American Geriatrics Society* 2000;48(11):1424-29.

87. Jung S, Yabushita N, Kim M, et al. Obesity and muscle weakness as risk factors for mobility limitation in community-dwelling older Japanese women: A two-year follow-up investigation. *The Journal of nutrition, health and aging* 2016;20(1):28-34.

88. Legrand D, Vaes B, Matheï C, et al. Muscle strength and physical performance as predictors of mortality, hospitalization, and disability in the oldest old. *Journal of the American Geriatrics Society* 2014;62(6):1030-38.

89. López-Teros T, Gutiérrez-Robledo L, Pérez-Zepeda M. Gait speed and handgrip strength as predictors of incident disability in Mexican older adults. *J Frailty Aging* 2014;3(2):109-12.

90. Marincolo JCS, Assumpção D, Santimaria MR, et al. Low grip strength and gait speed as markers of dependence regarding basic activities of daily living: the FIBRA study. *Einstein (Sao Paulo)* 2024;22:eAO0637. doi: 10.31744/einstein_journal/2024AO0637

91. Morera A, Calatayud J, Casaña J, et al. Handgrip strength and work limitations: A prospective cohort study of 70,820 adults aged 50 and older. *Maturitas* 2023;177 doi: 10.1016/j.maturitas.2023.107798

92. Okabe T, Abe Y, Tomita Y, et al. Age-specific risk factors for incident disability in activities of daily living among middle-aged and elderly community-dwelling Japanese women during an 8-9-year follow up: The Hizen-Oshima study. *Geriatrics & gerontology international* 2017;17(7):1096-101. doi: 10.1111/ggi.12834

93. Peterson MD, Casten K, Collins S, et al. Muscle weakness is a prognostic indicator of disability and chronic disease multimorbidity. *Experimental gerontology* 2021;152:111462.

94. Rantanen T, Guralnik JM, Foley D, et al. Midlife hand grip strength as a predictor of old age disability. *Jama* 1999;281(6):558-60.

95. Seidel D, Brayne C, Jagger C. Limitations in physical functioning among older people as a predictor of subsequent disability in instrumental activities of daily living. *Age and ageing* 2011;40(4):463-69.

96. Shinkai S, Kumagai S, Fujiwara Y, et al. Predictors for the onset of functional decline among initially non‐disabled older people living in a community during a 6‐year follow‐up. *Geriatrics & Gerontology International* 2003;3:S31-S39.

97. Sirola J, Tuppurainen M, Rikkonen T, et al. Correlates and predictors of self-rated health and ambulatory status among elderly women - Cross-sectional and 10 years population-based cohort study. *Maturitas* 2010;65(3):244-52. doi: 10.1016/j.maturitas.2009.11.014

98. Song Q, Shu X, Li Y, et al. Association of handgrip strength asymmetry and weakness with functional disability among middle-aged and older adults in China. *J Glob Health* 2024;14:04047. doi: 10.7189/jogh.14.04047

99. Stessman J, Rottenberg Y, Fischer M, et al. Handgrip strength in old and very old adults: mood, cognition, function, and mortality. *Journal of the American Geriatrics Society* 2017;65(3):526-32.

100. Taekema DG, Gussekloo J, Maier AB, et al. Handgrip strength as a predictor of functional, psychological and social health. A prospective population-based study among the oldest old. *Age and ageing* 2010;39(3):331-37.

101. Zhang L, Chen Y, Liu J, et al. Novel physical performance-based models for activities of daily living disability prediction among Chinese older community population: a nationally representative survey in China. *BMC geriatrics* 2022;22(1):267. doi: 10.1186/s12877-022-02905-y

102. Cabanas-Sánchez V, Esteban-Cornejo I, Parra-Soto S, et al. Muscle strength and incidence of depression and anxiety: findings from the UK Biobank prospective cohort study. *Journal of cachexia, sarcopenia and muscle* 2022 doi: 10.1002/jcsm.12963

103. Carvalho AF, Maes M, Solmi M, et al. Is dynapenia associated with the onset and persistence of depressive and anxiety symptoms among older adults? Findings from the Irish longitudinal study on ageing. *Aging & Mental Health* 2021;25(3):468-75.

104. Gordon BR, McDowell CP, Lyons M, et al. Associations between grip strength and generalized anxiety disorder in older adults: Results from the Irish longitudinal study on ageing. *Journal of Affective Disorders* 2019;255:136-41. doi: 10.1016/j.jad.2019.05.043

105. Bao M, Chao J, Sheng M, et al. Longitudinal association between muscle strength and depression in middle-aged and older adults: A 7-year prospective cohort study in China. *Journal of affective disorders* 2022;301:81-86.

106. Chan LLY, Delbaere K, Numbers K, et al. Poor mobility and lower limb weakness are associated with three distinct depressive symptom trajectories over 6 years in older people. *Australasian Journal on Ageing* 2024;43(2):333-42. doi: 10.1111/ajag.13273

107. Gu Y, Zhang T, Wang J, et al. Genetic risk, muscle strength and risk of incident major depressive disorder: results from the UK Biobank. *Age and ageing* 2023;52(3) doi: 10.1093/ageing/afad021

108. Hamer M, Batty GD, Kivimaki M. Sarcopenic obesity and risk of new onset depressive symptoms in older adults: English Longitudinal Study of Ageing. *International journal of obesity* 2015;39(12):1717-20.

109. Lian Y, Wang GP, Chen GQ, et al. Bidirectional Associations between Handgrip Strength and Depressive Symptoms: A Longitudinal Cohort Study. *Journal of the American Medical Directors Association* 2021;22(8):1744-+. doi: 10.1016/j.jamda.2021.04.006

110. López-Bueno R, Calatayud J, Andersen LL, et al. Dose-response association of handgrip strength and risk of depression: a longitudinal study of 115 601 older adults from 24 countries. *British Journal of Psychiatry* 2023;222(3):135-42. doi: 10.1192/bjp.2022.178

111. Luo J, Yao W, Zhang T, et al. Exploring the bidirectional associations between handgrip strength and depression in middle and older Americans. *Journal of Psychosomatic Research* 2022;152:110678.

112. McDowell CP, Gordon BR, Herring MP. Sex-related differences in the association between grip strength and depression: Results from the Irish Longitudinal Study on Ageing. *Experimental gerontology* 2018;104:147-52.

113. Mendorf S, Schönenberg A, Heimrich KG, et al. Prospective associations between hand grip strength and subsequent depressive symptoms in men and women aged 50 years and older: insights from the Survey of Health, Aging, and Retirement in Europe. *Frontiers in Medicine* 2023;10 doi: 10.3389/fmed.2023.1260371

114. Song Q, Shu X, Zhao Y, et al. Association of handgrip strength asymmetry and weakness with depression among middle-aged and older population in China: A cohort study. *Journal of Affective Disorders* 2024;363:401-08. doi: 10.1016/j.jad.2024.07.120

115. Veronese N, Stubbs B, Trevisan C, et al. Poor physical performance predicts future onset of depression in elderly people: Progetto Veneto Anziani Longitudinal Study. *Physical therapy* 2017;97(6):659-68.

116. Zhao Z, Ji C, Liu Y, et al. Higher handgrip strength predicts a lower risk of depressive symptoms in rural Chinese populations. *Journal of affective disorders* 2020;269:12-17.

117. Zheng H, He Q, Xu H, et al. Lower grip strength and insufficient physical activity can increase depressive symptoms among middle-aged and older European adults: a longitudinal study. *BMC Geriatrics* 2022;22(1):1-10. doi: 10.1186/s12877-022-03392-x

118. Balogun S, Winzenberg T, Wills K, et al. Prospective associations of low muscle mass and strength with health-related quality of life over 10-year in community-dwelling older adults. *Experimental Gerontology* 2019;118:65-71.

119. Gómez-Bruton A, López-Torres O, Gómez-Cabello A, et al. How important is current physical fitness for future quality of life? Results from an 8-year longitudinal study on older adults. *Experimental Gerontology* 2021;149:111301.

120. Gum AM, Segal-Karpas D, Avidor S, et al. Grip strength and quality of life in the second half of life: hope as a moderator. *Aging and Mental Health* 2018;22(12):1600-05. doi: 10.1080/13607863.2017.1383972

121. Alfaro-Acha A, Snih SA, Raji MA, et al. Handgrip strength and cognitive decline in older Mexican Americans. *The Journals of Gerontology Series A: Biological Sciences and Medical Sciences* 2006;61(8):859-65.

122. Auyeung TW, Lee J, Kwok T, et al. Physical frailty predicts future cognitive decline—a four-year prospective study in 2737 cognitively normal older adults. *The journal of nutrition, health and aging* 2011;15(8):690-94.

123. Boyle PA, Buchman AS, Wilson RS, et al. Association of muscle strength with the risk of Alzheimer disease and the rate of cognitive decline in community-dwelling older persons. *Archives of neurology* 2009;66(11):1339-44.

124. Buchman AS, Wilson RS, Boyle PA, et al. Grip strength and the risk of incident Alzheimer’s disease. *Neuroepidemiology* 2007;29(1-2):66-73.

125. Chen Z, Ho M, Chau PH. Handgrip strength asymmetry is associated with the risk of neurodegenerative disorders among Chinese older adults. *Journal of Cachexia Sarcopenia and Muscle* 2022;13(2):1013-23. doi: 10.1002/jcsm.12933

126. Chou M-Y, Nishita Y, Nakagawa T, et al. Role of gait speed and grip strength in predicting 10-year cognitive decline among community-dwelling older people. *BMC geriatrics* 2019;19:1-11.

127. Feng W, Chen Q, Ma M, et al. Sex-modified association between grip strength and mild cognitive impairment: a cross-sectional and follow-up study in rural China. *BMC Geriatr* 2023;23(1):710. doi: 10.1186/s12877-023-04376-1

128. Heward J, Stone L, Paddick S-M, et al. A longitudinal study of cognitive decline in rural Tanzania: rates and potentially modifiable risk factors. *International psychogeriatrics* 2018;30(9):1333-43.

129. Jeong S, Kim J. Prospective association of handgrip strength with risk of new-onset cognitive dysfunction in Korean adults: a 6-year national cohort study. *The Tohoku journal of experimental medicine* 2018;244(2):83-91.

130. Jeong S-m, Choi S, Kim K, et al. Association among handgrip strength, body mass index and decline in cognitive function among the elderly women. *BMC geriatrics* 2018;18:1-9.

131. Jiang R, Westwater ML, Noble S, et al. Associations between grip strength, brain structure, and mental health in > 40,000 participants from the UK Biobank. *BMC Medicine* 2022;20(1):1-14. doi: 10.1186/s12916-022-02490-2

132. Kang M, Lee I, Hong H, et al. Predictors of changes in cognitive function in older Korean adults: The 2006–2018 Korean longitudinal study of aging. *International Journal of Environmental Research and Public Health* 2021;18(12):6345.

133. Kim JH. Effect of grip strength on mental health. *JOURNAL OF AFFECTIVE DISORDERS* 2019;245:371-76. doi: 10.1016/j.jad.2018.11.017

134. Kim K, Kim H. Handgrip Strength and Cognitive Function among Elderly Koreans: Insights from the Korean Longitudinal Study of Ageing. *International journal of environmental research and public health* 2022;19(9) doi: 10.3390/ijerph19095262

135. Kim GR, Sun J, Han M, et al. Evaluation of the directional relationship between handgrip strength and cognitive function: the Korean Longitudinal Study of Ageing. *academicoupcom* 2019

136. Kim KH, Park SK, Lee DR, et al. The relationship between handgrip strength and cognitive function in elderly Koreans over 8 years: a prospective population-based study using Korean longitudinal study of ageing. *Korean journal of family medicine* 2019;40(1):9.

137. McGrath R, Lee D-C, Kraemer W, et al. Weakness is associated with time to incident chronic heart failure in aging Americans. *The journal of nutrition, health & aging* 2019;24:16-19.

138. McGrath R, Robinson-Lane SG, Cook S, et al. Handgrip strength is associated with poorer cognitive functioning in aging Americans. *Journal of Alzheimer's Disease* 2019;70(4):1187-96.

139. Peng TC, Chiou JM, Chen TF, et al. Grip Strength and Sarcopenia Predict 2-Year Cognitive Impairment in Community-Dwelling Older Adults. *Journal of the American Medical Directors Association* 2023;24(3):292-+. doi: 10.1016/j.jamda.2022.10.015

140. Peng TC, Chiou JM, Chen YC, et al. Handgrip strength asymmetry and cognitive impairment risk: Insights from a seven-year prospective cohort study. *Journal of Nutrition Health & Aging* 2024;28(1) doi: 10.1016/j.jnha.2023.100004

141. Sattler C, Erickson KI, Toro P, et al. Physical fitness as a protective factor for cognitive impairment in a prospective population-based study in Germany. *Journal of Alzheimer's Disease* 2011;26(4):709-18.

142. Stijntjes M, Aartsen MJ, Taekema DG, et al. Temporal relationship between cognitive and physical performance in middle-aged to oldest old people. *Journals of Gerontology Series A: Biomedical Sciences and Medical Sciences* 2017;72(5):662-68.

143. Veronese N, Stubbs B, Trevisan C, et al. What physical performance measures predict incident cognitive decline among intact older adults? A 4.4 year follow up study. *Experimental gerontology* 2016;81:110-18.

144. Werneck AO, Araujo RHO, Silva DR, et al. Handgrip strength, physical activity and incident mild cognitive impairment and dementia. *Maturitas* 2023;176:107789. doi: 10.1016/j.maturitas.2023.107789

145. Doi T, Tsutsumimoto K, Nakakubo S, et al. Physical performance predictors for incident dementia among Japanese community-dwelling older adults. *Physical Therapy* 2019;99(9):1132-40.

146. Duchowny KA, Ackley SF, … WDBJN, et al. Associations Between Handgrip Strength and Dementia Risk, Cognition, and Neuroimaging Outcomes in the UK Biobank Cohort Study. *jamanetworkcom* 2022

147. Esteban-Cornejo I, Ho FK, Petermann-Rocha F, et al. Handgrip strength and all-cause dementia incidence and mortality: findings from the UK Biobank prospective cohort study. *Journal of cachexia, sarcopenia and muscle* 2022;13(3):1514-25. doi: 10.1002/jcsm.12857

148. Hatabe Y, Shibata M, Ohara T, et al. Decline in handgrip strength from midlife to late-life is associated with dementia in a Japanese community: the Hisayama Study. *Journal of epidemiology* 2020;30(1):15-23.

149. He P, Zhou C, Ye Z, et al. Walking pace, handgrip strength, age, APOE genotypes, and new-onset dementia: the UK Biobank prospective cohort study. *Alzheimer's research & therapy* 2023;15(1):1-10.

150. Kuo K, Zhang YR, Chen SD, et al. Associations of grip strength, walking pace, and the risk of incident dementia: A prospective cohort study of 340212 participants. *Alzheimers & Dementia* 2023;19(4):1415-27. doi: 10.1002/alz.12793

151. Sibbett RA, Russ TC, Allerhand M, et al. Physical fitness and dementia risk in the very old: a study of the Lothian Birth Cohort 1921. *BMC psychiatry* 2018;18:1-11.

152. Stephan Y, Sutin AR, Luchetti M, et al. Balance, Strength, and Risk of Dementia: Findings From the Health and Retirement Study and the English Longitudinal Study of Ageing. *Journals of Gerontology Series a-Biological Sciences and Medical Sciences* 2024;79(8) doi: 10.1093/gerona/glae165

153. Liu MY, He PP, Ye ZL, et al. Association of handgrip strength and walking pace with incident Parkinson's disease. *Journal of Cachexia Sarcopenia and Muscle* 2024;15(1):198-207. doi: 10.1002/jcsm.13366

154. Mey R, Calatayud J, Casaña J, et al. Is Handgrip Strength Associated With Parkinson's Disease? Longitudinal Study of 71 702 Older Adults. *Neurorehabilitation and Neural Repair* 2023;37(10):727-33. doi: 10.1177/15459683231207359

155. Wu KM, Kuo K, Deng YT, et al. Association of grip strength and walking pace with the risk of incident Parkinson's disease: a prospective cohort study of 422,531 participants. *J Neurol* 2024;271(5):2529-38. doi: 10.1007/s00415-024-12194-7

156. Studenski SA, Peters KW, Alley DE, et al. The FNIH sarcopenia project: rationale, study description, conference recommendations, and final estimates. *Journals of Gerontology Series A: Biomedical Sciences and Medical Sciences* 2014;69(5):547-58.
