# Supplementary material for: Clinical importance of simple muscular fitness tests to predict long-term health conditions: a systematic review and meta-analysis of 94 cohort studies
Source: Br J Sports Med. 2026 Feb 10;60(6):e109173. doi: 10.1136/bjsports-2024-109173 (PMC13018823; doi:10.1136/bjsports-2024-109173)
Supplement: online supplemental appendix 3 [file bjsports-60-6-s003.docx]

**Appendix 3. Online Supplemental Figures**


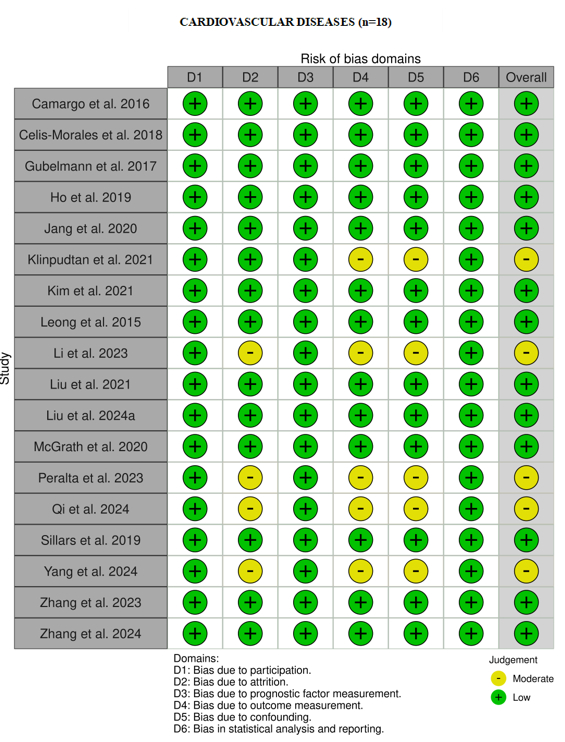


**Figure S1.** Risk of bias assessment using QUIPS tool for cardiovascular diseases.


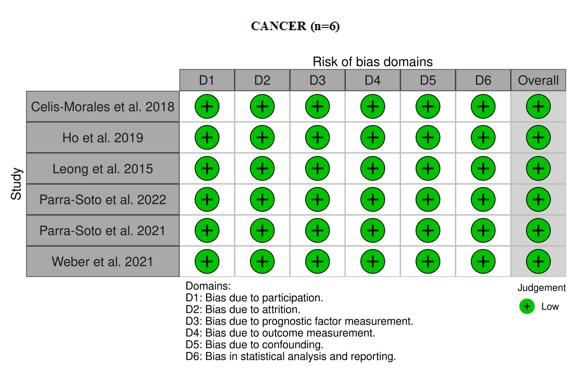


**Figure S2.** Risk of bias assessment using QUIPS tool for cancer.


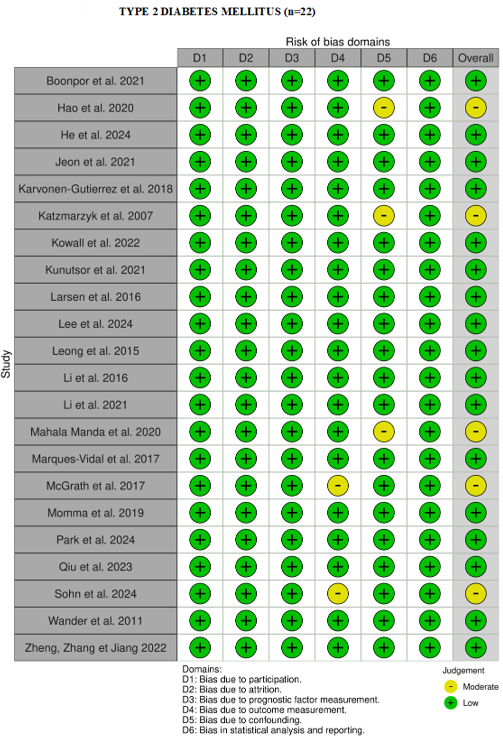


**Figure S3.** Risk of bias assessment using QUIPS tool for type 2 diabetes mellitus.


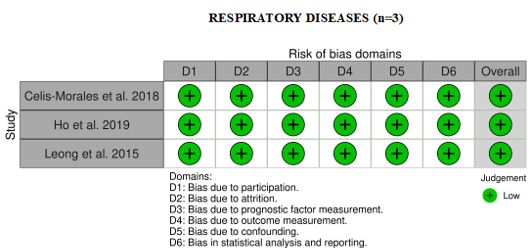


**Figure S4.** Risk of bias assessment using QUIPS tool for respiratory diseases.


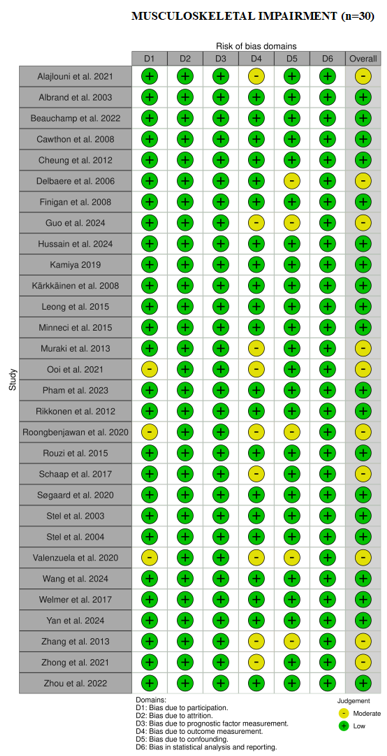


**Figure S5.** Risk of bias assessment using QUIPS tool for musculoskeletal impairment.


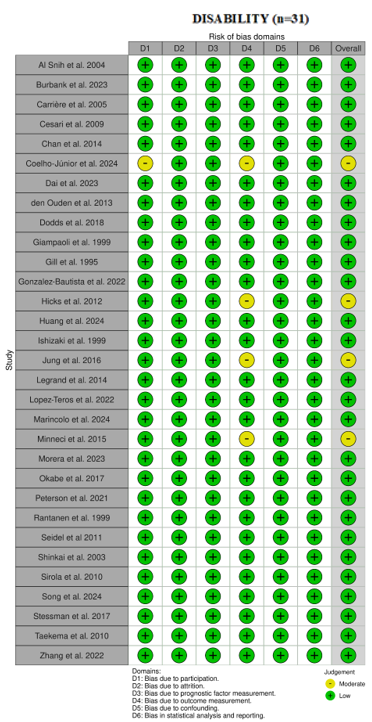


**Figure S6.** Risk of bias assessment using QUIPS tool for disability (disability activities of daily living, functional mobility, ambulatory status).


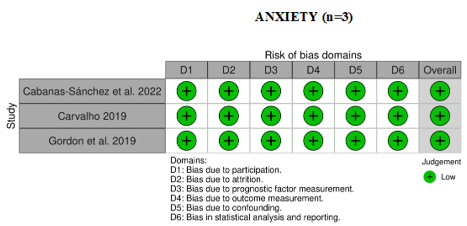


**Figure S7.** Risk of bias assessment using QUIPS tool for anxiety (diagnosed cases or subclinical symptoms).


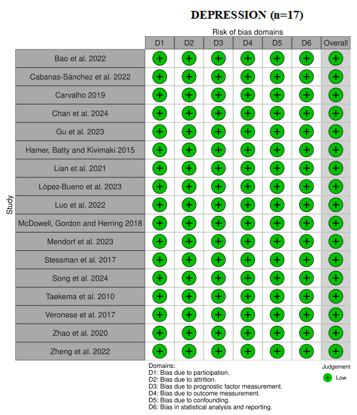


**Figure S8.** Risk of bias assessment using QUIPS tool for depression (diagnosed cases or subclinical symptoms).


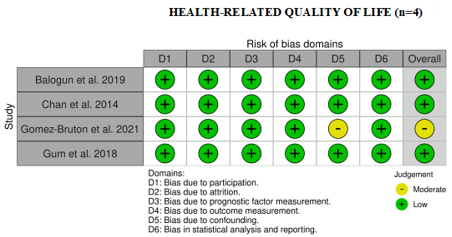


**Figure S9.** Risk of bias assessment using QUIPS tool for health-related quality of life.


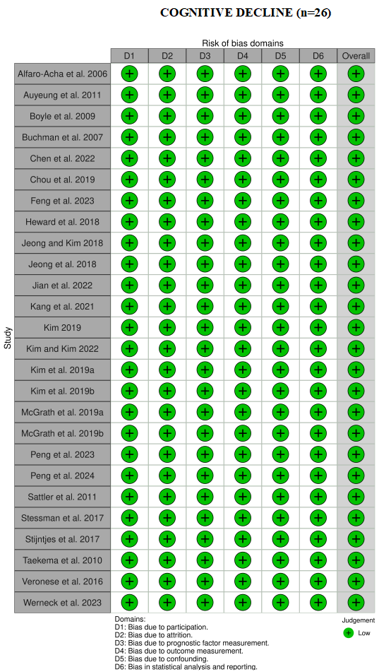


**Figure S10.** Risk of bias assessment using QUIPS tool for cognitive decline.


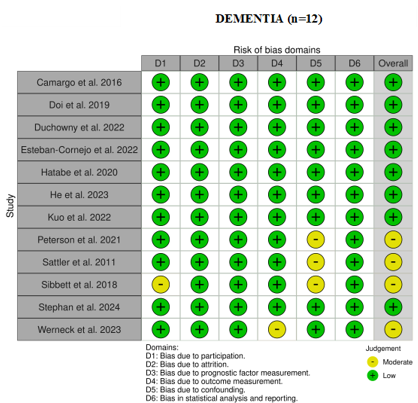


**Figure S11.** Risk of bias assessment using QUIPS tool for dementia.


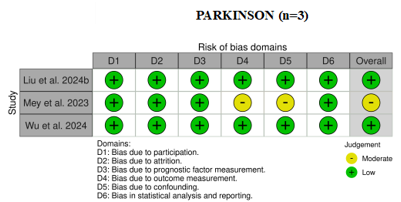


**Figure S12.** Risk of bias assessment using QUIPS tool for Parkinson.


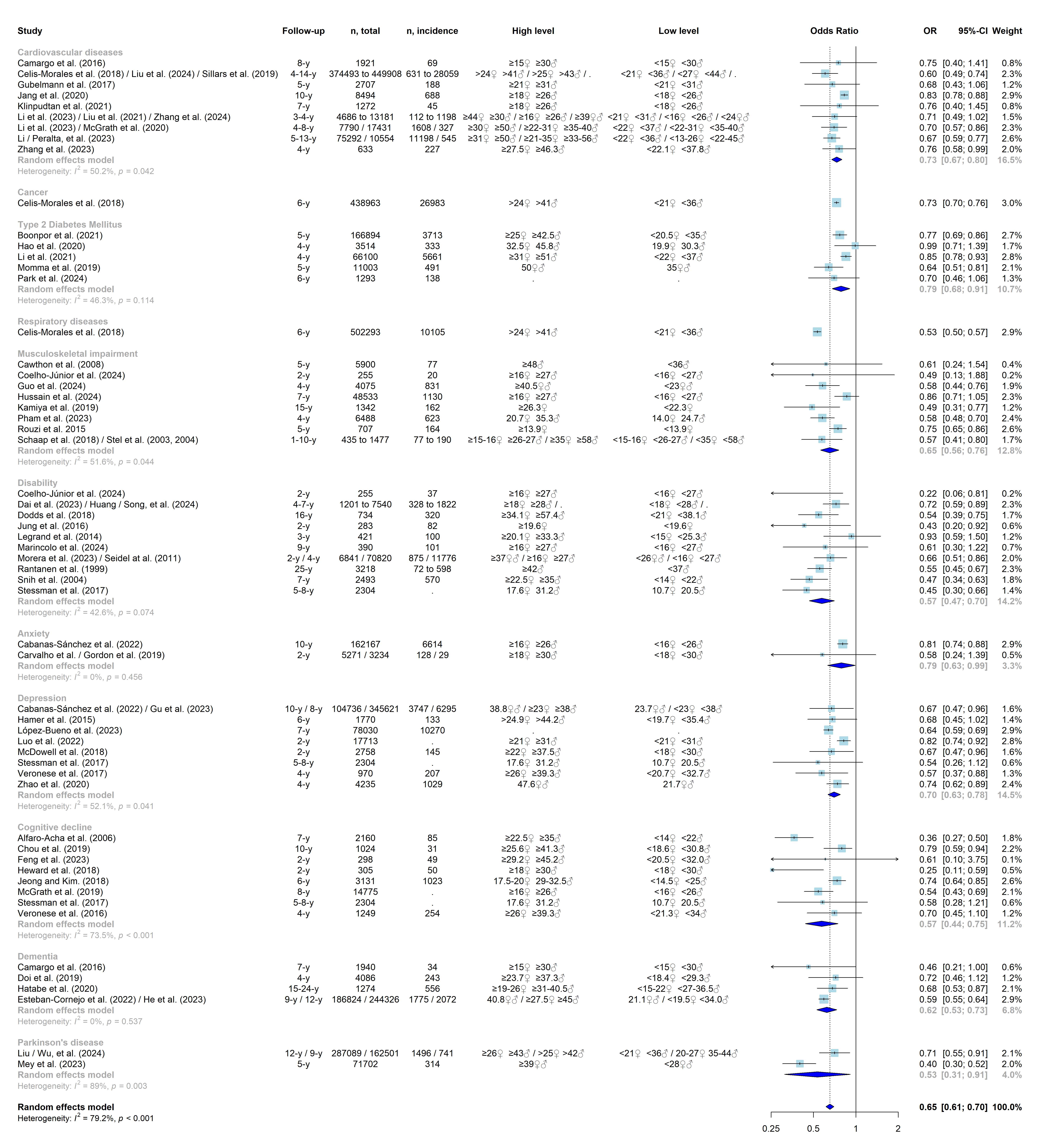


**Figure S13.** Forest plot showing the pooled odds ratios for incident long-term health conditions comparing high versus low (reference) handgrip strength, presented for each individual study and grouped by long-term health condition.

*Notes*: High handgrip strength was defined as the highest group and low handgrip strength as the lowest group (i.e., tertile 3 vs. tertile 1, quartile 4 vs. quartile 1, quintile 5 vs. quintile 1). Residual heterogeneity in this analysis may reflect differences in the cut-off values used to define handgrip strength categories across the included studies.

Long-term health conditions (**online supplemental table S2** provides the specific outcomes) were classified and defined following Medical Subject Headings (MeSH) criteria when available. **Cardiovascular Diseases, “**pathological conditions involving the cardiovascular system including the heart; the blood vessels; or the pericardium”; **Cancer (*classified as Neoplasms*),** “new abnormal growth of tissue. Malignant neoplasms show a greater degree of anaplasia and have the properties of invasion and metastasis, compared to benign neoplasms”; **Type 2 Diabetes Mellitus,** “a subclass of Diabetes Mellitus that is not insulin-responsive or dependent”; **Respiratory disease (*classified as Respiratory Tract Diseases*)**, “diseases involving the respiratory system”; **Musculoskeletal impairment *(classified as Musculoskeletal Diseases*)** “diseases of the muscles and their associated ligaments and other connective tissue and of the bones and cartilage viewed collectively”; **Disability (*classified as Mobility Limitation*)**, “difficulty in walking from place to place”; **Anxiety (clinical diagnosis or symptom severity)**, “persistent and disabling anxiety, or feelings or emotions of dread, apprehension, and impending disaster”; **Depression (clinical diagnosis or symptom severity),** “depressive states usually of moderate intensity, in contrast with major depressive disorder present in neurotic and psychotic disorders; an affective disorder manifested by either a dysphoric mood or loss of interest or pleasure in usual activities, in which the mood disturbance is prominent and relatively persistent, with major depression defined as a disorder in which five (or more) symptoms have been present during the same 2-week period and represent a change from previous functioning, with at least one symptom being depressed mood or loss of interest or pleasure”; **Cognitive decline, (*classified as*** ***Cognitive Dysfunction*)** “diminished or impaired mental and/or intellectual function”; **Dementia**, “an acquired organic mental disorder with loss of intellectual abilities of sufficient severity to interfere with social or occupational functioning. The dysfunction is multifaceted and involves memory, behaviour, personality, judgment, attention, spatial relations, language, abstract thought, and other executive functions. The intellectual decline is usually progressive and initially spares the level of consciousness”; and **Parkinson's disease,** “a progressive, degenerative neurologic disease characterized by a tremor that is maximal at rest, retropulsion (i.e. a tendency to fall backwards), rigidity, stooped posture, slowness of voluntary movements, and a masklike facial expression”.


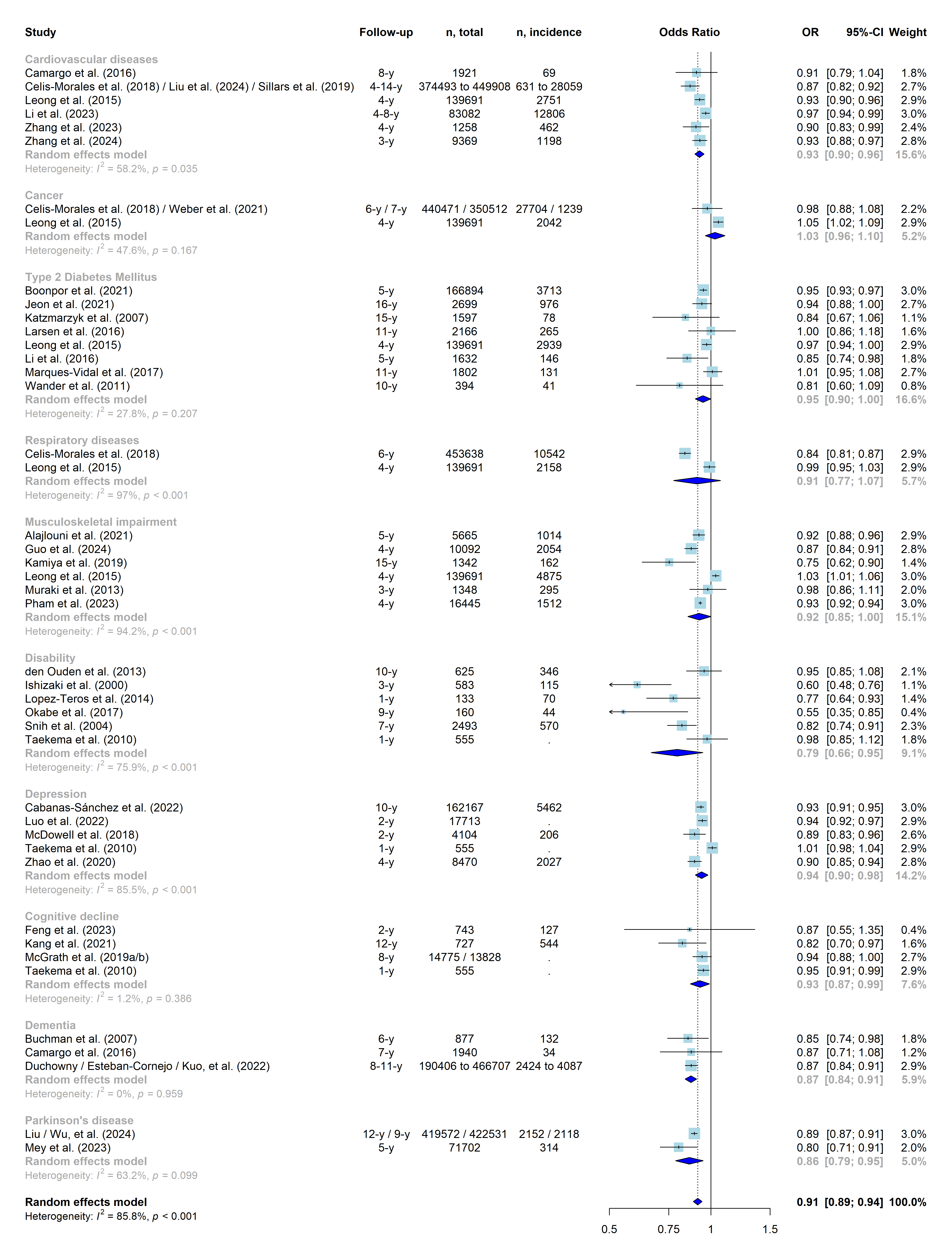


**Figure S14.** Forest plot showing the pooled odds ratios for incident long-term health conditions per 5-kg increment in handgrip strength, presented for each individual study and grouped by long-term health condition.

*Notes*: Residual heterogeneity in this analysis may reflect differences in the increment values used for handgrip strength across the included studies, for which a 5-kg increment was applied to derive comparable estimates. Long-term health conditions (**online supplemental table S2** provides the specific outcomes) were classified and defined following Medical Subject Headings (MeSH) criteria when available. **Cardiovascular Diseases, “**pathological conditions involving the cardiovascular system including the heart; the blood vessels; or the pericardium”; **Cancer (*classified as Neoplasms*),** “new abnormal growth of tissue. Malignant neoplasms show a greater degree of anaplasia and have the properties of invasion and metastasis, compared to benign neoplasms”; **Type 2 Diabetes Mellitus,** “a subclass of Diabetes Mellitus that is not insulin-responsive or dependent”; **Respiratory disease (*classified as Respiratory Tract Diseases*)**, “diseases involving the respiratory system”; **Musculoskeletal impairment *(classified as Musculoskeletal Diseases*)** “diseases of the muscles and their associated ligaments and other connective tissue and of the bones and cartilage viewed collectively”; **Disability (*classified as Mobility Limitation*)**, “difficulty in walking from place to place”; **Depression (clinical diagnosis or symptom severity),** “depressive states usually of moderate intensity, in contrast with major depressive disorder present in neurotic and psychotic disorders; an affective disorder manifested by either a dysphoric mood or loss of interest or pleasure in usual activities, in which the mood disturbance is prominent and relatively persistent, with major depression defined as a disorder in which five (or more) symptoms have been present during the same 2-week period and represent a change from previous functioning, with at least one symptom being depressed mood or loss of interest or pleasure”; **Cognitive decline, (*classified as*** ***Cognitive Dysfunction*)** “diminished or impaired mental and/or intellectual function”; **Dementia**, “an acquired organic mental disorder with loss of intellectual abilities of sufficient severity to interfere with social or occupational functioning. The dysfunction is multifaceted and involves memory, behaviour, personality, judgment, attention, spatial relations, language, abstract thought, and other executive functions. The intellectual decline is usually progressive and initially spares the level of consciousness”; and **Parkinson's disease,** “a progressive, degenerative neurologic disease characterized by a tremor that is maximal at rest, retropulsion (i.e. a tendency to fall backwards), rigidity, stooped posture, slowness of voluntary movements, and a masklike facial expression”.


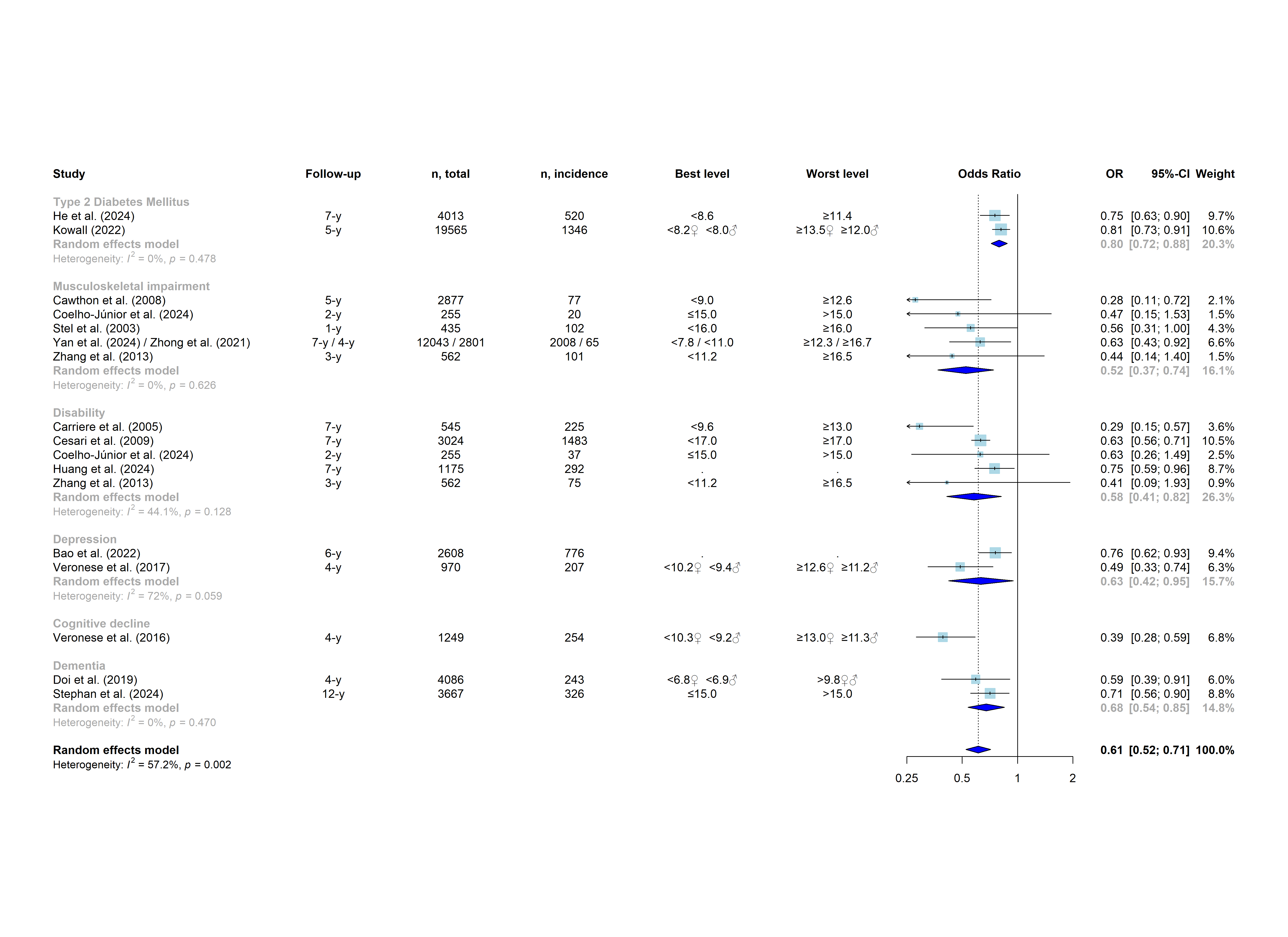


**Figure S15.** Forest plot showing the pooled odds ratios for incident long-term health conditions comparing the best versus the worst (reference) category of 5-repetition chair-stand performance, presented for each individual study and grouped by long-term health condition.

*Notes*: Best 5-repetition chair-stand performance was defined as the highest group and worst 5-repetition chair-stand performance as the lowest group (i.e., tertile 3 vs. tertile 1, quartile 4 vs. quartile 1, quintile 5 vs. quintile 1). Residual heterogeneity in this analysis may reflect differences in the cut-off values used to define chair-stand performance categories across the included studies.

Long-term health conditions (**online supplemental table S2** provides the specific outcomes) were classified and defined following Medical Subject Headings (MeSH) criteria when available. **Type 2 Diabetes Mellitus,** “a subclass of Diabetes Mellitus that is not insulin-responsive or dependent”; **Musculoskeletal impairment *(classified as Musculoskeletal Diseases*)** “diseases of the muscles and their associated ligaments and other connective tissue and of the bones and cartilage viewed collectively”; **Disability (*classified as Mobility Limitation*)**, “difficulty in walking from place to place”; **Depression (clinical diagnosis or symptom severity),** “depressive states usually of moderate intensity, in contrast with major depressive disorder present in neurotic and psychotic disorders; an affective disorder manifested by either a dysphoric mood or loss of interest or pleasure in usual activities, in which the mood disturbance is prominent and relatively persistent, with major depression defined as a disorder in which five (or more) symptoms have been present during the same 2-week period and represent a change from previous functioning, with at least one symptom being depressed mood or loss of interest or pleasure”; **Cognitive decline, (*classified as*** ***Cognitive Dysfunction*)** “diminished or impaired mental and/or intellectual function”; and **Dementia**, “an acquired organic mental disorder with loss of intellectual abilities of sufficient severity to interfere with social or occupational functioning. The dysfunction is multifaceted and involves memory, behaviour, personality, judgment, attention, spatial relations, language, abstract thought, and other executive functions. The intellectual decline is usually progressive and initially spares the level of consciousness”.


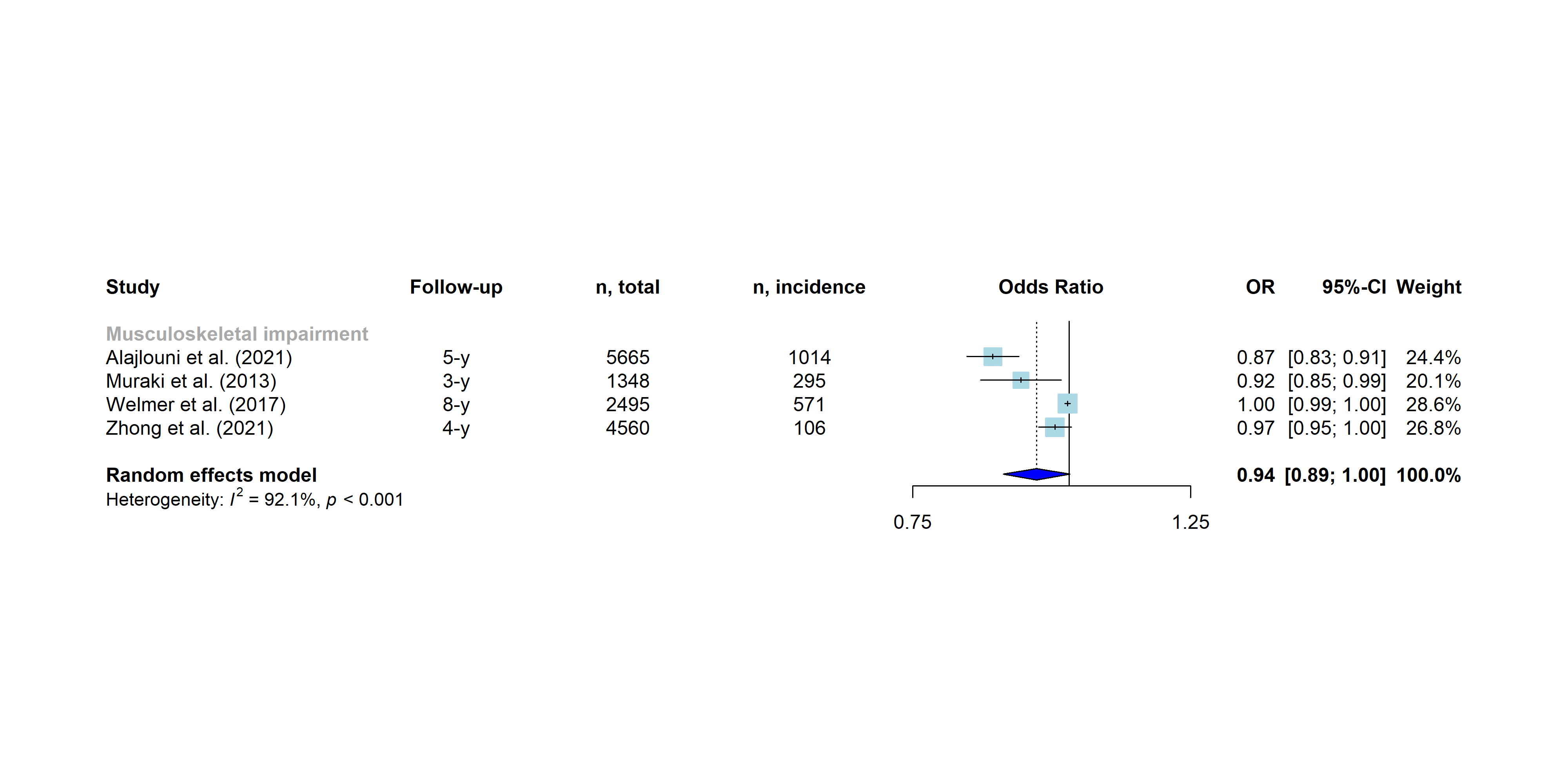


**Figure S16.** Forest plot showing the pooled odds ratios for incident long-term health conditions per 1-second decrease in the 5-repetition chair-stand test, presented for each individual study and grouped by long-term health condition.

*Notes*: Residual heterogeneity in this analysis may reflect differences in the decrease values used for chair-stand performance across the included studies, for which a 1-s decrease was applied to derive comparable estimates. Long-term health conditions (**online supplemental table S2** provides the specific outcomes) were classified and defined following Medical Subject Headings (MeSH) criteria when available. **Musculoskeletal impairment *(classified as Musculoskeletal Diseases*)** “Diseases of the muscles and their associated ligaments and other connective tissue and of the bones and cartilage viewed collectively”.


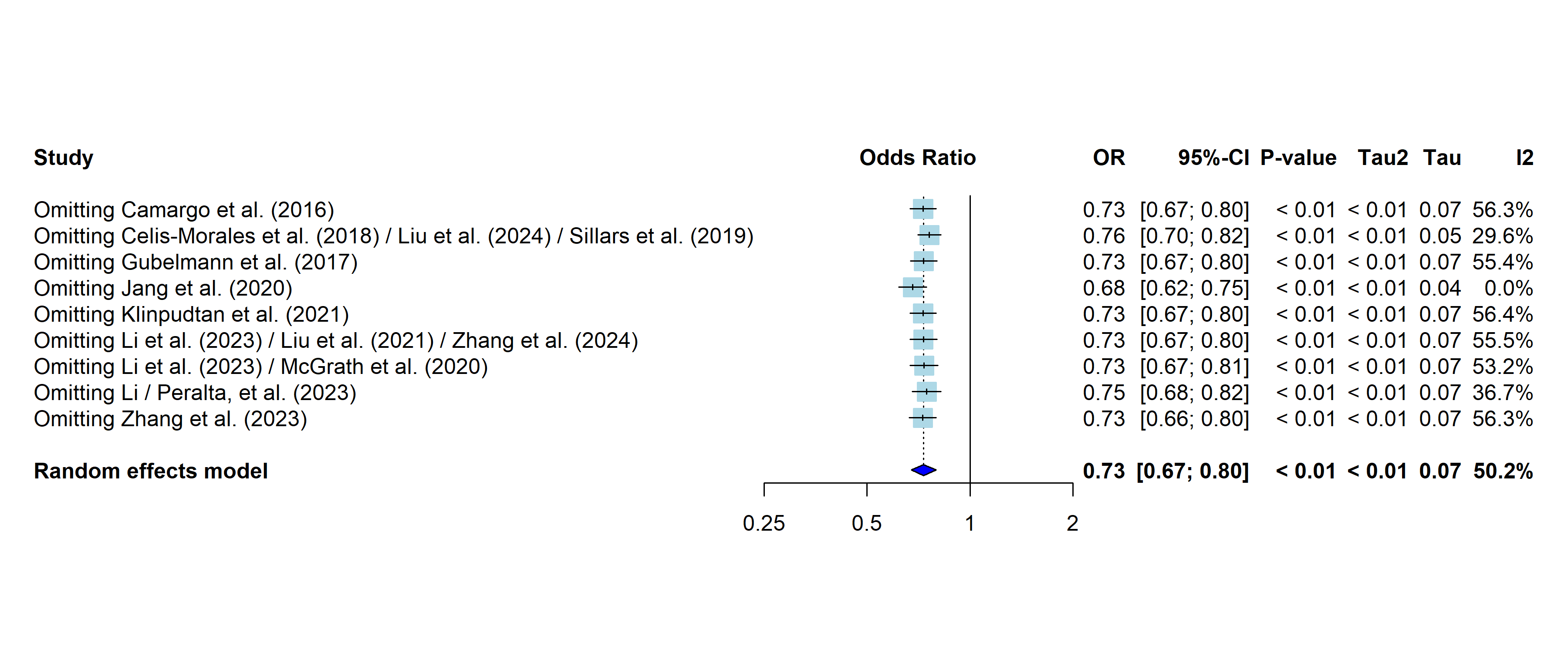


**Figure S17.** Sensitivity analysis (leave-one-out method) of the pooled odds ratios for the association between handgrip strength (highest vs. lowest category) and incident cardiovascular disease.


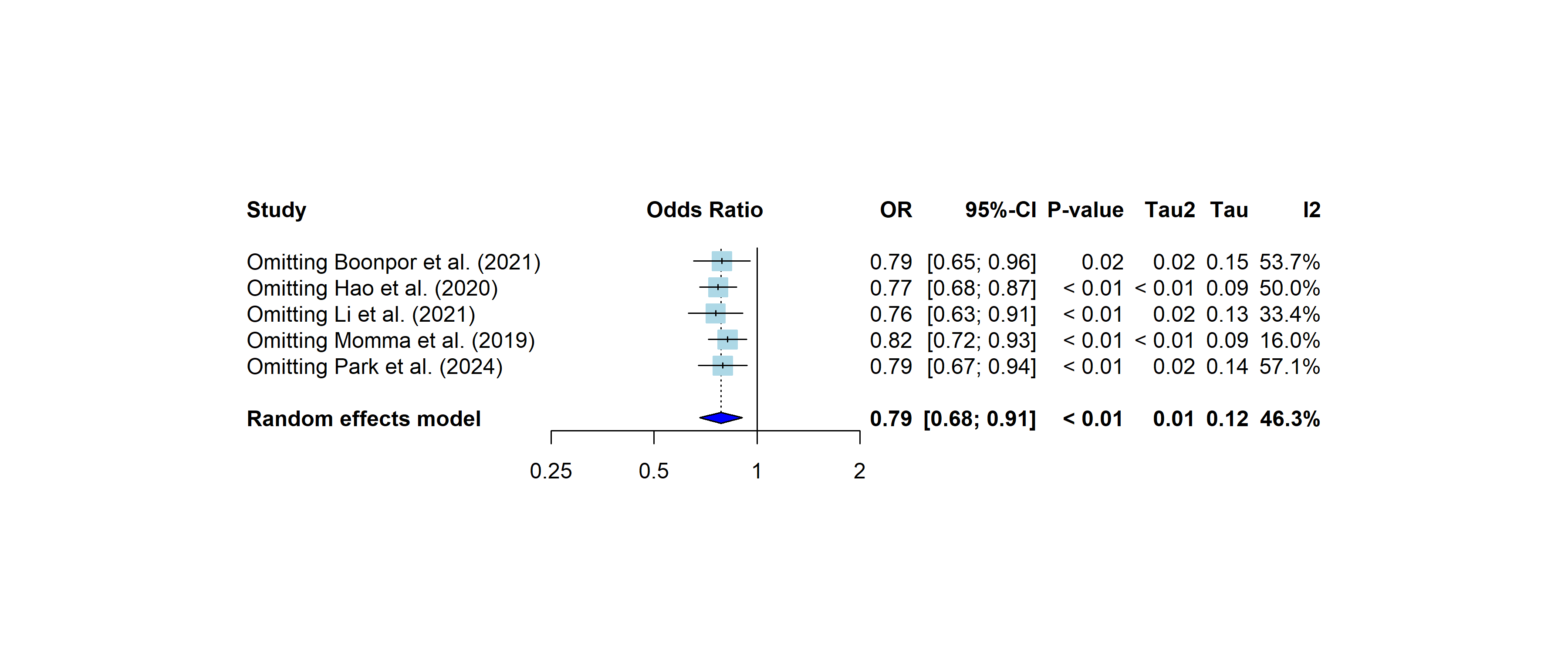


**Figure S18.** Sensitivity analysis (leave-one-out method) of the pooled odds ratios for the association between handgrip strength (highest vs. lowest category) and incident type 2 diabetes mellitus.


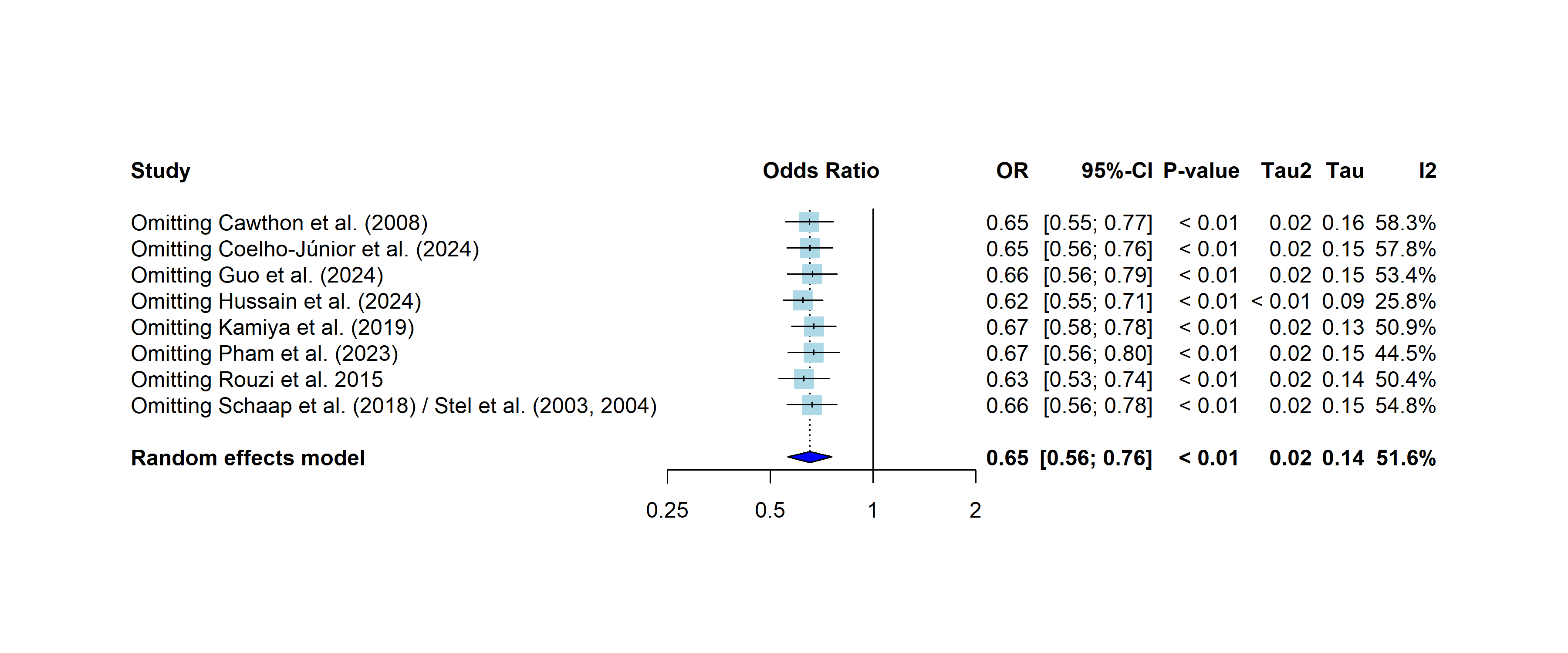


**Figure S19.** Sensitivity analysis (leave-one-out method) of the pooled odds ratios for the association between handgrip strength (highest vs. lowest category) and incident musculoskeletal impairment.


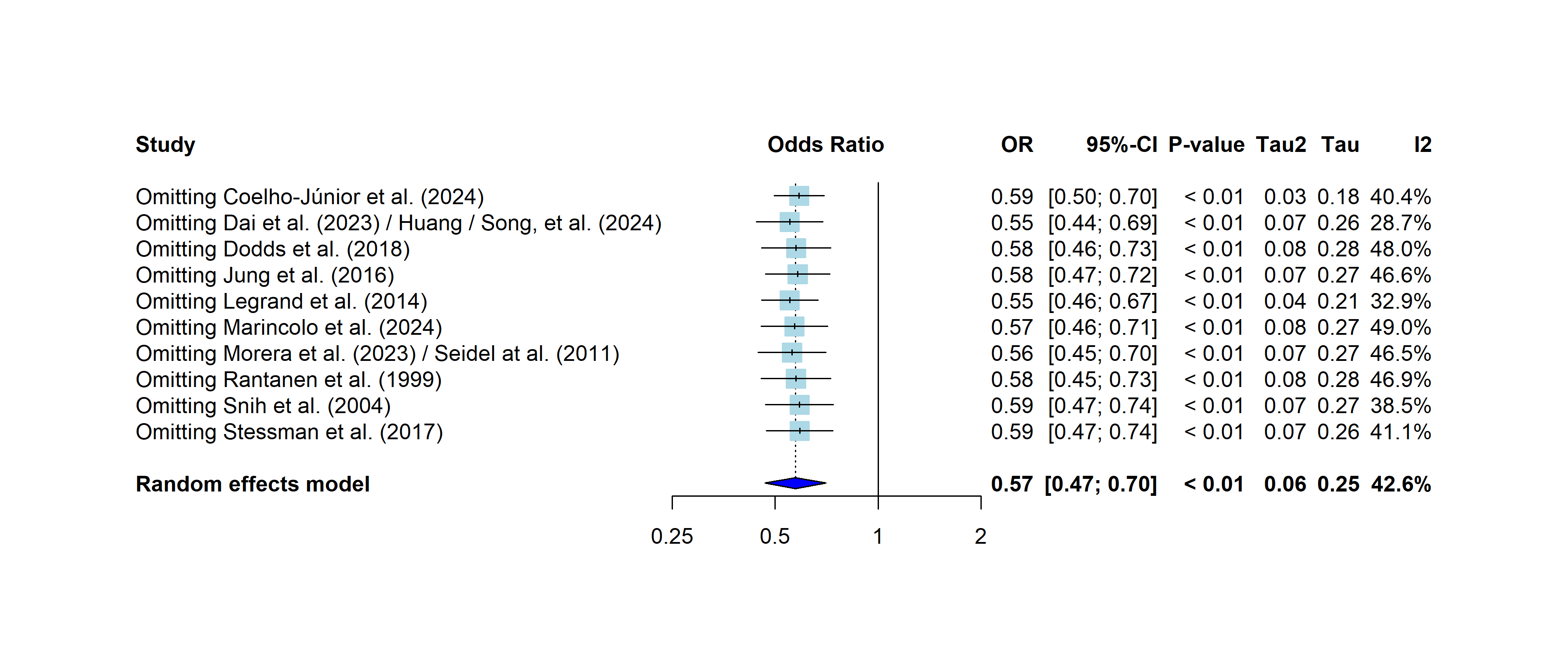


**Figure S20.** Sensitivity analysis (leave-one-out method) of the pooled odds ratios for the association between handgrip strength (highest vs. lowest category) and incident disability (disability activities of daily living, functional mobility, ambulatory status).


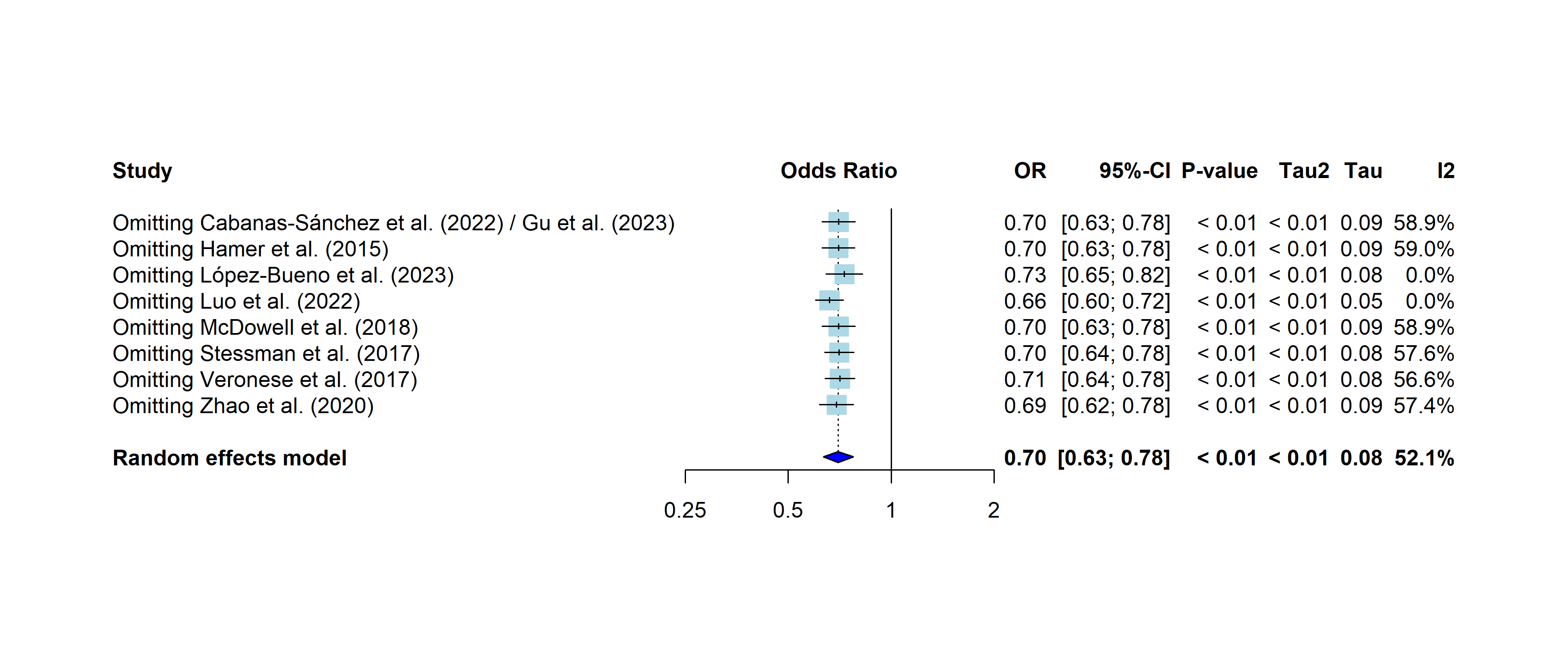


**Figure S21.** Sensitivity analysis (leave-one-out method) of the pooled odds ratios for the association between handgrip strength (highest vs. lowest category) and incident depression (clinical diagnosis or mild to severe symptoms).


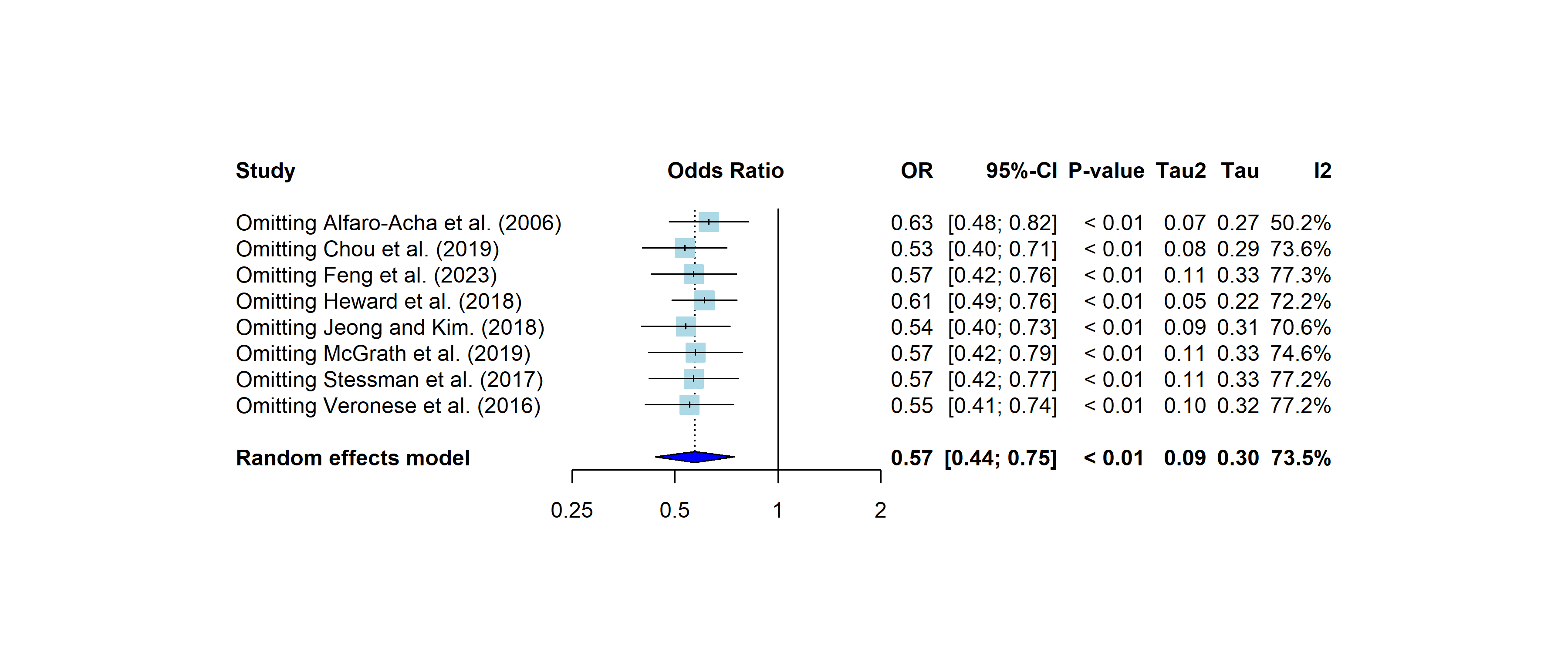


**Figure S22.** Sensitivity analysis (leave-one-out method) of the pooled odds ratios for the association between handgrip strength (highest vs. lowest category) and incident cognitive decline.


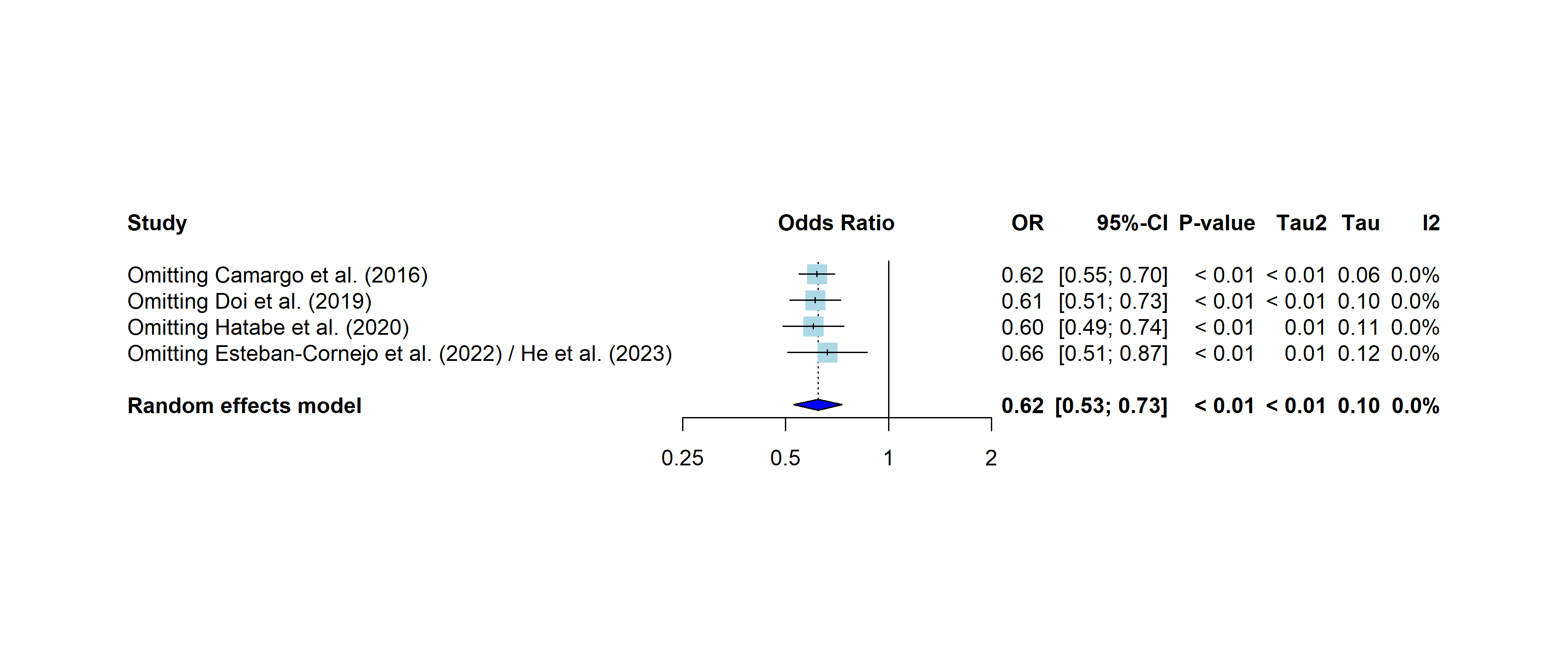


**Figure S23.** Sensitivity analysis (leave-one-out method) of the pooled odds ratios for the association between handgrip strength (highest vs. lowest category) and incident dementia.


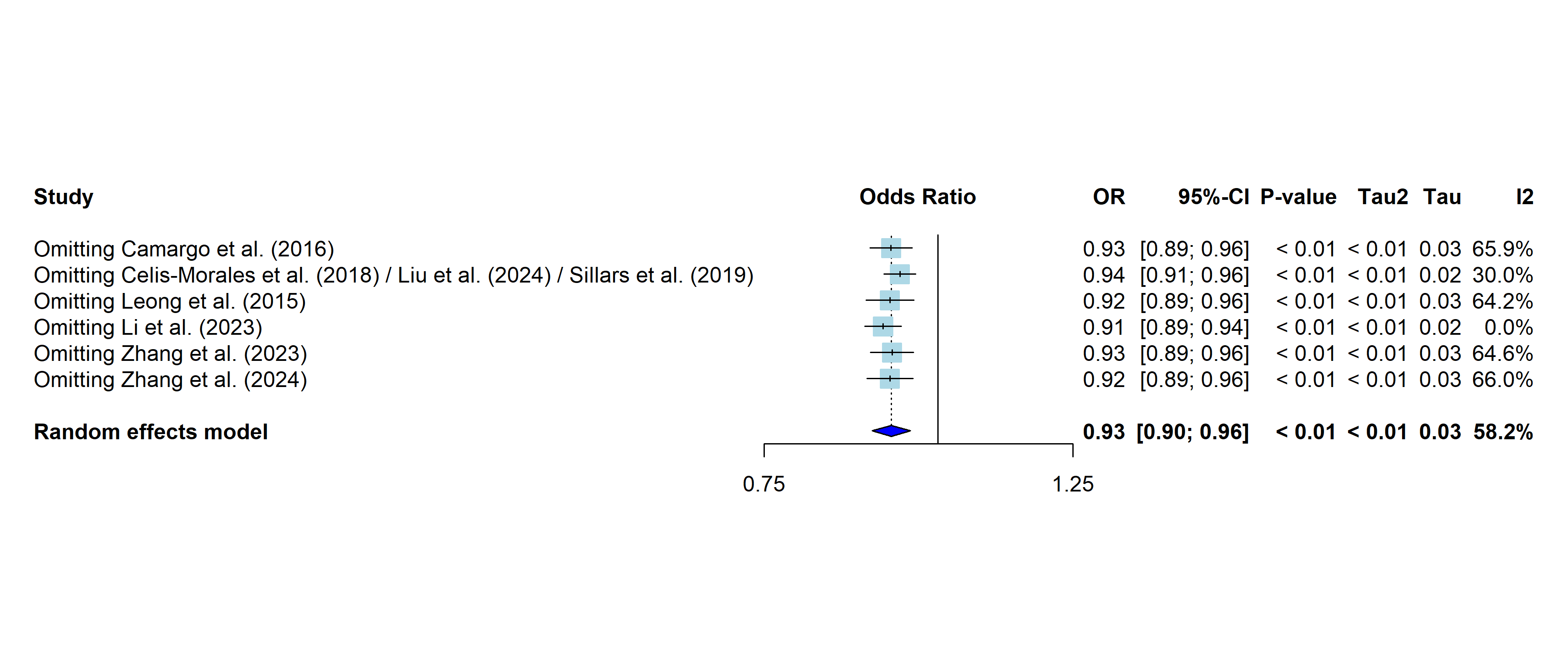


**Figure S24.** Sensitivity analysis (leave-one-out method) of the pooled odds ratios for the association between handgrip strength (per 5-kg increment) and incident cardiovascular disease.


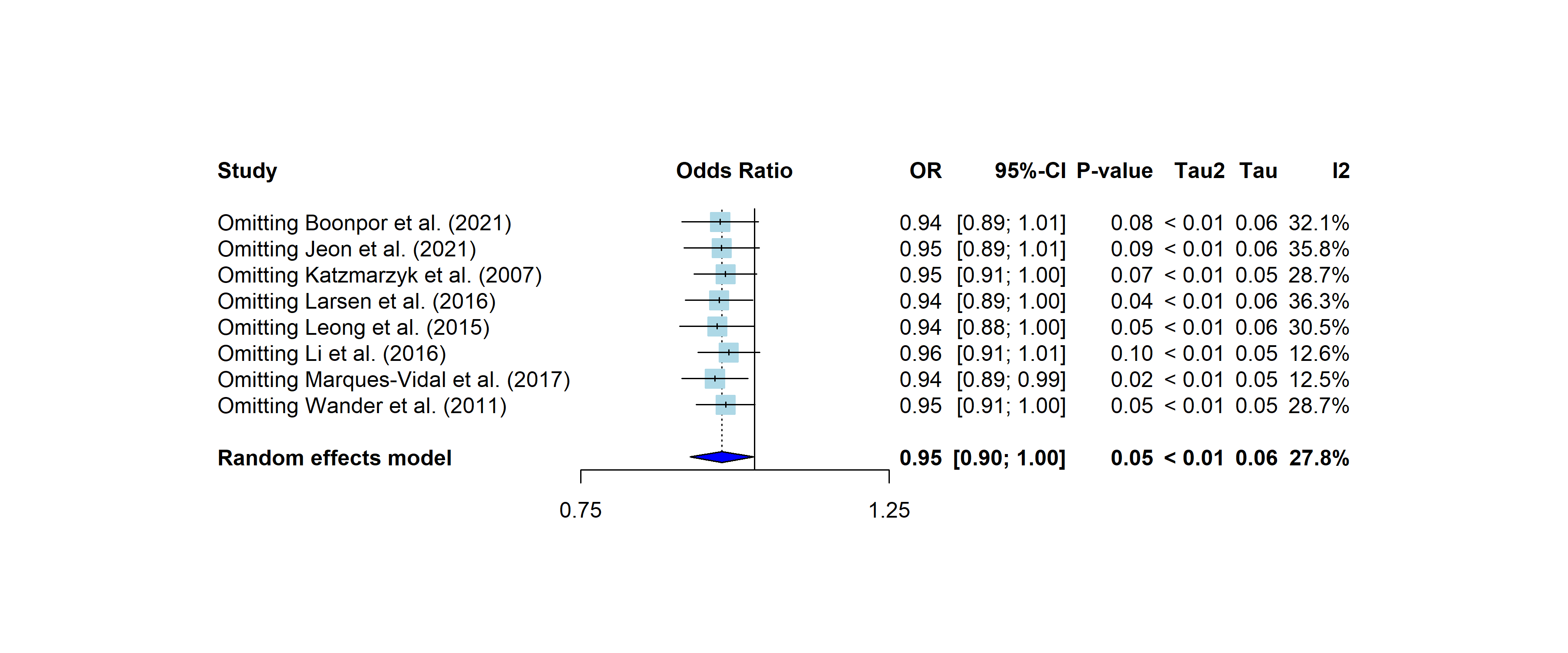


**Figure S25.** Sensitivity analysis (leave-one-out method) of the pooled odds ratios for the association between handgrip strength (per 5-kg increment) and incident type 2 diabetes mellitus.


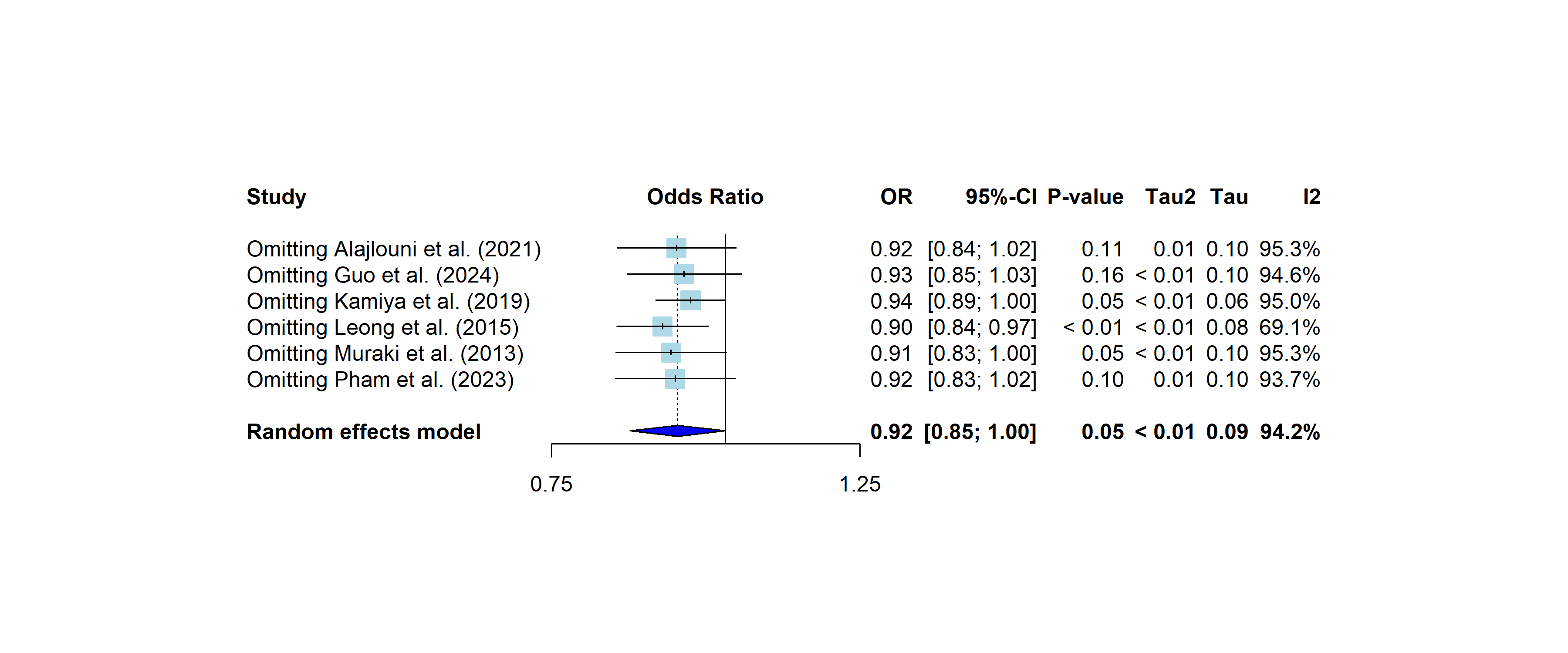


**Figure S26.** Sensitivity analysis (leave-one-out method) of the pooled odds ratios for the association between handgrip strength (per 5-kg increment) and incident musculoskeletal impairment.


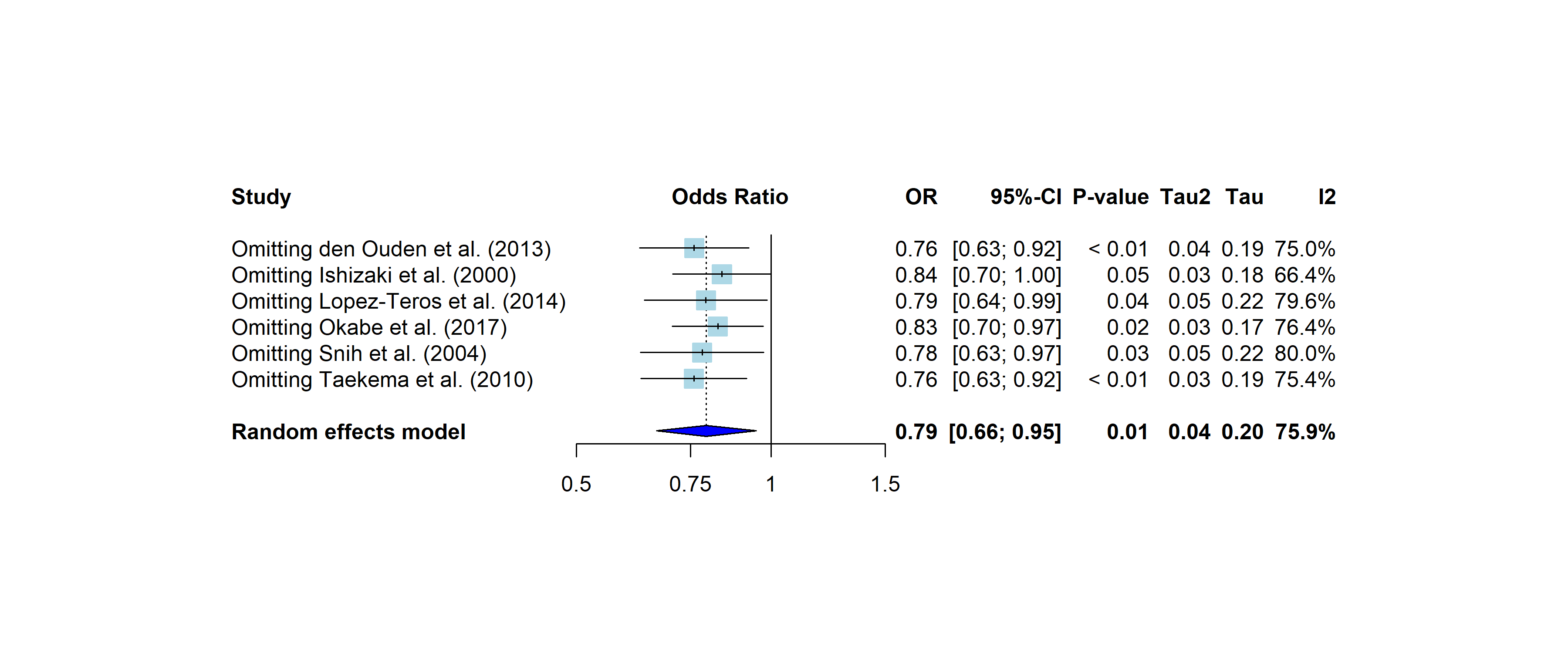


**Figure S27.** Sensitivity analysis (leave-one-out method) of the pooled odds ratios for the association between handgrip strength (per 5-kg increment) and incident disability (disability activities of daily living, functional mobility, ambulatory status).


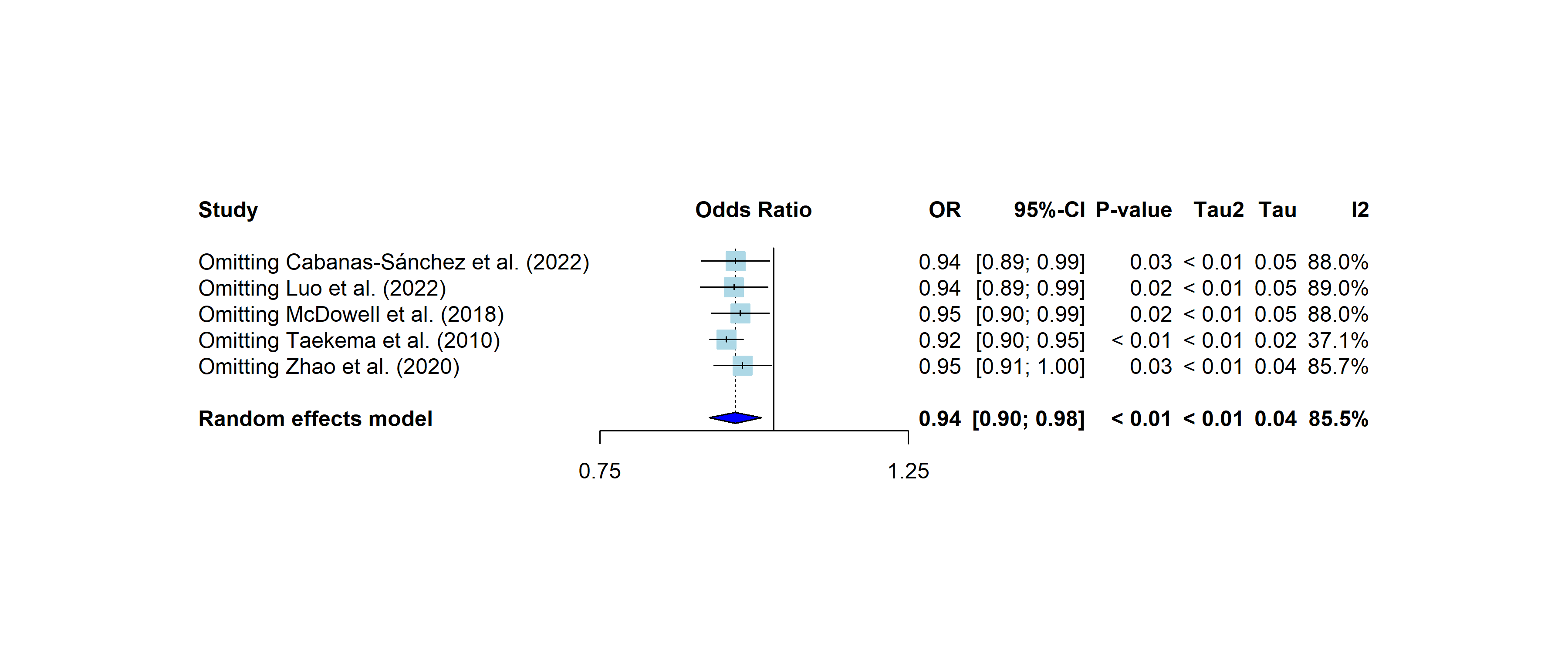


**Figure S28.** Sensitivity analysis (leave-one-out method) of the pooled odds ratios for the association between handgrip strength (per 5-kg increment) and incident depression (clinical diagnosis or mild to severe symptoms).


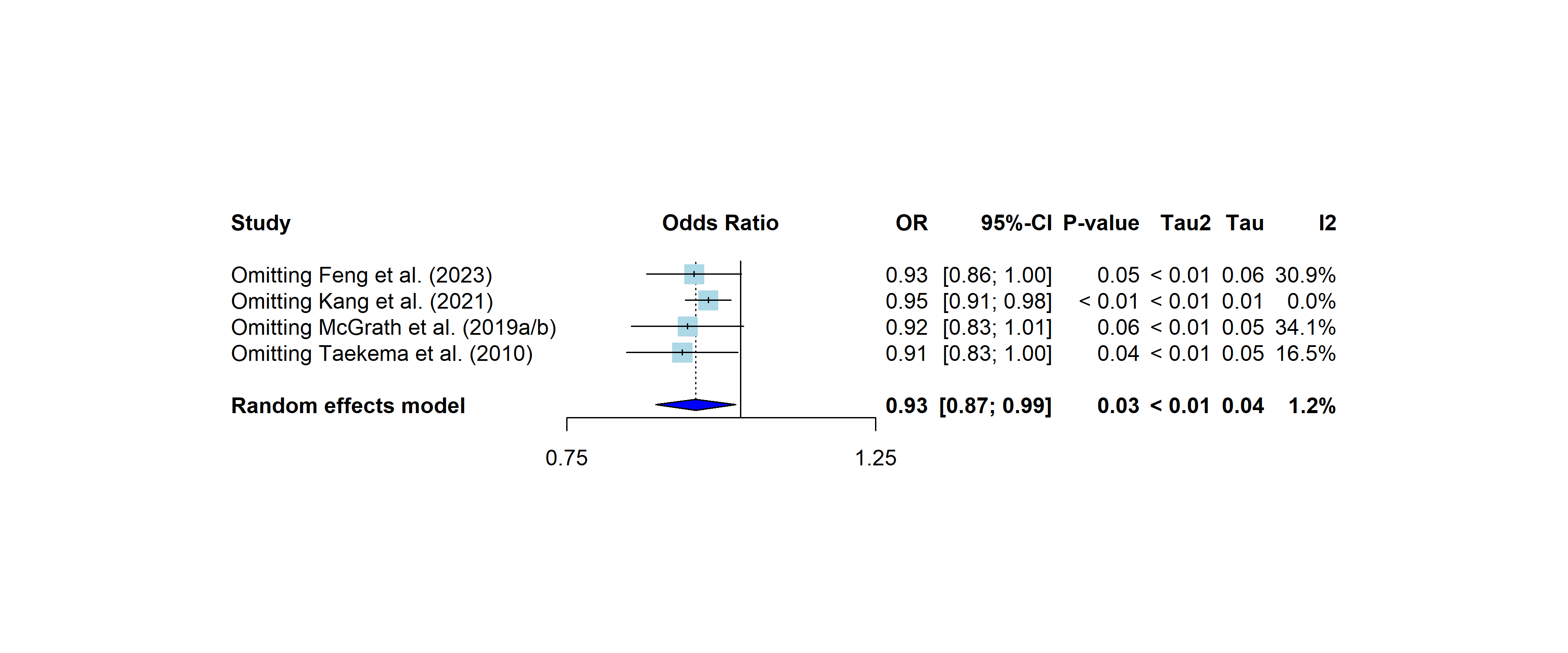


**Figure S29.** Sensitivity analysis (leave-one-out method) of the pooled odds ratios for the association between handgrip strength (per 5-kg increment) and incident cognitive decline.


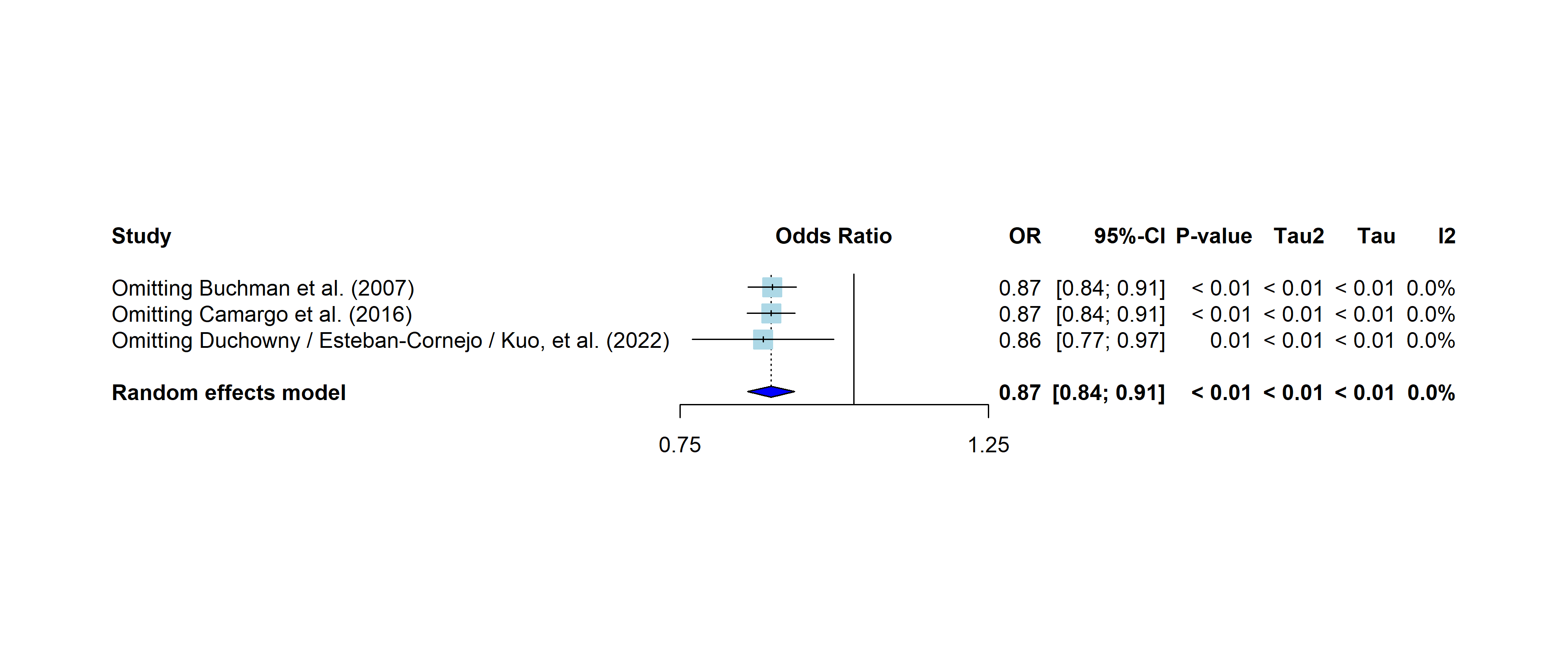


**Figure S30.** Sensitivity analysis (leave-one-out method) of the pooled odds ratios for the association between handgrip strength (per 5-kg increment) and incident dementia.


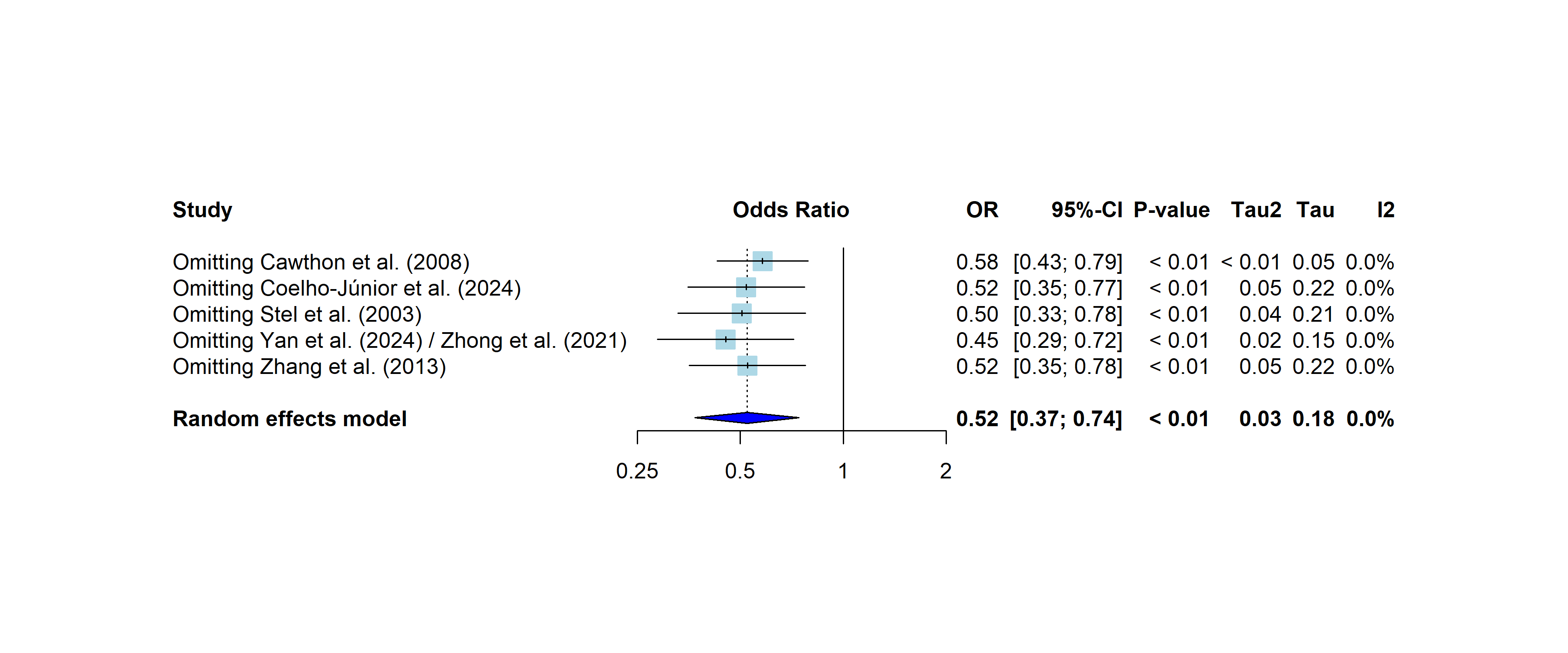


**Figure S31.** Sensitivity analysis (leave-one-out method) of the pooled odds ratios for the association between 5-repetition chair-stand performance (best vs. worst category) and incident musculoskeletal impairment.


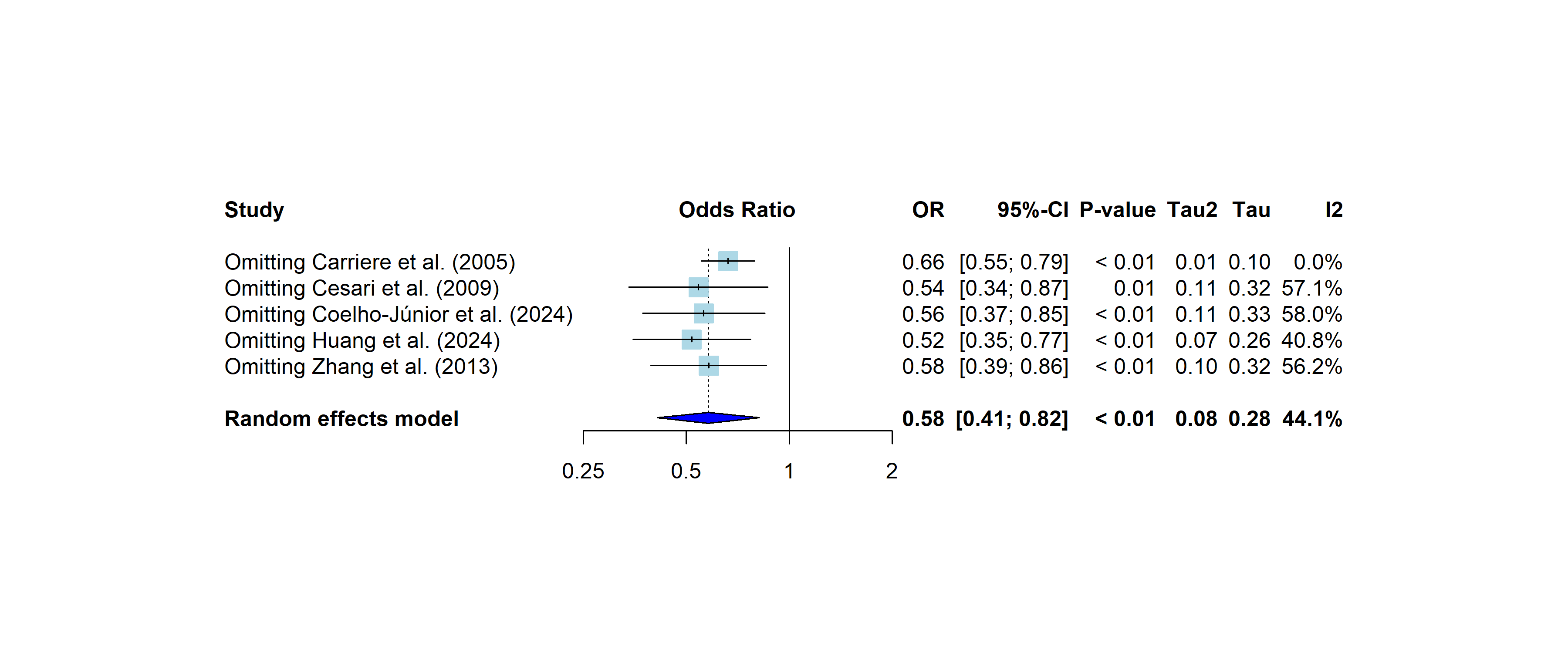


**Figure S32.** Sensitivity analysis (leave-one-out method) of the pooled odds ratios for the association between 5-repetition chair-stand performance (best vs. worst category) and incident disability (disability activities of daily living, functional mobility, ambulatory status).


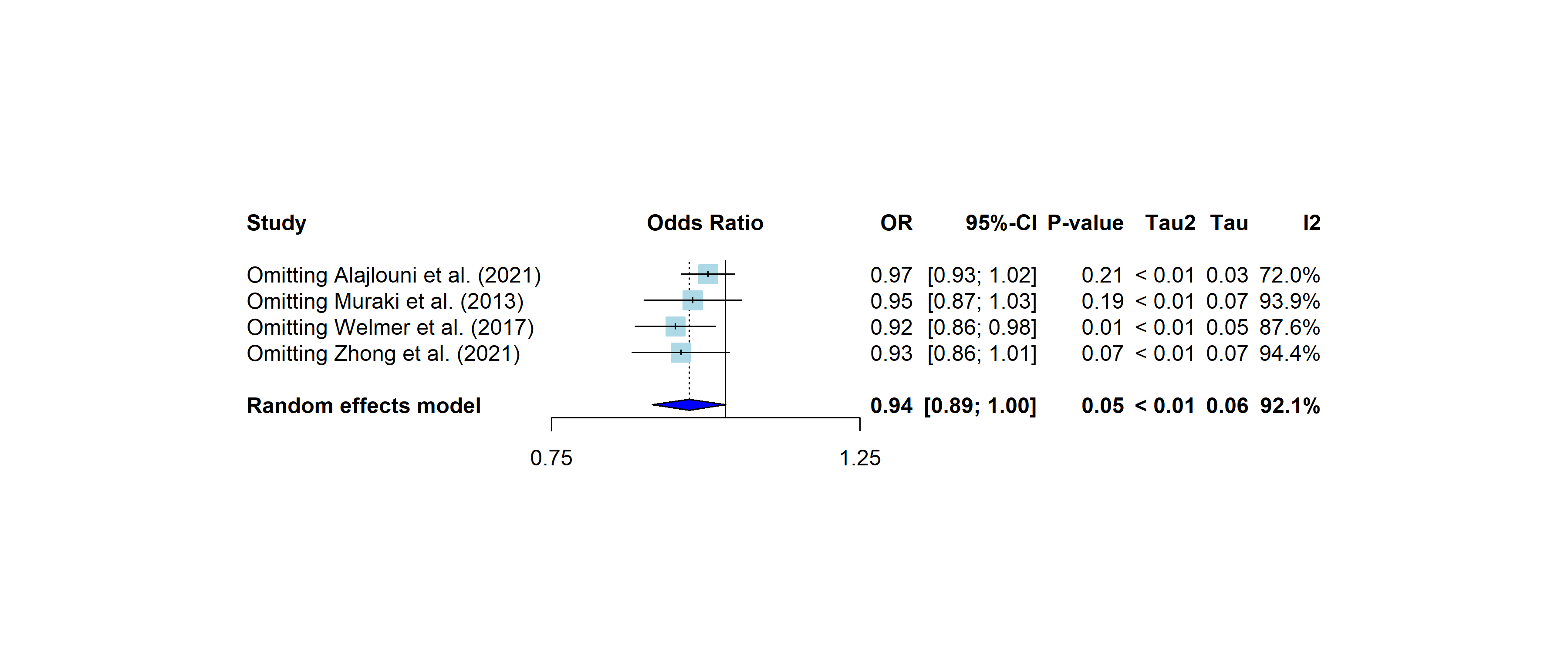


**Figure S33.** Sensitivity analysis (leave-one-out method) of the pooled odds ratios for the association between 5-repetition chair-stand performance (per 1-second decrease) and incident musculoskeletal impairment.
